# Abstract

**DOI:** 10.1111/aogs.14608

**Published:** 2023-12-11

## 1. GLOBAL HEALTH

## Association between maternal factors and risk of metabolic‐associated fatty liver disease in offspring

### **Hanna De Ruyter** ^1^, Linnea Aitokari^1,2^, Tiina Jääskeläinen^3,4^, Heini Huhtala^5^, Hannele Laivuori^1,3^, Kalle Kurppa^1,2,6^

#### ^1^Tampere Center for Child, Adolescent and Maternal Health Research, Tampere University and Tampere University Hospital, Tampere, Finland; ^2^Celiac Disease Research Center, Tampere University, Tampere, Finland; ^3^Medical and Clinical Genetics, University of Helsinki and Helsinki University Hospital, Helsinki, Finland; ^4^Department of Food and Nutrition, University of Helsinki, Helsinki, Finland; ^5^Faculty of Social Sciences, Tampere University, Tampere, Finland; ^6^University Consortium of Seinäjoki, Seinäjoki, Finland

**Introduction/Purpose:** Due to steep increase of obesity, metabolic‐associated fatty liver disease (MAFLD) has become the most common chronic hepatic condition in children and adolescents. Maternal and pregnancy‐related factors have been implicated in the development of metabolic disturbances in offspring, but data on MAFLD remain scarce.

**Methods:** Data of 460 overweight and obese children aged 2–16 years was collected for a case–control study. MALFD was defined as alanine aminotransferase >2× upper limit of normal. Children with and without MAFLD were compared regarding various maternal factors and pregnancy‐related complications. Children with and without MAFLD were compared regarding various maternal factors and pregnancy‐related complications. Multivariate regression analysis was used to study associations by including factors that were significant (*P* < 0.1) in univariate analysis or suggested by literature.

**Results:** Median age of the study children was 11.8 (quartiles 9.1–14.2) years and 44% were girls. Children with MAFLD (17.8%) were significantly older (12.7 vs 11.6 years, *P* = 0.002) than those without MAFLD, whereas the two groups did not differ in BMI‐Z score or gender. In a multivariable model considering child‐related (age and BMI‐Z at present, sex) and maternal factors (pregestational overweight, smoking, gestational hypertension and preeclampsia), child's older age (odds ratio 1.16, 95% confidence interval (CI) 1.06–1.27), maternal smoking (2.04, 95% CI 1.19–3.50), gestational hypertension (3.41, 95% CI 1.07–10.9) and preeclampsia (2.84, 95% CI 1.13–7.18) were associated with MAFLD. There was no significant association between MAFLD and maternal BMI, birth anthropometrics or perinatal complications.

**Conclusions:** Maternal smoking, gestational hypertension and preeclampsia were associated with MAFLD among overweight and obese offspring. Further prospective studies are needed to confirm causality.

## Beneficial effect of breastfeeding on offspring bodyweight and asthma: consequence or preexisting risk profile?

### **Borghild Farsund** ^1^, Rønnaug Ødegård^2,3^, Eszter Vanky^3,4^, Melanie Rae Simpson^5^

#### ^1^Faculty of Medicine and Health Sciences, Norwegian University of Science and Technology, Trondheim, Norway; ^2^Center of Obesity Research, Department of Surgery, St. Olav's Hospital, Trondheim, Norway; ^3^Department of Clinical and Molecular Medicine, Faculty of Medicine and Health Sciences, Norwegian University of Science and Technology, Trondheim, Norway; ^4^Department of Obstetrics and Gynecology, St. Olav's Hospital, Trondheim, Norway; ^5^Department of Public Health and Nursing, Faculty of Medicine and Health Sciences, Norwegian University of Science and Technology, Trondheim, Norway

**Introduction/Purpose:** Breastfeeding is reported to reduce the risk of overweight/obesity and asthma in offspring, but studies are conflicting. Maternal metabolic health is rarely accounted for in studies on breastfeeding and offspring health, despite its association with breastfeeding. We aimed to evaluate the role of maternal metabolic health on the association between breastfeeding and offspring overweight/obesity and asthma.

**Methods:** Using data from the Norwegian Mother, Father, and Child Cohort Study (MoBa) and the Medical Birth Registry of Norway (MBRN) on >18 000 mother–child dyads, we estimated the effect of different breastfeeding durations on the odds of overweight/obesity and asthma at age 3 and 7 in term‐born singletons, using logistic generalized estimating equations. We adjusted for traditionally included confounding factors and additionally for maternal metabolic factors, in partially and fully adjusted models.

**Results:** Before adjusting for confounders, longer breastfeeding durations were associated with lower odds of overweight/obesity at age 3 and 7. Breastfeeding appeared to have some protective effect against overweight/obesity at age 3 in the fully adjusted model. However, at 7 years, the effect of breastfeeding of any duration on overweight/obesity was negligible in the fully adjusted models, although we could not rule out either a negative or positive effect of breastfeeding. This was also the case for the relationship between breastfeeding and asthma at age 3 and 7.

**Conclusions:** The previously reported beneficial effects of breastfeeding on offspring overweight/obesity may be due to the mother's healthy preexisting metabolic state rather than breastfeeding per se. For asthma, maternal metabolic health appears to be of minor importance.

## A part of my life – the meaning of female genital mutilation and its health consequences: a qualitative study from Sweden

### **Bita Eshraghi** ^1^, Lena Marions^1^, Jonas Hermansson^2^, Vanja Berggren^3^

#### ^1^Department of Clinical Science and Education, Södersjukhuset, Karolinska Institutet, Stockholm, Sweden; ^2^Department of Research, Angered Hospital, SV‐Hospital Group, Gothenburg, Sweden; ^3^Department of Neurobiology, Caring Science and Society and Health (NVS), Karolinska Institutet, Stockholm, Sweden

**Introduction/Purpose:** Female genital mutilation (FGM) comprises all procedures that involve partial or total removal of the female genitalia or other injuries to them for non‐medical reasons. The aim of this study was to elucidate women's experiences of FGM and perceived consequences on their health.

**Methods:** A qualitative study was performed through interviews with eight women who had undergone FGM and lived in Sweden. The transcribed narratives were analyzed using content analysis.

**Results:** The findings were shown in two main‐categories. The first, *The meaning of living with FGM*, had sub‐categories: *A unique experience with mixed feelings and flashbacks*, *Ambivalence*, *Persuading culture but with culture clash*, and *Living with lifelong health consequences*. The latter included both *Challenges when peeing and at menstrual periods*, *The defibulation operation seen as a positive turning point*, *Lifelong learning on sexual enjoyment* and *Psychological suffering as consequences of others expecting negative health consequences*. The second main category: *The meaning of FGM in the encounter with the healthcare*, had five sub‐categories: *Feeling confirmed in the encounter with health care providers*, *Feeling ignored*, *Experiences of insulting attitudes* and *Feeling having no choice*.

**Conclusions:** To prevent the continuation of FGM after immigration, more studies about FGM and culture clashes after immigration is of importance. These findings indicate a need for increased knowledge of FGM among healthcare personnel. Improved feeling of participation in the caring planning could be recommended.

## Examining the quality of informed consent in intrapartum clinical trials: a prospective study in Uganda

### **Mehreen Zaigham** ^1,2,3^, Paola Del Cueto^3^, Lisa Bebell^4,5,6^, Kaitlyn James^3^, Rohini Dutta^1,3^, Bizu Gelaye^6,7,8^, Henry M. Lugobe^9^, Godfrey R. Mugyenyi^9^, Jessica E. Haberer^4,5,6^, Francis Bajunirwe^9^, Joseph Ngonzi^9^, Adeline A. Boatin^1,3,5,6^

#### ^1^Program of Global Surgery and Social Change, Harvard Medical School, Boston, MA, USA; ^2^Obstetrics & Gynecology, Skåne University Hospital, Malmö, Sweden and Department of Clinical Sciences Lund, Lund University, Lund, Sweden; ^3^Department of Obstetrics and Gynecology, Massachusetts General Hospital, Boston, MA, USA; ^4^Department of Medicine, Massachusetts General Hospital, Boston, MA, USA; ^5^Center for Global Health, Massachusetts General Hospital, Boston, MA, USA; ^6^ Harvard Medical School, Boston, MA, USA; ^7^The Chester M. Pierce, M.D. Division of Global Psychiatry, Massachusetts General Hospital, Boston, MA, USA; ^8^Department of Epidemiology, Harvard T. Chan School of Public Health, Boston, MA, USA; ^9^Department of Obstetrics and Gynecology, Mbarara University of Science and Technology, Mbarara, Uganda

**Introduction/Purpose:** Despite the ethical and scientific importance of obtaining quality informed consent in clinical trials, empirical data is lacking on how participants understand research‐related information, particularly in low‐ and middle‐income countries (LMICs). We evaluated the quality of consent among trial participants in Uganda to identify domains at risk of misunderstanding, and explore the impact of sociodemographic characteristics on understanding.

**Methods:** This is a sub‐study of a wireless vital‐sign monitoring trial after cesarean birth in Mbarara, Uganda. Participants were recruited from May 2021 to February 2022. After completing all parent trial procedures, including a baseline questionnaire, participants completed the Quality of Informed Consent (QuIC) survey to assess *objective* (QuIC‐A) and *subjective* (QuIC‐B) understanding of consent. Survey components were scored from 0 to 100, with 100 indicating complete understanding. Scores were compared using multivariate logistic regression.

**Results:** We analyzed 583 surveys. The mean QuIC‐A score was 60.0 (SD ±12.4). Low‐scoring components included the experimental nature of the research, data confidentiality, and voluntary participation (Figure 1). Higher scores were correlated with household income (*P* < 0.001). Consent obtained <30 min before birth was associated with a lower score (*P* < 0.01). Mean QuIC‐B score was 36.5 (SD ±25.8). Low‐scoring components included understanding of research purposes, confidentiality, and study contact information. QuIC‐B scores were strongly correlated with higher education level (*P* < 0.001).**Conclusions:** In the largest intrapartum study examining the quality of informed consent among LMIC participants, we found overall low levels of objective and subjective understanding of informed consent. Innovative approaches are needed to improve consent processes, particularly for participants with lower levels of education and from poorer households.

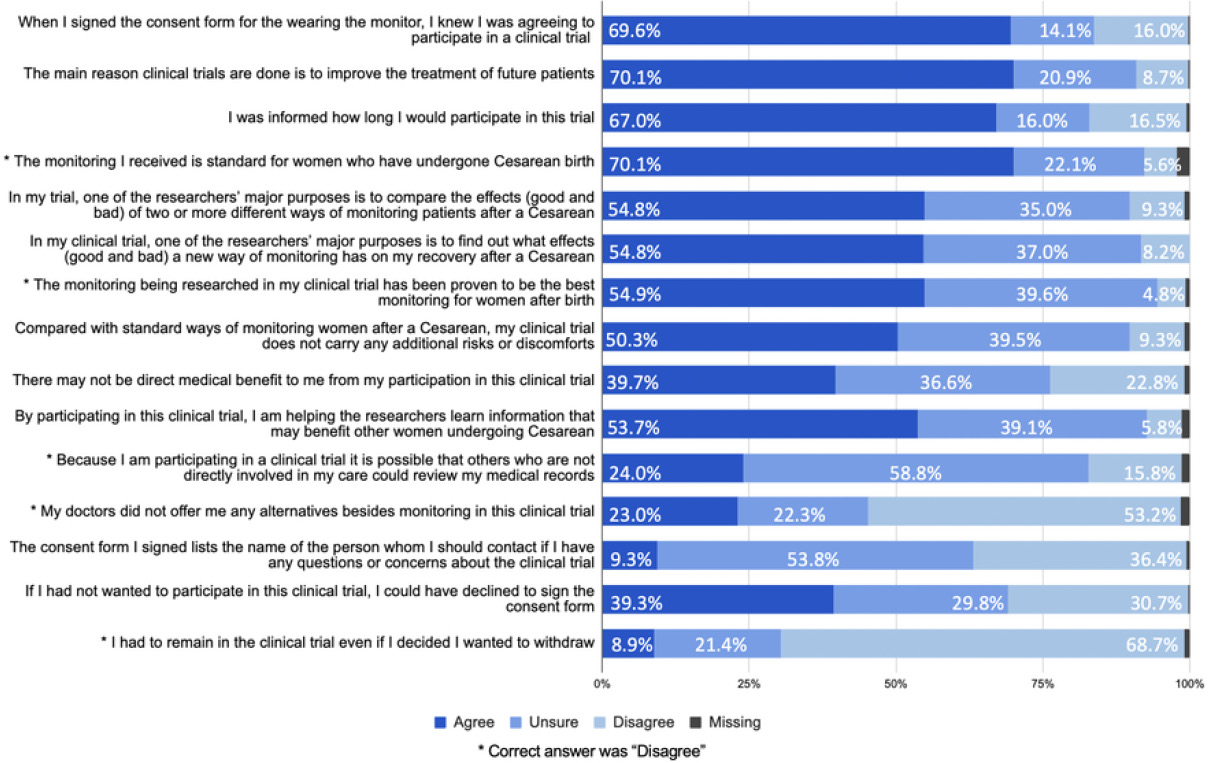

## Prioritized research questions regarding pregnancy‐related pelvic girdle pain

### **Helena Domeij** ^1^, Karin Rydin^1^, Irini Åberg^1^, Helen Elden^2,3^, Elin Naurin^4^, Annelie Gutke^5^

#### ^1^ SBU – Swedish Agency for Health Technology Assessment and Assessment of Social Services, Stockholm, Sweden; ^2^Department of Obstetrics and Gynecology, Region Västra Götaland, Sahlgrenska University Hospital, Gothenburg, Sweden; ^3^Institute of Health and Caring Sciences, Sahlgrenska Academy, University of Gothenburg, Gothenburg, Sweden; ^4^Department of Political Science, University of Gothenburg, Gothenburg, Sweden; ^5^Department of Health and Rehabilitation, Institute of Neuroscience and Physiology, Sahlgrenska Academy, University of Gothenburg, Gothenburg, Sweden

**Introduction/Purpose:** Pregnancy‐related pelvic girdle pain is characterized by pain in the pelvis and a reduced function capacity, foremost during the pregnancy, but also following childbirth. In severe cases, women with pregnancy‐related pelvic girdle pain experience high levels of discomfort due to which they require help with their daily activities. The aim of this project was to provide a top‐10 list of the most important research questions, according to patients with pregnancy‐related pelvic girdle pain, carers and clinicians.

**Methods:** The project used the James Lind Alliance (JLA) method. The project consisted of two parts: an inventory part where the research questions were identified, and a second part where the identified research questions were prioritized, resulting in a list of prioritized research questions ranked from 1 to 10.

**Results:** The most prioritized research question focused on the effects of a coherent course of care. In addition, socioeconomic effects, knowledge‐raising measures for clinicians, effects of preventive and treatment interventions, follow‐up after childbirth, attitudes in the society, as well as effects on the quality of life for the woman and her family, were included in the top 10 research questions.

**Conclusions:** The top 10 research questions that were considered the most prioritized in this project included treatment of pelvic girdle pain and effects on the patient's daily life.

## Screening for gestational diabetes mellitus (GDM) and co‐creation of a self‐care intervention in Vietnam – protocol for the VALID‐II study

### **Ditte Søndergaard Linde** ^1,2^, Tine M. Gammeltoft^3^, Vibeke Rasch^1,2^, Ib Bygbjerg^4^, Dan Meyrowitsch^4^, Thanh Duc Nguyen^5^, Ngọc Anh Thị Đặng^5^, Kien Dang Nguyen^6^, Aí Nguyen^5^, Hiếu Minh Lê^7^, Tran Huong^5^, Dung Kim Vu^5^, Tien Van Nguyen^5^, Jannie Nielsen^4^, Jens Søndergaard^8^, Christina A. Vinter^1,2^

#### ^1^Department of Gynecology & Obstetrics, Odense University Hospital, Odense, Denmark; ^2^Department of Clinical Research, University of Southern Denmark, Odense, Denmark; ^3^Department of Anthropology, University of Copenhagen, Copenhagen, Denmark; ^4^Department of Public Health, University of Copenhagen, Copenhagen, Denmark; ^5^Department of Public Health, Thai Binh University of Medicine and Pharmacy, Thai Binh, Vietnam; ^6^Department of Obstetrics and Gynecology, Thai Binh University of Medicine and Pharmacy, Thai Binh, Vietnam; ^7^Department of Internal Medicine, Thai Binh University of Medicine and Pharmacy, Thai Binh, Vietnam; ^8^Department of Public Health, University of Southern Denmark, Odense, Denmark

**Introduction/Purpose:** GDM is an increasing issue in low‐ and middle‐income countries with long‐term health consequences for mother and child. Yet little is known about the prevalence, risk factors and how best to reduce neonatal and maternal health consequences of GDM in such settings.

The aim of the VALID II study is to:

- Implement universal screening to determine the prevalence and risk factors of GDM
- Develop a “self‐care/informal support” intervention
- Improve maternal and neonatal health outcomes of GDM through the “self‐care/informal support” intervention

**Methods:** Pregnant women will be screened for GDM at a gestational age of 24–28 with a 75‐g 2 h oral glucose tolerance test and diagnosed according to the WHO 2013 criteria. The intervention will be co‐created with women diagnosed with GDM, their informal support persons and healthcare providers and tested in a parallel 2‐arm non‐randomized intervention study with a delayed start for the intervention group.

**Results:** Recruitment started in February 2023 in the Thai Binh Province in the North Delta in Vietnam. A total of 2000 pregnant women will be screened for GDM; an expected 400 women will be diagnosed with GDM, among which 200 will be enrolled into the control arm and 200 into the intervention arm.**Conclusions:** The VALID study is an international, cross‐disciplinary, capacity‐building research project supported by Danida.

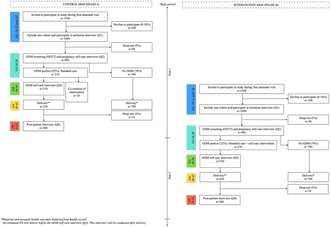

## 2. OBSTETRICS

## Lateral episiotomy or not in vacuum‐assisted delivery in nulliparous women to reduce obstetric anal sphincter injury: a randomized controlled trial

### Sandra Bergendahl^1^, Maria Jonsson^2^, Susanne Hesselman^3^, Victoria Ankarcrona^1^, Åsa Leijonhufvud^4^, Anna‐Carin Wihlbäck^5^, Tove Wallström^6^, Emmie Rydström^7^, Hanna Friberg^8^, **Sophia Brismar Wendel** ^1^

#### ^1^Department of Clinical Sciences, Danderyd Hospital, Karolinska Institutet, Stockholm, Sweden; ^2^Department of Women's and Children's Health, Uppsala Universitet, Akademiska Hospital, Uppsala, Sweden; ^3^Department of Women's and Children's Health, Center for Clinical Research Dalarna, Falu Hospital, Uppsala Universitet, Uppsala, Sweden; ^4^Department of Clinical Sciences Lund/Clinical Science Helsingborg, Helsingborg Hospital, Lunds Universitet, Lund, Sweden; ^5^Department of Clinical Sciences, Norrland University Hospital, Umeå Universitet, Umeå, Sweden; ^6^Department of Clinical Science and Education, South General Hospital, Karolinska Institutet, Stockholm, Sweden; ^7^Växjö Central Hospital, Region Kronoberg, Växjö, Sweden; ^8^Sahlgrenska University Hospital, Göteborg, Sweden

**Introduction/Purpose:** Nulliparous women giving birth with vacuum extraction (VE) are at increased risk of obstetric anal sphincter injury (OASIS). We aimed to investigate whether routine lateral episiotomy vs no episiotomy in VE in nulliparous women affected the prevalence of OASIS in a multicenter randomized controlled trial (EVA).

**Methods:** Nulliparous women with a singleton, live fetus in cephalic presentation were recruited in eight Swedish hospitals. Consenting women with indication for VE after gestational week 34 were randomized at 1:1 ratio to lateral episiotomy or no episiotomy. A lateral episiotomy was defined as cut at crowning, 1–3 cm from the midline, at 60° to the midline, and 3–5 cm long. The primary outcome was a clinical diagnosis of OASIS. In total, 710 women were needed to demonstrate a 50% reduction in OASIS from 12.4% to 6.2% with 80% power and *P* < 0.05. Secondary short‐term outcomes were blood loss, maternal pain postpartum and neonatal Apgar <7 at 5 minutes. Results are presented as proportions and relative risks with 95% confidence intervals.

**Results:** The inclusion was closed February 14, 2023, when we had included 714 nulliparous women with a singleton, live, cephalic fetus requiring VE after gestational week 34. The statistical analysis plan will be finalized in February 2023. Data lock is planned in March 2023. Analyses will take place in April 2023 and results are expected to be presentable in May 2023.

**Conclusions:** We aim to deliver an updated abstract with results in May 2023 and present the conclusion of the EVA trial at the NFOG Congress.

## Use of endoanal ultrasound in detecting obstetric anal sphincter injury immediately after birth

### **Malin Huber** ^1^, Charlotta Larsson^2^, Mathilda Harrysson^3^, Karin Strigård^2^, Jan‐P. Lehmann^4^, Pär Nordin^2^, Katarina Tunon^1^

#### ^1^Department of Clinical Sciences, Obstetrics and Gynecology, Umeå University, Umeå, Sweden; ^2^Department of Surgical and Perioperative Sciences, University of Umeå, Umeå, Sweden; ^3^Department of Obstetrics and Gynecology, Östersund Hospital, Östersund, Sweden; ^4^Department of Surgery, Östersund Hospital, Östersund, Sweden

**Introduction/Purpose:** Obstetric anal sphincter injury (OASI) complicates around 5% of deliveries in primiparas. The study objective was to assess the utility of three‐dimensional endoanal ultrasonography (3D‐EAUS) in the diagnosis of OASI.

**Methods:** The present study was designed to mirror screening settings with an unselected cohort of nulliparous women. All enrolled patients underwent clinical examination of the perineum by the caregiver, and 3D‐EAUS was conducted. Post‐processing of ultrasonography volume data was performed by an experienced colorectal surgeon who was blinded to all other data. The sensitivity, specificity, negative predictive value and positive predictive value of 3D‐EAUS in the diagnosis of OASI was evaluated. The trial is registered at ISCRTN: 18006769.

**Results:** A total of 680 scans were performed, of which 18.5% were judged as “non assessable”, leaving 554 assessable recordings. Sphincter defects were observed in 12.8% of all assessable recordings on 3D‐EAUS (*n* = 71). With clinical examination set as the reference standard, ultrasound sensitivity in the diagnosis of OASI was 30.4% and specificity was 87.9%. The negative predictive value was 96.7% and the positive predictive value was only 9.9%. Comments were left on 175 examinations, of which 74% referred to the management of the examination.

**Conclusions:** Using 3D‐EAUS in a maternity ward is demanding because staff generally have little experience in endoanal ultrasound. Acquisition of immediate postpartum optimal endoanal ultrasound images presents significant challenges that may affect image quality and interpretation. When 3D‐EAUS is performed to mirror screening settings, it adds no convincing diagnostic power to clinical examination in the diagnosis of OASI.

## Secondary fear of birth after instrumental vaginal delivery, Bergen birth study

### **Sindre Grindheim** ^1,2^, Johanne Kolvik Iversen^3^, Jørg Kessler^1,2^, Elham Baghestan^1,2^

#### ^1^Department of Obstetrics and Gynecology, Haukeland University Hospital, Bergen, Norway; ^2^Department of Clinical Science, University of Bergen, Bergen, Norway; ^3^Department of Obstetrics and Gynecology, Oslo University Hospital, Oslo, Norway

**Introduction/Purpose:** Instrumental vaginal delivery occurs in 19% of primiparae in Norway. Differences in maternal and neonatal outcome between vacuum and forceps have been studied. However, maternal experience of labor and development of secondary fear of childbirth (FOC) between the instruments is unknown. We aimed to determine whether choice of instrument affects the woman's risk of developing secondary FOC.

**Methods:** The Bergen birth study, a prospective observational study assessing maternal and neonatal outcome after instrumental vaginal delivery, included patients between June 2021 and February 2023.

950 women were included postpartum, evenly distributed between forceps, vacuum and spontaneous vaginal deliveries. Wijma birth experience score (WDEQ‐B) was completed in the first days after childbirth as well as preferred mode of delivery next pregnancy between cesarean delivery (CD), vaginal delivery (VD) or uncertain.

Mean WDEQ‐B score was calculated for each group and analyzed using ANOVA. Data will be adjusted to indication, duration of labor, analgesia, birth tears, Apgar score and transfer to neonatal intensive care unit, and analyzed accordingly.

**Results:** Preliminary results show no difference of mean WDEQ‐B score between spontaneous, vacuum assisted or forceps assisted deliveries (*P* = 0.483). There was no difference in preferred mode of delivery in the next pregnancy between the groups (*P* = 0.806). Women who reported vaginal delivery as the referred mode of delivery in future pregnancy had a trend for lower mean WDEQ score than women preferring CD or uncertain (*P* = 0.06).

**Conclusions:** Final data will be analyzed and presented at the NFOG Congress as inclusion is completed in February 2023.

## Duration of first stage of labor, severe perineal lacerations and maternal complications in the early postpartum period

### **Lisa Kogner** ^1^, Louise Lundborg^2^, Xingrong Liu^2^, Linnea Ladfors^2^, Mia Ahlberg^1,2^, Olof Stephansson^1,2^, Anna Sandström^1,2^

#### ^1^Department of Women's Health, Division of Obstetrics and Gynecology, Karolinska University Hospital, Stockholm, Sweden; ^2^Clinical Epidemiology Division, Department of Medicine, Solna, Karolinska Institutet, Stockholm, Sweden

**Introduction/Purpose:** Few studies have investigated how first‐stage labor duration impacts maternal outcomes. We aimed to study the association between first‐stage labor duration and maternal complications in the early postpartum period.

**Methods:** A population‐based cohort study including 159 459 term, singleton, vertex pregnancies with spontaneous onset or induction in Stockholm‐Gotland 2008–2020. The exposure was first‐stage labor duration, categorized in percentiles. Outcomes were: postpartum infection, urinary retention, hematoma in birth canal or ruptured sutures, and severe perineal lacerations (third or fourth degree). Poisson regression analysis was performed with 95% confidence interval (CI) with adjustments for confounders. To investigate the effect of second‐stage duration, mediation analysis was performed.

**Results:** Postpartum infection, urinary retention, hematoma or ruptured sutures, and severe perineal laceration affected 2.4%, 2.5%, 0.7% and 4.7% of all women in the study population, respectively. Hematoma or ruptured sutures were not associated with first‐stage duration. For all other outcomes, absolute risks increased with longer first stage in a dose–response fashion. Adjusted relative risks (aRR) increased with longer first stage, using <50th percentile as reference. In the ≥90th percentile category the aRR for postpartum infection were 1.64 (95% CI 1.46–1.84) in primiparous, 2.43 (95% CI 1.98–2.98) in parous with no previous cesarean delivery (CD) and 2.33 (95% CI 1.65–3.28) in parous with previous CD, respectively. The proportion mediated by second‐stage duration ranged from 33.4% to 36.9% for the outcomes in primiparous women.**Conclusions:** First‐stage duration is associated with maternal complications in the early postpartum period.

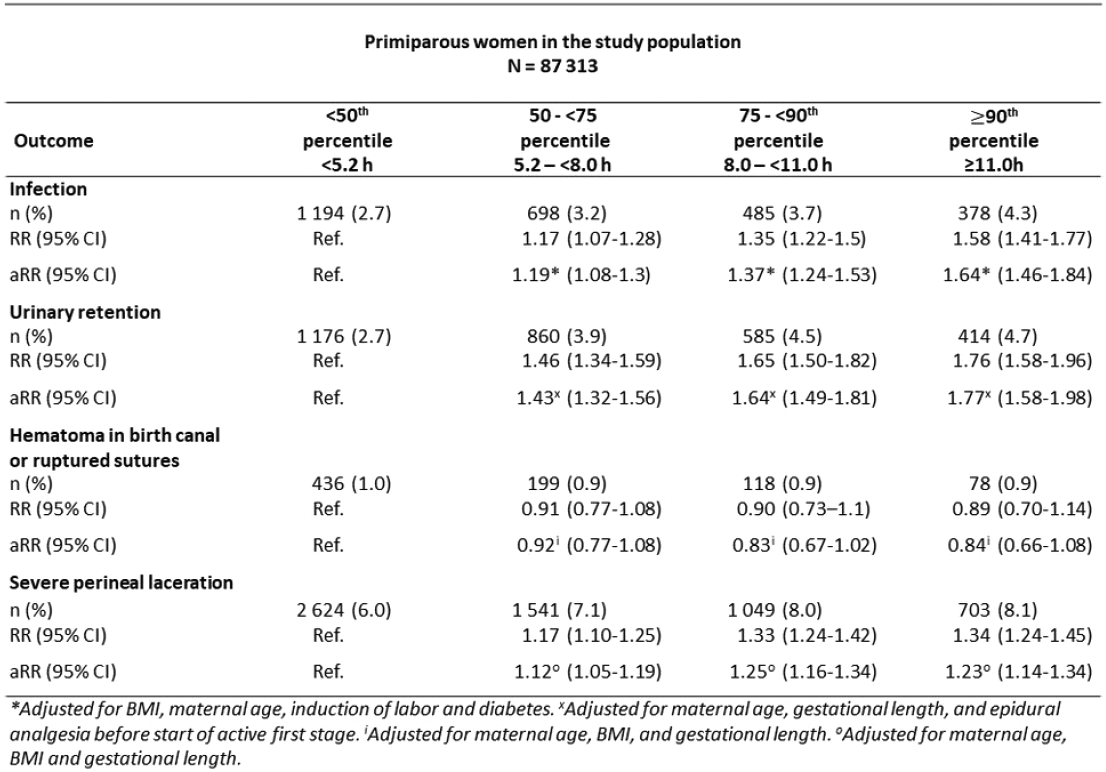

## Classification of intrapartum cesarean deliveries: a prospective nationwide study in Norway – The NOR‐WHY cesarean study

### **Jørg Kessler** ^1,2^, Ferenc Macsali^1,3^, Janne Rossen^4^, Christian Tappert^5^, Michael Robson^6^

#### ^1^Department of Obstetrics and Gynecology, Haukeland University Hospital, Bergen, Norway; ^2^Department of Clinical Medicine, University of Bergen, Bergen, Norway; ^3^National Institute of Health, the Medical Birth Registry of Norway, Oslo, Norway; ^4^Department of Obstetrics and Gynecology, Sørlandet Hospital, Kristiansand, Norway; ^5^Department of Obstetrics and Gynecology, St. Olav University Hospital, Trondheim, Norway; ^6^National Maternity Hospital, Dublin, Ireland

**Introduction/Purpose:** There is no consensus on classification of indications for cesarean delivery (CD) and none of the systems described previously fulfill the criteria of usefulness, robustness, simplicity and reproducibility. The aim of this study was to verify a classification system of intrapartum CD (Figure 1) using information about the indication for delivery, the progress of labor and the use of oxytocin.

**Methods:** Prospective observational study on singleton term deliveries in cephalic presentation with spontaneous or induced labor, February–August 2017 in Norway. Study collaborators from each unit received education and training before data collection. All CD were classified according to the system (Figure 1) based on the partograph, use of oxytocin, interpretation of fetal heart rate and linked to routine data collected to the Medical Birth Registry of Norway.

**Results:** The participating 25 units accounted for 82% of all deliveries in Norway. A total of 19 134 women went into labor and 1498 (7.8%) of these were delivered by CD and classified according to the system. CD for fetal indication (no oxytocin) was more common in induced than spontaneous labor. CD for dystocia related to inefficient uterine action and treated with oxytocin occurred more often in induced than spontaneous labor. The prevalence of CD due to dystocia and efficient uterine action (malposition or cephalopelvic disproportion) was generally low.**Conclusions:** The system for classification of intrapartum CD was successfully tested in a nationwide study and may become a valuable objective tool for analyzing and optimizing management of labor.

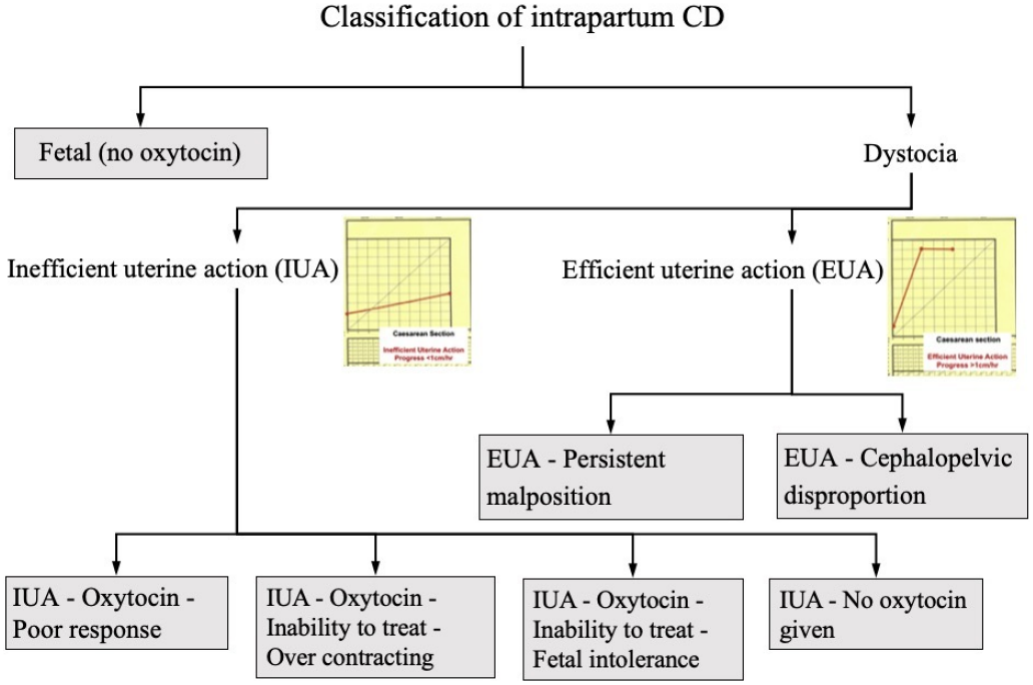

## External validation of the California maternal quality care collaborative postpartum hemorrhage risk assessment tool in Sweden

### **Linnea Ladfors** ^1^, Olof Stephansson^1,2^

#### ^1^Clinical Epidemiology Division, Department of Medicine ‐ Solna, Karolinska Institutet, Stockholm, Sweden; ^2^Department of Women's Health, Division of Obstetrics, Karolinska University Hospital, Stockholm, Sweden

**Introduction/Purpose:** The postpartum hemorrhage (PPH) risk assessment tool, introduced by the California Maternal Quality Care Collaborative (CMQCC), is used in US delivery wards, at admission and during labor, to identify women at risk for PPH. The tool classifies women as low, medium and high‐risk based on factors such as prior cesarean, bleeding disorders, placental disorders and anemia. Before considering implementation of this risk assessment tool in clinical practice, a validation in a Swedish setting is crucial.

**Methods:** We included 335 153 singleton births recorded in The Stockholm Gotland Perinatal cohort between 2008 and 2020. Information on maternal and obstetric characteristics and diagnostic codes for maternal conditions were collected and women were classified as low, medium and high‐risk according to the CMQCC risk assessment tool. Rates of PPH (estimated blood loss >1000 mL) in each group were calculated and the sensitivity and specificity of the tool in identifying women who later developed PPH were estimated.

**Results:** The PPH rate was highest in women classified as high‐risk (13.8%; 95% CI 13.4–14.2) and lowest in those classified as low‐risk (6.2%; 95% CI 6.1–6.3). The sensitivity of the tool in identifying women at risk (medium or high) for PPH was 53.6% (95% CI 52.9–54.2) and the specificity was 59.0% (95% CI 58.8–59.2).

**Conclusions:** In this Swedish cohort, almost 50% of women who developed PPH had been classified as low‐risk using the CMQCC risk assessment tool. Policymakers should be aware of the limited accuracy of this tool before implementation in clinical practice.

## Risk of obstetric anal sphincter tear among primiparous women with a history of female genital mutilation, giving birth in Sweden

### **Bita Eshraghi** ^1^, Jonas Hermansson^2^, Vanja Berggren^3^, Lena Marions^1^

#### ^1^Department of Clinical Science and Education, Södersjukhuset, Karolinska Institutet, Stockholm, Sweden; ^2^Department of Research, Angered Hospital, SV‐Hospital Group, Gothenburg, Sweden; ^3^Department of Neurobiology, Caring Science and Society and Health (NVS), Karolinska Institutet, Stockholm, Sweden

**Introduction/Purpose:** Female genital mutilation (FGM) is a practice that includes partial or total removal of the external female genitalia for non‐medical reasons. FGM has been associated with several adverse obstetrical complications. However, the obstetric outcomes in high‐income countries are not clear. The aim of this study was to compare the risk of obstetric anal sphincter injury (OASI) among primiparous women, with and without a history of FGM, giving birth in Sweden.

**Methods:** A population‐based cohort‐study based on data from the Swedish Medical Birth Register during 2014–2018. The study included primiparous women with singleton term pregnancies. We compared the risk, using multivariable logistic regression, of OASI between women with a diagnosis of FGM and women without a diagnosis of FGM. Secondary outcomes included episiotomy and instrumental vaginal delivery.

**Results:** A total of 239 486 primiparous women with a term singleton pregnancy were identified. We included 1444 women with a diagnosis of FGM and 186 294 women without a diagnosis of FGM in our analysis. We found that women with a diagnosis of FGM had a significantly increased odds ratio (OR) of OASI (OR 2.69, 95% CI 2.14–3.37) compared with women without a diagnosis of FGM. We also found an association between FGM and instrumental delivery as well as the use of episiotomy.

**Conclusions:** Women with a history of FGM have an almost tripled risk of OASI in comparison with women without FGM, when giving birth in a Swedish setting.

## Reduced fetal movements and perinatal outcome: a retrospective population‐based cohort study after implementation of new national guidelines

### **Emma D.Y.E. Jonasson** ^1^, Lars Ladfors^1^, Ylva Carlsson^1^

#### ^1^Department of Obstetrics, Sahlgrenska University Hospital/Östra, Sahlgrenska Academy, University of Gothenburg, Gothenburg, Sweden

**Introduction/Purpose:** New national guidelines regarding reduced fetal movements (RFM) were implemented in 2016 in order to decrease the stillbirth rate in Sweden. The aim of this study was to examine whether the new guidelines had improved the stillbirth rate and perinatal outcome.

**Methods:** This retrospective cohort study included all singleton births from January to December 2018 at Sahlgrenska University Hospital. Data from the Swedish Pregnancy Register and the hospital's visitor register was used. Perinatal and maternal outcome were compared between patients who had and those who had not experienced RFM. The study also contains analysis of stillbirth rate between 2009 and 2020. A similar study from 2003 was used for comparison.

**Results:** 9790 pregnancies were eligible. 2066 patients had experienced RFM, which contributed to 2763 visits. The number of visits due to RFM had increased by 690% between 2003 and 2018. A higher probability for induction was found in the group with RFM, relative risk (RR) 1.71 (1.51–1.93) and odds ratio (OR) 1.53 (1.33–1.76) when adjusted for risk factors for stillbirth. The stillbirth rate did not decrease from 2009 to 2020.

**Conclusions:** We could not find an improved perinatal outcome in patients with RFM. The probability of induction of labor was higher in the group who experienced RFM. There was no decrease in the incidence of stillbirths at Sahlgrenska University Hospital between the years 2009 and 2020, despit the implementation of an extensive control program for RFM.

## Frequency of fetal blood sampling, delivery mode and neonatal outcome after revised CTG classification and updated lactate meter in Sweden

### Klara Gröndal^1^, Erika Gyllencreutz^1,2^, Kari Johansson^1^, Stina Wretler^1,3^, **Malin Holzmann** ^1,3^

#### ^1^Karolinska Institutet, Stockholm, Sweden; ^2^Östersund Hospital, Östersund, Sweden; ^3^Karolinska University Hospital, Stockholm, Sweden

**Introduction/Purpose:** A revised CTG‐classification system was implemented in Sweden during 2017. Simultaneously, an updated version of the most commonly used lactate meter was shown to measure 50% higher than the previous version, necessitating new cutoff levels at fetal blood sampling (FBS). We hypothesized that the revised CTG‐classification might decrease FBS rates, that FBS at more severe CTG‐patterns might increase rates of scalp blood acidemia and neonatal outcome among sampled fetuses, but not in the entire population, and that the higher lactate values might have an impact on delivery mode.

**Methods:** A retrospective cohort study of labors in Stockholm‐Gotland during 2014–2015 and 2018–2019, including singleton pregnancies >34 weeks, cephalic presentation, with spontaneous or induced start of labor.

**Results:** There were 28 841 and 30 190 births during the two periods. FBS decreased from 11.8% to 8.1% (*P* < 0.05). The proportion of acidemia at FBS increased from 7% to 13.5% (*P* < 0.05) among sampled fetuses and from 0.8% to 1.0% in the total population (*P* < 0.05). Immediate cesareans increased among sampled fetuses (2.3% vs 3.3%, *P* = 0.02) but not among non‐sampled fetuses (0.5% vs 0.6%, *P* = 0.74). The incidence of Apgar scores <4 at 5 minutes remained similar after FBS (0.4% vs 0.5%, *P* = 0.66) but increased among non‐sampled newborns (0.1% vs 0.16%, *P* = 0.03). HIE 2–3 was not increased in either group (*P* > 0.15).

**Conclusions:** Rates of FBS was lower after implementation of the revised Swedish CTG‐classification. Scalp blood acidemia among sampled fetuses was more frequent but fetal acidemia at FBS in the total population was still low. Low Apgar scores increased among newborns both with and without FBS, but HIE 2–3 rates were unchanged.

## Prediction of fetal acidemia before delivery using machine‐learning in an experimental sheep model

### **Ganesh Acharya** ^1,2^, Jonas Johnson^1,2^, Mervi Haapsamo^3^, Juulia Lantto^3^, Heikki Huhta^3^, Juha Voipio^4^, Juha Räsänen^4,5^

#### ^1^Division of Obstetrics and Gynecology, CLINTEC, Karolinska Institutet, Stockholm, Sweden; ^2^Center for Fetal Medicine, Karolinska University Hospital, Stockholm, Sweden; ^3^Oulu University Hospital, Oulu, Finland; ^4^University of Helsinki, Helsinki, Finland; ^5^Helsinki University Hospital, Helsinki, Finland

**Introduction/Purpose:** Prediction of fetal acid–base status using CTG during labor remains elusive even with the so called “physiological interpretation” based on various fetal heart rate (FHR) parameters with computer assistance. We aimed to investigate whether blood pressure (BP) can predict fetal arterial pH better than CTG.

**Methods:** We performed intermittent umbilical cord compression on chronically instrumented fetal sheep near term. The fetal ECG and arterial BP were recorded continuously at 1000 Hz. Arterial blood sampling was performed at baseline and intermittently during the experiment until the pH dropped below 7.0, when an emergency cesarean section was performed to deliver the fetus.

We used the MATLAB Classification Learner app to test and train different machine learning models to classify data and chose the K‐Nearest Neighbors model for supervised learning. The model with the best accuracy in validation and testing was selected to predict fetal arterial pH.

**Results:** The difference between the highest BP during cord occlusion and the lowest BP immediately after release of occlusion had the best accuracy (76%), with an AUC of 0.82 for predicting fetal acidemia (pH <7.1).

The use of CTG‐based parameters, ie FHR, short‐term variation (STV) and stress index (SI), had an accuracy of 73%, with an AUC of 0.72. Combining BP with CTG‐based parameters slightly increased the accuracy to 79%, with an AUC of 0.78, but did not improve the BP‐based prediction model.

**Conclusions:** Fetal BP response appears to reflect fetal acid‐base status better than the CTG alone. Development of technology to measure fetal BP could improve intrapartum fetal monitoring.

## Offspring anthropometrics associated with maternal thyroid function in pregnant women with polycystic ovary syndrome treated with metformin or placebo

### **Anastasia Trouva** ^1,2^, Michael Alvarsson^1^, Jan Calissendorff^1^, Bjørn Olav Åsvold^3,4,5^, Dorina Ujvari^6,7^, Angelica Lindén Hirschberg^6,8^, Eszter Vanky^9,10^

#### ^1^Department of Molecular Medicine and Surgery, Karolinska Institutet, Stockholm, Sweden; ^2^Department of Internal Medicine, Section of Diabetes and Endocrinology, Södersjukhuset, Stockholm, Sweden; ^3^K.G. Jebsen Center for Genetic Epidemiology, Department of Public Health and Nursing, NTNU, Norwegian University of Science and Technology, Trondheim, Norway; ^4^ HUNT Research Center, Department of Public Health and Nursing, NTNU, Norwegian University of Science and Technology, Levanger, Norway; ^5^Department of Endocrinology, Clinic of Medicine, St. Olav's Hospital, Trondheim University Hospital, Trondheim, Norway; ^6^Department of Women's and Children's Health, Karolinska Institutet, Stockholm, Sweden; ^7^Department of Microbiology, Tumor and Cell Biology, National Pandemic Center, Center for Translational Microbiome Research, Karolinska Institutet, Solna, Sweden; ^8^Department of Gynecology and Reproductive Medicine, Karolinska University Hospital, Stockholm, Sweden; ^9^Department of Clinical and Molecular Medicine, Faculty of Medicine and Health Sciences, Norwegian University of Science and Technology, Trondheim, Norway; ^10^Department of Obstetrics and Gynecology, St. Olav's Hospital, Trondheim University Hospital, Trondheim, Norway

**Introduction/Purpose:** Polycystic ovary syndrome (PCOS) is linked to adverse pregnancy and neonatal outcomes. Even small variations in thyroid function within the normal range may influence fetal growth. Metformin‐treatment of pregnant women with PCOS resulted in a smaller decrease of fT4 compared with placebo and exposure in utero affected offspring anthropometrics, leading to larger head size. Our aim was to investigate whether newborn anthropometrics are associated with maternal thyroid function in PCOS pregnancies.

**Methods:** Post‐hoc analyses of two randomized controlled trials, in which pregnant women with PCOS were treated with metformin or placebo, from first trimester to delivery. Maternal serum levels of thyroid stimulating hormone (TSH) and free thyroxine (fT4) were measured at gestational weeks 5–12, 19, 32 and 36 in 309 pregnancies. The mean *z*‐scores of birthweight, birth length and head circumference were estimated in offspring. The associations between TSH and fT4 and offspring anthropometrics were studied using linear regression.

**Results:** There were no associations between maternal TSH and fT4 and newborn head circumference. In the first trimester, fT4 was inversely associated with birthweight (*b* = −0.082, *P* = 0.022) and birth length (*b* = −0.086, *P* = 0.048). The change in fT4 during pregnancy correlated positively to both birthweight and length (*b* = 0.11, *P* = 0.007 and *b* = 0.10, *P* = 0.047 respectively).

**Conclusions:** In PCOS women, early pregnancy fT4 was inversely associated with offspring birthweight and birth length. A greater decrease in fT4 during pregnancy was associated with a lower birthweight and shorter birth length. Subclinical changes in maternal thyroid function may influence birth anthropometrics in babies born to women with PCOS.

## Tafoxiparin, a new drug candidate (phase II B) for cervical and uterine priming at planned delivery

### **Gunvor** **Ekman‐Ordeberg** ^1^ , Marie Blomberg^2^, Maria Jonsson^3^, Malin Engberg^4^, Kati Tihtonen^5^, Leena Rahkonen^6^

#### ^1^Department of Obstetrics and Gynecology, Karolinska Institute, Stockholm, Sweden; Department of Women's and Children's Health, Uppsala University Hospital, Uppsala, Sweden; ^2^Department of Obstetrics and Gynecology, Department of Biomedical and Clinical Sciences Linköping University, Linköping, Sweden; ^3^Department of Women's and Children's Health, Uppsala University Hospital, Uppsala, Sweden; ^4^Department of Obstetrics and Gynecology, Skövde, Sweden; ^5^Department of Obstetrics and Gynecology, Tampere University Hospital, Tampere, Finland; ^6^Department of Obstetrics and Gynecology HUS, University of Helsinki, Helsinki, Finland

**Introduction/Purpose:** Labor induction is increasing with the aim to avoid complications. Tafoxiparin, a new drug candidate (Dilafor AB), which has been shown to induce increase in cytokines in human cervical cells and to increase Ca‐influx in myometrial cells, has shown effects on both the cervical ripening and the myometrium. In an explorative study (Phase IIa) a positive effect on cervical ripening was registered in nulliparous women planned for induction. Today we present our positive data of labor priming with tafoxiparin in a Phase IIb study.

**Methods:** A randomized, double‐blind, placebo‐controlled, parallel‐group proof of concept study. 174 nulliparous women with unripe cervix (≤4p) planned for induction. Up to seven injections of tafoxiparin/placebo were given subcutaneously and cervical state was checked every day. In women not starting labor spontaneously, amniotomy and oxytocin were used with a ripe cervix and a balloon was applied if unripe. Endpoint: Efficacy of tafoxiparin on cervical ripening change.

**Results:** The cervical ripening rate during up to 7 days was highly significant compared with placebo (*P* = 0.008). The time from start of treatment until ripe cervix/spontaneous onset of labor was also significant after tafoxiparin (*P* = 0.009) and there were significantly fewer instrumental deliveries (*P* = 0.025). Operation delivery for fetal distress was halved in the active group. No hyperstimulation problem was reported. Tafoxiparin does not pass the placenta.

**Conclusions:** Tafoxiparin is a result of 40 years of translational research and new drug candidate with different mechanism of action compared with earlier drugs resulting in cervical and myometrial priming without hyperstimulation and negative fetal influence. Tafoxiparin may be optimal in the outpatient setting.

## Variations in the use of oxytocin for augmentation of labor – a population‐based cohort study

### **Karin Johnson** ^1,2^, Kari Johansson^1,2^, Charlotte Elvander^1^, Sissel Saltvedt^2,3^, Malin Edqvist^1,2^

#### ^1^Clinical Epidemiology Division, Department of Medicine Solna, Karolinska Institutet, Solna, Sweden; ^2^Department of Women's Health and Health Professions, Karolinska University Hospital, Stockholm, Sweden; ^3^Department of Women's and Children's Health, Department of Medicine Solna, Karolinska Institutet, Solna, Sweden

**Introduction/Purpose:** Oxytocin for augmentation of labor is a commonly used medical intervention with potentially harmful side‐effects. In Sweden, there is variation in the use of oxytocin between regions but also between obstetric units. The aim of this study was to investigate whether maternal and fetal characteristics or hospital size can explain the variation in use of oxytocin for augmentation of labor between obstetric units in Sweden.

**Methods:** This population‐based cohort study using data from the Swedish Pregnancy Register included all women in Robson group 1 and 3 giving birth from January 1, 2018 until December 31, 2021. Main outcome measure was the use of oxytocin for augmentation of labor (y/n). Exposures were divided into groups related to maternal and fetal characteristics and obstetric unit size, ie based on annual birth rate. Obstetric units were categorized into three groups according to their use of oxytocin: low (46%–57%), intermediate (58%–62%) or high (63%–73%). Logistic regression models were used to estimate risk ratios (RR) with 95% confidence intervals (CI) for oxytocin augmentation stratified by Robson groups.

**Results:** The final cohort comprised 242 218 women. Of these, 63 440 (59%) women in Robson group 1 and 21 252 (16%) women in Robson group 3 had their labors augmented with oxytocin. When comparing the obstetric units, units with high augmentation rates used more oxytocin regardless of maternal or fetal characteristics or obstetric unit size. Adjusted analyses will be completed, and the full results will be presented at the conference.

**Conclusions:** Conclusions will be presented at the conference.

## Niche development after closure of cesarean uterotomy by double‐suture or single‐suture technique (NICUM): a randomized trial

### **Julie Glavind** ^1,2^, Axel Forman^1^, Maria Judi Johansen^1^, Niels Uldbjerg^1^, Lone Hvidman^1^, Isil Pinar Bor^1,2^

#### ^1^Institute for Clinical Medicine, Department Obstetrics and Gynecology, Aarhus University Hospital, Aarhus, Denmark; ^2^Department of Obstetrics and Gynecology, Regional Hospital of Randers, Randers, Denmark

**Introduction/Purpose:** The cesarean uterotomy closure technique is speculated to be an important factor in the development of a cesarean scar niche. In this study, we compared two different techniques for uterotomy closure on the occurrence of a cesarean scar niche.

**Methods:** The study was a randomized controlled trial performed in a tertiary Danish hospital with inclusion of singleton pregnant women with a scheduled, first cesarean section. We randomized participants to two different uterotomy closure techniques: (1) double‐suture technique using a non‐locked, transverse suture involving only the myometrium followed by a second non‐locked longitudinal suture or (2) single‐suture technique with a non‐locked transverse suture alternating between deep myometrial and superficial serosa stitches. The primary outcome was the rate of cesarean scar niches >2 mm deep at 6 months’ follow‐up using transvaginal hysterosalinography. Secondary outcomes were residual myometrial thickness and scar niche measurements. Analyses were by intention‐to‐treat.

**Results:** In all, 230 women were randomized; 115 for each intervention group. The follow‐up rate was >70% in both groups. The primary outcome occurred in 42/81 women (52%) after double‐suture vs 35/81 women (43%) after single‐suture closure (*P* = 0.35). Women in the single‐suture group had significantly more additional stitches to the uterotomy closure. There were no differences in ultrasonograpic or maternal clinical secondary outcomes between the two groups.

**Conclusions:** Cesarean uterotomy closure with double‐ or single‐suture technique may have a similar risk of cesarean scar niche development.

Registration: www.clinicaltrials.gov ID NCT02410395.

## Reduced cesarean section rates in Norway and associations with perinatal and neonatal health

### **Katariina Laine** ^1,2^, Aase S. D. Pay^3,4^, Branka M. Yli^5^

#### ^1^Institute of Clinical Medicine, Faculty of Medicine, University of Oslo; ^2^Norwegian Research Center for Women's Health, Oslo University Hospital, Oslo, Norway; ^3^Department of Gynecology and Obstetrics, Bærum Hospital, Vestre Viken HF, Bærum, Norway; ^4^Department of Nursing and Health Promotion, Oslo Metropolitan University, Oslo, Norway; ^5^ Department of Obstetrics, Oslo University Hospital, Oslo, Norway

**Introduction/Purpose:** A continuously increasing cesarean section (CS) rate has been observed across the world, whereas the CS rates in Norway have been reduced in the last 15 years. The aim of this study was to assess the time trends in CS rates in Norway, and the association with fetal and neonatal health.

**Methods:** The study population consisted of 1 329 795 births registered in the Medical Birth Registry Norway in 1999–2021. CS rates are presented in the Robson Ten classification system; with statistical significance in confidence intervals.

**Results:** The overall CS rate increased from 12.9% in 1999 to 16.7% in 2008. After 2008 the CS decreased to 15.0% in 2021. Significant changes were observed after 2008:

Robson group 1: CS rate was reduced by 17.9% from 8.9% in 2008 to 7.3% in 2021.

Robson 2a: CS rate was reduced by 22.2% from 26.6% to 20.7%.

Robson 3: CS rate was reduced by 30% from 2.0% in 2009 to 1.4%.

Robson 4a: CS rate was reduced with 33% from 6.6% to 4.4%.

Robson 6–10: CS rate was reduced by 4%–20%.

Mean maternal age increased from 29.5 years to 31.5, the proportion of women >35 years increased from 13,6 in 1999 to 22.3%. Labor induction increased from 10.5% to 28.4%.

Gestational diabetes prevalence increased from 0.7% to 6.3%.

Perinatal mortality was reduced from 7.6‰ to 4.1‰. Early neonatal mortality was reduced from 7.1‰ to 3.5‰.

**Conclusions:** A significant reduction in CS rates was observed from 2008 to 2021. Norwegian obstetricians and midwives have managed to keep the CS rates stable and low without compromising the fetal outcome.

## The impact of sepsis on maternal deaths in the Nordic countries

### **Sedina Atic Kvalvik** ^1,2^, Lill Trine Nyfløt^3,4^, Hanna Åmark^5^, Rikke Beg Helmig^6^, Outi Äyräs^7^, Svein Rasmussen^2^, Steinar Skrede^2,8^, Elham Baghestan^1,2^

#### ^1^Department of Obstetrics and Gynecology, Haukeland University Hospital, Bergen, Norway; ^2^Department of Clinical Science, University of Bergen, Bergen, Norway; ^3^Norwegian National Advisory Unit on Women's Health, Oslo University Hospital, Oslo, Norway; ^4^Department of Obstetrics, Drammen Hospital, Drammen, Norway; ^5^Department of Obstetrics, Sødersjukhuset, Stockholm, Sweden; ^6^Department of Obstetrics, Aarhus University Hospital, Skejby, Denmark; ^7^Department of Obstetrics and Gynecology, Helsinki University Central Hospital; ^8^Department of Medicine, Haukeland University Hospital, Bergen, Norway

**Introduction/Purpose:** Sepsis is an increasingly important cause of maternal morbidity and mortality in both low‐/middle income and high‐income countries. Maternal mortality reviews from the US and the UK show that up to 70% of the maternal deaths due to sepsis were preventable, indicating a potential for reducing the number of deaths in other countries as well. Both early identification and expeditious treatment of sepsis patients have been shown to improve outcomes in this patient category. Through audits on maternal deaths, we aim to prevent future maternal deaths from sepsis by identifying suboptimal factors in treatment.

**Methods:** We identified maternal deaths in the Nordic countries from 2005 to 2021 through linked registers and direct reporting from hospitals. The national audit groups in each country performed case assessments based on hospital records, classified the cause of death, and evaluated the standards of clinical care provided. Potential improvements to care were evaluated.

**Results:** We identified 300 maternal deaths in the study period, giving a maternal mortality rate of 6.03 deaths per 100 000 live births. Sepsis accounted for approximately 10% of the maternal deaths. Data regarding the sepsis cases are being analyzed. The results and learning points, as well as conclusions, are to be presented at NFOG August 2023.

**Conclusions:** Learning points for the improvement in handling of maternal sepsis will be prepared.

## Swedish Perinatal Core Outcome Set (SPeCOS) for management of labor and delivery at or near term

### **Julia Savchenko** ^1,2^, Malin Asp^3^, Marie Blomberg^4^, Charlotte Elvander^5^, Anna Hagman^6^, Cecilia Pegelow Halvorsen^2,7^, Pelle Lindqvist^1,2^, Maria Nelander^8^, Béatrice Skiöld^9,10^, Sophia Brismar Wendel^11,12^

#### ^1^Department of Obstetrics and Gynecology, Stockholm South General Hospital (Södersjukhuset), Stockholm, Sweden; ^2^Department of Clinical Science and Education, Stockholm South General Hospital (Södersjukhuset), Karolinska Institutet, Stockholm, Sweden; ^3^Swedish Infant Death Foundation, Stockholm, Sweden; ^4^Department of Obstetrics and Gynecology and Department of Biomedical and Clinical Sciences, Linköping University, Linköping, Sweden; ^5^Clinical Epidemiology Division, Department of Medicine, Karolinska Institutet, Stockholm, Sweden; ^6^Prenatal Care and Reproductive Health Unit, Gothenburg, Sweden; ^7^Neonatal Unit, Sachs’ Children and Youth Hospital, Södersjukhuset, Stockholm, Sweden; ^8^Department of Women's and Children's Health, Uppsala University, Uppsala, Sweden; ^9^Department of Neonatology, Karolinska University Hospital, Stockholm, Sweden; ^10^Department of Women's and Children's Health, Karolinska Institutet, Stockholm, Sweden; ^11^Department of Obstetrics and Gynecology, Danderyd Hospital, Stockholm, Sweden; ^12^Department of Clinical Sciences, Karolinska Institutet, Danderyd Hospital, Stockholm, Sweden

**Introduction/Purpose:** Choice of perinatal outcome measures is crucial for obstetric research, audit and clinical counseling. Unwarranted variation in reported outcomes impairs research synthesis, validity and implementation, clinical benchmarking and longitudinal comparisons. Our aim was to develop a short‐term perinatal (fetal/neonatal) Core Outcome Set (COS) to be used in research and quality assurance of management of labor and delivery at or near term.

**Methods:** The project was prospectively registered in the Core Outcome Measures in Effectiveness Trials database. A list of potential outcomes was created based on systematic review of studies on peripartum management at or near term, including timing and type of labor onset, intrapartum care and mode of delivery. It was entered into a two‐round Delphi survey with predefined consensus criteria. Participants included clinicians, researchers, patient representatives and other stakeholders. A consensus meeting was held to reach a final agreement.

**Results:** The response rate for Delphi rounds was 82.1% and 92.7%, respectively. Seventeen outcomes were included in the final COS, reflecting mortality, health or morbidity, including asphyxia, central nervous system status, infection, neonatal resuscitation and admission, breastfeeding and mother–infant interaction, operative delivery due to fetal distress, as well as birthweight and gestational age. Two of these outcomes were suggested by patient representatives.**Conclusions:** The Swedish Perinatal COS (SPeCOS) study involved a broad circle of participants and reached consensus on a minimal set of perinatal outcomes to be reported in future studies on management of labor and delivery at or near term, regardless of specific population or condition. This could improve obstetric research, clinical practice, audit, and comparisons in childbirth care.

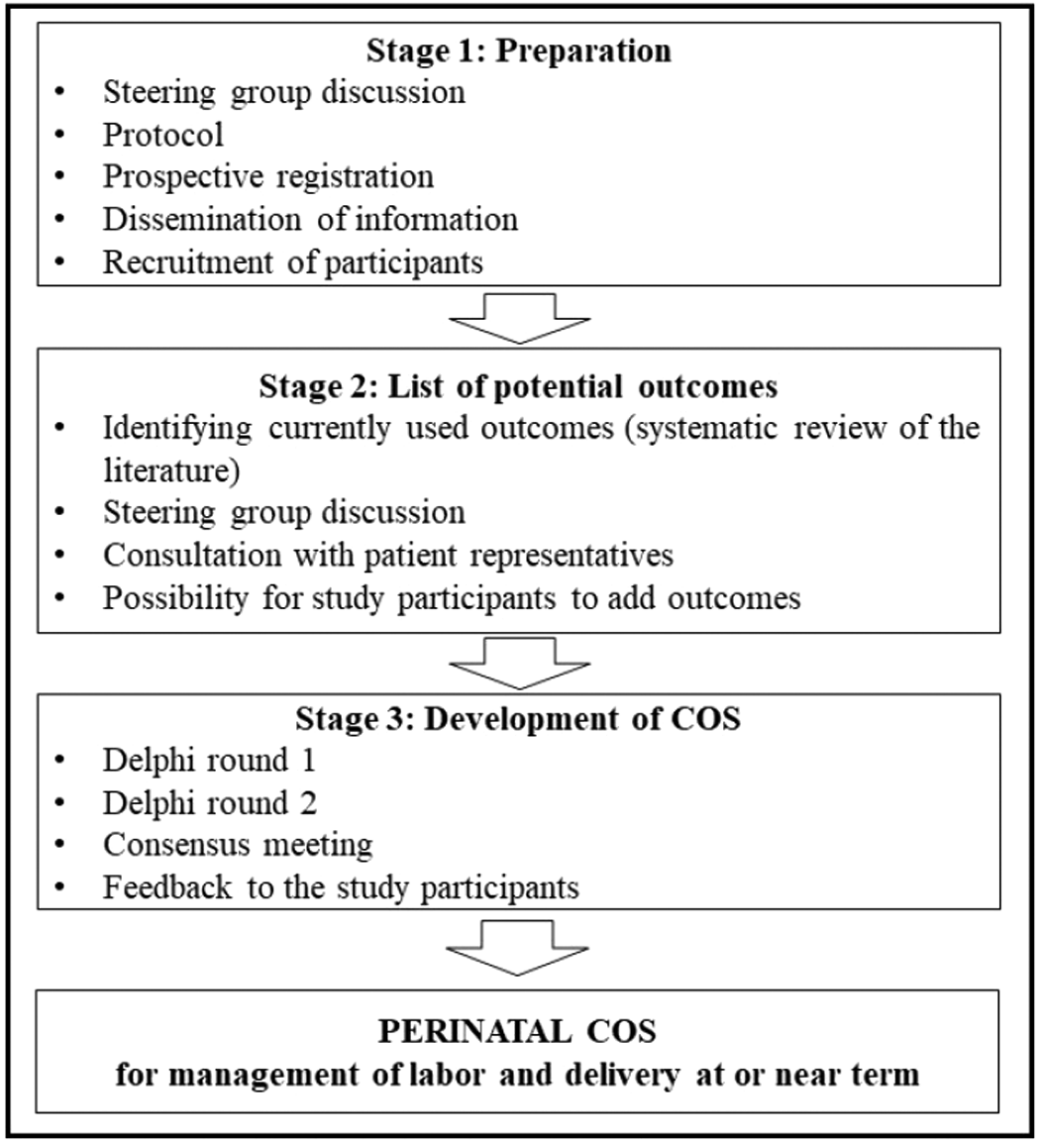

## Sleep duration and sleep loss during pregnancy – a longitudinal cohort study

### **Linda Aukia** ^1^, Juulia Paavonen^2,3^, Linnea Karlsson^4,5,6^, Hasse Karlsson^4,6,7^, Päivi Polo‐Kantola^1^

#### ^1^Department of Obstetrics and Gynecology, Turku University Hospital and University of Turku, Turku, Finland; ^2^Pediatric Research Center, Child Psychiatry, University of Helsinki and Helsinki University Hospital, Helsinki, Finland; ^3^National Institute for Health and Welfare, Helsinki, Finland; ^4^ FinnBrain Birth Cohort Study, Turku Brain and Mind Center, Institute of Clinical Medicine, University of Turku, Turku, Finland; ^5^Department of Child Psychiatry, Turku University Hospital and University of Turku, Turku, Finland; ^6^Center for Population Health Research, Turku University Hospital and University of Turku, Turku, Finland; ^7^Department of Psychiatry, Turku University Hospital and University of Turku, Turku, Finland

**Introduction/Purpose:** Sleep disturbances are common during pregnancy and previous literature has proposed an association between sleep disturbances and adverse pregnancy and delivery outcomes. Yet, the longitudinal studies evaluating sleep duration and sleep loss in multiple measure points during pregnancy are sparse.

**Methods:** A cohort of 3067 women was enrolled from FinnBrain Birth Cohort Study. Self‐reported sleep duration and sleep loss, the latter calculated from preferred sleep need and actual sleep duration, were measured four times during pregnancy in gestation week (gw) 14, 24 and 34, and at delivery. Associations between sleep and age, body mass index, marital status, parity, education, smoking, as well as depressive and anxiety symptoms were evaluated.

**Results:** Sleep duration was longest in early pregnancy, decreasing until delivery (7.92 to 7.75 hours, *P* < 0.001). Proportion of short sleepers (<6 hours) increased from 1.5% to 6.0% (*P* < 0.001). Sleep loss remained quite stable, only in late pregnancy was the mean sleep loss (55.2 minutes) lower compared with the other pregnancy points (*P* < 0.001). The proportion of women with notable sleep loss (>2 hours) increased until delivery (14.3%, *P* < 0.001). Older, multiparous and more depressive women slept less throughout pregnancy (*P* = 0.017, *P* < 0.001 and *P* < 0.001). Women with higher BMI were more likely to sleep <6 hour in mid‐ and late pregnancy. Concerning sleep loss, multiparous, more depressive and women with higher BMI reported more sleep loss (*P* < 0.001, *P* < 0.001 and *P* = 0.048, respectively).

**Conclusions:** Sleep duration decreased in the course of pregnancy and notable sleep loss increased during the last month before delivery. Depressive symptoms were notably associative factors for sleep.

## Cesarean section on maternal request

### Ellika Andolf

#### Department of Clinical Sciences, Karolinska Institutet, Danderyd University Hospital, Stockholm, Sweden

**Introduction/Purpose:** To investigate the somatic risks for mother and child of planned cesarean section (CS) without medical indication, to perform a qualitative analysis of experiences among women and staff concerning CS and to conduct health economic analyses.

**Methods:** The report was conducted in accordance with the Cochrane handbook and the PRISMA statement. Short‐term maternal complications were retrieved from Swedish registry data. The certainty of results was assessed using GRADE and GRADE‐CERQual for qualitative methodology.

**Results:** The somatic results are based on 49 non‐randomized studies with a control group of intended vaginal delivery (VD). Maternal risk after CS was higher, although rare and potentially more serious than after VD. Lowered risks with CD included less damage to the pelvic floor. For the child, there were increased risks for complications with CS such as breathing difficulties at birth, NICU admission and later asthma and diabetes (low certainty of evidence). No lowered risks were found.

Qualitative results are based on 13 articles. Results differed between the two groups: women associated CS with lower risks than VD, thought they had the right to demand a CS and wanted their request to be accepted, whereas staff highlighted the importance of support.

Planned VD leads to lower costs for hospital care and somatic health gains, estimated difference at about 75–93 million SEK annually.

See also www.sbu.se/343

**Conclusions:** CS conferred higher risks for mother and child as well as higher costs. Psychologic effects and risks occuring with VD only were not studied.

## Metformin treatment during PCOS pregnancies and its impact on maternal infections and offspring allergy and eczema

### Mariell Ryssdal^1,2^, **Johanne Eikeland Skage** ^1,2^, Anders Hagen Jarmund^1,2^, Liv Guro Engen Hanem^1,3^, Tone Shetelig Løvvik^1,3^, Guro F. Giskeødegård^4^, Ann‐Charlotte Iversen^1,2,3^, Eszter Vanky^1,3^

#### ^1^Department of Clinical and Molecular Medicine, Norwegian University of Science and Technology (NTNU), Trondheim, Norway; ^2^Center of Molecular Inflammation Research (CEMIR), NTNU, Trondheim, Norway; ^3^Department of Obstetrics and Gynecology, St. Olav's Hospital, Trondheim University Hospital, Trondheim, Norway; ^4^K.G. Jebsen Center for Genetic Epidemiology, Department of Public Health and Nursing, NTNU, Trondheim, Norway

**Introduction/Purpose:** Polycystic ovary syndrome (PCOS) is an endocrine disorder with implications for pregnancy and long‐term health for both mother and child. Metformin, an anti‐diabetic drug, has been shown to reduce pregnancy complications in PCOS pregnancies and alter long‐term cardiometabolic health in the offspring, but immune‐related clinical outcomes remain unexplored. This study aimed to determine the effect of metformin on immune‐related clinical outcomes in pregnant women with PCOS and their offspring.

**Methods:** Two randomized controlled trials, PregMet and PregMet2 (*n* = 646), were analyzed post‐hoc to examine the effects of metformin vs placebo on pregnant women with PCOS throughout pregnancy. Offspring from the PregMet study (*n* = 153) were followed up at 8 years of age. Self‐reported maternal infections in pregnancy and the incidence of allergy, asthma and eczema in the children were compared.

**Results:** Women treated with metformin had fewer overall infections in pregnancy (risk ratio (RR) = 0.76, 95% CI 0.64–0.91, *P* = 0.002). Accordingly, the prevalence of viral infections was reduced (RR = 0.77, 95% CI 0.62–0.96, *P* = 0.03), but reduction in bacterial infections did not reach statistical significance (RR = 0.82, 95% CI 0.59–1.15, *P* = 0.25). Children exposed to metformin in utero had a significantly higher prevalence of allergy (RR = 3.46, 95% CI 1.19–10.02, *P* = 0.01) and eczema (RR = 2.39, 95% CI 1.28–4.46, *P* = 0.004), but not asthma (RR = 1.39, 95% CI 0.56–3.46, *P* = 0.48), at 8 years of age compared with those exposed to placebo.

**Conclusions:** Metformin exposure in pregnancy seems to reduce maternal infections and increase the prevalence of allergy and eczema in the offspring at 8 years of age.

## Neonatal outcome following metformin‐treated gestational diabetes mellitus: a prospective population‐based cohort study

### **Johanna Molin** ^1^, Magnus Domellöf^1^, Christel Häggström^2^, Itay Zamir^1^, Eva Östlund^3^, Marie Bixo^1^

#### ^1^Department of Clinical Sciences, Umeå University, Umeå, Sweden; ^2^Northern Registry Center, Department of Public Health and Clinical Medicine, Umeå University, Umeå, Sweden; ^3^Department of Clinical Sciences and Education, Södersjukhuset, Karolinska Institute, Stockholm, Sweden

**Introduction/Purpose:** Most studies evaluating neonatal outcome following metformin‐treated gestational diabetes mellitus (GDM) have in analyses included 30%–50% requiring supplemental insulin to achieve normoglycemia. Few studies have evaluated outcome against diet–only‐treated GDM. We aimed to study effects of metformin exposure (GDM‐M) separately from metformin with supplemental insulin (GDM‐MI) and evaluate neonatal outcome compared with insulin‐treated (GDM‐I) and diet–only‐treated GDM (GDM‐D).

**Methods:** We performed a Swedish prospective population‐based cohort study including 16 628 mother–infant pairs from 16 405 pregnancies complicated by GDM, 2019–2021. The cohort was linked to national and health quality registers to retrieve individual‐level data. Exposure was mode of GDM treatment. Main outcome measures were neonatal hypoglycemia, hyperbilirubinemia, respiratory morbidity, birth asphyxia morbidity, NICU admittance and gestational age at birth.

**Results:** GDM‐M had **a** significantly lower prevalence of neonatal hypoglycemia, hyperbilirubinemia and respiratory morbidity compared with GDM‐I, and a lower prevalence of respiratory morbidity and birth asphyxia morbidity compared with GDM‐D (Table 1).

GDM‐MI had a significantly lower prevalence of neonatal hypoglycemia, and hyperbilirubinemia compared with GDM‐I; there was no difference in prevalence of any outcome compared with GDM‐D. Pregnancy continued beyond gestational week 40+6 in 7.3% of GDM‐M and 14.1% of GDM‐D (*P* < 0.001).**Conclusions:** Metformin treatment not requiring supplemental insulin to achieve normoglycemia is associated with lower risk of adverse neonatal outcome compared with both insulin‐treated and diet only‐treated GDM. The lower risk of birth asphyxia morbidity in GDM‐M compared with GDM‐D was unexpected and was not entirely explained by difference in prevalence of prolonged gestation beyond week 40+6. Equal attention to the intensity of intrapartum fetal monitoring should be ensured in diet–only‐treated and pharmacologically treated GDM in clinical practice.

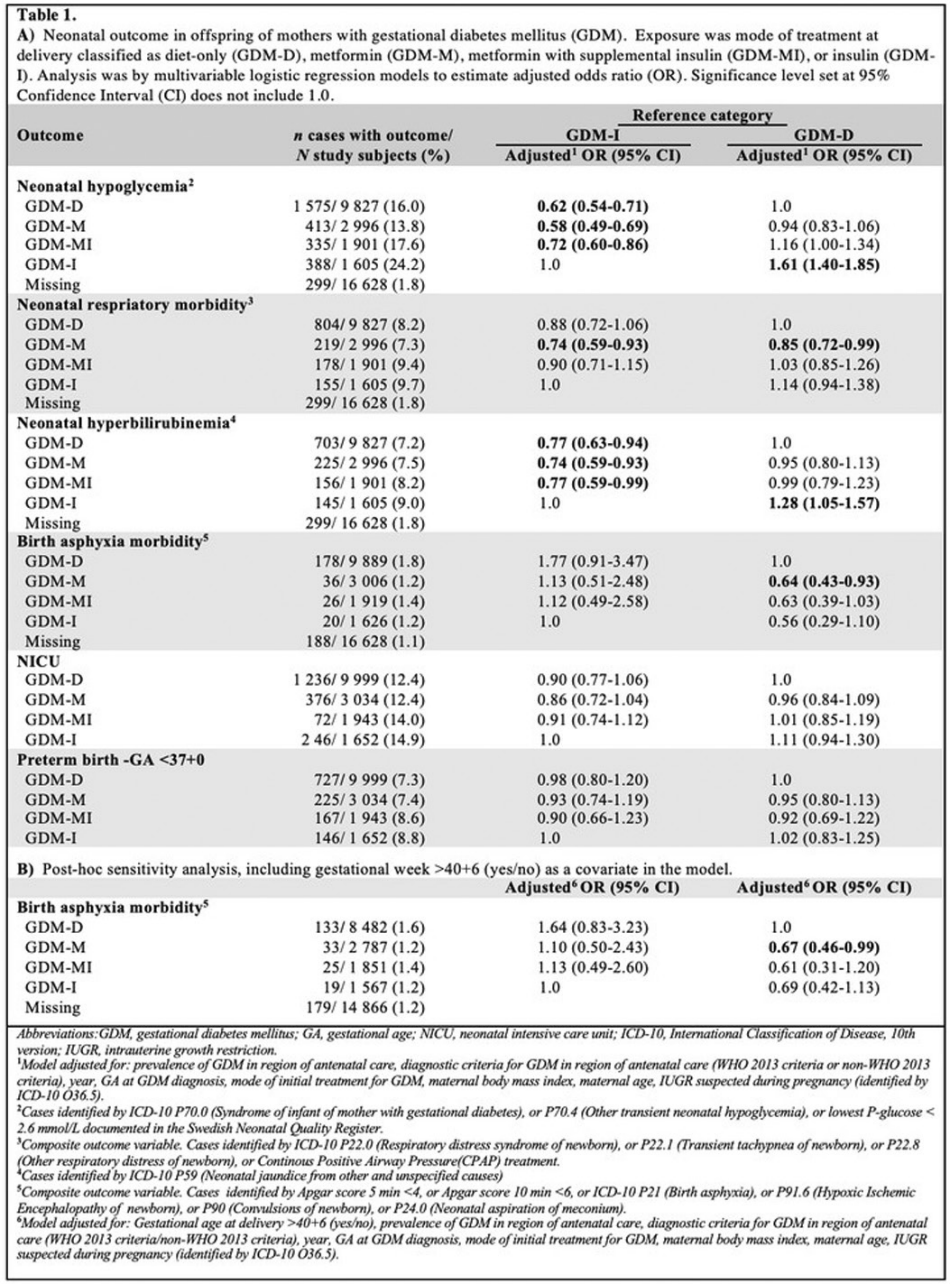

## Decision threshold for Kryptor SFLT‐1/PLGF ratio in women with suspected preeclampsia: retrospective study in a routine clinical setting

### **Lise Lotte Torvin Andersen** ^1^, Annemarie Helt^1^, Lene Sperling^1,2^, Martin Overgaard^2,3^

#### ^1^Department of Obstetrics and Gynecology, Odense University Hospital, Odense, Denmark; ^2^Department of Clinical Research, University of Southern Denmark, Odense, Denmark; ^3^Department of Clinical Biochemistry and Pharmacology, Odense University Hospital, Odense, Denmark

**Introduction/Purpose:** The objective was to evaluate predictive performance and optimal decision threshold of the Kryptor sFlt‐1/PlGF ratio when implemented for routine management of women presenting with symptoms of preeclampsia.

**Methods:** Observational retrospective study of a cohort of 501 women with suspected preeclampsia after 20 weeks’ gestation. Women referred to the maternity ward for observation of preeclampsia had an sFlt‐1/PlGF ratio test included in the routine diagnostic workup. Maternal and offspring characteristics data included maternal risk factors, outcomes, delivery mode and indication for suspected preeclampsia. Biochemical measurements to determine sFlt‐1/PlGF were performed using the BRAHMS/Kryptor sFlt‐1/PlGF immunoassays. Results were analyzed by area under curve‐receiver‐operating characteristic (AUC‐ROC).

**Results:** Preeclampsia occurred in 150/501 (30%) of symptomatic women with an sFlt‐1/PlGF ratio determined before the time of diagnosis. AUC‐ROC for diagnosis of early‐onset preeclampsia within 1 and 4 weeks was respectively 0.98 (95% CI 0.96–1.00) and 0.95 (95% CI 0.92–0.98). For late‐onset preeclampsia, predictive performance within 1 and 4 weeks was lower: 0.90 (95% CI 0.85–0.94) and 0.85 (95% CI 0.80–0.90), respectively. The optimal single sFlt‐1/PlGF threshold for all preeclampsia and late‐onset preeclampsia within 1 and 4 weeks was 66. The negative and positive predictive values to rule out and rule in developing preeclampsia within 1 week was 96% and 70%, respectively.

**Conclusions:** The Kryptor sFlt‐1/PlGF ratio is a useful clinical tool ruling out and ruling in preeclampsia within 1 week. Prediction within 4 weeks is superior for early‐onset preeclampsia. A single decision threshold of 66 is indicated for use in clinical routine.

## Stroke recurrence and subsequent pregnancy outcome after pregnancy‐associated stroke

### **Liisa Verho** ^1,2^, Minna Tikkanen^3^, Outi Äyräs^2^, Karoliina Aarnio^1^, Kirsi Rantanen^1^, Aino Korhonen^1^, Anna Richardt^1^, Hannele Laivuori^4,5,6^, Mika Gissler^7,8^, Petra Ijäs^1^

#### ^1^Neurology, University of Helsinki and Helsinki University Hospital, Helsinki, Finland; ^2^Obstetrics and Gynecology, University of Helsinki, Helsinki University Hospital, Helsinki, Finland; ^3^Obstetrics and Gynecology, University of Helsinki, Helsinki, Finland; ^4^Medical and Clinical Genetics, University of Helsinki and Helsinki University Hospital, Helsinki, Finland; ^5^Institute for Molecular Medicine Finland, Helsinki Institute of Life Science, University of Helsinki, Helsinki, Finland; ^6^Department of Obstetrics and Gynecology, Tampere University Hospital and Tampere University, Faculty of Medicine and Health Technology, Center for Child, Adolescent, and Maternal Health Research, Tampere, Finland; ^7^Department of Knowledge Brokers, Finnish Institute for Health and Welfare, Helsinki, Finland; ^8^Region Stockholm, Academic Primary Health Care Center, Stockholm, Sweden and Karolinska Institutet, Department of Molecular Medicine and Surgery, Stockholm, Sweden

**Introduction/Purpose:** Pregnancy‐associated stroke (PAS) is a life‐threatening emergency for the mother and the child. Our objective was to examine the PAS recurrence and outcomes of the subsequent pregnancies from women with a previous PAS in comparison with matched controls.

**Methods:** All women with a PAS in Finland from 1987 to 2016 (*n* = 235) and their matched controls (*n* = 694) were included in this population‐based retrospective cohort study. We identified all subsequent deliveries and induced and spontaneous abortions for women with a previous PAS and their controls from the Medical Birth Register and the Hospital Discharge Register until 2016. The number, pregnancy complications and neonatal outcomes of the subsequent pregnancies were compared. Patient records were studied for the PAS recurrence.

**Results:** Women with a previous PAS had fewer subsequent deliveries: 73 (31.1%) women had 122 deliveries in all, whereas 303 (47.3%) of the controls had 442 deliveries (age‐adjusted odds ratio [OR] 0.54, 95% confidence interval [CI] 0.38–0.76). Hypertensive disorders of pregnancy (HDP) (17.2% vs 5.7%, age‐adjusted OR 4.0, 95% CI 1.7–9.3), especially chronic hypertension (age‐adjusted OR 5.9, 95% CI 1.5–24.7), and any diabetes during pregnancy (24.6% vs 14.5%, age‐adjusted OR 2.0, 95% CI 1.1–3.8) were more common in cases. Regarding HDP, the difference between groups was explained by underlying factors such as index pregnancy HDP (multivariable OR 2.4, 95% CI 0.8–6.7). PAS recurred in four cases (5.5%).

**Conclusions:** Subsequent pregnancies of women with a history of PAS are more often complicated with hypertensive disorders of pregnancy and any diabetes during pregnancy. PAS recurrence risk is considerable.

## Polygenic risk scores for preeclampsia and hypertension reflect predisposition to both preeclampsia and later cardiovascular morbidity

### **Anna Kivioja** ^1^, Anni Kivelä^2^, Elli Toivonen^1,3^, Jaakko Tyrmi^1^, FINNPEC, Tiina Jääskeläinen^2,4^, Tanja Saarela^1,5^, Hannele Laivuori^1,2,3,6^

#### ^1^Center for Child, Adolescent and Maternal Health Research, Faculty of Medicine and Health Technology, Tampere University, Tampere, Finland; ^2^Medical and Clinical Genetics, University of Helsinki and Helsinki University Hospital, Helsinki, Finland; ^3^Department of Obstetrics and Gynecology, Tampere University Hospital, Tampere, Finland; ^4^Department of Food and Nutrition, University of Helsinki, Helsinki, Finland; ^5^Department of Clinical Genetics, Kuopio University Hospital, Kuopio, Finland; ^6^Institute for Molecular Medicine Finland (FIMM), Helsinki Institute of Life Science, University of Helsinki, Helsinki, Finland

**Introduction/Purpose:** Preeclampsia (PE) is known to be associated with cardiovascular diseases (CVDs) in later life. Whether PE is the first manifestation or an independent risk factor for CVD is not known. We investigated whether polygenic risk scores (PRS, a variable representing an individual's genetic risk for a disease) for high systolic and diastolic blood pressure (SBP‐PRS and DBP‐PRS, respectively) and preeclampsia (PE‐PRS) are associated with PE and its subtypes. Moreover, we evaluated the association between these PRSs and cardiovascular morbidity in later life.

**Methods:** The analyses were performed in the Finnish Genetics of Pre‐Eclampsia Consortium (FINNPEC) study cohort of 1371 PE and 1078 control women. PRS was developed using genetic associations found in two non‐related datasets (InterPregGen and Estonian biobank) totaling 10 979 preeclampsia cases. The data in FINNPEC were divided into percentiles to compare women with high PRS (above the 90th percentile) with other women. We assessed the incidence of PE, its subtypes and CVDs in the two groups. Data on CVDs were collected from the Finnish Hospital Discharge Register.

**Results:** PE‐PRS was associated with PE (odds ratio [OR] 1.5 [1.1–2.0], *P* = 0.014) and its form with severe symptoms. In addition, SBP‐PRS and DBP‐PRS were associated with PE as well as its forms with recurrence and severe symptoms. Furthermore, SBP‐PRS (OR 3.0 [1.7–5.6], *P* < 0.001) DBP‐PRS and PE‐PRS were associated with hypertensive diseases in later life.

**Conclusions:** The results suggest that PE‐PRS, SBP‐PRS and DBP‐PRS are associated with PE and its more severe subtypes. Moreover, these PRSs are associated with hypertensive diseases in later life. The analyses will be replicated in a larger FinnGen‐project.

## Associations between mid‐pregnancy cardiovascular biomarkers and preeclampsia

### **Paliz Nordlöf Callbo** ^1^, Katja Junus^1^, Katja Gabrysch^2^, Lina Bergman^1,3,4^, Inger Sundström Poromaa^1^, Susanne Lager^1^, Anna‐Karin Wikström^1^

#### ^1^Department of Women's and Children's Health, Uppsala University, Sweden; ^2^Uppsala Clinical Research Center, Uppsala, Sweden; ^3^Department of Obstetrics and Gynecology, Institute of Clinical Sciences, Sahlgrenska Academy, University of Gothenburg, Gothenburg, Sweden; ^4^Department of Obstetrics and Gynecology, Stellenbosch University, Cape Town, South Africa

**Introduction/Purpose:** Identifying women at risk of preeclampsia before clinical symptoms would be valuable for improved prevention, monitoring and treatment of the disorder. Cardiovascular disease and preeclampsia share similar pathophysiological entities and risk factors. In this nested case–control study, we aimed to explore the associations between mid‐pregnancy cardiovascular biomarkers and subsequent preeclampsia.

**Methods:** We included healthy women with singleton pregnancies donating blood in mid‐pregnancy (~18 weeks’ gestation). Cases were women with subsequent preeclampsia (*n* = 296, among whom 30 developed early‐onset preeclampsia [<34 weeks]). Controls were women who continued having healthy pregnancies (*n* = 333). Cases and controls were matched for early pregnancy body mass index and parity. We used the Proseek Multiplex cardiovascular II panel immunoassay, measuring 92 protein biomarkers in plasma. Boruta was used to estimate the associations between the biomarkers and preeclampsia development.

**Results:** The following eight biomarkers were associated with the development of preeclampsia (in descending order of importance in the Boruta analysis): matrix metalloproteinase‐12, natriuretic peptide B, placental growth factor, alpha‐l‐iduronidase, sortilin, growth hormone, kidney injury molecule and mitochondrial carbonic anhydrase 5A. In sub‐analyses, matrix metalloproteinase‐12, placental growth factor and lectin‐like oxidized low‐density lipoprotein receptor 1 were associated with early‐onset preeclampsia. In addition, late‐onset preeclampsia was associated with matrix metalloproteinase‐12, natriuretic peptide B, placental growth factor and alpha‐l‐iduronidase.

**Conclusions:** Metalloproteinase‐12 was consistently associated with both early‐ and late‐onset preeclampsia. Therefore, we suggest matrix metalloproteinase‐12 as a promising new preeclampsia biomarker. Moreover, this explorative study presents several new biomarkers that may be of value in enhancing prediction models for preeclampsia.

## Temporal trends in maternal preexisting chronic conditions during pregnancy in Sweden and Canada, 1999–2019

### **Louise Lundborg** ^1^, Sven Cnattingius^1^, Cande V. Ananth^2,3,4,5^, Neda Razaz^1^, K.S. Joseph^6,7^

#### ^1^Department of Medicine, Solna, Karolinska Institutet, Stockholm, Sweden; ^2^Division of Epidemiology and Biostatistics, Department of Obstetrics, Gynecology, and Reproductive Sciences, Rutgers Robert Wood Johnson Medical School, New Brunswick, NJ, USA; ^3^Cardiovascular Institute of New Jersey, Rutgers Robert Wood Johnson Medical School, New Brunswick, NJ, USA; ^4^Department of Biostatistics and Epidemiology, Rutgers School of Public Health, Piscataway, NJ, USA; ^5^Environmental and Occupational Health Sciences Institute, Rutgers Robert Wood Johnson Medical School, Piscataway, NJ, USA; ^6^Department of Obstetrics and Gynecology, University of British Columbia and the Children's and Women's Hospital and Health Centre of British Columbia, Vancouver, British Columbia, Canada; ^7^School of Population and Public Health, University of British Columbia, Vancouver, British Columbia, Canada

**Introduction/Purpose:** There has been a substantial increase in several chronic health conditions at all ages, including in the child‐bearing population, and such conditions contribute significantly to severe maternal morbidity and maternal mortality.

**Purpose:** To examine temporal trends in the rate of preexisting chronic conditions in pregnancy and to evaluate the extent to which changes in maternal age and obesity are associated with these trends.

**Methods:** We performed a population‐based cross‐sectional analysis of 2.8 million deliveries in Sweden and British Columbia (BC), Canada, between 1999 and 2019. Maternal chronic illnesses examined included 20 diseases. Prevalence rates and rate ratios with 95% confidence intervals (CI) expressing the frequency and change in maternal preexisting chronic conditions in relation to maternal age at delivery, period, and birth cohort were derived through age‐period‐cohort models.

**Results:** The prevalence of ≥1 chronic condition during pregnancy increased substantially in Sweden, from <3% in 1999 to almost 18% (*n* = 13 364) in 2019. In BC, Canada, in 2019, 34.1% (*n* = 14 732) of the women had a preexisting chronic condition during pregnancy, although the temporal trend was stable during the study period. The sharpest increase was observed for psychiatric conditions, and this was most pronounced among women born after 1990 in both regions. Advancing maternal age was associated with a sharp increase in preexisting circulatory system conditions during pregnancy in both populations.**Conclusions:** In Sweden, the temporal burden of preexisting chronic conditions during pregnancy has increased substantially in the last 20 years. The high prevalence of chronic conditions calls for coordinated multi‐speciality care in pregnancy.

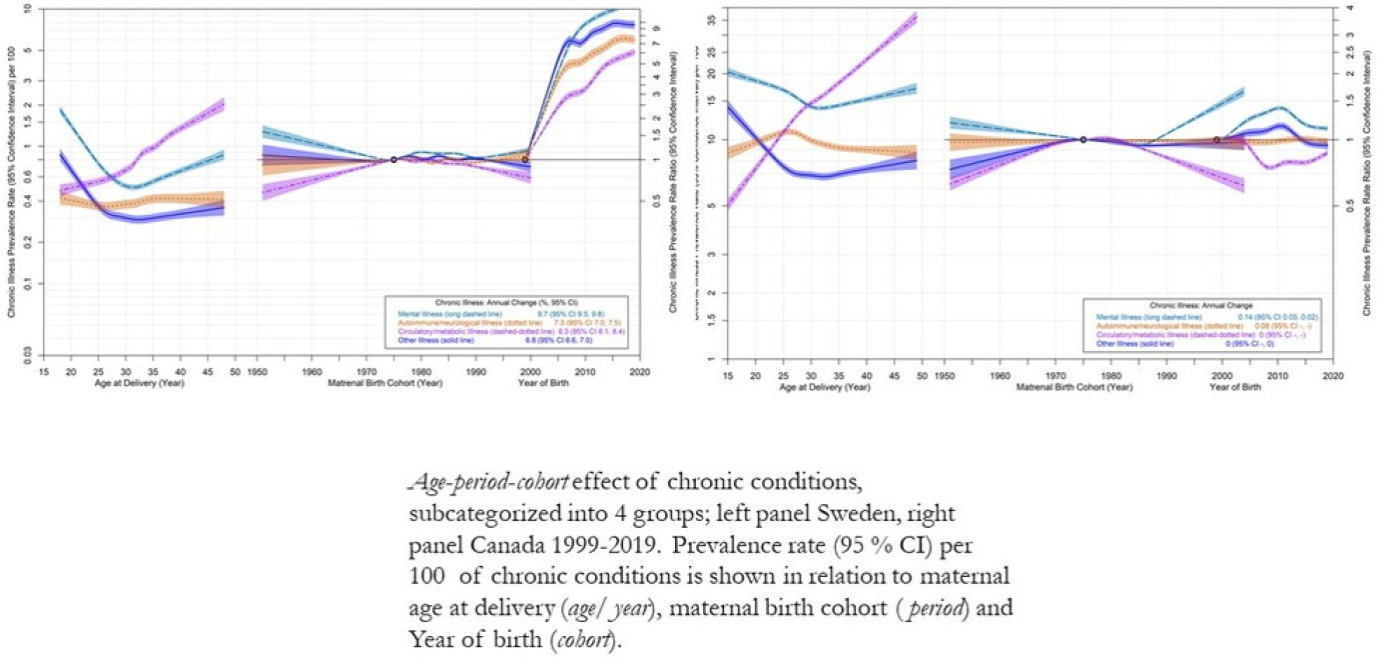

## Decoding the immune and lipid landscape of preeclampsia: a study of disease subtypes and activity

### **Anders Hagen Jarmund** ^1,2^, Guro F. Giskeødegård^3^, Mariell Ryssdal^1,2^, Signe Haaland Buer^1,2^, Marie Austdal^4^, Bjørg Steinkjer^1,2^, Line Bjørge^5^, Liv Cecilie Vestrheim Thomsen^5,6^, Eszter Vanky^1,7^, Ann‐Charlotte Iversen^1,2,7^

#### ^1^Department of Clinical and Molecular Medicine, Norwegian University of Science and Technology (NTNU), Trondheim, Norway; ^2^Center of Molecular Inflammation Research (CEMIR), NTNU, Trondheim, Norway; ^3^K.G. Jebsen Center for Genetic Epidemiology, Department of Public Health and Nursing, NTNU, Trondheim, Norway; ^4^Department of Research, Stavanger University Hospital, Stavanger, Norway; ^5^Department of Gynecology and Obstetrics, Haukeland University Hospital, Bergen, Norway; and Center for Cancer Biomarkers CCBIO, Department of Clinical Science, University of Bergen, Bergen, Norway; ^6^Medical Birth Registry of Norway, Norwegian Institute of Public Health, Bergen, Norway; ^7^Department of Obstetrics and Gynecology, St. Olav's Hospital, Trondheim University Hospital, Trondheim, Norway

**Introduction/Purpose:** Preeclampsia is a severe and heterogeneous condition with significant health impacts. The placenta plays a central role in preeclampsia and immunological disturbances and dyslipidemia substantially influence the disease progression. This study aimed to explore maternal serum cytokine and lipid profiles of preeclampsia subtypes during active disease.

**Methods:** Maternal serum collected shortly before cesarean section from normal pregnancies (*n* = 42), pregnancies with preeclampsia alone (*n* = 16) or in combination with fetal growth restriction (*n* = 28), or fetal growth restriction alone (*n* = 15) were analyzed. Clinical phenotypes were obtained from medical records and defined according to national guidelines. Placental dysfunction has previously been classified from fetal growth, sFlt‐1 and placental tissue metabolites. In all, 27 serum cytokines were mapped by multiplex assay and serum lipid measurements acquired by 600 MHz 1H‐NMR spectroscopy.

**Results:** Preeclampsia was associated with strong immune activation dominated by increased interleukin (IL)‐6 and IL‐8 and an atherogenic‐like lipid profile characterized by reduced large HDLs and increased triglycerides, small HDLs and VLDLs. Severe preeclampsia presented markedly stronger immune activation of inflammatory character than non‐severe preeclampsia. Early‐onset preeclampsia showed a broader and more potent immune activation compared with late‐onset preeclampsia, despite higher levels of IL‐6 and IL‐8 levels in the latter. Surprisingly, fetal growth restriction and placental dysfunction did not impact the maternal cytokine and lipid profiles.

**Conclusions:** The immune and lipid landscape in maternal serum provides sensitive identification of the diverging characteristics of different preeclampsia subtypes. The complex serum cytokine and lipid patterns seem to reflect disease activity rather than the underlying etiology during active preeclampsia.

## Obstetric infections and clinical characteristics of maternal sepsis. A hospital‐based historical cohort study

### **Sedina Atic Kvalvik** ^1,2^, Sofie Brænes Zakariassen^2^, Sofie Overrein^2^, Svein Rasmussen^2^, Steinar Skrede^2,3^, Elham Baghestan^1,2^

#### ^1^Department of Obstetrics and Gynecology, Haukeland University Hospital, Bergen, Norway; ^2^Department of Clinical Science, University of Bergen, Bergen, Norway; ^3^Department of Medicine, Haukeland University Hospital, Bergen, Norway

**Introduction/Purpose:** Sepsis is responsible for 50% of intrahospital maternal deaths. The incidence is increasing in both low‐/middle income and high‐income countries. There are few data on incidence and clinical outcomes of obstetric infections including maternal sepsis in the Nordic countries. The aims of this study are to give estimates of the occurrence of obstetric infections and maternal sepsis in a Norwegian hospital cohort, assess the quality of management of maternal sepsis cases, and evaluate the usefulness of diagnostic codes to identify maternal sepsis retrospectively.

**Methods:** We conducted a historical cohort study of pregnant, laboring and postpartum women at the women's clinic at Haukeland University Hospital, during 2016. Both inpatient and outpatient women diagnosed with one or several obstetric infection codes were included. We assessed the medical records and ascertained the diagnoses. For patients who met the WHO criteria for maternal sepsis, we registered patient characteristics, vital signs, antibiotic treatment and level of care. We validated the diagnostic code most frequently applied for maternal sepsis.

**Results:** We found 7.8% infection among 5182 pregnant, laboring and postpartum women. Most infections occurred during the puerperium. The incidence of maternal sepsis was 0.3%. The code O85 *Puerperal fever* was most frequently applied to encode maternal sepsis; however, its positive predictive value was only 20% for this condition.

**Conclusions:** Obstetric infections contribute significantly to maternal morbidity in Norway's second largest maternity hospital. Retrospective identification of maternal sepsis cases depends on thorough assessment of medical records, no specific code for this condition exists.

## Pregnancy management of women living with human immunodeficiency virus 2010–2019 in Stockholm, Sweden

### **Kristina Pettersson** ^1,2^, Elin Jones^1^, Karin Pettersson^1,2^, Lars Naver^3^, Annika Karlsson^4^

#### ^1^Unit of Pregnancy and Delivery, Women's Health, Karolinska University Hospital, Stockholm, Sweden; ^2^Department of Obsterics and Gynecology, Clintec, The Karolinska Institute, Stockholm, Sweden; ^3^Department of Pediatrics, Clintec, The Karolinska Institute, Stockholm, Sweden; ^4^Laboratory Medicine, Clinical Microbiology, The Karolinska Institute, Stockholm, Sweden

**Introduction/Purpose:** Antenatal and delivery care of women living with HIV is organized to optimize prevention of mother to child transmission as well as to provide care that does not stigmatize the women. Around 2010, the Swedish Reference Group for Antiretroviral Therapy altered the guidelines regarding mode of delivery and use of specific groups of antiretroviral agents. Therefore, we aimed to observe changes in obstetric management during the decade 2010–2019.

**Methods:** We performed a retrospective observational study of all women living with HIV‐1 who gave birth at Karolinska University hospital in 2010–2019. Obstetric data were collected from medical records, and data on antiretroviral therapy was obtained from the InfCare® register. Descriptive and comparative analyses based on year of delivery were carried out.

**Results:** A total of 303 deliveries were identified. Diagnosis during pregnancy occurred in 44 women (14.4%). During the study period, the use of Integrase inhibitors increased from 16.1% to 84.6%. Delivery by cesarean section decreased from 83.9% to 26.9%. Women diagnosed during pregnancy had a higher viral load at term compared with women diagnosed before pregnancy.

**Conclusions:** Viral levels were overall low but undiagnosed HIV at conception seems to increase the risk of detectable virus at term. The prevalence of pregnancy complications is similar to the background population. Vaginal delivery and the use of Integrase inhibitors significantly increased during the study period. There were no cases of mother to child transmission of HIV.

## “Stop intimate partner violence in pregnancy (STOP)” – a digital safety intervention addressing intimate partner violence during pregnancy in Denmark

### **Karen Andreasen** ^1,2^, Berit Schei^2,3,4^, Ditte S. Linde^1,2^, Vibeke Rasch^1,2^

#### ^1^Department of Gynecology and Obstetrics, Odense University Hospital, Odense, Denmark; ^2^Department of Clinical Research, University of Southern Denmark, Odense, Denmark; ^3^Institute of Public Health, Norwegian University of Science and Technology, Trondheim, Norway; ^4^Department of Obstetrics and Gynecology, St. Olav's University Hospital, Trondheim, Norway

**Introduction/Purpose:** Intimate partner violence (IPV) during pregnancy is a serious public health issue with wide‐ranging consequences for both the mother and the unborn child. During antenatal care it is possible to screen pregnant women for IPV; this offers a unique opportunity for early intervention. Therefore, all hospitals in the Region of Southern Denmark launched the STOP project.

This study explores (1) the feasibility of digital IPV screening and a digital supportive intervention targeting pregnant women exposed to IPV and (2) the effect of the intervention on IPV exposure and maternal mental health.

**Methods:** As part of the STOP project, pregnant women were screened digitally for IPV exposure during routine antenatal care using validated tools; women who screened positive were offered 3–6 video consultations with specially trained midwives and access to a safety planning app. Questionnaire data were collected before and after the intervention on exposure to violence and depression score.

**Results:** From January 2021 to July 2022, 14 013 women (81.4%) completed the screening; 1195 screened positive for IPV exposure (*n* = 8.5%), with 25.5% (*n* = 305) women eligible to receive the intervention. A total of 54 women accepted the intervention (18%) and 40 completed it (13.1%).

Preliminary findings found a significant reduction in IPV exposure (ISA score −4.8, 95% CI −9.6 to −0.8) and depression score (EPDS −3.8, 95% CI −5.5 to –2.1) after completing the intervention with an average of five consultations.

**Conclusions:** Digital screening for IPV among pregnant women is feasible in an antenatal care context. Among the women who accepted the intervention, we found a positive effect on maternal health.

## National screening of substance use in pregnancy among Danish women

### **Nete Lundager Klokker Rausgaard** ^1,2^, Inge Olga Ibsen^2^, Palle Bach Nielsen Fruekilde^3^, Ellen Aagaard Nohr^1,2^, Per Damkier^1,4^, Pernille Ravn^1,2^

#### ^1^Department of Clinical Research, University of Southern Denmark, Odense, Denmark; ^2^Department of Gynecology and Obstetrics, Odense University Hospital, Odense, Denmark; ^3^Department of Clinical Biochemistry, Odense University Hospital, Odense, Denmark; ^4^Department of Clinical Pharmacology, Odense University Hospital, Odense, Denmark

**Introduction/Purpose:** There is a paucity of information regarding substance use among Danish pregnant women. We estimated the prevalence of substance use including alcohol and tobacco in the entire Danish pregnant population.

**Methods:** In this anonymous, national, cross‐sectional, descriptive study, pregnant women were invited when attending the ultrasound scan offered as part of a routine midwife visit between November 2019 and December 2020 at nine Danish hospitals. Study subjects submitted a urine sample and filled out a questionnaire. Urine samples were tested on site with a urine dipstick for 15 substances including alcohol, tobacco, opioids, amphetamines, cannabis and benzodiazepines. All screen‐positive urine samples underwent secondary analyses with gold standard, LC/MS‐analysis. No ethics or data collection permission was required.

**Results:** 1904 of 2155 pregnant women participated (88%). Mean age was 30.4 years. Mean gestational age was 23.3 weeks. Overall prevalence of screen‐positive urine samples was 25% (13% alcohol, 41% tobacco, 6% opioids, 19% amphetamines, 20% other substances). 54% of screen‐positive urine samples were confirmed positive. Further analyses are ongoing. Results will include region‐ and substance‐specific prevalences, and results will be compared with questionnaire information to analyze validity of self‐report and examine possible cross‐reactions. Non‐participant analyses based on information on age and reason for refusal will be carried out. Additionally, we will analyze the validity of using urine dipsticks on site.

**Conclusions:** This abstract presents preliminary data which are close to final. Final data will be presented at the Congress. The study is essential for future, national strategies within identification of and help to pregnant women with use of potential teratogenic substances.

## Maternal human papillomavirus infection during pregnancy and preterm delivery, a mother–child cohort study in Norway and Sweden

### **Johanna Wiik** ^1,2,3^, Magdalena R. Værnesbranden^1,4^, Christine M. Jonassen^5,6^, Anne Cathrine Staff^4,7^, Karin C. L. Carlsen^4,8^, Berit Granum^9^, Guttorm Haugen^4,7^, Gunilla Hedlin^10,11^, Katarina Hilde^4,7^, Bo Jacobsson^1,3,12^, Staffan Nilsson^13,14^, Björn Nordlund^10,11^, Anbjørg Rangberg^6^, Eva Maria Rehbinder^4,15^, Verena Sengpiel^1,3^, Håvard Skjerven^4,8^, Birgitte K Sundet^4,7^, Cilla Söderhäll^10,11^, Riyas Vettukattil^4,8^, Katrine Sjøborg^2^

#### ^1^Department of Obstetrics and Gynecology, Institute of Clinical Sciences, Sahlgrenska Academy, University of Gothenburg, Gothenburg, Sweden; ^2^Department of Gynecology and Obstetrics, Østfold Hospital Trust, Kalnes, Norway; ^3^Region Västra Götaland, Sahlgrenska University Hospital, Department of Obstetrics and Gynecology, Gothenburg, Sweden; ^4^Institute of Clinical Medicine, Faculty of Medicine, University of Oslo, Oslo, Norway; ^5^Department of Virology, Norwegian Institute of Public Health Oslo, Norway; ^6^Genetic Unit, Center for Laboratory Medicine, Østfold Hospital Trust, Kalnes, Norway. ^7^Division of Obstetrics and Gynecology, Oslo University Hospital, Oslo, Norway; ^8^Division of Pediatric and Adolescent Medicine, Oslo University Hospital, Oslo, Norway; ^9^Department of Chemical Toxicology, Norwegian Institute of Public Health, Oslo, Norway; ^10^Astrid Lindgren Children's Hospital, Karolinska University Hospital, Stockholm, Sweden; ^11^Department of Women's and Children's Health, Karolinska Institutet, Stockholm, Sweden; ^12^Department of Genetics and Bioinformatics, Division of Health Data and Digitalization, Institute of Public Health, Oslo, Norway; ^13^Department of Laboratory Medicine, Institute of Biomedicine, Sahlgrenska Academy, University of Gothenburg, Gothenburg, Sweden; ^14^Department of Mathematical Sciences, Chalmers University of Technology, Gothenburg, Sweden; ^15^Department of Dermatology and Venereology, Oslo University Hospital, Oslo, Norway

**Introduction/Purpose:** Human papillomavirus (HPV) infection is common in women of reproductive age. Infection and inflammation are leading causes for preterm delivery (PTD), but the role of HPV infection in PTD and prelabor rupture of membranes (PROM) is unclear. We aimed to explore whether HPV infection during pregnancy in general, and high‐risk HPV infection specifically, increased the risk of PTD, preterm prelabor rupture of membranes (pPROM) and PROM at term.

**Methods:** In pregnant women, participating in a prospective multicenter cohort study from a general population in Norway and Sweden (PreventADALL, ClinicalTrials.gov NCT02449850), HPV‐DNA was analyzed in available urine‐samples at mid‐gestation and at delivery. The risk of PTD, pPROM and PROM was analyzed using logistic regression analyses for any‐HPV (28 genotypes), subgroups of HPV (low‐risk/possibly high‐risk‐HPV, high‐risk‐HPV‐non‐16 and high‐risk‐HPV‐16) and presence of multiple HPV infections, compared with HPV‐negative women. Samples for HPV analyses were available from 950 women with singleton pregnancies (mean age 32 years) at mid‐gestation and in 753 at delivery as well.

**Results:** At mid‐gestation, 40% of women were positive for any‐HPV and 24% for any of 12 high‐risk‐HPV‐genotypes. High‐risk‐HPV‐positive women more often had PTD (8/231, 3.5%) compared with HPV‐negative women (13/573, 2.3%) at mid‐gestation, but the association was not statistically significant (odds ratio [OR] 1.55, 95% confidence interval [CI] 0.63–3.78; *P* = 0.34). Neither presence of any‐HPV, subgroups of HPV nor multiple HPV infections were significantly associated with increased risk for PTD, pPROM or PROM.

**Conclusions:** HPV infection during pregnancy was not significantly associated with increased risk for PTD, pPROM or PROM among women from a general population with a low incidence of adverse obstetric outcomes.

## Increased proportion of spontaneous vaginal deliveries in Norway

### **Katariina Laine** ^1,2^, Branka M. Yli^3^

#### ^1^Norwegian Research Center for Women's Health, Oslo University Hospital, Oslo, Norway; ^2^Institute of Clinical Medicine, Faculty of Medicine, University of Oslo, Oslo, Norway; ^3^Department of Obstetrics, Oslo University Hospital, Oslo, Norway

**Introduction/Purpose:** Operative delivery is a life‐saving procedure for the mother and the child when medically indicated. Overuse of operative deliveries with no or weak indications increases maternal and fetal morbidity. The aim of this study was to explore the time trends of operative deliveries and spontaneous vaginal deliveries in Norway, and the associations with perinatal and neonatal outcomes.

**Methods:** The study population consisted of 1 329 795 births registered in the Medical Birth Registry Norway in 1999–2021. The proportion of annual spontaneous deliveries was calculated from the total number of births by extracting operative deliveries (cesarean section, vacuum extraction and forceps delivery). Statistical significance was assessed with confidence intervals.

**Results:** Cesarean delivery rate was reduced from 16.7% in 2008 to 15.0% in 2021, and operative vaginal deliveries from 10.6% in 2016 to 9.9%, resulting in an increasing proportion of spontaneous vaginal deliveries, from 72.6% in 2014 to 75.1% in 2021.

Intrapartum mortality was reduced from 0.5‰ to 0.14‰. Early neonatal mortality was reduced from 7.1‰ to 3.5‰. Proportion of newborns with Apgar scores <7 at 5 minutes of age remained unchanged.

Changes in study population: The proportion of women aged 35 years increased from 13.6% in 1999 to 22.3% in 2021. Labor induction increased from 10.5% to 28.4%. Use of epidural pain relief increased from 23.3% in 1999 to 43.0% in 2021.

**Conclusions:** The use of operative deliveries has been reduced and the proportion of spontaneous vaginal birth has increased since 2014 in Norway, without compromising the fetal outcome.

## Adherence to different forms of plant‐based diet and pregnancy outcomes in the Danish national birth cohort

### **Signe Hedegaard** ^1^, Ellen Aagaard Nohr^2^, Sjurdur Frodi Olsen^3^, Thorhallur Ingvi Halldorsson^3,4^, Kristina Martha Renault^5,6^

#### ^1^Department of Obstetrics and Gynecology, Herlev University Hospital, University of Copenhagen, Herlev, Denmark; ^2^Research Unit for Obstetrics and Gynecology, Department of Clinical Research, University of Southern Denmark, Odense, Denmark; ^3^Department of Epidemiologic Research, Statens Serum Institut, Copenhagen, Denmark; ^4^Faculty of Food Science and Nutrition, University of Iceland, Reykjavik, Iceland; ^5^Department of Obstetrics, Rigshospitalet, Juliane Marie Center, University of Copenhagen, Copenhagen, Denmark; ^6^University of Copenhagen, Faculty of Health and Medical Sciences, Department of Clinical Medicine, Copenhagen, Denmark

**Introduction/Purpose:** The prevalence of those adhering to plant‐based diets has increased dramatically in recent years, fueled by both environmental and animal welfare concerns. Beneficial or possible adverse consequences of such diets, particularly the most restrictive forms during pregnancy, have been minimally explored.

Aim: To examine associations between different forms of plant‐based diets during pregnancy with birth outcomes and pregnancy complications.

**Methods:** We included 66 738 pregnancies from the Danish National Birth Cohort, recruited in 1996–2002. Dietary and supplemental intake was assessed in gestational week 25 and women were characterized as fish/poultry‐vegetarians, lacto/ovo‐vegetarians, vegans or omnivorous based on their self‐report.

**Results:** A total of 98.7% (*n* = 65 872) of study participants were defined as omnivorous, while 1.0% (*n* = 666), 0.3% (*n* = 183) and 0.03% (*n* = 18) identified themselves as fish/poultry vegetarians, lacto/ovo vegetarians or vegans, respectively. Protein intake as percentage of energy was substantially lower among lacto/ovo vegetarians (13.3%) and vegans (10.4%) compared with omnivores (15.4%). Intake of micronutrients such as vitamin B12, calcium and vitamin D was considerably lower among vegans, but when dietary supplements were taken into consideration, no major differences were observed. Offspring of vegan compared with omnivorous mothers had on average −240 g (95% CI −450 to −30) lower birth‐weight and higher prevalence of low birthweight and preeclampsia. No such differences were observed for fish/poultry or lacto/ovo vegetarians.

**Conclusions:** The few women reported to adhere to vegan diets during pregnancy had lower mean birthweight and higher risk of preeclampsia compared with omnivorous mothers. Low protein intake may be one plausible explanation for the observed association with birthweight.

## Adherence to recommended physical activity‐restrictions due to threatened preterm delivery – a descriptive multi‐center study

### **Jane M. Bendix** ^1,2^, Mette G. Backhausen^3^, Hanne K. Hegaard^4,5,6^, Ane L. Rom^4,6,7^, Stig Molsted^2,5^, Ellen C. L. Lokkegaard^1,5^

#### ^1^Department of Gynecology and Obstetrics, Copenhagen University Hospital – North Zealand, Hillerød, Denmark; ^2^Department of Clinical Research, Copenhagen University Hospital – North Zealand, Hillerød, Denmark; ^3^Department of Gynecology and Obstetrics, Zealand University Hospital, Roskilde, Denmark; ^4^Department of Obstetrics, Copenhagen University Hospital – Rigshospitalet, Copenhagen, Denmark; ^5^Department of Clinical Medicine, University of Copenhagen, Copenhagen, Denmark; ^6^The Interdisciplinary Research Unit of Women's, Children's and Families’ Health, The Juliane Marie Center for Women, Children and Reproduction Copenhagen University Hospital – Rigshospitalet, Copenhagen, Denmark; ^7^Research Unit of Gynecology and Obstetrics, Department of Clinical Research, University of Southern Denmark, Odense, Denmark

**Introduction/Purpose:** Threatened preterm delivery is a serious complication and physical activity restrictions are often prescribed. Adherence to recommended activity restriction is unknown. This study aimed to assess the objectively measured physical positions and activities of pregnant women recommended activity restrictions due to threatened preterm delivery, compared with a reference group of uncomplicated pregnant women without restrictions, and to explore whether admission status influenced adherence to activity restrictions.

**Methods:** A Danish descriptive, clinical multi‐center study included singleton pregnancies between 22 and 33 gestational weeks who were either prescribed activity restrictions due to threatened preterm delivery or uncomplicated controls without restrictions. For 7 days, participants wore tri‐axial accelerometric SENS® monitors. Accelerometric data included time spent in five different positions, activities and step counts.

**Results:** Seventy‐two pregnant women participated; 31% were prescribed strict activity restrictions, 15% moderate, 3% light, 8% unspecified and 43% had no activity restrictions. Strict activity‐restriction participants rested in the supine/lateral position for 17.7 median hours/day; sat upright 4.9 hours/day; took 1520 steps/day, and 64% were inpatients. Moderate activity‐restriction participants rested in the supine/lateral position for 15.1 hours/day; sat upright 5.6 hours/day; took 3310 steps/day, and 64% were outpatients. Participants with no activity restrictions rested 10.5 hours/day in supine/lateral position; sat upright 7.6 hours/day and took 9235 steps/day. Among strict activity restriction participants, admission status did not alter time spent in the physical positions nor the step count.

**Conclusions:** Overall, participants adhered closely to the recommended activity restriction. However, discriminating between strict and moderate activity restriction recommendations did not alter how physical resting positions and activities were carried out. The admission status did not influence how participants adhered to strict activity restriction.

## AI‐assisted telephone triage in emergency maternity care

### **Maria Jeppegaard** ^1^, Lone Krebs^1,2^, Lene Drasbek Huusom^3^, Lars Maaløe^4^, Helene Westring Hvidman^5^

#### ^1^Department of Gynecology and Obstetrics, Amager Hvidovre Hospital, Hvidovre, Denmark; ^2^Department of Clinical Medicine, University of Copenhagen, Copenhagen, Denmark; ^3^Department of Gynecology and Obstetrics, Herlev‐Gentofte Hospital, Herlev, Denmark; ^4^Corti Aps, Copenhagen, Denmark; ^5^Department of Gynecology and Obstetrics, Roskilde Hospital, Roskilde, Denmark

**Introduction/Purpose:** Every day a large number of women call the birth units and report different problems in terms of, among other problems, vaginal bleeding, pain or decreased fetal movements. These conversations constitute a big challenge and responsibility for the midwives taking the calls. It is known that phone triage is affected by high workload, the interpersonal interaction influenced by the healthcare professional's experience and the woman's cultural and socioeconomic background. Correct phone triage is a difficult task, but crucial for treatment quality, patient safety and efficient use of resources. A machine‐learning framework might be trained to recognize severe obstetric outcomes from the recorded calls and thereby contribute to optimizing visitation and prevent severe obstetric complications.

The aim with this study is to optimize the triage in the emergency maternal care and ensure the same high quality around the clock. This will be achieved by developing algorithms based on machine learning. The algorithms will interpret the telephone conversations in real‐time and support the midwives during the phone call so the right treatment is offered.

**Methods:** All phone calls to the emergency maternity unit at Hvidovre and Herlev Hospital, Denmark, have been recorded since 2022.

**Results:** Currently, we map all the phone calls to determine how often, why and when the pregnant women call and how they are triaged. Subsequently, we will develop and train algorithms based on machine learning to assist the midwife receiving the calls.

**Conclusions:** We will present the project and the first results at the NFOG Congress in August 2023.

## Alcohol exposure prior to pregnancy – does hazardous consumption affect placenta‐ and inflammatory‐mediated pregnancy outcomes?

### **Joline Asp** ^1,2^, Lina Bergman^2,3,4^, Susanne Lager^2^, Ove Axelsson^1,2^, Anna‐Karin Wikström^2^, Susanne Hesselman^2,5^

#### ^1^Center for Clinical Research Sörmland, Uppsala University, Eskilstuna, Sweden; ^2^Department of Women's and Children's Health, Uppsala University, Uppsala, Sweden; ^3^Department of Obstetrics and Gynecology, Institute of Clinical Science, Sahlgrenska Academy, University of Gothenburg, Gothenburg, Sweden; ^4^Department of Obstetrics and Gynecology, Stellenbosch University, Stellenbosch, South Africa; ^5^Center for Clinical Research Dalarna, Falun, Sweden

**Introduction/Purpose:** Alcohol consumption during pregnancy is related to severe birth complications such as low birthweight, preterm birth and birth defects. During the last decade, the Alcohol Use Disorders Identification Test (AUDIT) has been used as a screening tool in Swedish maternal healthcare units to identify hazardous, pre‐pregnancy alcohol use. However, evaluation of the screening with AUDIT, as well as adverse maternal or neonatal outcomes, has not been assessed at a national level.

**Methods:** This was a population‐based cohort study of 530 458 births from 2013 to 2018 using health data from the Swedish Pregnancy Register. Self‐reported alcohol consumption in the year before pregnancy, measured as AUDIT scores, was categorized into moderate and high‐risk consumption, with low‐risk consumption as the reference group. Associations with pregnancy‐ and birth outcomes were explored with logistic regressions.

**Results:** High‐risk and moderate pre‐pregnancy alcohol consumption was associated with preeclampsia, preterm birth and birth of a small‐for‐gestational‐age (SGA) infant, but these associations were nonsignificant after adjustments. Prior moderate‐risk (adjusted odds ratio [aOR] 1.29, 95% confidence interval [CI] 1.17–1.42) and high‐risk consumption (aOR 1.62, 95% CI 1.17–2.25) increased the likelihood of intrapartum and neonatal infections.**Conclusions:** Apart from identifying hazardous alcohol consumption prior to pregnancy and the offer of counseling, screening with the AUDIT in early pregnancy identifies a high risk of inflammatory/placenta‐mediated pregnancy and birth outcomes. For most outcomes, AUDIT was not an independent contributor when adjusting for confounding factors. Hazardous alcohol use prior to pregnancy was independently linked to intrapartum and neonatal infections, conditions associated with morbidity and long‐term sequalae.

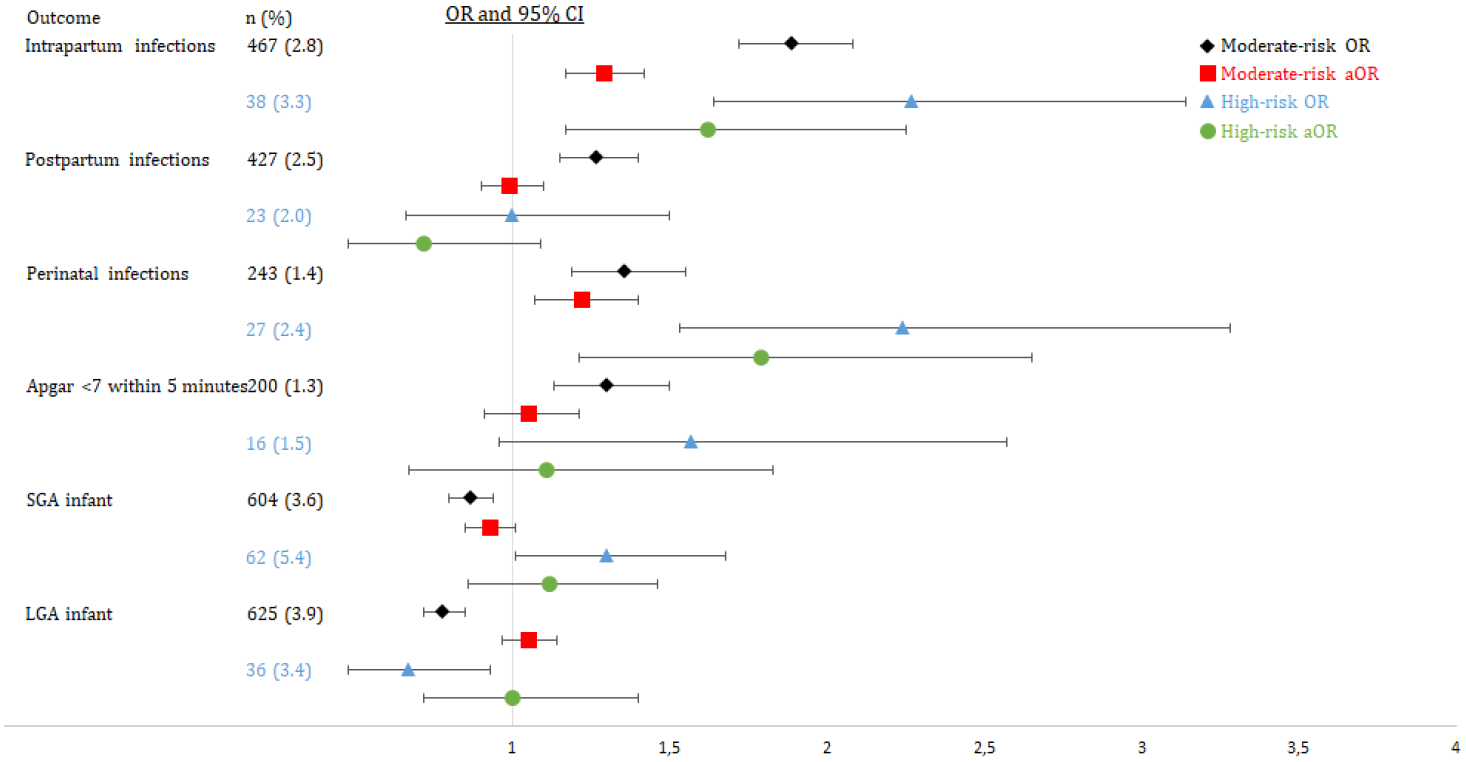

## Alterations in signaling pathways in placentas – does the dose of vitamin D supplementation in pregnancy matter?

### **Anna Louise Vestergaard** ^1,2^, Matilde Kanstrup Andersen^1,3^, Pinar Bor^1,2^, Agnete Larsen^3^

#### ^1^Department of Gynecology and Obstetrics, Randers Regional Hospital, Randers, Denmark; ^2^Department of Clinical Medicine, Aarhus University, Aarhus, Denmark; ^3^Department of Biomedicine, Aarhus University, Aarhus, Denmark

**Introduction/Purpose:** Vitamin D deficiency in pregnancy remains an obstetric challenge due to its high prevalence and because it heightens the risk of placenta‐related complications. However, very little is known about alterations occurring in placentas at a broader genetic level and which signaling pathways are affected when placentas are exposed to different vitamin D levels. Such knowledge would help us to understand better why vitamin D is so important in pregnancy and whether extra vitamin D could improve placenta function and prevent placenta‐related complications. Hence, we aim to identify vitamin D‐associated signaling pathways in the placenta and their response to increased supplementation.

**Methods:** Placentas were collected as part of a randomized clinical trial (GRAVITD) investigating the effect of high (90 μg) vs low (10 μg) vitamin D doses. Seventy‐two placenta samples with equal distribution across vitamin D dose regimens were randomly selected for next generation RNA sequencing.

**Results:** As one of the first of its kind, this study will show which gene expression and signaling pathways are altered in placentas according to the dose of vitamin D used in pregnancy. Given the size of the study and the variation of the pregnancies from which tissue were included, we will discuss whether vitamin D affects fluctuations in placental function associated with growth patterns and maternal risk factors such as overweight.

**Conclusions:** The study will help determine whether increased vitamin D supplementation is needed to optimize placental function.

## Birthweight and gestational age in the Faroe Islands

### **Sunnvá Hanusardóttir Olsen** ^1^, Diana Reynstind^1^, Hlynur Hallgrímsson^2^, Ulrik Schiøler Kesmodel^3^

#### ^1^Department of Obstetrics and Gynecology, National Hospital of the Faroe Islands, Tórshavn, Faroe Islands; ^2^The City of Reykjavík's Office of Data Services, Reykjavík, Iceland; ^3^Department of Obstetrics and Gynecology, Aalborg University Hospital, Aalborg, Denmark

**Introduction/Purpose:** This study aimed to examine Faroese infants’ birthweight (BW) and gestational age (GA) in 2010–2019 and compare these findings with other Nordic countries. Risk factors for high BW among Faroese infants were also investigated in this study.

**Methods:** All singleton live‐born infants registered in the Faroese Birth Registry in 2010–2019 were included (*n* = 6121). A comparison was made with data from Denmark, Iceland, Norway and Sweden.

**Results:** The mean BW increased significantly from 3652 g (95% confidence interval [CI] 3505–3699 g) in 2010 to 3745 g (95% CI 3700–3790 g) in 2019, a mean increase in BW of 93 g (95% CI 28–158 g) (*P* < 0.05). The proportion of infants weighing ≥4500 g increased significantly from 6.1% in 2010 to 9.6% in 2019 (*P* < 0.05). The risk of giving birth to an infant weighing ≥4000 g was consistently associated with previously giving birth (odds ratio [OR] 1.98, 95% CI 1.71–2.30

and GA (OR 1.28, 95% CI 1.23–1.33, per week increase in GA). Compared with other Nordic countries, Faroese infants’ mean BW was high, the Faroe Islands had a higher number of infants born with a weight of ≥4000 g and a higher proportion of infants born in gestational week 41 or later.**Conclusions:** The mean BW and the proportion of infants with high BW significantly increased during 2010–2019 in the Faroe Islands. The mean BW, the proportion of infants with high BW and the GA for Faroese infants was higher than all other Nordic countries. The reasons for this require further investigation.

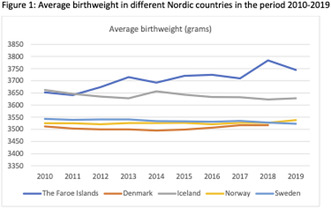

## Breastfeeding and insulin requirements in women with type 1 diabetes mellitus in the first year postpartum

### **Gitte Øskov Skajaa** ^1,2^, Ulla Kampmann^1,3^, Per Glud Ovesen^1,2,3^, Jens Fuglsang^1,2,3^

#### ^1^Department of Clinical Medicine, Aarhus University, Aarhus, Denmark; ^2^Department of Obstetrics and Gynecology, Aarhus University Hospital, Aarhus, Denmark; ^3^Steno Diabetes Center Aarhus, Aarhus, Aarhus University Hospital, Aarhus, Denmark

**Introduction/Purpose:** This study aims to explore whether breastfeeding affects postpartum insulin requirements, HbA1c levels, and pregnancy weight retention (PWR) in women with Type 1 diabetes mellitus (T1DM) in the first year after delivery.

**Methods:** This prospective study included 66 women with T1DM. The women were divided into two groups based on whether they were breastfeeding (BF) at 6 months postpartum (BF_yes_, *n* = 32) or not (BF_no_, *n* = 34). Mean daily insulin requirement (MDIR), HbA1c levels and PWR at five time‐points from discharge to 12 months postpartum were compared.

**Results:** MDIR increased by 35% from 35.7 IU at discharge to 48.1 IU at 12 months postpartum (*P* < 0.001). MDIR in BF_yes_ and BF_no_ were comparable, however, in BF_yes_, MDIR was continuously lower compared with BF_no_. Postpartum HbA1c increased rapidly from 6.8% at 1 month to 7.4% at 3 months postpartum and settled at 7.5% at 12 months postpartum. The increase in HbA1c during the first 3 months postpartum was most pronounced in BF_no_ (*P* < 0.001). Although neither were statistically significant, from 3 months postpartum, HbA1c levels were highest in the BF_no_ and BF_no_ had a higher PWR compared with BF_yes_ (*P* = 0.31).

**Conclusions:** We observed that in women with T1DM, insulin requirements and HbA1c levels during the first year postpartum were similar when comparing women who breastfed at 6 months postpartum to women who did not. In addition, HbA1c deteriorated postpartum, resulting in HbA1c being significantly higher at 12 months postpartum compared with the pre‐pregnancy level. Overall, PWR was low and not significantly affected by breastfeeding.

## Cesarean section in women with juvenile idiopathic arthritis – a population‐based study

### **Carina Skorpen** ^1,2^, Stian Lydersen^3^, Kjell Salvesen^4,5^, Marianne Wallenius^2,6^

#### ^1^Department of Rheumatology Ålesund, Helse More og Romsdal HF, Ålesund, Norway; ^2^Institute of Neuromedicine and Movement Science, The Norwegian University of Science and Technology, Trondheim, Norway; ^3^Department of Mental Health, Regional Center for Child and Youth Mental Health and Child Welfare, The Norwegian University of Science and Technology, Trondheim, Norway; ^4^Department of Clinical and Molecular Medicine, The Norwegian University of Science and Technology, Trondheim, Norway; ^5^Department of Obstetrics and Gynecology, St. Olav's hospital, Trondheim University Hospital, Trondheim, Norway; ^6^The Norwegian National Advisory Unit on Pregnancy and Rheumatic Diseases, Department of Rheumatology, St. Olav's Hospital, Trondheim University Hospital, Trondheim, Norway

**Introduction/Purpose:** The literature on delivery methods in women with juvenile idiopathic arthritis (JIA) is insufficient. Active inflammation is a risk factor for cesarean section (CS) in other arthritic diseases. A CS entails a higher risk for complications than vaginal delivery and a restricted physical activity the first weeks after birth.

Our objective was to explore a possible association of inflammatory active disease and the proportion of CS in women with JIA.

**Methods:** Data from the Norwegian nationwide observational register RevNatus were linked with data from the Medical Birth Registry of Norway (MBRN). Cases comprised singleton births in women with JIA (*n* = 196) included in RevNatus from 2010 to 2019. Singleton births registered in MBRN during the same period of time and excluding mothers with rheumatic inflammatory diseases (*n* = 575 798) served as population controls. Disease activity was assessed using Disease Activity Score with CRP (DAS28‐CRP‐3). We defined inactive JIA as DAS28‐CRP‐3 <2.6 and active JIA as DAS28‐CRP‐3 ≥2.6.

**Results:** CS was more frequent in women with JIA (20.4%) than in population controls (15.6%) and occurred most frequently in inflammatory active JIA (30.0%). Women with JIA had similar risk for elective CS (risk difference 1.1%, 95% confidence interval [CI] −1.7 to 5.4) and higher risk for emergency CS (risk difference 3.8%, 95% CI −0.4 to 9.3) compared with population controls. Active disease increased the risk for emergency CS (risk difference 14.0%, 95% CI 4.3–27.4).

**Conclusions:** Women with active JIA had higher risk for emergency CS compared with population controls.

## Childbirth experience in late‐ and post‐term nulliparous women with labor induction

### **Katariina Place** ^1^, Heidi Kruit^1^, Leena Rahkonen^1^

#### ^1^Department of Obstetrics and Gynecology, Helsinki University Hospital and University of Helsinki, Helsinki, Finland

**Introduction/Purpose:** Childbirth experience affects the mother's health and well‐being profoundly. Labor induction and nulliparity are known risk factors for a negative experience, and passing the due date is often distressing. Hence, information on factors affecting the childbirth experience in this vulnerable group of women is of great importance.

**Methods:** Nulliparous women with labor induction at ≥41+0 gestational weeks in six Finnish hospitals participating in two prospective studies on labor induction from 2018 to 2020 answered the Childbirth Experience Questionnaire (CEQ). The scale is 1–4, where 1 represents the most negative and 4 the most positive experience possible.

**Results:** In this sub‐analysis of 260 women, the factors affecting the total mean CEQ score negatively were: BMI ≥30, misoprostol, two consecutive cervical ripening methods, oxytocin, fetal scalp sampling, maternal intrapartum infection, induction to delivery interval ≥24 hours, duration of labor ≥12 hours, hemorrhage ≥1000 mL, vacuum extraction, cesarean section, adverse maternal or neonatal outcome, and neonate in the intensive care unit. When comparing women with labor induction at 41+0 to 41+4 (~late‐term) vs 41+5 to 42+ (~post‐term), no differences in the CEQ were detectable.

**Conclusions:** Lean women experiencing a swift and efficient labor induction process, non‐prolonged labor and a non‐instrumental vaginal delivery are more likely to experience their labor positively. Not using misoprostol, oxytocin or more than one cervical ripening method was also seen as favorable. Avoiding maternal and neonatal adverse outcomes could, in addition to physical health benefits, promote a better childbirth experience. The timing of labor induction at ≥41+0 weeks did not influence the experience.

## Childbirth experiences in women with polycystic ovary syndrome

### **Anne Engtrø Husby** ^1,2^, Melanie Rae Simpson^3^, Rebecka Dalbye^4,5^, Eszter Vanky^1,2^, Tone Shetelig Løvvik^1,2^

#### ^1^Department of Obstetrics and Gynecology, St. Olav's University Hospital, Trondheim, Norway; ^2^Department of Clinical and Molecular Medicine, Faculty of Medicine and Health Sciences, Norwegian University of Science and Technology, Trondheim, Norway; ^3^Department of Public Health and Nursing, Faculty of Medicine and Health Sciences, Norwegian University of Science and Technology, Trondheim, Norway; ^4^Department of Obstetrics and Gynecology, Østfold Hospital Trust, Grålum, Norway; ^5^Department of Nursing and Health Promotion, Faculty of Health Sciences, Oslo Metropolitan University, Oslo, Norway

**Introduction/Purpose:** Women with polycystic ovary syndrome (PCOS) have more pregnancy complications, and metformin has been used in an attempt to improve pregnancy outcomes. Childbirth experiences are influenced by previous life and birth experiences as well as mode and outcome of delivery. Our study aims to explore childbirth experiences in women with PCOS compared with a reference population, and to explore the effect of metformin vs placebo on the childbirth experiences in women with PCOS.

**Methods:** This study combines data from two RCTs. In the PregMet2 study, women with PCOS were randomized to metformin or placebo. Main outcomes were miscarriage and preterm delivery. The Labor Progression Study (LaPS) (reference population) compared the WHO partograph to the Zhang's guidelines for progression of labor. Both studies used the Childbirth Experience Questionnaire (CEQ). Total CEQ scores and four domain scores were compared between women with PCOS in the PregMet2 study (*n* = 131) and the LaPS study (*n* = 3604), and metformin (*n* = 179) and placebo (*n* = 185) in the PregMet2 study.

**Results:** There was no difference in total CEQ score between women with PCOS and the reference population (LaPS). Women with PCOS had a higher domain score for “perceived safety” (*P* < 0.05) and lower score for “own capacity” (*P* < 0.05) compared with LaPS. Total CEQ‐score and domain‐scores were similar for metformin and placebo in PregMet2.

**Conclusions:** Women with PCOS had similar childbirth experiences compared with reference women. However, they reported higher perceived safety and lower own capacity. Metformin treatment in pregnancy did not influence childbirth experience in women with PCOS.

## Community‐based doula support during labor and birth to improve migrant women's intrapartum care experiences and emotional well‐being – an RCT

### **Erica Schytt** ^1,2,3^, Amani Eltayb^1^, Anna Wahlberg^1^, Nataliia Tsekhmestruk^1^, Rhonda Small^4^, Helena Lindgren^1^

#### ^1^Karolinska Institutet, Division of Reproductive Health, Department of Women's and Children's Health, Stockholm, Sweden; ^2^Center for Clinical Research Dalarna – Uppsala University, Falun, Sweden; ^3^Western Norway University of Applied Sciences Norway, Faculty of Health and Social Sciences, Bergen, Norway; ^4^La Trobe University, Judith Lumley Centre, Melbourne, Victoria 3086, Australia

**Introduction/Purpose:** The aim was to evaluate the effectiveness of community‐based bilingual doula (CBD) support for improving the intrapartum care experiences and postnatal wellbeing of migrant women giving birth in Sweden.

**Methods:** Randomized controlled trial. 164 pregnant Somali‐, Arabic‐, Polish‐, Russian‐ and Tigrinya‐speaking women who could not communicate fluently in Swedish, were ≥18 years and had no contraindications for vaginal birth were recruited from six antenatal care clinics in Stockholm, Sweden. In addition to standard labor support, women were randomized to CBD support (*n* = 88) or no such support during labor (*n* = 76). Trained CBDs met with women prior to labor, provided support by telephone after labor had started, then provided emotional, physical and communication support throughout labor and birth, and met again with women postpartum. Primary outcomes: women's overall ratings of intrapartum care experiences (Migrant Friendly Maternity Care Questionnaire) and postnatal wellbeing (Edinburgh Postnatal Depression Scale) at 6–8 weeks after birth.

**Results:** Of women allocated CBD support, 60 (73.2%) received support during labor. There were no differences between the groups regarding women's intrapartum care experiences (very happy with care: CBD 80.2% (*n* = 65) vs SC 79.1% (*n* = 53); odds ratio [OR] 1.07, 95% confidence interval [CI] 0.48–2.40) or emotional wellbeing (EPDS mean value: CBD 4.71 (SD 4.96) vs SC 3.38 (SD 3.58); mean difference 1.33; 95% CI 0.10–2.75).

**Conclusions:** Community‐based doula support during labor and birth for migrant women neither increased women's ratings of their care for labor and birth nor their emotional well‐being 2 months postpartum compared with receiving standard care only.

## Birth outcomes in Nordic countries

### **Charlotte Elvander** ^1^, Eva Rydahl^2^, Emma Swift^3^, Ellen Blix^4^

#### ^1^Karolinska Institutet, Clinical Epidemiology Division, Stockholm, Sweden; ^2^University College of Copenhagen, Department of Midwifery, Copenhagen, Denmark; ^3^University of Iceland, Faculty of Nursing and Midwifery, Reykjavik, Iceland; ^4^ OsloMet – Storbyuniversitetet, Fakultet for Helsevitenskap, Oslo, Norway

**Introduction/Purpose:** Composite outcome measures in maternity care can be useful when measuring and comparing overall quality of care in different countries, regions and birth units over time. By combining a set of single outcomes that reflect the health of the mother, the health of the baby and interventions used, the combined outcome measure can be a pragmatic salutogenic quality indicator. The aim of this study is to describe and compare birth outcomes in Nordic countries by using the two different composite outcome measures: *Births without major interventions and complications and Births without obstetric interventions and complications*.

**Methods:** The Nordic birth registers were used to compare and describe prevalence of births defined by the two composite outcomes in Denmark, Sweden, Iceland and Norway in 2020. The results are stratified by Robson group. *Births without major interventions and complications* is defined as births without vacuum extraction, cesarean section, postpartum hemorrhage >1500 mL, blood transfusion, severe sphincter tear or Apgar <7 at 5 minutes. *Births without obstetric interventions and complications* is defined as spontaneous start, no epidural, no oxytocin, no perineotomy, non‐instrumental vaginal birth, Apgar >6 at 5 minutes, PPH >1500 mL.

**Results:** In progress. Estimated sample size is 200 000 women. Preliminary analyses show differences between the Nordic countries. For example, the rate of *Births without major interventions and complications* in Norway in Robson group 1 is 70% vs 76% in Sweden, mainly due to the use of vacuum extraction. Rates in Robson group 3 is 93% in Norway and 95% in Sweden.

**Conclusions:** Presented at conference.

## Continued vs discontinued oxytocin stimulation in the active phase of labor (CONDISOX): individual management based on artificial intelligence

### **Sidsel Boie** ^1,2,3^, Julie Glavid^2,4^, Pinar Bor^2,3^, Philip Steer^5^, Anders Hammerich Riis^6^, Bo Thiesson^6^, Niels Uldbjerg^2^

#### ^1^Department of Obstetrics and Gynecology, Aalborg University Hospital, Aalborg, Denmark; ^2^Department of Clinical Medicine, Aarhus University, Aarhus, Denmark; ^3^Department of Obstetrics and Gynecology, Randers Regional Hospital, Randers, Denmark; ^4^Department of Obstetrics and Gynecology, Aarhus University Hospital, Aarhus, Denmark; ^5^Academic Department of Obstetrics and Gynecology, Chelsea and Westminster Hospital, Imperial College London, London, UK; ^6^Enversion A/S, Aarhus, Denmark

**Introduction/Purpose:** Current guidelines regarding oxytocin stimulation are not tailored to individuals, as they are based on randomized controlled trials. The objective of the study was to develop an artificial intelligence model for individual prediction of the risk of cesarean delivery in women reaching the active phase of labor after oxytocin stimulation for induced labor.

**Methods:** Design: Secondary analysis of data from the CONDISOX randomized controlled trial of discontinued vs continued oxytocin infusion in the active phase of labor.

**Population:** 1060 women with a 6‐cm cervical dilation after labor induction. The cesarean section rate was 16.1% (discontinued oxytocin) and 14.1% (continued oxytocin).

**Clinical relevance:** A difference in risk of cesarean section above >5 percentage points between continued and discontinued oxytocin.

The tailored prediction model included variables known before labor induction as well as variables describing the course of the labor induction: extreme gradient boosting (XGBoost) software, Shapley additive explanation (SHAP) values, SHAP plots and force plots.

**Results:** Women who delivered vaginally were more likely to be parous, taller, have a lower estimated birthweight, and to be stimulated with a smaller amount of oxytocin.

The tailored model favored continuation in 81 women (7.6% of 1060), discontinuation in 27 women (2.5%), and neither in 952 (90%) women.

In all, 138 (11.5%) of the randomized women did not receive the allocated treatment, primarily due to rapid progression. Most probably these women would benefit from discontinuation.

**Conclusions:** In women undergoing labor induction, this model can assist the mother and clinician in tailored management of oxytocin stimulation.

## Cord clamping time and placental transfusion in cesarean sections: an observational multicenter study

### **Anna Sand** ^1,2,3^, Deborah Utjés^2^, Ola Andersson^3,4^, Jenny Svedenkrans^3,5,6^

#### ^1^Department of Women's and Children's Health, Karolinska Institutet, Stockholm, Sweden; ^2^Department of Gynecology and Obstetrics, Karolinska University Hospital, Stockholm, Sweden; ^3^Department of Clinical Science, Lund University, Lund, Sweden; ^4^Department of Pediatric Surgery and Neonatology, Skåne University Hospital, Malmö/Lund, Sweden; ^5^Department of Clinical Science, Intervention and Technology (CLINTEC), Karolinska Institutet, Stockholm, Sweden; ^6^Department of Neonatology, Karolinska University Hospital, Stockholm, Sweden

**Introduction/Purpose:** Delaying cord clamping may have several advantages for the infant, including increased placental transfusion, improved iron stores and improved neurodevelopment. The effect of cord clamping time on placental transfusion in elective and emergency cesarean sections was evaluated. Furthermore, negative short‐term side effects of delayed cord clamping were analyzed.

**Methods:** Observational study on cesarean deliveries at ≥35 gestational weeks. Indication for cesarean section, cord clamping time, timing of uterotonics administration and estimated maternal blood loss were recorded at birth. Placentas were drained from remaining blood, which was weighed and registered. The relation between cord clamping time, remaining placental blood volume and maternal blood loss was analyzed.

**Results:** Measurements were available for 143 placentas. Of these, 24 (16.8%) were delivered after emergency cesarean section. Median (IQR) cord clamping time was 68 (56–91) s, mean (SD) remaining blood in the placenta was 35.5 (28.8) g. There was a significant negative correlation between cord clamping time in minutes and remaining placental blood in an adjusted linear regression model (*B* = −9.2, *P* = 0.005). There was no correlation between cord clamping time and maternal blood loss (*B* = 1,5, *P* = 0.12). Administration of uterotonics before cord clamping was associated with less residual placental blood volume (mean difference 13.4 mL, *P* = 0.008). No difference was found between emergency and elective cesarean sections.

**Conclusions:** An expectant management of cord clamping may enhance placental transfusion in cesarean sections without increased maternal blood loss. Timing of uterotonics administration may affect placental transfusion. Larger studies are needed to evaluate the importance of indication for cesarean section.

## COVID‐19 in pregnancy: a meta‐analysis of population‐based surveillance in five Nordic countries yields lessons for current and future pandemics

### **Reetta Varpula** ^1^, **Reetta Varpula** ^1^, Outi Äyräs^1^, Anna Aabakke^2,3^, Lone Krebs^4,5^, Teresia Svanvik^6^, Aura Pyykönen^1^, Eva Jonasdottir^7^, Hilde Engjom^8^

#### ^1^Helsinki University Hospital Department of Obstetrics and Gynecology and Helsinki University, Helsinki, Finland; ^2^Department Obstetrics and Gynecology, Copenhagen University Hospital – North Zealand Hospital, Hillerød, Denmark; ^3^Department of Obstetrics and Gynecology, Copenhagen University Hospital Holbæk, Denmark; ^4^Department of Clinical Medicine, University of Copenhagen, Copenhagen, Denmark; ^5^Department of Obstetrics and Gynecology, Copenhagen University Hospital – Amager and Hvidovre, Hvidovre, Denmark; ^6^Department of Obstetrics and Gynecology, University of Gothenburg, Gothenburg, Sweden; ^7^Department of Obstetrics and Gynecology, Landspitali University Hospital, Reykjavik, Iceland; ^8^Haukeland University Hospital, Bergen, and The Norwegian Institute of Public Health, Oslo, Norway

**Introduction/Purpose:** The aim of this study was to assess the frequency of COVID‐19‐related hospital admissions of pregnant women in the Nordic countries during the first year of the pandemic in relation to timing and implementation of public health restrictions for limiting viral transmission. We also analyzed the severity of COVID‐19 disease and associated maternal risk factors and perinatal outcomes.

**Methods:** This is a meta‐analysis of population‐based cohort studies in the five Nordic countries with national or regional surveillance in the NOSS collaboration: Denmark, Finland, Iceland, Norway and Sweden (regional). Hospital admissions of women giving birth from March 1 to December 31, 2020 with a positive SARS‐CoV‐2 PCR test ≤14 days prior to admission were included. Admissions were further categorized as admission for COVID‐19 symptoms or for non‐COVID‐19 reasons. The Stringency Index was used to assess public health restrictions.

**Results:** Among a total of 392 382 maternities, 600 women were admitted, 137 due to COVID‐19 symptoms. The estimated incidence of admission due to COVID‐19 was 0.5 per 1000 maternities, ranging from no admissions in Iceland to 1.9 per 1000 maternities in the Swedish regions. The difference in incidence was related to the varying implementation of public health restrictions, most notably when comparing Sweden to the other Nordic countries. Maternal risk factors for admission due to symptomatic COVID‐19 were obesity and a history of asthma.

**Conclusions:** Suppression policies protected pregnant women from severe COVID‐19 disease prior to the availability of individual protection with vaccines. The risk factors associated with COVID‐19 admissions were similar to previous studies.

## Definitions of failed induction of labor: a systematic review

### **Emma Louise Christiansen**, Lise Qvirin Krogh, Julie Glavind

#### Department of Obstetrics and Gynecology, Aarhus University Hospital, Aarhus, Denmark

**Introduction/Purpose:** Although failed induction of labor (FIOL) is a commonly used term, no agreed definition exists. This may challenge the comparability between studies investigating methods or regimens for induction, in terms of both efficacy and clinical outcomes, and also hampers the usability in clinical practice. The purpose of this study was to describe and compare definitions of FIOL reported in the research literature.

**Methods:** A systematic literature search was conducted in PubMed and Embase databases. We included randomized controlled trials (RCT) published between January 2000 and April 2022 with a reported definition of FIOL. We extracted the definition from each study and categorized the various components of the definitions.

**Results:** We identified 15 RCTs reporting a total of 17 definitions of FIOL. None of the definitions was identical, and there was a large heterogeneity among the definitions. Eight different components were included in the definitions: the duration of induction (70.6%), failure to enter active labor (23.5%), cervical dilation (23.5%), Bishop score (23.5%), number of doses of induction medicaments (23.5%), frequency or sufficiency of contractions (23.5%), the inability to perform amniotomy (23.5%), and delivery from cesarean section (17.6%).

**Conclusions:** There is no consensus on how to define FIOL in RCTs investigating induction of labor. This calls for caution when evaluating and comparing induction methods/regimens and calls for a standardized definition of FIOL.

## Depressive symptoms and sleep disturbances in late pregnancy: associations with experience of induction of labor with a catheter

### **Henna Haavisto** ^1^, Kirsi Rinne^1^, Terhi Kolari^2^, Ella Anttila^1^, Elina Ojala^1^, Päivi Polo‐Kantola^1^

#### ^1^Department of Obstetrics and Gynecology, Turku University Hospital and University of Turku, Turku, Finland; ^2^Department of Biostatistics, University of Turku, Turku, Finland

**Introduction/Purpose:** Depressive symptoms and sleep disturbances have been found to be associated with negative labor experiences. However, the associations between maternal depressive symptoms and sleep disturbances and the experience of induction of labor (IOL) remain unknown. In this study, we evaluated these associations with balloon catheter IOL.

**Methods:** A prospective study was conducted on 106 women with planned IOL. Depressive symptoms were evaluated with The Edinburgh Postnatal Depression Scale and sleep disturbances with the Basic Nordic Sleep Questionnaire at the beginning of IOL. The IOL experience was investigated both during the IOL and postpartum with the same nine visual analog scale questions.

**Results:** Regarding sleep disturbances, during the IOL, women with worse general sleep quality were less satisfied (*P* = 0.019), less relaxed (*P* = 0.008) and experienced more pain in general (*P* = 0.002). Furthermore, women who experienced difficulties falling asleep were less relaxed (*P* = 0.009), reported more general pain (*P* < 0.001) and contraction pain (*P* = 0.005). Postpartum, women with worse general sleep quality reported more general pain (*P* = 0.003), whereas women with longer sleep duration and those with higher sleep loss reported more anxiety (*P* = 0.009 and *P* = 0.024, respectively). Additionally, women who woke up too early in the morning were less satisfied (*P* = 0.042), less relaxed (*P* = 0.004) and reported more general pain (*P* = 0.018). Depressive symptoms were associated with less general pain postpartum (*P* = 0.027).

**Conclusions:** Women with sleep disturbances during pregnancy were more likely to report more negative experiences during the IOL. Specifically, they reported more pain, feeling less relaxed and they were less satisfied with IOL.

## Diabetes in pregnancy associated with postpartum depression

### **Richelle Duque Björvang** ^1,2^, Iliana Liakea^3^, Beatrice Carpentsier^1,4^, Zoltan Kozinszky^5^, Alkistis Skalkidou^1^, Emma Fransson^1,6^

#### ^1^Department of Women's and Children's Health, Uppsala University, Uppsala, Sweden; ^2^Department of Clinical Science, Intervention and Technology, Karolinska Institute, Stockholm, Sweden; ^3^Department – Behavioral Science Institute, Radboud University, Nijmegen, Netherlands; ^4^Department of Public Health and Caring Sciences, Uppsala University, Uppsala, Sweden; ^5^Department of Obstetrics and Gynecology, Danderyd Hospital, Stockholm, Sweden; ^6^Department of Microbiology, Tumor and Cell Biology, Karolinska Institute, Stockholm, Sweden

**Introduction/Purpose:** Diabetes is often associated with depression, and both conditions are prevalent during pregnancy. Studies investigating the association between diabetes in pregnancy (DIP) and perinatal depression (PND) are inconsistent. The aim of this study was to assess their association in a Swedish population‐based cohort.

**Methods:** Using the BASIC (Biology, Affect, Stress, Imaging and Cognition) project, we have identified 4769 participants aged 18–48 years with available data on DIP and PND. The diagnosis of DIP was extracted from medical records and national register data, and was then classified as pre‐gestational (PGDM), gestational (GDM) or unspecified diabetes. PND was assessed using psychometric instruments, interviews and register data. It was categorized into antepartum depression (APD) or postpartum depression (PPD). Logistic regression was employed, with DIP as exposure and APD or PPD as outcomes. Models were adjusted for age, pre‐pregnancy body mass index, parity and depression (Model 1) and traumatic life events (Model 2).

**Results:** Of 4769 women, 1605 (32.2%) women had depressive symptoms at some time during the study period (PND) (8.2% APD, 11.6% PPD and 12.4% both APD and PPD). DIP had a prevalence of 1.3%. DIP accounted for a twofold higher odds for PPD but not for APD. When data were stratified by type of diabetes, PGDM and unspecified diabetes remained significant predictors, with fourfold odds for PPD. GDM was not significantly associated with APD or PPD.

**Conclusions:** Our study shows that DIP is associated with a higher risk for PPD and might be considered a risk factor when screening for high‐risk groups for PPD.

## Evaluation of CTG guidelines and their impact on clinical decision

### **Frida Ekengård**, Monika Cardell, Andreas Herbst

#### Department of Obstetrics and Gynecology, Skåne University Hospital, Institution of Clinical Sciences Lund University, Lund, Sweden

**Introduction/Purpose:** FIGO introduced a CTG guideline in 2015 without prior evaluation which was implemented in Sweden in 2017 in a modified version. This is an evaluation of these templates and the former Swedish template, based on the FIGO 1987 guideline.

**Methods:** CTG tracings from neonates born with acidemia in the first and second stage of labor and tracings from the corresponding stage from non‐acidemic neonates were interpreted by midwives and physicians. The impact of the two Swedish guidelines on decision making was examined.

**Results:** The FIGO guideline had a sensitivity of 71% in the first stage of labor and 50% in the second in identifying neonates with acidemia. The former Swedish guideline had a sensitivity in the first stage of 95% and a specificity of 90%, respectively, compared with 87% and 56%, in the second stage. The new Swedish guideline had a sensitivity for classification pathological in the first stage of 77% and in the second stage 62%. For cut‐off for suspicious patterns, the sensitivity increased to 90% for the first, and 83% for the second stage, with corresponding specificities at 93% and 68%. The guideline in use affected decisions. With the current Swedish guideline, the agreement was κ = 0.77 between classification pathological and need for intervention.

**Conclusions:** With the current Swedish guideline awareness must be raised for suspicious tracings, which may warrant intervention. The former Swedish guideline had the best sensitivity but a lower specificity in the second stage, carrying a risk of unnecessary interventions. The new international template is not safe to use.

## Evaluation of intrapartum rapid detection of group B streptococci (GBS) tests in women with GBS bacteriuria during pregnancy

### Gitte Nyvang Hartmeyer^1^, Anna Kjær Redin^1^, Emma Krusborg Kristensen^1^, Marianne Nielsine Skov^1^, **Christina Anne Vinter** ^2,3^

#### ^1^Department of Clinical Microbiology, Odense University Hospital, Odense, Denmark; ^2^Department of Gynecology and Obstetrics, Odense University Hospital, Odense, Denmark; ^3^Department of Clinical Research, University of Southern Denmark, Odense, Denmark

**Introduction/Purpose:** Group B streptococci (GBS) remain a leading cause of neonatal sepsis despite declines due to intrapartum antibiotic prophylaxis (IAP). Many pregnant women are treated with IAP to prevent vertical transmission of GBS to the fetus. In Denmark, the procedure to prevent GBS disease in neonates is based on a risk assessment strategy, where women with certain risk factors are given IAP. One of these is GBS bacteriuria during pregnancy. Newer data suggest that women with GBS colonization, tested for GBS during labor, should only receive IAP when PCR is positive for GBS. Intrapartum rapid polymerase chain reaction (PCR) testing for GBS was introduced at Odense University Hospital (OUH) in 2019.

**Aim:** To examine the use of PCR testing during labor in women with GBS bacteriuria in pregnancy, in order to determine how many could potentially avoid IAP.

**Methods:** Data from the Department of Clinical Microbiology at OUH from 2019 to 2021 from pregnant women with GBS bacteriuria were extracted from the laboratory system. Data were correlated with data for using intrapartum rapid GBS PCR test in the same period.

**Results:** The study included 1034 GBS‐positive urine samples from 1001 women with bacteriuria. Results correlated with intrapartum PCR for GBS are seen in Table 1

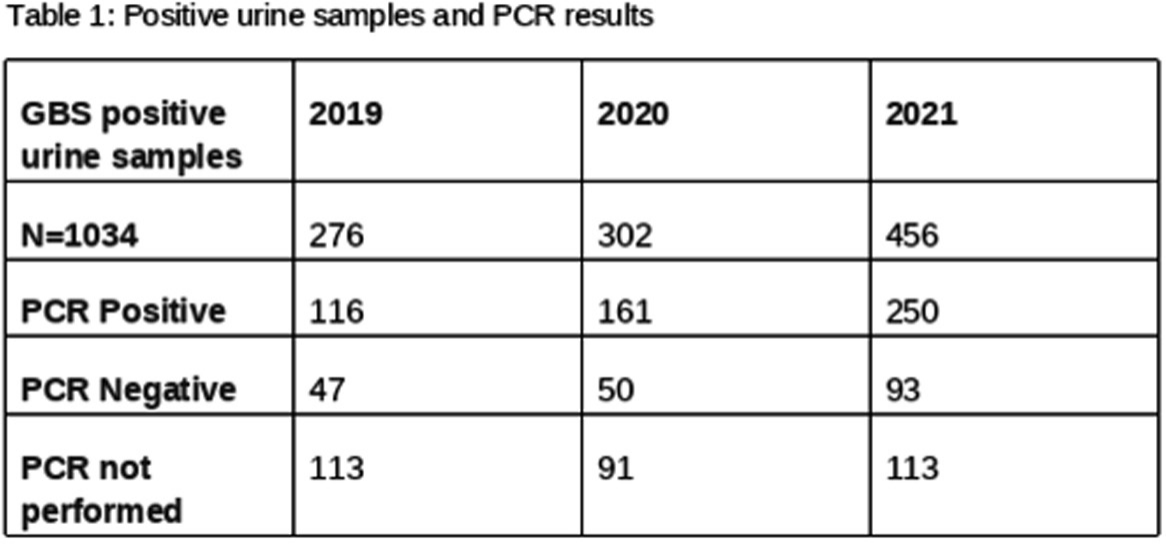

**Conclusions:** In total, 70% of women were tested with intrapartum PCR, and 26% were GBS‐negative and could potentially avoid IAP. However, 30% of women with GBS bacteruria in pregnancy did not have an intrapartum PCR test and reasons for this will be further explored using data from medical journals.

## Evaluation of the symphysis‐fundal height curve to estimate fetal growth – a Swedish retrospective cohort study

### **Valerie Stålberg**, Anna Granat, Marie Bladh, Ann Josefsson, Caroline Lilliecreutz

#### Division of Children's and Women's Health, Department of Biomedical and Clinical Sciences, Linköping University, Linköping, Sweden

**Introduction/Purpose:** To detect abnormal fetal growth during pregnancy is important and most challenging. Symphysis‐fundal height (SFH) measurement is an established method in the antenatal care setting to identify fetuses at risk, but evidence for the method is lacking. Thus, the aim was to evaluate the SFH‐curves’ ability to estimate fetal growth.

**Methods:** Adult women (*n* = 3113) with singleton pregnancies giving birth between May 2021 and January 2022 were included. Data was extracted from medical records and the Obstetrix®database. The SFH‐curves were categorized as normal (A), 2 SD below normal (B) or 2 SD above normal (C).

**Results:** The majority of women were classified in the A‐curve group (86.0%, *n* = 2688) and gave birth to an appropriate for gestational age (AGA) child in 95.9%, a small‐for‐gestational‐age (SGA) child in 1.9% and a large‐for‐gestational‐age (LGA) child in 2.2% of the pregnancies. The corresponding numbers for women classified in the B‐curve group (3.8% *n* = 117) were 90.6%, 9.4% and 0%, and similar numbers of women were classified in the C‐curve (9.9% *n* = 308) (79.2%, 0.6% and 20.1%), respectively. The sensitivity and specificity of the SFH‐curves to detect an SGA and LGA child were 9.4%, 1.7% and 51.2%, 48.8%, respectively. Among women with SGA (*n* = 63) and LGA (*n* = 121) children, 17.5% and 51.2% were correctly identified using the SFH‐curve.

**Conclusions:** This study indicates that the SFH‐curve is unreliable for detecting abnormal fetal growth, especially for children born SGA. Since the majority of pregnant women with abnormal SFH‐curves give birth to AGA children, there is a potential risk for unnecessary interventions.

## Fetal membrane cells in maternal blood at term

### **Emmeli Mikkelsen** ^1,2^, Katarina Ravn^3^, Torben Steiniche^1,4^, Berthold Huppertz^5^, Lotte Hatt^3^, Palle Schelde^3^, Ramkumar Menon^6^, Ripudaman Singh^3^, Niels Uldbjerg^1,2^

#### ^1^Department of Clinical Medicine, Aarhus University, Aarhus, Denmark; ^2^Department of Obstetrics and Gynecology, Aarhus University Hospital, Aarhus, Denmark; ^3^ ARCEDI Biotech, Vejle, Denmark; ^4^Department of Histopathology, Aarhus University Hospital, Aarhus, Denmark; ^5^Division of Cell Biology, Histology, and Embryology, Gottfried Schatz Research Center, Medical University of Graz, Graz, Austria; ^6^Department of Obstetrics and Gynecology, University of Texas Medical Branch at Galveston, TX, USA

**Introduction/Purpose:** Rupture of the fetal membranes is preceded by cellular senescence and the formation of “microfractures” in both term and preterm pregnancies. We hypothesize that this process is associated with the shedding of cells through the microfractures into maternal blood. Previously, by RNA sequencing, we identified specific gene markers highly expressed in fetal membrane cells compared with maternal white blood cells. This study aimed to detect fetal membrane cells in maternal blood using antibodies against these markers.

**Methods:** Blood samples (30 mL) were drawn at term (GA +37 weeks) from 16 pregnant women with a normal pregnancy. All women had rupture of membranes and contractions. Eleven cell‐surface antibodies were used to enrich cells by magnetic activated cell sorting (MACS). Hereafter, the cells were stained using a fluorescent‐labeled pool of 12 cytoplasmic antibodies and isolated by florescent activated cell sorting (FACS). Their fetal origin was verified by STR analysis.

**Results:** Fetal membrane cells were identified in six of 16 blood samples. The concentration of cells was 12, 2, 1, 2, 1 and 1 per 30 mL of blood, respectively. They were identified in blood from pregnant women with prelabor, artificial and spontaneous rupture of membranes.

**Conclusions:** We report successful isolation of fetal membrane cells from maternal blood at term using specific markers. The number of fetal membrane cells is sparse and few at term; however, the perspective is that their number increases with threatening PPROM. If so, they provide a potential biomarker for early diagnosis of high‐risk pregnancies for PPROM indicative of membrane dysfunction.

## First trimester maternal serum PAPP‐A and free Β‐HCG levels and risk of SGA or LGA with GDM patients and controls

### **Tiina Kantomaa** ^1^, Marja Vääräsmäki^1^, Mika Gissler^2^, Jaana Nevalainen^1^

#### ^1^Department of Obstetrics and Gynecology, Oulu University Hospital, Oulu, Finland, Research Unit of Clinical Medicine, Wellbeing Services County of North Ostrobothnia, Oulu, Finland; ^2^Finnish Institute for Health and Welfare, Helsinki, Finland; Region Stockholm, Academic Primary Health Care Center, Stockholm, Sweden; Karolinska Institute, Department of Molecular Medicine and Surgery, Stockholm, Sweden

**Introduction/Purpose:** Gestational diabetes mellitus (GDM), small‐ (SGA) and large‐for‐gestational age (LGA) birthweights are associated with increased morbidity. We studied the association of low or high pregnancy‐associated plasma protein‐A (PAPP‐A) and free beta human chorionic gonadotropin (fβ‐hCG) MoM levels with SGA or LGA in pregnancies with or without GDM.

**Methods:** In this retrospective cohort study, 23 482 women with singleton pregnancies who participated in first trimester combined screening in Northern Finland and delivered in 2014–2018, were included. PAPP‐A and β‐hCG MoM were analyzed as a part of routine combined screening protocol. Women with or without GDM were divided into groups with PAPP‐A and fβ‐hCG MoM levels below or above 10 and 90 percentiles.

**Results:** In GDM pregnancies (*n* = 4697) the proportion of SGA was 2.6% and LGA 4.5% compared with 3.3% (*P* = 0.011^2^) and 1.8% (*P* < 0.001^2^) in 18 392 controls, respectively. In low PAPP‐A MoM groups, adjusted odds ratios (aOR) for SGA were 2.0 (95% confidence interval [CI] 1.3–3.2) in GDM and 2.6 (95% CI 2.1–3.2) in the control group. In low fβ‐hCG MoM groups, aORs for SGA were 1.0 (95% CI 0.6–1.8) in GDM and 2.0 (95% CI 1.6–2.5) in the control group. The aOR for LGA were not statistically significant in high PAPP‐A groups. In ≥90 percentile fβ‐hCG cases, aOR in GDM group was 1.6 (1.0–2.4) for LGA and 1.2 (0.9–1.7) in the control group.

**Conclusions:** Low PAPP‐A MoM was associated with SGA both in GDM and control groups and low fβ‐hCG MoM only in the control group. High fβ‐hCG MoM was associated with LGA in GDM pregnancies.

## Full‐term induction of labor vs expectant management in women with obesity: systematic review and meta‐analysis

### **Lise Qvirin Krogh** ^1,2^, Julie Glavind, Tine Brink Henriksen^2,3^, Jim Thornton^4^, Jens Fuglsang^1,2,5^, Sidsel Boie^1,6^

#### ^1^Department of Obstetrics and Gynecology, Aarhus University Hospital, Aarhus, Denmark; ^2^Department of Clinical Medicine, Aarhus University, Aarhus, Denmark; ^3^Department of Pediatrics, Aarhus University Hospital, Aarhus, Denmark; ^4^Department of Obstetrics and Gynecology, Nottingham University, Nottingham, UK; ^5^Steno Diabetes Center, Aarhus University Hospital, Aarhus, Denmark; ^6^Department of Obstetrics and Gynecology, Aalborg University Hospital, Aalborg, Denmark

**Introduction/Purpose:** In pregnancy, the risk of cesarean delivery is doubled in women with obesity compared with women of normal weight. Observational studies suggest lower cesarean delivery rates with induction of labor compared with expectant management in women with obesity. The objective of this systematic review is to synthesize evidence on full‐term induction of labor vs expectant management by cesarean delivery and other adverse maternal and neonatal outcomes in women with obesity.

**Methods:** The literature search was performed in PubMed, EMBASE, Scopus, Clinicaltrials.gov and Cochrane Library. Cohort or randomized controlled trials were eligible if they compared induction of labor full‐term with expectant management and included women with a BMI ≥30 kg/m^2^. The primary outcome was cesarean delivery. Secondary outcomes on maternal and neonatal mortality and morbidity were also evaluated. Risk of bias was assessed using the Risk of Bias in Non‐Randomized Studies of Interventions tool. Only studies with low or moderate risk of bias contributed data to the meta‐analysis. Data were synthesized using random‐effects models.

**Results:** Thirteen studies were identified, of which four observational studies contributed with data to the meta‐analysis for the primary outcome. Full‐term induction of labor may reduce the risk of cesarean delivery compared with expectant management (19.7% vs 24.5%; relative risk 0.71; 95% confidence interval 0.63–0.81; low‐level certainty), as well as of several other maternal and neonatal outcomes.

**Conclusions:** In this meta‐analysis of observational studies, induction of labor at full‐term in women with obesity may be associated with reduced cesarean delivery compared with expectant management.

## High vitamin D levels moderate the prothrombotic profile in pregnancy by reducing plasminogen activator inhibitor 1

### **Isabella Hangaard Rüdiger** ^1^, Matilde Kanstrup Andersen^1,2^, Anna Louise Vestergaard^1,3^, Mustafa Vakur Bor^4,5^, Agnete Larsen^2^, Pinar Bor^1^

#### ^1^Department of Obstetrics and Gynecology, Regional Hospital of Randers, Randers, Denmark; ^2^Department of Biomedicine, Aarhus University, Aarhus, Denmark; ^3^Department of Clinical Medicine, Aarhus University, Aarhus, Denmark; ^4^Department of Regional Health Research, Thrombosis Research, University of Southern Denmark, Esbjerg, Denmark; ^5^Department of Clinical Biochemistry, University Hospital of Southern Denmark, Esbjerg, Denmark

**Introduction/Purpose:** Pregnancy is associated with a prothrombotic profile with an increased thromboembolic risk. Vitamin D deficiency has been suggested as an independent risk factor for thrombo‐embolic events, though the underlying mechanism is not delineated. Notably, vitamin D deficiency is common in pregnancy. However, pregnant women are commonly excluded from studies investigating the hemostatic system.

Our purpose is to investigate whether the serum level of vitamin D affects hemostasis in late first‐trimester pregnant women.

**Methods:** A cross‐sectional study comparing the hemostatic profile of pregnant women defined as vitamin D‐deficient (≤50 nmol/L) (*n* = 70) and vitamin D‐sufficient (≥100 nmol/L) (*n* = 59). Biomarkers of primary and secondary hemostasis (von Willebrand factor [vWF], fibrinogen, thrombin generation, factor VIII), fibrinolysis (plasminogen activator inhibitor [PAI‐1], tissue plasminogen activator, D‐dimer) and C‐reactive protein were investigated.

**Results:** The PAI‐1 level was statistically significantly decreased in the group of pregnant women with serum vitamin D levels ≥100 nmol/L after adjusting for pre‐pregnancy body mass index, smoking and use of fish oil supplements, indicating possible thrombo‐protective properties of vitamin D. A significantly higher level of vWF was also found, but this finding was not statistically significant after adjustment for smoking.

**Conclusions:** Vitamin D deficiency is associated with increased PAI‐1 levels in pregnant women and therefore with a prothrombotic profile. Further research is needed to determine the optimal vitamin D levels in pregnancy and understand the links between vitamin D homeostasis and pregnancy outcome.

## History of induced abortion is associated to fear of childbirth in primiparous women. A Finnish population‐based register study

### **Venla Kemppainen** ^1^, Oskari Heikinheimo^2^, Maarit Niinimäki^3^, Maarit Mentula^1^

#### ^1^Helsinki University Hospital, Helsinki, Finland; ^2^University of Helsinki, Helsinki, Finland; ^3^Oulu University Hospital, Oulu, Finland

**Introduction/Purpose:** This population‐based register study evaluated the association of induced abortion (IA) and fear of childbirth (FOC). Study population was primigravid women with IA between 2000 and 2015 whose subsequent pregnancy ended in a live singleton birth up to 2017. The study cohort had a control group (1:3) whose first pregnancy ended in a singleton live birth (matched by age at the time of delivery and residential area). Our primary outcome was the incidence of FOC. Secondarily we assessed risk factors for FOC.

**Methods:** Population‐based register study.

**Results:** The study cohort consisted of 21 455 women with a history of IA and 63 425 controls. The incidence of FOC was higher in women with a history of IA than in controls (5.6% vs 3.7%, *P* < 0.001). Factors increasing the odds for FOC were history of IA (adjusted odds ratio [aOR] 1.20, 95% confidence interval [CI] 1.11–1.30) or a psychiatric diagnosis (aOR 3.48, 95% CI 3.15–3.83), advanced maternal age (30–39 years old, aOR 1.55, 95% CI 1.43–1.67 [*P* < 0.001]; ≥40 years old, aOR 3.00, 95% CI 2.37–3.77 [*P* < 0.001]) and smoking (aOR 1.20, 95% CI 1.11–1.31 [*P* < 0.001]). Women living in densely populated or rural areas, and those with lower socioeconomic class had lower odds for FOC.

**Conclusions:** A history of IA is associated with increased odds for FOC in subsequent pregnancy. However, the associations of FOC with history of psychiatric diagnosis and elevated maternal age (especially ≥40 years old) are more pronounced.

## How do different childbirth experience scales predict childbirth‐related posttraumatic stress symptoms and disorder?

### **Tia Mäkelä** ^1,2^, Terhi Saisto^1,2^, Katariina Salmela‐Aro^3^ , Hanna Rouhe^1,2^

#### ^1^Helsinki University Hospital, Department of Obstetrics and Gynecology, Helsinki, Finland; ^2^University of Helsinki, Helsinki, Finland; ^3^University of Helsinki, Faculty of Educational Sciences, Helsinki, Finland

**Introduction/Purpose:** At worst, childbirth experience is not only negative but also traumatic and may lead to post‐traumatic stress disorder (PTSD). PTSD after childbirth possesses severe and lasting effects. Screening of the childbirth experience may speed up early recognition of PTSD. Systematic reviews have no set consensus on how and when to measure childbirth experience and what its clinical implication is. We aimed to identify a reliable and simple childbirth experience scale suitable for screening with minimum risk of missing PTSD.

**Methods:** This study evaluated 1527 unselected women's childbirth experience with the following scales: Wijma Delivery Experience Questionnaire (W‐DEQ‐B), Delivery Satisfaction Scale (DSS) and Visual Analogue Scale (VAS). VAS was first measured less than a week (VAS1) after childbirth and then, together with the others, a couple of months after childbirth (VAS2). The ability of the scales to identify PTSD (measured with Traumatic event scale, TES) was evaluated and compared with ROC analysis. Diagnostic accuracy and clinical usefulness were used to suggest cut‐off values for all scales.

**Results:** Altogether, 14.6% of the responders experienced childbirth negatively, and 33% of them led to partial/full PTSD. However, a traumatic childbirth experience (in 9.7%) led to partial/full PTSD in 63.8%. W‐DEQ‐B showed the highest recognition of partial/full PTSD, as an area under the ROC curve was 0.96 in W‐DEQ‐B, 0.92 in VAS2, 0.91 in DSS, and 0.82 in VAS1.

**Conclusions:** All studied scales recognized partial/full PTSD well. Although W‐DEQ‐B performed best in clinical circumstances it is long and arduous to complete. VAS with a cut‐off value of 50 mm is most suitable for screening.

## Immunological development in preeclampsia and gestational hypertension assessed by longitudinal serum cytokine profiling

### **Signe Haaland Buer** ^1,2^, Anders Hagen Jarmund^1,2^, Guro F. Giskeødegård^3^, Mariell Ryssdal^1,2^, Live Marie Tobiesen Stokkeland^1,2^, Bjørg Steinkjer^1,2^, Line Bjørge^4^, Tone Shetelig Løvvik^1,5^, Signe Stafne^3^, Trine Moholdt^6^, Ann‐Charlotte Iversen^1,2,5,7^, Eszter Vanky^1,5,7^

#### ^1^Department of Clinical and Molecular Medicine, Norwegian University of Science and Technology (NTNU), Trondheim, Norway; ^2^Center of Molecular Inflammation Research (CEMIR), Norwegian University of Science and Technology (NTNU), Trondheim, Norway; ^3^Department of Public Health and Nursing, Norwegian University of Science and Technology (NTNU), Trondheim, Norway; ^4^Department of Gynecology and Obstetrics, Haukeland University Hospital, and Center for Cancer Biomarkers CCBIO, Department of Clinical Science, University of Bergen, Bergen, Norway; ^5^Department of Obstetrics and Gynecology, St. Olav's Hospital, Trondheim University Hospital, Trondheim, Norway; ^6^Department of Circulation and Medical Imaging, Norwegian University of Science and Technology (NTNU); and Department of Women's Health, St. Olav's Hospital, Trondheim University Hospital, Trondheim, Norway; ^7^These authors contributed equally.

**Introduction/Purpose:** Preeclampsia and gestational hypertension (GH) share common characteristics such as hypertension and deviant immune responses during pregnancy, whereas placental dysfunction is specific to preeclampsia. Through broad cytokine profiling in maternal serum, we have previously revealed distinct maternal immunological status in preeclampsia and GH in early pregnancy. This study aimed to explore disease‐specific immunologic development throughout pregnancy in women with and without preeclampsia and GH.

**Methods:** Women with normal pregnancies (*n* = 1463), pregnancies with preeclampsia (*n* = 119) or GH (*n* = 44) were included from six cohorts. Maternal serum samples (*n* = 5111) were analyzed for 27 cytokines (Bio‐Plex Pro Human Cytokine 27‐plex Assay) and C‐reactive protein (CRP). Repeated measures‐ASCA+ was used to identify differences in serum cytokine profiles.

**Results:** Preeclampsia and GH were associated with increased immune activation throughout pregnancy compared with normal pregnancies, and the effect was stronger in preeclampsia than in GH. Cytokine profiles in preeclampsia deviated most from normal pregnancies in the first and third trimesters, with the strongest contribution from interleukin (IL)‐6, eotaxin, MCP‐1 and human interferon‐inducible protein (IP)‐10. In contrast, the GH‐induced immune activation deviated most from normal pregnancies in the second trimester and was promoted by IP‐10, IL‐13, IL‐8 and CRP. When comparing preeclampsia and GH, the cytokines PDGF‐BB, IP‐10 and MCP‐1 showed the most contrasting patterns.

**Conclusions:** Preeclampsia and GH induce a broad maternal immune activation that starts before onset of clinical symptoms and persists throughout pregnancy. The disorders differ from each other in having distinct serum cytokine patterns and strikingly different timing of immune activation, supporting separate underlying pathophysiology.

## Improving teams’ clinical performance in the labor and delivery unit using video‐assisted debriefing of real‐life events

### **Lena Have Rosvig** ^1,2^, Lone Hvidman^3^, Niels Uldbjerg^2,3^, Ole Kierkegaard^1^, Stina Lou^2,4,5^, Tanja Manser^6^, Louis Halamek^7^, Lise Brogaard^2,3^

#### ^1^Department of Obstetrics and Gynecology, Horsens Regional Hospital, Horsens, Denmark; ^2^Department of Clinical Medicine, Aarhus University, Aarhus, Denmark; ^3^Department of Obstetrics and Gynecology, Aarhus University Hospital, Aarhus, Denmark; ^4^ DEFACTUM ‐ Public Health & Health Services Research, Aarhus, Denmark; ^5^Fetal Medicine, Aarhus University Hospital, Aarhus, Denmark; ^6^ FHNW School of Applied Psychology, University of Applied Sciences and Arts Northwestern Switzerland, Olten, Switzerland; ^7^Department of Pediatrics, Stanford University, Stanford, CA, USA

**Introduction/Purpose:** Video‐assisted debriefings (VAD) of real‐life emergencies offer an opportunity to review the care delivered in high‐risk, time‐critical situations. One emergency for which VAD may be of value is postpartum hemorrhage (PPH). The objective of this study was to examine the effects of VAD of real‐life clinical events on the obstetric teams’ management of PPH.

**Methods:** The present study was a non‐randomized controlled clinical trial. The study was conducted in Denmark in the obstetric departments at Horsens Regional Hospital and Aarhus University Hospital. *Inclusion criteria*: Vaginal deliveries with blood loss ≥1000 mL (major PPH), gestational age ≥37 weeks. All delivery rooms were equipped with an automatic video recording system. Video inclusion required informed consent. Video recordings were included over two separate time periods: prior to the introduction of VAD – the baseline period, and after VAD had been established as standard operating procedure – the intervention period. Videos included during the intervention period were used for VAD. All videos were used for analysis of team performance. Primary outcome: Clinical performance assessed by the “Team Obstetric Postpartum Hemorrhage” checklist. Secondary outcomes: Non‐technical performance assessed by the “Assessment of Obstetric Team Performance” checklist score and total blood loss (mL).

**Results:** We included 226 videos before implementation of VAD and 206 after implementation of VAD. Of these, 93 videos were used in a subsequent VAD. Analysis will be conducted during the spring of 2023.

**Conclusions:** This project provides a novel opportunity to learn from clinical cases and to improve the overall quality of care during emergency treatment.

## Incidence of shoulder dystocia and risk factors for recurrence in the subsequent pregnancy

### **Marie Larsen** ^1^, Maria Jeppegaard^2,3^, Amalie Thams^1^, Amalie Schmidt^3^, Steen Rasmussen^2,3^, Lone Krebs^2,3^

#### ^1^Department of Clinical Medicine, University of Copenhagen, Copenhagen, Denmark; ^2^Department of Gynecology and Obstetrics, University of Copenhagen, Holbæk Hospital, Holbæk, Denmark; ^3^Department of Gynecology and Obstetrics, University of Copenhagen, Amager Hvidovre Hospital, Hvidovre, Denmark

**Introduction/Purpose:** To estimate the incidence and risk factors for recurrence of shoulder dystocia and to identify women at high risk of recurrence in a subsequent vaginal delivery.

**Methods:** National register‐based study including data from the Danish Medical Birth Registry and National Patient Register 2007–2017. Nulliparous women with a singleton fetus, in cephalic presentation alive at labor onset were selected for analysis of risk factors in primary and subsequent delivery.

**Results:** 6002 cases of shoulder dystocia were reported with an overall incidence among women with vaginal delivery of 1.2%. Among 222 225 nulliparous women with vaginal births, shoulder dystocia complicated 2209 (1.0%) births. A second birth was registered in 1106 (50.07%) of women with shoulder dystocia in their first delivery, of which 837 (77.8%) delivered vaginally. Recurrence of shoulder dystocia was reported in 60 (7.2%) with a sixfold increased risk compared with women without a prior history of shoulder dystocia (odds ratio [OR] 6.07, 95% confidence interval [CI] 4.64–7.93; adjusted OR [aOR] 3.72, 95% CI 2.77–5.81). Significant risk factors from first delivery for recurrence of shoulder dystocia were low maternal height and obesity. Significant risk factors in the second delivery were low maternal height, birthweight >4000 g, an increase of birthweight >250 g from first to second delivery, and operative vaginal delivery.

**Conclusions:** The incidence of shoulder dystocia among nulliparous women with vaginal delivery is 1.0% with a 7% risk of recurrence in a subsequent vaginal delivery. Important risk factors for recurrence are low maternal height, high fetal weight and operative vaginal delivery.

## Intimate partner violence among pregnant women in Denmark from 2019 to 2021 – a hospital‐based cross‐sectional study

### **Hanne Hegaard** ^1,2,3^, Heidi Sharif^1^, Lea B. S. Ankerstjerne^4,5^, Seda Serhatlioglu^2,6^, Anne‐Mette Schroll^7^, Julie Midtgaard^3,8^, Kristina M. Renault^1,3^, Lotte Broberg^1,9^

#### ^1^Department of Obstetrics, Copenhagen University Hospital ‐ Rigshospitalet, Copenhagen, Denmark; ^2^The Interdisciplinary Research Unit of Women's, Children's and Families’ Health, The Juliane Marie Center, Copenhagen University Hospital ‐ Rigshospitalet, Copenhagen, Denmark; ^3^University of Copenhagen, Faculty of Health and Medical Sciences, Department of Clinical Medicine, Denmark, Copenhagen, Denmark; ^4^Department of Clinical Research, University of Southern Denmark, Odense, Denmark; ^5^Department of Gynecology and Obstetrics, Odense University Hospital, Odense, Denmark; ^6^Antalya Bilim University, Faculty of Health Sciences, Department of Midwifery, No: 290 A Dösemealti, Antalya, Turkey; ^7^The Danish Association of Midwives, Copenhagen, Denmark; ^8^Center for Applied Research in Mental Health Care (CARMEN), Mental Health Center Glostrup, Glostrup, Denmark; ^9^Center for Clinical Research and Prevention, Frederiksberg Hospital, Frederiksberg, Denmark

**Introduction/Purpose:** Violence against women, including intimate partner violence (IPV), represents a significant global public health problem. The prevalence of IPV (physical, emotional and/or sexual) during pregnancy or the year preceding pregnancy is 25% worldwide and 5% in Europe.

IPV threatens the health of the pregnant woman and her unborn child. It is associated with an increased risk of miscarriage, preterm delivery, perinatal mortality, postpartum depression and longtime adverse effects on offspring development. Pregnancy is recommended as a unique opportunity to screen and identify women experiencing IPV, partly because the longitudinal process in antenatal care provides an opportunity to develop trust between women/families and the healthcare providers. In Denmark, screening for IPV has been routinely carried out at the Department of Obstetrics, Copenhagen University Hospital ‐ Rigshospitalet.

This study aims to assess, among early pregnant women in Denmark, the prevalence of lifetime physical IPV, recent emotional IPV, and related maternal characteristics and health.

**Methods:** We performed a hospital‐based cross‐sectional study. Data were sourced from the Copenhagen Pregnancy Cohort (*n* = 7361), collected from pregnant women attending antenatal care at Copenhagen University Hospital—Rigshospitalet from 2019 to 2021.

**Results:** The prevalence of lifetime physical and recent emotional IPV was 4.0% and 0.9%, respectively. For both lifetime physical and recent emotional IPV, the highest prevalence rate was seen among women aged 18–24, non‐cohabiting, smokers, lwith ow level of educational attainment, and women with chronic medical and psychiatric disease and lower psychological well‐being.

**Conclusions:** Identification and provision of specialized care to pregnant women who have experienced IPV appear warranted.

## Labor duration and risk of adverse neonatal outcome

### Louise Lundborg^1^, Katarina Åberg^1^, Anna Sandström^1,2^, Xingrong Liu^1^, Ellen Tilden^3,4^, Jenny Bolk^1,5,6^, Linnea V. Ladfors^1^, Olof Stephansson^1,2^, **Mia Ahlberg** ^1^

#### ^1^Clinical Epidemiology Division, Department of Medicine, Solna, Karolinska Institutet, Stockholm, Sweden; ^2^Department of Women's Health, Division of Obstetrics and Gynecology, Karolinska University Hospital, Stockholm, Sweden; ^3^Department of Nurse‐Midwifery, Oregon Health & Science University School of Nursing, Portland, OR, USA ^4^Department of Obstetrics and Gynecology, Oregon Health & Science University School of Medicine, Portland, OR, USA; ^5^Department of Clinical Science and Education Södersjukhuset, Karolinska Institutet, Stockholm, Sweden; ^6^Sachs’ Children and Youth Hospital, South General Hospital, Stockholm, Sweden

**Introduction/Purpose:** Over the last decades, efforts have been made to redefine normal labor duration. Recent research concludes that first‐stage duration can last longer than previously understood without evidence of maternal/child risk.

**Methods:** Population‐based cohort study. We included 46 040 women allocated to Robson group 1 using the obstetric database from the Stockholm‐Gotland region in Sweden 2008–2020. Modified Poisson regression was applied to investigate the association between increasing active first stage of labor duration and risk of two composite adverse neonatal outcomes.

**Results:** The absolute risk for a severe outcome was 2.1% and absolute risk for a moderate outcome was 3.7% in this study population. For severe neonatal outcomes significantly, the relative risk increased ≥10.1 hours (adjusted RR [aRR] 1.53, 95% CI 1.26–1.87). For moderate neonatal outcomes the relative risk significantly increased ≥5.1 hours, (aRR 1.40, 95% CI 1.24–1.58). Mediation analysis showed that less than 20% of the risk was mediated through second stage duration.

**Conclusions:** We did not observe a clear labor duration risk threshold, rather the risk is cumulatively increased with longer active phase of labor duration from 5–10 hours for moderate neonatal outcomes, with a twofold observed increased risk for labors beyond 10 hours. A less pronounced pattern of increasing risk is also found among the rare cases of severe outcomes. The findings indicate the importance of widening our focus beyond labor duration per se and considering combinations of factors, such as medical interventions and anthropometrics, that could increase or decrease fetal tolerance for labor.

## LIPO TEEN (the lifestyle in pregnancy and offspring teenagers): identification of modifiable antenatal risk factors for childhood obesity – a protocol

### Birgitte Møller Luef^1,2^, Nanna Husted Jensen^3^, Sine Knorr Johnsen^4^, Kurt Kristensen^4^, Martin Overgaard^2,5^, Helle Terkildsen Maindal^3^, Dorte Møller Jensen^1,2,6^, **Christina Anne Vinter** ^1,2,6^

#### ^1^Department of Gynecology and Obstetrics, Odense University Hospital, Odense, Denmark; ^2^Department of Clinical Research, University of Southern Denmark, Odense, Denmark; ^3^Department of Public Health, Aarhus University, Aarhus, Denmark; ^4^Steno Diabetes Center Aarhus, Aarhus, Denmark; ^5^Department of Clinical Biochemistry, Odense University Hospital, Odense, Denmark; ^6^Steno Diabetes Center Odense, Odense University Hospital, Odense, Denmark

**Introduction/Purpose:** Aims: To evaluate the effect of lifestyle intervention during pregnancy on offspring body composition measured by DEXA scanning in 14‐year‐old offspring and to explore the effect of lifestyle intervention during pregnancy on dysmetabolic traits in mothers and offspring, by investigating the following:

- Association between lifestyle, metabolic and inflammatory markers in pregnancy, and dysmetabolic traits in mothers and teenagers 14 years after pregnancy
- Associations between maternal mental health during pregnancy and postpartum and the long‐term risk of maternal and offspring obesity
- Mental health and health literacy in mother and offspring 14 years after pregnancy and the risk of obesity

**Methods:** Follow‐up of a RCT. In the original trial, 360 pregnant women with BMI ≥30 kg/m^2^ from Odense University Hospital and Aarhus University Hospital were randomized to intervention with diet counseling and physical activity from gestational age 12 weeks and until delivery – or to a standard care control group.

The women and their offspring will be invited to a 2‐hour clinical examination including fasting venous blood samples, DEXA scanning, anthropometric measures, questionnaires on diet, physical activity, mental health and health literacy. The examinations include continuous glucose monitoring and activity tracking for 7–10 days.

**Results:** Recruiting participants from March 2023.

**Conclusions:** The study investigates long‐time effects of prenatal lifestyle intervention in teenage offspring. Our unique data addresses important knowledge gaps to come closer to an understanding of childhood obesity. If we can show an effect of intervention in pregnancy on the risk of offspring obesity, there will be a rationale for implementation in clinical practice.

## Long‐term neurodevelopmental outcome in children born after vacuum‐assisted delivery compared to second stage caesarean delivery and spontaneous vaginal delivery

### **Stefhaine Romero** ^1,2^, Katarina Lindström^3^, Johanna Listermar^4^, Magnus Westgren^1^, Gunilla Ajne^1,2^

#### ^1^Division of Obstetrics and Gynecology, Department of Clinical Science, Intervention and Technology, Karolinska Institutet, Stockholm, Sweden; ^2^Department of Pregnancy Care and Childbirth, Karolinska University Hospital, Stockholm, Sweden; ^3^Division of Pediatrics, Department of Clinical Science, Intervention and Technology, Karolinska Institutet, Stockholm, Sweden; ^4^Karolinska Institutet, Stockholm, Sweden

**Introduction/Purpose:** To evaluate long–term neurodevelopment in children born after low‐ or mid‐station vacuum–assisted delivery (VAD).

**Methods:** Prospective observational cohort study at Karolinska University Hospital, Sweden, including 440 children born by low‐ or mid‐station VAD, 844 children born after a spontaneous vaginal delivery (SVD) and 171 children born via an emergency cesarean delivery (ECD) during prolonged second stage. The Five‐to‐Fifteen questionnaire was used as a validated screening method for neurodevelopmental difficulties. In addition, registered neurodevelopmental ICD‐10 diagnoses were collected. Regression analyses compared the results for each delivery mode.

**Results:** Children born after VAD exhibited an increased rate of long‐term neurodevelopmental difficulties in motor skills (odds ratio [OR] 2.2, 95% confidence interval [CI] 1.3–3.8) and perception (OR 1.7, 95% CI 1.002–2.9) compared with children born after SVD. Similar findings were seen in the group delivered with an ECD compared with SVD (motor skills: OR 3.3, 95% CI 1.8–6.4 and perception: OR 2.3, 95% CI 1.2–4.4). A multivariable regression analysis showed an increased odds of problems with motor skills when delivered with VAD (OR 2.2, 95% CI 1.1–4.7) or ECD (OR 3.3, 95% CI 1.4–7.9) compared with SVD. There were significantly more children in the VAD group with neurodevelopmental ICD–10 diagnoses such as attention deficit/hyperactivity disorder.

**Conclusions:** The significant differences in long–term neurodevelopmental difficulties in children delivered with a VAD or ECD compared with children delivered with a SVD in the studied cohort is worrying. Further research is needed on the management of obstructed labor, including VADs and ECDs at the second stage, followed up by validated methods.

## Maternal origin matters – country of birth as a risk factor for obstetric anal sphincter injuries: a national cohort study

### **Kristin André** ^1,2^, Andrea Stuart^1,2^, Karin Källén^1,3^

#### ^1^Institution of Clinical Sciences, Lund University, Lund, Sweden; ^2^Department of Obstetrics and Gynecology, Helsingborg Central Hospital, Helsingborg, Sweden; ^3^Center for Reproductive Epidemiology, Tornblad Institute, Lund University, Lund, Sweden

**Introduction/Purpose:** Obstetric anal sphincter injuries (OASIS) are severe complications to vaginal births with potential long‐term consequences. It is uncertain what part maternal origin plays in the overall risk. The study objective was therefore to assess the association between maternal country of birth and OASIS.

**Methods:** A Swedish nationwide cohort study including singleton term vaginal births 2005–2016. Data were extracted from the Swedish Medical Birth Registry and Statistics Sweden. Modified Poisson regression analyses were performed to obtain crude and adjusted risk ratios (aRR). Adjustments were made in four cumulative steps. Sub‐analyses were performed to investigate the risk of OASIS associated with female genital mutilation.

**Results:** 988 804 births were included. The rate of OASIS in Swedish‐born women was 3.5%. Women from South/Eastern had an increased risk of OASIS (aRR 1.71, 95% confidence interval [CI] 1.60–1.83), as did women born in Sub‐Saharan Africa (aRR 1.60, 95% CI 1.49–1.72). Conversely, women from South/ Central America had a decreased risk of OASIS (aRR 0.65, 95% CI 0.56–0.76). Female genital mutilation was associated with an increased risk of OASIS (aRR 3.05, 95% CI 2.60–3.58). Episiotomy appeared to be overall protective (aRR 0.95, 95% CI 0.92–0.98) but was not significantly more protective among women with female genital mutilation.

**Conclusions:** Country of birth plays an important role in the risk of OASIS. Women from Foreign Asia and Sub‐Saharan Africa are at significantly increased risk as compared with Swedish‐born women, whereas women from South/Central America are at lower risk. Female genital mutilation is also a significant risk factor for OASIS.

## Maternal thyroid function as predictor for markers of insulin resistance in pregnant women with polycystic ovary syndrome

### **Anastasia Trouva** ^1,2^, Eszter Vanky^3,4^, Michael Alvarsson^1^, Jan Calissendorff^1^, Bjørn Olav Åsvold^5,6,7^, Dorina Ujvari^8,9^, Angelica Lindén Hirschberg^8,10^

#### ^1^Department of Molecular Medicine and Surgery, Karolinska Institutet, Stockholm, Sweden; ^2^Department of Internal Medicine, Section of Diabetes and Endocrinology, Södersjukhuset, Stockholm, Sweden; ^3^Department of Clinical and Molecular Medicine, Faculty of Medicine and Health Sciences, Norwegian University of Science and Technology, Trondheim, Norway; ^4^Department of Obstetrics and Gynecology, St. Olav's Hospital, Trondheim University Hospital, Trondheim, Norway; ^5^K.G. Jebsen Center for Genetic Epidemiology, Department of Public Health and Nursing, NTNU, Norwegian University of Science and Technology, Trondheim, Norway; ^6^ HUNT Research Center, Department of Public Health and Nursing, NTNU, Norwegian University of Science and Technology, Levanger, Norway; ^7^Department of Endocrinology, Clinic of Medicine, St. Olav's Hospital, Trondheim University Hospital, Trondheim, Norway; ^8^Department of Women's and Children's Health, Karolinska Institutet, Stockholm, Sweden; ^9^Department of Microbiology, Tumor and Cell Biology, National Pandemic Center, Center for Translational Microbiome Research, Karolinska Institutet, Solna, Sweden; ^10^Department of Gynecology and Reproductive Medicine, Karolinska University Hospital, Stockholm, Sweden

**Introduction/Purpose:** Low maternal free thyroxine (fT4) levels have been associated with adverse metabolic phenotype and gestational diabetes. Our aim was to investigate whether maternal thyroid function could predict insulin resistance in polycystic ovary syndrome (PCOS) pregnancies.

**Methods:** Post‐hoc analyses of two randomized controlled trials. Women with PCOS were randomized to metformin or placebo in early pregnancy. Serum thyroid stimulating hormone (TSH) and fT4 were measured at gestational weeks (gw) 5–12, 19, 32 and 36 in 309 women. We analyzed fasting glucose and insulin at gw 19, 32 and 36 and calculated HOMA‐IR. We used linear regression to analyze statistical associations.

**Results:** There were no associations between TSH or fT4 and glucose levels later on in pregnancy. However, baseline fT4 was inversely associated with insulin levels at gw 19 (*b* = −1.40, *P* < 0.001), gw 32 (*b* = −1.26, *P* < 0.01) and gw 36 (*b* = −1.66, *P* < 0.01). Furthermore, fT4 at gw 19 was inversely associated with insulin levels at gw 32 (*b* = −1.02, *P* < 0.05) and gw36 (*b* = −1.54, *P* < 0.05), and fT4 at gw 32 was inversely associated with insulin levels at gw 36 (*b* = −1.88, *P* < 0.05). The change in TSH between baseline and gw 19 (increase) correlated negatively to insulin levels in gw 32 (*b* = −2.20, *P* < 0.01). Similar statistically significant correlations of fT4 and change in TSH were also observed with HOMA‐IR.

**Conclusions:** Low maternal fT4 levels and smaller TSH increase in the first and second trimester are associated with higher insulin levels and may predict insulin resistance later in pregnancy in women with PCOS.

## Midwifery continuity of care during pregnancy, birth and the postpartum period: a matched cohort study

### **Mia Ahlberg** ^1,2^, Katarina Åberg^1^, Louise Lundborg^1^, Mikael Norman^3,4,5^, Olof Stephansson^1,2^, Karin Pettersson^2^, Marie Ekborn^2^, Sven Cnattingius^1^

#### ^1^Clinical Epidemiology Division, Department of Medicine, Solna, Karolinska Institutet, Stockholm, Sweden; ^2^Department of Women's Health, Karolinska University Hospital, Stockholm, Sweden; ^3^Department of Clinical Science, Intervention, and Technology, Karolinska Institutet, Stockholm, Sweden; ^4^Department of Neonatal Medicine, Karolinska University Hospital, Stockholm, Sweden; ^5^The Swedish Neonatal Quality Register, Stockholm, Sweden

**Introduction/Purpose:** Midwifery Continuity of Care (MCoC) models have been proved safe and beneficial for mothers and newborns mainly in low‐risk pregnancies. This matched cohort study aimed to investigate labor outcomes in a MCoC model targeting women with fear of birth, and compare the outcomes with standard midwifery care.

**Methods:** Data from the Swedish Pregnancy Register was used to compare MCoC to standard care. Women giving birth at Karolinska University Hospital at Huddinge in Stockholm between January 1, 2019 to August 31, 2021 were included in the study population. Propensity score matching was applied to obtain a matched set from the standard care group for every woman in the MCoC model. Based on the matched cohort, we estimated risk ratios (RR) for binary outcomes with 95% confidence intervals (CI). The main outcome measures were: Interventions during labor, mode of birth, and preterm birth (<37 gestational weeks).

**Results:** Compared with standard care, women in the MCoC model were more likely to give birth spontaneously (relative risk [RR] 1.06, 95% CI 1.02–1.10) and less likely to have an elective cesarean section on maternal request (RR 0.24, 95% CI 0.11–0.51). The risk of preterm birth was reduced in the MCoC group (RR 0.51, 95% CI 0.32–0.82).

**Conclusions:** The MCoC model was associated with fewer medical interventions and improved maternal and neonatal outcomes.

## New insights on maternal platelet alloimmunization and placenta function

### **Nora Hersoug Nedberg** ^1,2^, Mona Nystad^2,3^, Gitta Turowski^4,5^, Borghild Roald^4,5^, Katarzyna Guz^6^, Małgorzata Uhrynowska^6^, Eirin Listau Bertelsen^1^, Maria Therese Ahlen^1,7^, Anne Husebekk^1^, Anne Cathrine Staff^5,8^, Ewa Brojer^6^, Heidi Tiller^2,3^

#### ^1^Immunology Research Group, Department of Medical Biology, Faculty of Health Sciences, UiT The Arctic University of Norway, Tromsø, Norway; ^2^Women's Health and Perinatology Research Group, Department of Clinical Medicine, Faculty of Health Sciences, UiT The Arctic University of Norway, Tromsø, Norway; ^3^Department of Obstetrics and Gynecology, University Hospital of North Norway, Tromsø, Norway; ^4^Department of Pathology, Center for Perinatal and Pregnancy‐Related Pathology, Oslo University Hospital, Oslo, Norway; ^5^Institute of Clinical Medicine, Faculty of Medicine, University of Oslo, Oslo, Norway; ^6^Department of Hematological and Transfusion Immunology, Institute of Hematology and Transfusion Medicine, Warsaw, Poland; ^7^Norwegian National Unit for Platelet Immunology, Department of Laboratory Medicine, University Hospital of North Norway, Tromsø, Norway; ^8^Division of Obstetrics and Gynecology, Oslo University Hospital Ullevål, Oslo, Norway

**Introduction/Purpose:** Fetal and neonatal alloimmune thrombocytopenia (FNAIT) is defined as the destruction of fetal platelets due to incompatibility in human platelet antigens (HPA) between mother and fetus. In white Europeans, antibodies against HPA‐1a are responsible for most FNAIT‐cases. Maternal anti‐HPA‐1a antibodies can bind epitopes on trophoblast cells and are suggested to affect trophoblast function. An association with lower birthweight has been reported. The aim of this project was to investigate whether maternal anti‐platelet antibodies during pregnancy associates with altered placenta‐associated clinical outcomes and biomarkers.

**Methods:** Term placentas from 27 HPA‐1a alloimmunized women were compared with 21 placentas from non‐immunized HPA‐1a‐negative controls, collected in Poland and Norway. Maternal plasma samples and maternal/neonatal clinical data were obtained. Hematoxylin–eosin‐stained placenta sections were analyzed for chronic inflammation. Circulating maternal placenta‐associated biomarkers (sFlt‐1, PlGF and Endoglin) were measured in maternal plasma from the second and third trimesters using enzyme‐linked immunoassays (R&D Systems).

**Results:** Chronic inflammation was observed in 74% of placentas from HPA‐1a alloimmunized pregnancies, compared with 38% among controls (*P* = 0.02). The immunized pregnancies had significantly higher plasma Endoglin concentrations during the second trimester than controls (*P* = 0.007, Mann–Whitney *U*‐test). Alloimmunized pregnancies with chronic placental inflammation had a lower sFlt‐1/PlGF ratio during the third trimester than those without (*P* = 0.03, Mann–Whitney *U*‐test).

**Conclusions:** Pregnancies with maternal anti‐platelet antibodies more often had histologic evidence of chronic placental inflammation compared with controls. Our finding of altered circulating angiogenic protein biomarker patterns in immunized pregnancies warrants further research to elucidate their mechanistic and clinical implications.

## Next‐generation effects of vitamin D supplementation in pregnancy

### **Nanna S. Svensson** ^1,2,3^, Anna L. Vestergaard^1,2,4^, Esben T. Vestergaard^3,4^, Agnete Larsen^2^, Pinar Bor^1,4^

#### ^1^Department of Obstetrics and Gynecology, Regional Hospital Randers, Denmark; ^2^Department of Biomedicine, Aarhus University, Denmark; ^3^Department of Pediatrics, Regional Hospital Randers, Randers, Denmark; ^4^Department of Clinical Medicine, Aarhus University, Aarhus, Denmark

**Introduction/Purpose:** The majority of pregnant women in Denmark are supplemented by 10 μg vitamin D daily, but vitamin D deficiency is nonetheless common. Maternal vitamin D deficiency has adverse effects on children's health, including increased risks of obesity, asthma and multiple sclerosis. This study aims to investigate whether a maternal vitamin D supplement of 90 vs 10 μg/day in pregnancy improves the overall health, growth and immune function of children during their first year of life.

**Methods:** Children from pregnant women in an ongoing randomized controlled trial (90 vs 10 μg/day vitamin D) were invited to this follow‐up study. The immune cell reactivity from umbilical cord blood and from a blood sample at 11–13 months of age were investigated. Postnatal development wasevaluated based on questionnaires. A 12‐month clinical examination was performed including measurements of weight, length, head circumference, size of the anterior fontanel and growth rate. Further ASQ‐3 was used to evaluate the cognitive development of the children.

**Results:** The primary outcomes are growth curves and immune cell function. The secondary outcome is the infection rate of the children. The study is ongoing.

**Conclusions:** The results of this study will provide new knowledge on the effects of high intrauterine vitamin D exposure on growth, immune system and overall health of children during their first year of life. This knowledge can help guide health authorities when evaluating the need for new guidelines on an optimal vitamin D intake in pregnancy.

## Nordic randomized trial on laparoscopic vs vaginal cerclage (NORACT)

### **Lea Kirstine Hansen** ^1^, Kirsten Hald^2^, Oskari Heikinheimo^3^, Hulda Hjartardóttir^4^, Bo Jacobsson^5^, Pernille Tine Jensen^1^, Helena Karypidis^6^, Lise Qvirin Krogh^1^, Annie Lantto^7^, Andrew Shennan^8^, Niels Uldbjerg^1^, Julie Glavind & the NORACT group^9^

#### ^1^Department of Clinical Medicine & Department Obstetrics and Gynecology, Aarhus University Hospital, Aarhus, Denmark; ^2^Gynecological Department, Division of Obstetrics and Gynecology, Oslo University Hospital, Oslo, Norway; ^3^Department of Obstetrics & Gynecology, University of Helsinki and Helsinki University Hospital, Helsinki, Finland; ^4^Obstetrics and Prenatal Diagnosis Unit, Landspitali University Hospital, Reykjavík, Iceland; ^5^Department Obstetrics and Gynecology, Sahlgrenska University Hospital/Østra, Göteborg, Sweden; ^6^Department of Women's and Children's Health, Uppsala University Hospital, Uppsala, Sweden; ^7^Department of Obstetrics and Gynecology, Lund University Hospital, Lund, Sweden; ^8^Department of Women and Children's Health, School of Life Course Sciences, FoLSM, Kings College London, UK; ^9^ NORACT study sites

**Introduction/Purpose:** A vaginal cerclage as well as an abdominal cerclage can prevent preterm birth (PTB) due to cervical incompetence. There is uncertainty about the patients in whom an abdominal cerclage should be preferred to a vaginal cerclage.

The overall objective for this study is to compare vaginal cerclage and laparoscopic cerclage in women at moderate to high risk of PTB due to cervical incompetence.

**Methods:** NORACT is an open, multicenter, randomized controlled trial (RCT) with an embedded 18‐month internal pilot trial to evaluate feasibility, acceptability, safety and surgical proficiency. The study extends from sites in Denmark, Sweden, Norway, Finland, Iceland and UK.

Participants will be randomized to vaginal cerclage performed during pregnancy or laparoscopic cerclage performed pre‐pregnancy or until 10+0 weeks of gestation.

Eligibility will be based on an equipoise criterion, ie we will include women in whom the clinician is equipoised as regards whether a vaginal or laparoscopic cerclage is the best treatment for the prevention of PTB. Any circumstance under which the clinician is not willing to randomize is an exclusion criterion. The primary outcome is PTB <32+0 weeks of gestation in the first subsequent pregnancy beyond 16 weeks of gestation. A total sample of 190 participants will be included.

**Results:** Inclusion for the trial will be initiated through 2023.

**Conclusions:** NORACT will be the first RCT to compare vaginal and laparoscopic cerclages and the first Nordic RCT collaboration.We encourage all NFOG members to refer patients at high risk of extreme PTB to their national NORACT study centers.

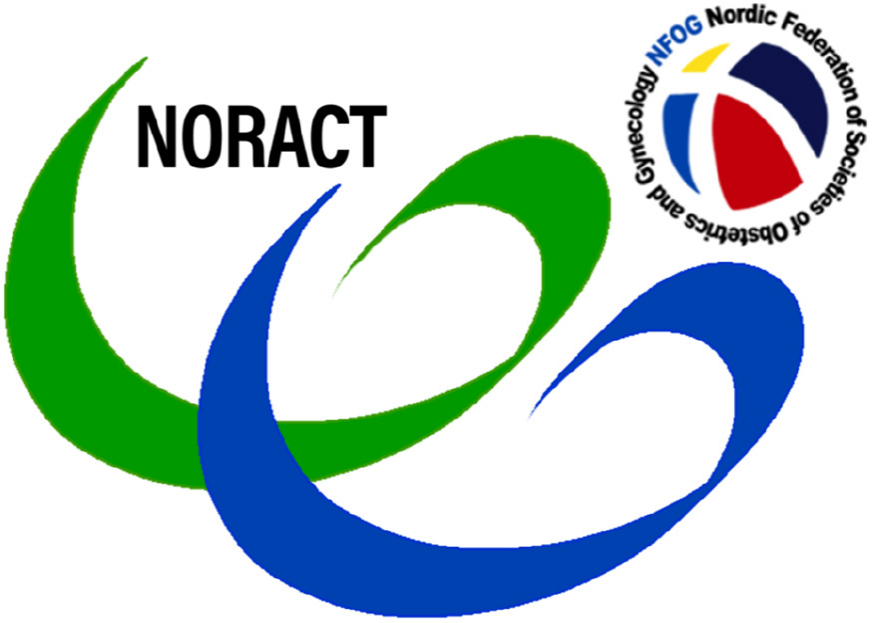

## Objectively measured physical activity during pregnancy is associated with maternal lipid profile, but not with HbA1c: data from the FITMUM RCT

### **Ida Karoline Bach Jensen** ^1^, Caroline Borup Roland^1,2^, Signe de Place Knudsen^1,2^, Anne Dsane Jessen^1,2^, Saud Alomairah^2,3^, Ole Hartvig Mortensen^2^, Lennart Friis‐Hansen^4^ , Jane Bendix^1,5^, Stig Molsted^5,6^, Bente Stallknecht^2^, Tine Dalsgaard Clausen^1,6^, Ellen Løkkegaard^1,6^

#### ^1^Department of Gynecology and Obstetrics, Copenhagen University Hospital ‐ North Zealand, Copenhagen, Denmark; ^2^Department of Biomedical Sciences, University of Copenhagen, Copenhagen Denmark; ^3^College of Health Sciences, Public Health Department, Saudi Electronic University, Riyadh, Saudi Arabia; ^4^Department of Clinical Biochemistry, Copenhagen University Hospital – Bispebjerg and Frederiksberg, Denmark; ^5^Department of Clinical Research, Copenhagen University Hospital – North Zealand, Copenhagen, Denmark; ^6^Department of Clinical Medicine, University of Copenhagen, Copenhagen, Denmark

**Introduction/Purpose:** Maternal lipid and glucose concentrations affect both fetal growth and the risk of pregnancy complications. The aim of this study was to investigate associations between objectively measured physical activity (PA), maternal blood lipid and HbA1c concentrations.

**Methods:** Healthy pregnant women (*n* = 219) were enrolled in the study at gestational week 6+4 to 15+0 and randomized to either PA interventions or a control group. PA was measured continuously with a wrist‐worn activity tracker from inclusion throughout pregnancy. PA measures included average daily burned active kilocalories and weekly minutes of moderate‐to‐vigorous intensity PA (MVPA). Venous blood samples were collected at 28+0–6 weeks, 34+0–6 weeks and at delivery. Total cholesterol, LDL‐C, HDL‐C, triglyceride and HbA1c concentrations were measured by standard biochemical methods. Associations between average cross‐sectional PA measures and maternal blood lipid and HbA1c concentrations were analyzed using linear regression analyses.

**Results:** Burned active kilocalories were inversely associated with total cholesterol concentrations (mmol/L) in week 28 (slope −0.001, *P* = 0.027) and week 34 (slope −0.0009, *P* = 0.009). Burned active kilocalories were inversely associated with HDL‐C concentrations (mmol/L) in week 28 (slope −0.0006, *P* < 0.001), week 34 (slope −0.0005, *P* < 0.001) and at delivery (slope −0.0005, *P* < 0.001). MVPA was inversely associated with total cholesterol (mmol/L) in week 34 (slope −0.020, *P* = 0.032) and with HDL‐C (mmol/L) in week 28 (slope −0.011, *P* = 0.006), week 34 (slope −0.007, *P* = 0.005) and at delivery (slope −0.008, *P* = 0.012). No other significant associations were detected.

**Conclusions:** In conclusion, higher PA during pregnancy was associated with lower maternal total cholesterol and HDL‐C concentrations, but not with lower LDL‐C, triglyceride or HbA1c concentrations.

## Oral *Lactobacillus rhamnosus* exposure during pregnancy and effects on maternal inflammatory response

### Mahsa Nordqvist

#### Obstetrics och Gynecology, Akleja Kvinnoklinik, Borås, Sweden

**Introduction/Purpose:** The aim of the study was to analyze whether intake of *Lactobacillus rhamnosus* (*LGG*) throughout pregnancy affects maternal systemic inflammation and inflammatory response, especially in women with a history of spontaneous preterm delivery (PTD) or preeclampsia.

**Methods:** A double‐blinded, randomized, placebo‐controlled study was performed in western Sweden between 2012 and 2020. In all, 120 pregnant women were included in three different subgroups: history of spontaneous PTD, history of preeclampsia and nulliparas or parous women without a history of preeclampsia or spontaneous PTD. The women were randomized to oral intake of capsules containing *LGG* or placebo. Whole blood was sampled at inclusion before gestational week 18, and around weeks 25 and 35. Respective changes in tumor necrosis factor alpha (TNF‐α) levels after stimulation with LPS from *Escherichia coli*, between baseline and visit two and three, were analyzed as primary outcomes. Secondary outcomes were respective changes in TNF‐α, interleukin (IL)‐10 and IL‐12 levels after stimulation with LPS, *Lactobacillus paracasei* and *Pseudomonas aeruginosa*, and levels of lymphocyte subpopulations between baseline and visits two and three, and visits two to three.

**Results:** No significant results were found in the primary outcomes. In the secondary outcomes, lower levels of total lymphocytes and T‐cells and generally lower IL‐10 and IL‐12 levels were found at visit two and higher IL‐10 and IL‐12 levels at visit three in the intervention group.

**Conclusions:** Intake of *LGG* may impact the immunological response in pregnant women, with decreased inflammatory cytokine levels in mid‐pregnancy and increased levels in late pregnancy.

## Paracetamol use prior to and in early pregnancy – prevalence and patterns among women with and without chronic medical diseases

### Mille Taagaard^1,2^, Line Rode^2,3^, Mie Gaarskjær de Wolff^4^, Peter Damm^1,5^, Casper P. Hagen^6,7^, Margit Bistrup Fischer^6,7^, Hanne Kristine Hegaard^1,5,8^, **Ane Lilleøre Rom** ^1,8,9^

#### ^1^Department of Obstetrics, The Juliane Marie Center, Copenhagen University Hospital, Rigshospitalet, Copenhagen, Denmark; ^2^Department of Clinical Biochemistry, Copenhagen University Hospital, Rigshospitalet, Copenhagen, Denmark; ^3^Department of Obstetrics, Center for Fetal Medicine, Copenhagen University Hospital, Rigshospitalet, Copenhagen, Denmark; ^4^Department of Gynecology and Obstetrics, Copenhagen University Hospital, Amager – Hvidovre, Denmark; ^5^Department of Clinical Medicine, University of Copenhagen, Copenhagen, Denmark; ^6^Department of Growth and Reproduction, Rigshospitalet, University of Copenhagen, Copenhagen, Denmark; ^7^International Center for Research and Research Training in Endocrine Disruption of Male Reproduction and Child Health (EDMaRC), Rigshospitalet, University of Copenhagen, Copenhagen, Denmark; ^8^The Interdisciplinary Research Unit of Women's, Children's and Families’ Health, The Juliane Marie Center, Copenhagen University Hospital Rigshospitalet, Copenhagen, Denmark; ^9^Research Unit of Gynecology and Obstetrics, Department of Clinical Research, University of Southern Denmark, Odense, Denmark

**Introduction/Purpose:** Paracetamol is commonly consumed by pregnant women, even though recent data has questioned the safety of use. Having chronic medical diseases (CMDs) may influence the prevalence of use. We aimed to assess the prevalence and patterns of use 3 months prior to pregnancy and in the first trimester among women with and without CMDs.

**Methods:** We used patient‐reported data from the Copenhagen Pregnancy Cohort from October 1, 2013 to May 23, 2019, with information on CMDs (various types) and paracetamol use. Prevalence and patterns of use were assessed descriptively and by multivariable logistic regression models.

**Results:** We included 24 019 pregnancies. Use of paracetamol prior to and in early pregnancy was significantly higher among women with CMDs compared with women without CMDs (40.7% vs 35.8% vs 9.1% vs 5.1%, respectively). Women with CMDs were 2.7 times more likely to have a frequent intake (daily or 1–2 times per week) compared with women without CMDs (adjusted odds ratio [aOR] 2.69, 95% confidence interval [CI] 2.05–3.32)). Migraine, rheumatoid arthritis and mental disease were specifically associated with a higher likelihood of frequent use of paracetamol (aOR 4.39 [95% CI 3.20–6.02), aOR 4.32 [95% CI 2.41–7.72] and aOR 2.74 [95% CI] 1.67–4.49), respectively).

**Conclusions:** Women with CMDs had a higher use of paracetamol before and during pregnancy compared with women without CMDs. Women with migraine, rheumatoid arthritis and mental disease showed the highest risk of frequent use. This study highlights the importance of discussing pain relief in pregnancy and evaluating the potential influence of maternal CMDs when assessing adverse effects of paracetamol use during pregnancy.

## Pelvic floor dysfunction 8 weeks after an obstetric second‐degree perineal laceration in relation to BMI

### **Maria Otterheim** ^1^, Linda Hjertberg^1,2^, Sofia Pihl^2,3^, Eva Uustal^2,3^, Marie Blomberg^2,3^

#### ^1^Department of Obstetrics and Gynecology in Norrköping, Sweden; ^2^Department of Biomedical and Clinical Sciences, Linköping University, Linköping, Sweden; ^3^Department of Obstetrics and Gynecology in Linköping, Sweden

**Introduction/Purpose:** The impact of body mass index (BMI) on pelvic floor function after an obstetric second‐degree perineal laceration is unknown. The aim of this study was to evaluate the hypothesis that pelvic floor dysfunction from a second‐degree perineal laceration is more common in overweight and obese women 8 weeks postpartum than in normal weight women.

**Methods:** A register‐based cohort study including 10 876 primiparous women with an obstetric second‐degree perineal laceration between 2014 and 2021. Data were extracted from the Swedish Perineal Laceration Registry. Primary outcomes in relation to maternal BMI were urinary incontinence (UI) and anal incontinence (AI) 8 weeks postpartum. Logistic regression models were used for comparison between normal weight (BMI <24.9, reference), overweight (25.0–29.9) and obese (≥30) women. Adjustments were made for age, pre‐pregnancy AI, pre‐pregnancy UI (UI), duration of second stage of labor (AI), episiotomy (AI) and mode of delivery (AI).

**Results:** There was a significant increased risk for UI in both overweight [14.4%] and obese women [15.5%] compared with normal weight women [12%]: adjusted OR (aOR) 1.21, 95% confidence interval [CI] 1.02–1.44 vs aOR 1.27 95% CI 1.13–1.58. In contrast, overweight [13.6%] and obese women [11.8%] had a significantly decreased risk for AI compared with normal weight women [15.9%]: aOR 0.81, 95% CI 0.68–0.96 vs aOR 0.72, 95% CI 0.57–0.90. Absolute rates are given in square brackets.

**Conclusions:** Findings in the present study are new and merit further study to find potential preventive factors for AI associated with higher BMI as well as possible interventions to decrease the proportion of women affected by UI.

## Perinatal outcomes after therapeutic rest in the latent phase of labor: a cohort study

### Nanna Bagger^1^, Ioanna Milidou^2^, Sidsel Boie^3,4^, **Julie Glavind**

#### ^1^Department of Obstetrics and Gynecology, Aarhus University Hospital, Palle Juul‐Jensens Blvd. 99, 8200 Aarhus N, Denmark; ^2^Department of Pediatrics and adolescent medicine, Gødstrup Hospital, Herning, Denmark; ^3^Department of Obstetrics and Gynecology, Aalborg University Hospital, Aalborg, Denmark; ^4^Department of Clinical Medicine, Aarhus University Hospital, Aarhus, Denmark

**Introduction/Purpose:** Therapeutic rest refers to the usage of medication to relieve pain in women in the latent phase of labor. Very few data are available to evaluate the safety and effect of its use. We aimed to evaluate perinatal outcomes in women who were treated with therapeutic rest during the latent phase of labor.

**Methods:** Retrospective cohort study with inclusion of nulliparous singleton pregnant women in the latent phase of labor presenting at the labor ward at Aarhus University Hospital, Denmark, from May 13, 2018 to June 1, 2021. We identified two groups: women who were treated with therapeutic rest and women who were not. The primary outcomes were neonatal admission and neonatal resuscitation.

**Results:** In our sample of 800 women in the latent phase of labor, 414 women (52%) were treated with therapeutic rest and 386 women (48%) were not. The most frequently used (*n* = 206) medication for therapeutic rest was a combination of paracetamol, triazolam and codeine. We found no significant difference in neonatal admission (9.2% vs 6.5%, adjusted odds ratio [OR] 1.2, 95% confidence interval [CI] 0.4–3.2) or neonatal resuscitation (2.4% vs 3.1%, aOR 0.7, 95% CI 0.1–4.0) between women treated with or without therapeutic rest.

**Conclusions:** This study found no significant association between therapeutic rest and neonatal admission or resuscitation. Our findings indicate that therapeutic rest is a safe method for managing the latent labor phase concerning neonatal health.

## Placental leptin and leptin receptor gene expression in women with polycystic ovary syndrome treated with metformin or placebo

### **Johanna Molin** ^1^, Eva Dehlin^1^, Eszter Vanky^2,3^, Marie Bixo^1^

#### ^1^Department of Clinical Sciences, Umeå University, Umeå, Sweden; ^2^Department of Clinical and Molecular Medicine, Faculty of Medicine and Health Sciences, Norwegian University of Science and Technology, Trondheim, Norway; ^3^Department of Obstetrics and Gynecology, St. Olav's Hospital, Trondheim University Hospital, Trondheim, Norway

**Introduction/Purpose:** The placenta contributes to increased serum leptin and soluble leptin‐receptor levels during pregnancy. Metformin treatment attenuates serum leptin increase and amplifies soluble leptin‐receptor increase in pregnant women with polycystic ovary syndrome (PCOS) and is associated with reduced weight gain. Whether metformin modulates placental leptin and leptin‐receptor synthesis is unknown. We aimed to investigate placental leptin and leptin‐receptor gene expression, and to explore correlations with maternal, neonatal and placental weight indexes in PCOS.

**Methods:** We assessed leptin and leptin‐receptor mRNA levels by RT‐qPCR in placental samples obtained from 57 women with PCOS, previously randomized to metformin (PCOS‐M) or placebo (PCOS‐P) treatment during pregnancy. Outcome measures were leptin and leptin‐receptor mRNA relative fold change, maternal weight gain, offspring birthweight and placental weight.

**Results:** Leptin gene expression was 3.7 times higher in PCOS‐M than in PCOS‐P (Figure 1A). Leptin gene expression related negatively with offspring birthweight and placental weight in PCOS‐M (Figure 1D,E), and positively with maternal gestational weight gain and infant birthweight in PCOS‐P (Figure 1F,G). Leptin‐receptor gene expression was equivalent in both groups (Figure 1B), and was not correlated with any of the weight indexes. PCOS‐M had significantly lower weight gain (9.3 vs 11.5 kg, *P* = 0.039) and placental weight (598 vs 697 g, *P* = 0.012) compared with PCOS‐P. Offspring birthweight was equivalent in both groups.**Conclusions:** Metformin treatment during pregnancy appears to upregulate placental leptin gene expression and modulate correlations with maternal, neonatal and placental weight indexes in PCOS. Sample size was limited in this explorative study, which reduced power in statistical analyses. The observed trends require confirmation in larger cohorts.

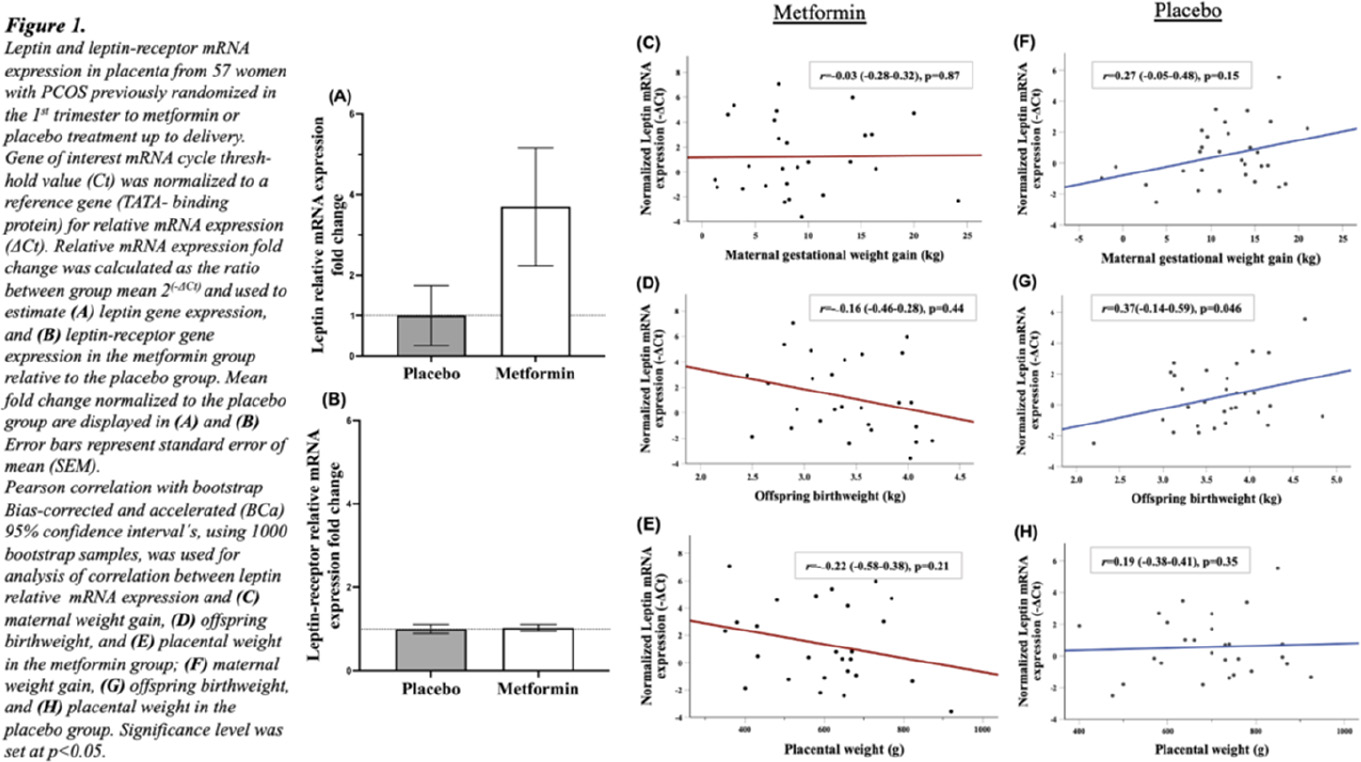

## Placental size at gestational week 37: comparison between ongoing pregnancies and deliveries

### **Helene Fjeldvik Peterson** ^1,2^, Vigdis Hillestad^2^, Silje Sommerfelt^1^, Anne Eskild^1,2^

#### ^1^Akershus University Hospital, Division of Gynecology and Obstetrics, Lørenskog, Norway; ^2^Oslo University, Faculty of Medicine, Oslo, Norway

**Introduction/Purpose:** We aimed to compare placental size and placental size relative to fetal size (ratio) in ongoing pregnancies at gestational week 37 with deliveries at gestational week 37.

**Methods:** Ongoing unselected singleton pregnancies (*n* = 89) were examined by magnetic resonance imaging (MRI) at gestational week 37 during 2017–2018, and placental and fetal volume (in cm^3^) were calculated. The placental size and ratio in ongoing pregnancies were compared with placental size (in grams) and ratio among all deliveries in Norway at gestational week 37 during 2016–2019 (*n* = 11 033).

**Results:** In ongoing pregnancies, mean placental size was 845 cm^3^ (standard deviation [SD] 183 cm^3^) and mean size of all delivered placentas was 615 g (SD 149 g). Among deliveries after spontaneous onset of labor (*n* = 5912), mean placental size was 604 g (SD 135 g). After elective cesarean delivery (*n* = 1020), mean placental size was 667 g (SD 165 g).

Mean ratio in ongoing pregnancies was 0.312 (SD 0.054) and mean ratio among all deliveries was 0.199 (SD 0.036). Among deliveries after spontaneous onset of labor, mean ratio was 0.196 (SD 0.036) and among elective cesarean deliveries mean ratio was 0.207 (SD 0.037).

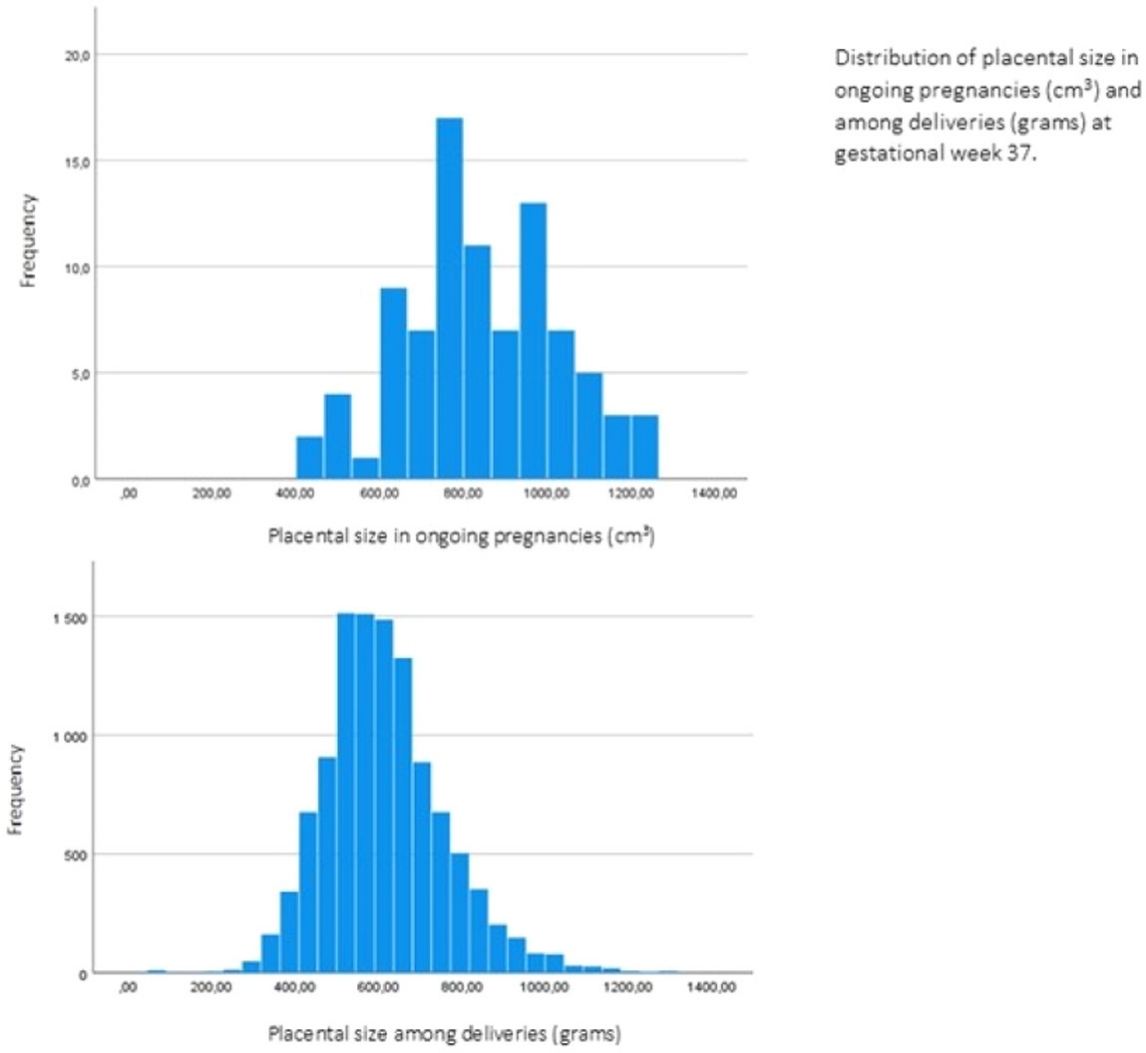

**Conclusions:** The placenta is larger in ongoing pregnancies than among deliveries. This finding suggests that placental size decreases during labor and delivery. Reference values of placental size based on delivered placentas may not be valid for ongoing pregnancies.

## Postpartum infection rates for intended vaginal delivery compared with intended cesarean delivery: a Danish national register study

### **Paul Axelsson** ^1^, Ellen Løkkegaard^2^

#### ^1^Odense University Hospital, Odense, Denmark; ^2^Nordsjællands Hospital, Hillerød, Denmark

**Introduction/Purpose:** Pregnant women who plan to give birth by cesarean delivery, are routinely informed of the risk of postpartum infections. The risk is seldom put into the perspective that intended vaginal delivery carries a risk of infection as well.

**Methods:** A Danish national registry‐based study in the 11‐year period from 2007 to 2017. Women were divided into two groups: intended vaginal delivery (iVD) or intended cesarean delivery (iCD). Outcomes were based on redeemed prescriptions for antibiotics and ICD‐10 diagnostic codes for infections up to 30 days postpartum. The study has relevant institutional approval.

**Results:** We included 647 871 women giving birth to live singletons in the study period, of which 574 471 were in the iVD group and 60 391 in the iCD group. In the iVD group 12.1% of women (*n* = 69 327) received some type of antibiotics compared with 14.4% (8678) of women in the iCD group. The difference became gradually smaller over the years between the groups, being the smallest in 2017: 12.1% and 12.6%, respectively. Surgical site infections were higher in the iCD group (0.9%, *n* = 555) than in the iVD group (0.3%, *n* = 1767) but were slightly lower for endometritis; 0.7% (*n* = 444) vs 0.8% (*n* = 4992). In logistic regression, the risk of receiving any antibiotic was higher in the iCD group (odds ratio [OR] 1.22, 95% confidence interval [CI] 1.19–1.25 vs aOR 1.21, 95% CI 1.18–1.24).

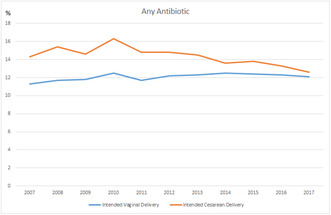

**Conclusions:** There is a slightly increased risk of infection for planned cesarean delivery compared with intended vaginal delivery. However, this difference in risk is clinically very small and appears to be decreasing with time.

## Predicting maternal morbidity in hypertension disorders in pregnancy with the shrunken pore syndrome ratio to optimize timing of delivery

### **Danielle Damm** ^1^, Anders Grubb^2^, Helena Strevens^1^

#### ^1^Department of Obstetrics and Gynecology, Lund University, Skåne University Hospital, Lund, Sweden; ^2^Department of Clinical Chemistry, Lund University, Lund, Sweden

**Introduction/Purpose:** Our aim is to evaluate a previously described marker of glomerular endothelial damage; the shrunken pore syndrome (SPS), to determine whether women with hypertensive disorders in pregnancy can be distinguished as low‐risk or high‐risk for developing severe maternal morbidity, according to this marker.

**Methods:** Women discharged from the perinatal ward at the Skane University Hospital in Lund during the period of September 1, 2016 to to August 31, 2017 under one of the diagnoses within hypertension disorders in pregnancy were considered for inclusion in the study. Records from included patients were reviewed and all registered measures of renal function were analyzed retrospectively. An SPS ratio (eg the ratio eGFR_cystatin C_/eGFR_creatinine_) of 0.60 or less was considered to define a high risk for severe adverse maternal outcome, since this ratio is associated with endothelial damage.

**Results:** Sixty‐nine women were included in the study. Fifty women were defined as high‐risk (SPS ratio ≤0.60), of which 17 (34%) developed a severe adverse maternal outcome. Of the 19 women defined as low‐risk (SPS ratio >0.60), only two (10.5%) developed severe adverse maternal outcome (*P*‐value 0.071). Among the 32 women diagnosed as “preeclampsia with severe features” we found an association between severe adverse maternal outcome and an SPS ratio ≤0.60 (*P*‐value 0.035).

**Conclusions:** The SPS ratio seems promising as a predictive marker for maternal morbidity in hypertension in pregnancy. Its performance as a tool in the monitoring of progressing disease should be evaluated further in larger cohorts. Delivery before the SPS ‐ratio decreases to or below 0.60 might avoid maternal complications.

## Predicting risk of anal sphincter injury at delivery in primi‐ and secundipara and at the first vaginal delivery after a cesarean section

### **Jennie Larsudd‐Kåverud** ^1,2^, Sigvard Åkervall^1^, Mattias Molin^3^, Ida E.K. Nilsson^1,2^, Ian Milsom^1,4^, Maria Gyhagen^1,2^

#### ^1^Gothenburg Continence Research Center, Institute of Clinical Sciences, Sahlgrenska Academy at Gothenburg University, Gothenburg, Sweden; ^2^Department of Obstetrics and Gynecology, Södra Älvsborgs Hospital, Borås, Sweden; ^3^Statistical Consultancy Group, Gothenburg, Sweden; ^4^Department of Obstetrics and Gynecology, Sahlgrenska University Hospital, Gothenburg, Sweden

**Introduction/Purpose:** Obstetric anal sphincter injuries (OASIs) contribute to the long‐term prevalence, severity and impact of anal incontinence. There is still no useful and generalizable prediction model for the individual, and for midwives and obstetricians to avoid this complication.

The aim was to develop validated prediction instruments for the probability of OASI in various risk scenarios, and compare model subsets based on the timeline and access to relevant predictors, and to construct an interactive tool for personal, clinical and educational use.

**Methods:** The Swedish Medical Birth Register was the main source of data: births *n* = 609 916, OASIs *n* = 25 245, study period 2009–2018. Three risk scenarios: first vaginal delivery (VD) in nulliparas (high risk, ~6%, *n* = 332 457), first VD after prior cesarean (high risk, ~10%, *n* = 22 829) and 2nd VD (low risk, ~1%, *n* = 254 630). Multiple logistic regression with backward elimination was used with minimization of Bayesian Information Criteria to select predictors for the final model. Data were split into development and validation parts for external narrow validation with bootstrapping to ensure stability and reduce overfitting.

**Results:** Fetal weight, vacuum extraction, prior OASI and maternal age were the strongest predictors for OASI. Without information on fetal weight and instrumental delivery antenatally, the c‐statistics dropped from 0.71, 0.67 and 0.79 to 0.68, 0.59, and 0.70 in the three risk scenarios, respectively.

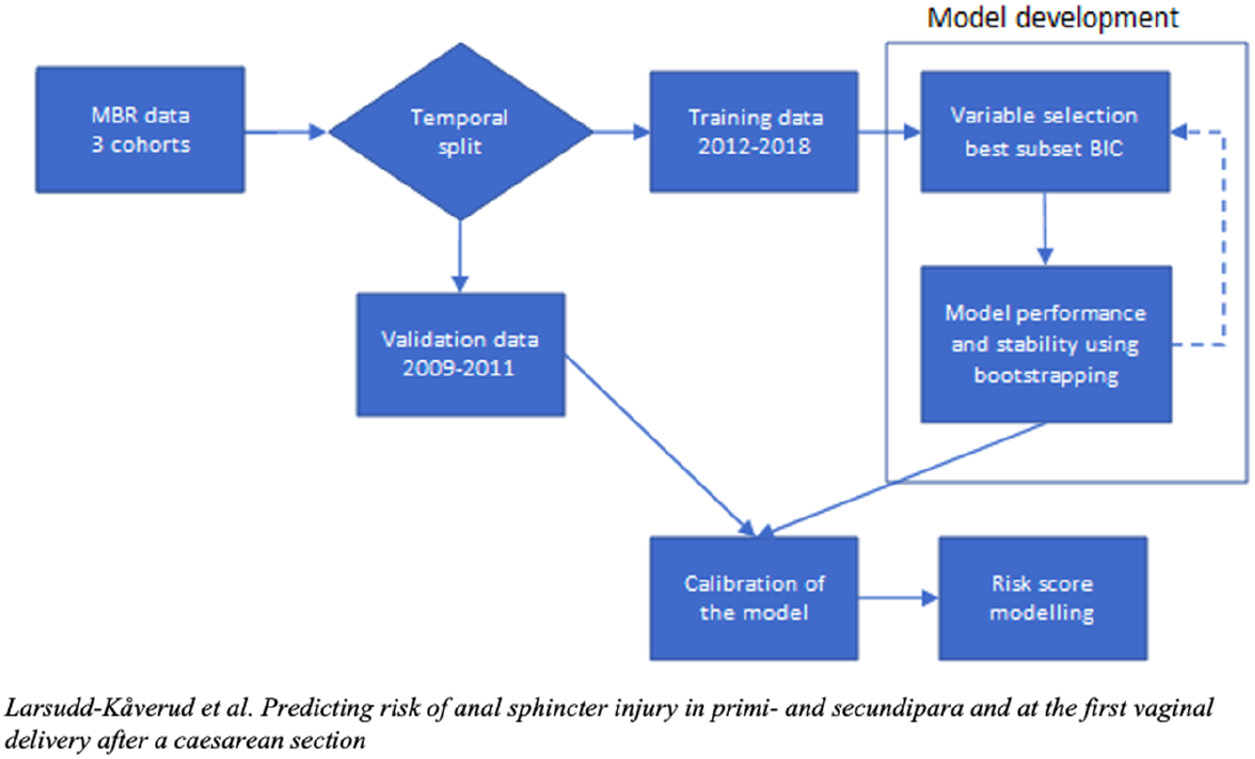

**Conclusions:** Provided that fetal biometrics are assessed antenatally, all models were accurate and useful in predicting OASI. This information supports ongoing efforts to use and further develop ultrasound and MRI imaging techniques for prenatal fetal biometry.

## Pregnant women's experiences with severe chronic pelvic pain treated with manual/musculoskeletal medicine: a qualitative interview study

### **Sif Emilie Carlsen** ^1^, Signe de Place Knudsen^1,2^, Erik Jensen^3^, Stig Molsted^4,5^, Ellen Løkkegaard^1,5^, Rie Adser Vikus^1^, Jane Bendix^1,4^

#### ^1^Department of Gynecology and Obstetrics, Copenhagen University Hospital – North Zealand, Hillerød, Denmark; ^2^Department of Biomedical Sciences, University of Copenhagen, Copenhagen, Denmark; ^3^Department of Anesthesiology, Copenhagen University Hospital – North Zealand, Hillerød, Denmark; ^4^Department of Clinical Research, Copenhagen University Hospital – North Zealand, Hillerød, Denmark; ^5^Department of Clinical Medicine, University of Copenhagen, Copenhagen, Denmark

**Introduction/Purpose:** During pregnancy, many women experience musculoskeletal pain. There is an increased tendency for pregnant women to be on long‐term sick leave due to musculoskeletal pain. Evidence for the effect of Manual/Musculoskeletal Medicine is sparse, including how pregnant women experience the treatment. This qualitative pilot study seeks to investigate women's experiences with severe chronic pelvic pain treated with Manual/Musculoskeletal Medicine during their pregnancies.

**Methods:** We conducted 10 semi‐structured telephone interviews with women who experienced musculoskeletal pain and were treated with Manual/Musculoskeletal Medicine at North Zealand Hospital during their pregnancies. The verbatim transcribed interviews were analyzed with a systematic text condensation approach.

**Results:** Overall, we identified four themes; *Before treatment – About being trapped in pain isolation*, *The encounter with the system – The game of chances*, *Experiences with the course of treatment – Holistic treatment*, and *After the treatment – A new life*. All 10 informants experienced pain reduction after treatment. However, one informant found the treatment so painful that she dropped out of the intervention. Nine informants experienced that they got a new life due to the treatment. Besides gaining a positive physical effect, they also encountered a positive psychological effect; the pain was subsequently easier to handle when they knew where to reach out for help.

**Conclusions:** Most women included had positive experiences with the Manual/Musculoskeletal Medicine and found it easier to manage musculoskeletal pain during pregnancy with the treatment. The results will be used as groundwork for a larger prospective study.

## Preterm birth among women living with HIV in Sweden

### **Kristina Pettersson** ^1,2^, Johanna Andersson^1^, Elin Jones^1^, Karin Pettersson^1,2^

#### ^1^Pregnancy and Delivery Unit, Women's Health, Karolinska University Hospital, Stockholm, Sweden; ^2^Obstetrics and Gynecology Department, Clintec, The Karolinska Institute, Stockholm, Sweden

**Introduction/Purpose:** Preterm birth is a major obstetric challenge. An increased risk of preterm birth is observed in women living with HIV in low‐income countries. This could be due to viral load, treatment or other co‐variants. In this study we wanted to investigate whether women with well‐treated HIV in a high‐income context are at an increased risk of preterm birth compared with a matched group of HIV‐negative controls.

**Methods:** 203 women giving birth at Karolinska University Hospital between 2013 and 2019 were identified from the InfCare® register. HIV‐negative controls were collected from the Swedish Pregnancy register®, matched 1:1 regarding maternal age, country of birth, and region and year of delivery. Primary outcome was preterm birth occurring before week 37+0 weeks. Secondary outcomes were other obstetric and neonatal outcomes.

**Results:** Among the controls, 9/182 women had a preterm birth, compared with 10/203 among the exposed (crude odds ratio 0.67, *P* = 0.39). An adjusted analysis considering socioeconomic factors did not alter the result. Secondary outcomes were similar between the two groups. The exposed group had an increased risk for delivery by cesarean section and lower incidence of vaginal delivery.

**Conclusions:** This study did not show an increased risk for preterm birth in women with HIV giving birth at Karolinska University Hospital. Limitations of the study include a small study group as well as missing data from the Swedish Pregnancy register in the control group regarding previous preterm birth and main outcome.

## Prevalence and risk factors of intimate partner violence among pregnant women in Denmark – a register‐based study

### **Lea Bo Sønderlund Ankerstjerne** ^1,2^, Berit Schei^3^, Ditte Linde^1,2^, Hanne Kristine Hegaard^4^, Vibeke Rasch^1,2^

#### ^1^Department of Gynecology and Obstetrics, Odense University Hospital, Odense, Denmark; ^2^Department of Clinical Research, University of Southern Denmark, Odense, Denmark; ^3^Department of Public Health and Nursing, Norwegian University of Science and Technology, St. Olav's Hospital, Trondheim, Norway; ^4^Department of Obstetrics, Copenhagen University Hospital ‐ Rigshospitalet, Copenhagen, Denmark

**Introduction/Purpose:** Intimate partner violence (IPV) is one of the most common forms of violence against women and a global health problem. IPV can have serious consequences for both the woman and her offspring. The study aims to assess the prevalence and risk factors of IPV among Danish pregnant women.

**Methods:** A total of 28 747 pregnant women who attended antenatal care at Rigshospitalet, Hvidovre Hospital or the hospitals in the Region of Southern Denmark from November 2019 to September 2022, were enrolled.

The prevalence of IPV is determined through the Abuse Assessment Screening tool, which is included in the Patient Reported Information (PRO‐data) questionnaire. Pregnant women were invited to respond. To assess IPV risk factors, the PRO data are linked to the Danish National Register, which holds information about marital status, educational attainment, income, social benefits received, occupational status and socioeconomic position.

For the statistical analysis, the outcome variables are emotional IPV, physical IPV, sexual IPV within 12 months, and ever exposed to physical IPV. Explanatory variables include age, marital situation, education, occupation, income, alcohol consumption and drug abuse. To determine the association between potential risk factors and IPV exposure, logistic regression will be used to calculate the crude odds ratios (ORs) and adjusted ORs with a 95% confidence interval (CI). Statistical analysis was performed using STATA17.

The study is approved by the Danish data protection agency and permission to use the data has been granted by the Region of Southern Denmark.

**Results:** The final results will be included in the presentation.

**Conclusions:** The study is ongoing.

## Proactive vs standard support of labor – a randomized controlled clinical trial

### **Marit Larsen** ^1,2^, Maria Underdal^1,2^, Negin Sadat^2^, Ingebjørg Laache^2^, Møyfrid Brenne^2^, Raija Dahlø^1^, Stine Bernitz^3^, Eszter Vanky^1,2^

#### ^1^Department of Clinical and Molecular Medicine, Norwegian University of Science and Technology; ^2^Department of Obstetrics, St. Olav's Hospital, Trondheim University Hospital; ^3^Department of Nursing and Health Promotion, Oslo Met, Oslo, Norway

**Introduction/Purpose:** Fear of childbirth and increasing prevalence of cesarean section (CS) is an obstetric challenge worldwide. Ten percent of all nulliparous women experience prolonged latency phase, and long total labor time, with increased risk for CS, instrumental delivery, hemorrhage, fetal asphyxia and a poor birth experience. With increasing age, maternal overweight and high expectations about delivery, there is a need for individualized labor support, especially among nulliparas with prolonged latency phase.

**Aim:** To explore whether proactive support of labor (PSL) compared with established, standard support of labor (SSL) in a randomized, controlled clinical trial (RCT) setting, results in more uncomplicated, vaginal deliveries.

**Methods:** A non‐blinded RCT that compared PSL with SSL. Women in Robson group 1 with fully effaced cervix (<5 mm), 1–3 cm cervix dilation and painful contractions were included. A midwife and an obstetrician (not involved in the study) monitored and validated eligibility to the study and protocol adherence blinded for primary outcome.

**Results:** We found no difference in the total number of complicated deliveries between PSL and SSL. The numbers of instrumental deliveries, vaginal examinations, fever, hemorrhage, transfer to NICU and Apgar <7 at 5 minutes in the PSL and SSL were similar. We found a reduction of labor duration by an average of 4 hours, as well as lower usage of oxytocin dose in PSL vs SSL.

**Conclusions:** Proactive support of labor can be considered as a safe alternative option for nulliparous women with prolonged latent phase.

## Remission or persistence? A tool to predict women at high risk for persistent depressive symptoms among women with symptoms early postpartum

### **Karin Gidén** ^1^, Lisa Vinnerljung^2^, Richelle Duque Björvang^1^, Stavros Iliadis^1^, Alkistis Skalkidou^1^, Emma Fransson^1,3^

#### ^1^Department of Women's and Children's Health, Uppsala University, Uppsala, Sweden; ^2^Department of Gynecology, Region Gavleborg, Hudiksvall, Sweden; ^3^Center for Translational Microbiome Research, Department of Microbiology, Tumor and Cell Biology, Karolinska Institutet, Stockholm, Sweden

**Introduction/Purpose:** Perinatal depression (PND) is a common complication with long‐term effects on the woman and her family. About 30%–50% of newly delivered women have continued symptoms of depression at 6–12 months postpartum. There is a need for development of better tools for prediction of persistent depression after delivery. The aim was to create a tool for prediction of long‐term symptomatology in women experiencing depressive symptoms at 6 weeks postpartum, based on clinically available information.

**Methods:** Data from the Biology, Affect, Stress, Imaging and Cognition (BASIC) study were used, identifying women scoring high (≥12) on the Edinburgh Postnatal Depression Scale (EPDS) 6 weeks postpartum (*n* = 697). After exclusions due to missing data, 586 women were included. Based on earlier studies and clinical experience, relevant variables were chosen (*n* = 42). Elastic net regression was performed to identify the best predictive factors of continued symptoms 6 months postpartum vs symptom remission.

**Results:** High EPDS score pregnancy week 17 and 32, anxiety during pregnancy, high EPDS score 6 weeks postpartum, neuropsychiatric diagnoses and smoking before pregnancy were predictive of long‐term symptomatology. An equation based on the most important variables was created as a tool to predict the EPDS score at 6 months postpartum.

**Conclusions:** This study underlines the importance of screening for depressive symptoms and anxiety during pregnancy, to identify women at high risk for persisting depressive symptoms in late postpartum. This information, together with information about earlier smoking and neuropsychiatric conditions, can be used to predict long‐term depressive symptoms after delivery.

## Salivary uric acid as a predictive test of preeclampsia, pregnancy‐induced hypertension and preterm delivery: a pilot study

### **Ida Catharina Püschl** ^1^, Lisbeth Bonde^2^, Isabel C. Reading^3^, Paddy Maguire^4^, Nicholas S. Macklon^1,5^, Bas B. Van Rijn^6^

#### ^1^Department of Gynecology and Obstetrics, Zealand University Hospital, Roskilde, Denmark; ^2^Department of Gynecology and Obstetrics, Rigshospitalet, Copenhagen, Denmark; ^3^Biostatistics, Faculty of Medicine, University of Southampton, Southampton, UK; ^4^Morgan Innovation and Technology Ltd., Petersfield, UK; ^5^London Women's Clinic, London, UK; ^6^Department of Obstetrics and Gynecology, Erasmus MC Rotterdam, Rotterdam, The Netherlands

**Introduction/Purpose:** There remains a need for a non‐invasive, low‐cost way of identifying pregnant women at risk of developing hypertensive disorders. This study evaluated the predictive value of longitudinal salivary uric acid measurement.

**Methods:** Pregnant women (*n* = 137) from 20 weeks of gestation were recruited at St Richards Hospital, Chichester, UK, for this prospective cohort study. Weekly samples of salivary uric acid were analyzed until delivery. Information regarding pregnancy and labor were obtained postpartum from the patient journal. Independent *t*‐tests were used to compare mean levels of salivary uric acid in women with hypertensive complications and adverse fetal outcomes with women with normal pregnancies. Main outcome measures were preeclampsia, pregnancy‐induced hypertension, spontaneous preterm delivery and small‐for‐gestational‐age babies.

**Results:** From 21 weeks of gestation until delivery, levels of salivary uric acid increased significantly in women who subsequently developed preeclampsia and pregnancy‐induced hypertension compared with women with normal pregnancies: preeclampsia – mean at gestational age 21–24 (mean _GA21–24_) 108, 95% confidence interval [95% CI] 63–185 vs 47, 95% CI 39–55 μmol/L; *P* = 0.005; pregnancy‐induced hypertension – mean _GA21–24_ 118, 95% CI 54–258 vs 47, 95% CI 39–55 μmol/L; *P* = 0.004). Regarding spontaneous preterm delivery, salivary uric acid levels increased significantly from 29 to 32 weeks of gestation (mean _GA29‐32_ 112, 95% CI 57–221 vs 59, 95% CI 50–71 μmol/L; *P* = 0.04). Concerning small‐for‐gestational‐age babies <10th and <3rd percentile, differences in salivary uric acid levels were insignificant.

**Conclusions:** Elevated levels of salivary uric acid precede the onset of preeclampsia, pregnancy‐induced hypertension and preterm delivery. Salivary uric acid may prove to be an early biomarker of hypertensive pregnancy disorders and spontaneous preterm delivery.

## Severe COVID‐19 during pregnancy in Sweden, Norway and Denmark

### **Anne K. Örtqvist** ^1,2^, Maria C. Magnus^3^, Anna J. M. Aabakke^4,5,6^, Anne Vinkel Hansen^7,8^, Stine Kjaer Urhoj^7^, Anne‐Marie Nybo Andersen^7^, Lone Krebs^5,9^, Karin Pettersson^10,11^, Siri E. Håberg^3^, Olof Stephansson^1,11^

#### ^1^Clinical Epidemiology Division, Department of Medicine, Solna, Karolinska Institutet, Stockholm, Sweden; ^2^Department of Obstetrics and Gynecology, Visby County Hospital, Visby, Sweden; ^3^Center for Fertility and Health, Norwegian Institute of Public Health, Oslo, Norway; ^4^Department of Obstetrics and Gynecology, Copenhagen University Hospital‐Holbæk, Holbæk, Denmark; ^5^Department of Clinical Medicine, University of Copenhagen, Copenhagen, Denmark; ^6^Department Obstetrics and Gynecology, Copenhagen University Hospital–Northzealand‐Hillerød, Hillerød, Denmark; ^7^Department of Public Health, University of Copenhagen, Copenhagen, Denmark; ^8^Statistics Denmark, Copenhagen, Denmark; ^9^Department Obstetrics and Gynecology, Copenhagen University Hospital–Hvidovre, Hvidovre, Denmark; ^10^Department of Clinical Science Intervention and Technology, Karolinska Institutet, Stockholm, Sweden; ^11^Division of Women's Health, Karolinska University Hospital, Stockholm, Sweden

**Introduction/Purpose:** We aimed to explore maternal characteristics, pregnancy outcomes, vaccination status and virus variants among pregnant women admitted to intensive care units (ICU) due to severe COVID‐19 in Scandinavia.

**Methods:** All pregnant women admitted to ICU in Sweden (*n* = 96), Norway (*n* = 31) and Denmark (*n* = 16) due to severe COVID‐19 were identified from national registers and clinical databases between March 2020 and February 2022 (Denmark), August 2022 (Sweden) and December 2022 (Norway). Their background characteristics, pregnancy outcomes and vaccination status were compared with that of all birthing women and SARS‐CoV‐2 test‐positive pregnant women during the same time period. We calculated the proportion admitted to ICU per birthing and SARS‐CoV‐2 test‐positive women during the Index, Alpha, Delta and Omicron periods.

**Results:** Women admitted to ICU had higher BMI, were more often of non‐Scandinavian origin, had lower education and income levels, a higher proportion of chronic and pregnancy‐related conditions, delivered preterm, had neonates with low Apgar scores and infants admitted to neonatal care. Only 7% of those admitted to ICU had been vaccinated prior to admission. The highest proportion of women admitted to ICU per birthing (4.1 per 10 000) was during the Delta period and per test‐positive (13.6 per 1000) during the Index period.

**Conclusions:** While admission to ICU due to COVID‐19 in pregnancy was a rare event in the Scandinavian countries, women who were unvaccinated, of non‐Scandinavian origin, and with lower socioeconomic status, were at higher risk of admission to ICU. In addition, women admitted to ICU for COVID‐19 had higher risk of adverse pregnancy outcomes.

## Severe maternal obesity and active labor

### **Maja Lecic Bonnichsen** ^1^, Lill T. Nyfløt^1,2^, Guttorm Haugen^3^, Marie Cecilie Roland^4^

#### ^1^Department of Gynecology and Obstetrics, Drammen Hospital, Drammen, Norway; ^2^Norwegian Research Center for Women's Health, Oslo University Hospital, Oslo, Norway; ^3^Institute of Clinical Medicine, University of Oslo, Oslo, Norway; ^4^Department of Obstetrics, Oslo University Hospital, Rikshospitalet, Oslo, Norway

**Introduction/Purpose:** Maternal obesity is associated with prolonged duration of labor and arrested labor along with an extensive list of adverse outcomes for mother and child.

We aimed to study expulsion time (ET) in induced and non‐induced, nulliparous and parous parturients with severe pre‐pregnant obesity (pBMI ≥35).

**Methods:** “Healthy mother‐lifelong health for mother and child” was a prospective, longitudinal, observational study, conducted at Drammen Hospital between 2016 and 2019, including 166 pregnant women with pBMI ≥35. We collected detailed data on labor induction, duration of active labor (AL, from 4 cm dilation and contractions) including expulsion time (ET), and mode of delivery. We compared groups by median regression, adjusting for parity.

**Results:** Among the 125 women (75%) with vaginal birth, 90 women (72%) were induced and 8 women (6%) were delivered by vacuum. 54% of induced women and 63% of non‐induced were parous. Median duration of AL was 3 hours (P0; 4 hours vs *P* ≥ 1; 2 hours, *P* = 0.091) in non‐induced labors and 3.5 hours (P0; 5 hours vs *P* ≥ 1; 2 hours, *P* = 0.008) in induced labors, while ET was 19.5 minutes (P0; 29 minutes vs *P* ≥ 1; 11 min, *P* = 0.006) and 17 minutes (P0; 25.5 minutes vs *P* ≥ 1; 8, *P* = 0.001), respectively. Neither duration of active labor (*P* = 0.540) nor expulsion time (*P* = 0.583) was significantly different in the two labor groups.

**Conclusions:** Despite the majority of labors being induced, we found that 75% delivered vaginally with a short median duration of active labor and expulsion time. We found no significant differences in active labor or expulsion time when comparing non‐induced and induced labors.

## Severe, but not life‐threatening – an increasing trend of severe postpartum hemorrhage was not followed by an increase in maternal near‐miss

### **Silje Pettersen** ^1,2^, Ragnhild Sørum Falk^3^, Siri Vangen^1,2^, Lill Trine Nyfløt^1,4^

#### ^1^Norwegian Research Center for Women's Health, Oslo University Hospital, Oslo, Norway; ^2^Institute of Clinical Medicine, University of Oslo, Oslo, Norway; ^3^Oslo Center for Biostatistics and Epidemiology, Oslo University Hospital, Oslo, Norway; ^4^Department of Obstetrics, Drammen Hospital, Drammen, Norway

**Introduction/Purpose:** An increasing trend of postpartum hemorrhage (PPH) has been described in several high‐income countries for the past three decades. We aimed to explore the trend of severe PPH at Oslo University Hospital, Norway.

**Methods:** A retrospective hospital‐based study performed during 2008–2017. The total number of deliveries in the period was 96 313. Severe PPH was defined as blood loss ≥1500 mL or transfusion of red blood cells. We defined a maternal near‐miss as receiving six or more bags of red blood cells, or undergoing hysterectomy or uterine embolization.

**Results:** Among 96 313 deliveries during the 10‐year study period, 2621 (2.7%) were diagnosed with severe PPH. The incidence rate doubled from 17.1/1000 in 2008 to 34.2/1000 in 2017, with an estimated annual percentage change of 6.0% (95% CI 4.6–7.5; *P* < 0.001). We also observed an increased rate of women receiving blood transfusion due to PPH, from 12.2/1000 in 2008 to 27.5/1000 in 2017, with a yearly increase of 6.3% (95% CI 4.7–8.0; *P* < 0.001).The incidence of transfusion of six or more units did not significantly increase throughout the study period (4.5%, 95% CI 0.8–10.0; *P* = 0.10). Maternal near‐miss occurred in 2.2/1000 deliveries (*n* = 211), and a non‐significant trend or increase was observed (4.6%, 95% CI 0.3–9.7; *P* = 0.06).

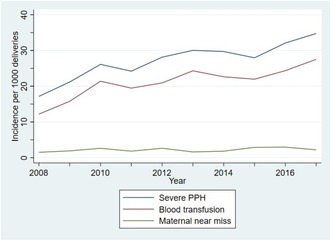

**Conclusions:** We found a significant increasing trend of severe PPH and related blood transfusions during the 10‐year study period, but this did not correspond with an increase in maternal near‐miss.

## Short inter‐pregnancy interval and birthweight: a follow‐up study of all women in Norway with two singleton deliveries during 1970–2019

### **Anne Eskild** ^1,2^, Irene Skau^3^, Camilla Haavaldsen^1^, Jostein Grytten^3^

#### ^1^Department of Obstetrics and Gynecology, Akershus University Hospital, Lørenskog, Norway; ^2^Institute of Clinical Medicine, University of Oslo, Oslo, Norway; ^3^Department of Community Dentistry, University of Oslo, Oslo, Norway

**Introduction/Purpose:** Short inter‐pregnancy interval has been associated with increased risk of low birthweight and other adverse pregnancy outcomes. Recent Nordic data are lacking. We studied mean changes in birthweight and gestational age from the first to the second delivery according to length of the inter‐pregnancy interval.

**Methods:** We followed all women in Norway from the first to the second consecutive singleton delivery at gestational week 22 or beyond during 1970–2019, covering a total of 654 100 women. Data were obtained from the Medical Birth Registry of Norway.

**Results:** Mean birthweight increase from the first to the second delivery was highest in pregnancies conceived <6 months after the first delivery. Adjusted mean increase was 227 g (95% confidence interval [CI] 219–236 g), 90 g higher than in pregnancies conceived 6–11 months after the first delivery (137 g, 95% CI 130–144 g). After exclusion of women with stillbirth at the first delivery, the adjusted mean increase in birthweight at inter‐pregnancy intervals <6 months was attenuated (152 g, 95% CI143–160 g) but remained higher than at longer inter‐pregnancy intervals. Also, mean increase in gestational age at delivery was highest at the shortest interval (2.3 days, 95% CI 2.1–2.5 days). In women with a live‐born infant weighing <2500 g at first delivery, mean birthweight increased by around 1000 g at the second delivery, and the increase was most prominent after <6‐month inter‐pregnancy intervals.

**Conclusions:** We found the highest mean increase in birthweight and in gestational age at the shortest inter‐pregnancy intervals. Our results do not generally discourage a short pregnancy interval.

## Sleep duration and sleep loss during pregnancy in women with gestational diabetes

### **Päivi** **Polo‐Kantola** ^1^ ^,2^, Otto Forsbom^3,4^, Linnea Karlsson^5,6,7^, Hasse Karlsson^5,7,8^, Laura Perasto^5^

#### ^1^Department of Obstetrics and Gynecology, Turku University Hospital and University of Turku, Turku, Finland; ^2^Sleep Research Unit, Department of Physiology, University of Turku, Turku, Finland; ^3^Department of Obstetrics and Gynecology, Lohja Distric Hospital, Lohja, Finland; ^4^Department of Obstetrics and Gynecology, University of Turku, Turku, Finland; ^5^ FinnBrain Birth Cohort Study, Turku Brain and Mind Center, Institute of Clinical Medicine, University of Turku, Turku, Finland; ^6^Department of Child Psychiatry, Turku University Hospital and University of Turku, Turku, Finland; ^7^Center for Population Health Research, Turku University Hospital and University of Turku, Turku, Finland; ^8^Department of Psychiatry, Turku University Hospital and University of Turku, Turku, Finland

**Introduction/Purpose:** To evaluate the association between sleep duration and sleep loss during pregnancy and the incidence and severity of gestational diabetes (GDM).

**Methods:** A cohort of 3738 women enrolled in the FinnBrain Birth Cohort Study. For sleep evaluation, the Basic Nordic Sleep Questionnaire regarding the sleep duration and sleep loss in early, mid‐ and late pregnancy and at delivery was used.

**Results:** When the women were divided into short (<7 hours/night), intermediate (≥7–<9 hours/night) and long (≥9 hours/night) sleepers, in the GDM group, there were more long sleepers in early (*P* = 0.031) and late (*P* = 0.022) pregnancy time points. In mid‐pregnancy, the sleep loss was higher in the GDM group (*P* = 0.002) but no other differences between the groups at other pregnancy time‐points emerged. Women whose GDM was diagnosed before 24 gestational weeks (gw) were more likely to be long sleepers in mid‐pregnancy compared with women diagnosed after 24 gw (*P* = 0.023). The sleep duration increased between mid‐ and late pregnancy in women whose GDM was diagnosed after 24 weeks (*P* = 0.032).

**Conclusions:** Women with GDM were more likely to be long sleepers and had more sleep loss, although these differences were not found consistently in the course of the pregnancy. Our results can be interpreted as deterioration in sleep in women with GDM, which may be in association with adverse pregnancy outcomes.

## Temporal trends in severe postpartum hemorrhage among nulliparous women with a spontaneous onset of labor at term

### **Camilla Tjønneland Mentzoni** ^1^, Kari Klungsøyr^1,2^, Hilde Marie Engjom^1^

#### ^1^Norwegian Institute of Public Health, Oslo, Norway; ^2^Department of Global Public Health and Primary Care, University of Bergen, Bergen, Norway

**Introduction/Purpose:** Increasing rates of postpartum hemorrhage (PPH) have been reported in high‐income countries during recent decades. Changes in maternal characteristics and increased interventions have been suggested as explanations for this rising trend. Our aim was to investigate the incidence of PPH among nulliparous women with a spontaneous onset of labor at term and temporal trends in this group from 2000 to 2020.

**Methods:** A population‐based cohort study using data from the Medical Birth Registry of Norway (MBRN) from 2000 to 2020. Nulliparous women with a singleton fetus in a cephalic presentation at term (37+0 to 41^+^6 weeks) and spontaneous onset of labor were included. The primary exposure was the calendar year of birth, and the primary outcome was severe PPH, defined as blood loss >1500 mL and/or blood transfusion. Cross‐tables and regression analysis were used to assess time trends and to adjust for potential confounders. The period 2000–2004 was used as reference.

**Results:** 330 244 women were included and 7601 (2.30%) had severe PPH. The incidence increased from 1.24% (950/76 775) in 2000–2004 to 3.83% (3390/88 580) in 2015–2020, relative risk (RR) 3.09 (95% confidence interval [CI] 2.88–3.32). Adjustment for maternal age, epidural analgesia and augmentation with oxytocin resulted in minor changes in the estimate (adjusted RR 2.90, 95% CI 2.70–3.12).

**Conclusions:** We found an increasing incidence of severe PPH among nulliparae with a spontaneous onset of labor. The increase in incidence is not solely explained by known risk factors and changes in maternal characteristics.

## The community‐based bilingual doula – a new actor filling gaps in labor care for migrant women. Midwives’ and obstetricians’ experiences

### **Erica Schytt** ^1,2,3^, Anna Wahlberg^1^, Rhonda Small^4^, Amani Eltayb^1^, Helena Lindgren^1^

#### ^1^Karolinska Institutet, Division of Reproductive Health, Department of Women's and Children's Health, Stockholm, Sweden; ^2^Center for Clinical Research Dalarna – Uppsala University, Falun, Sweden; ^3^Western Norway University of Applied Sciences Norway, Faculty of Health and Social Sciences, Bergen, Norway; ^4^La Trobe University, Judith Lumley Centre, Melbourne, Victoria 3086, Australia

**Introduction/Purpose:** To explore midwives’ and obstetricians’ views about community‐based bilingual doula (CBD) support during migrant women's labor and birth and their experiences of collaborating with CBDs.

**Methods:** A qualitative study with semi‐structured individual interviews with seven midwives and four obstetricians holding clinical positions in labor care in Stockholm, Sweden, who all had experiences of working with a CBD. Data analysis followed the framework of thematic analysis.

**Results:** The overarching theme was *A new actor filling gaps in labor care – with appropriate boundary setting, CBDs can help improve care for migrant women*. One year after the introduction of CBDs, the midwives and obstetricians had mainly positive experiences of CBDs, who were considered to fill important gaps in maternity care for migrant women, being with the woman and simultaneously being part of the care team, and this made providing high quality care easier. The CBDs’ main contribution was to help migrant women navigate the maternity care system, to bridge language and cultural divides, and guarantee continuous labor and birth support. However, midwives and obstetricians sometimes experienced CBDs interfering with their professional assessments and decisions, and the role of the CBD was somewhat unclear to them.

**Conclusions:** Community‐based bilingual doula support was viewed as improving migrant women's well‐being during labor and birth and increasing the possibilities for midwives and obstetricians to provide good and safe care; however, some ambivalence remained about the CBDs’ role and boundaries.

## The impact of exclusion due to COVID‐19 restrictions on partners’ satisfaction with Swedish hospital postnatal ward care

### **Lisa Berglin** ^1^, Verena Sengpiel^2^

#### ^1^Department of Obstetrics and Gynecology, Institute of Clinical Science, Sahlgrenska Academy, University of Gothenburg Region Västra Götaland, Sahlgrenska University Hospital, Department of Obstetrics and Gynecology, Gothenburg, Sweden; ^2^Department of Obstetrics and Gynecology, Institute of Clinical Science, Sahlgrenska Academy, University of Gothenburg Region Västra Götaland, Sahlgrenska University Hospital, Department of Obstetrics and Gynecology, Gothenburg, Sweden

**Introduction/Purpose:** To prevent the spread of SARS‐CoV‐2, hospitals around the world adopted protocols that in various ways resulted in the exclusion of partners from hospital postnatal care wards. The objective of this study was to examine the effect this exclusion had on partners’ satisfaction with postnatal care.

**Methods:** An on‐line survey (OLS; the Swedish Pregnancy Panel) including free text comments was conducted before and during the first wave of the COVID‐19 pandemic; partners of pregnant women were recruited at an early ultrasound appointment and followed until 2 months after childbirth. Data were linked to the Swedish Pregnancy Register.

**Results:** The survey was completed by 524 partners of women who gave birth during the pandemic and 203 partners of women who gave birth before. Partner's satisfaction with hospital postnatal care dropped 29.8% (−0.94 OLS, 95% CI = −1.17 to −0.72). The drop was largest for partners of first‐time mothers (−1.40 OLS, 95% CI = −1.69 to −1.11) but was unrelated to clinical outcomes such as mode of birth, and most social backgrounds, except higher income. The qualitative analysis showed that partners (1) felt excluded, as partners and parents, (2) thought the strain on staff led to deficiencies in the care provided and (3) perceived the decision regarding partner restrictions as illogical.**Conclusions:** The exclusion of partners from the hospital postnatal wards clearly impaired satisfaction with care, and partners of first‐time mothers were particularly affected. Planning for future restrictions of partners from hospital wards should factor in these consequences.

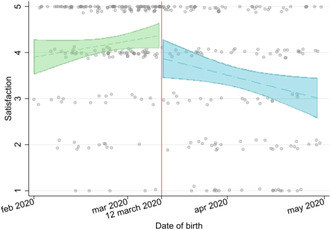

## The Norwegian WHO Labor Care Guide trial: a stepped wedge non‐inferiority trial for safety and wellbeing in labor

### Rebecka Dalbye^1,2^, **Stine Bernitz** ^1,2^, Pål Øian^3^, Torbjørn Moe Eggebø^4,5^, Annetine Staff^6,7^, Ingvil Krarup Sørbye^6,7^, Trond Melbye Michelsen^6,7^, Olufemi Oladapo^8^, Ana Pilar Betran^8^

#### ^1^Department of Obstetrics and Gynecology, Østfold Hospital Trust, Grålum, Norway; ^2^Department of Nursing and Health Promotion, Faculty of Health Sciences, OsloMet ‐ Oslo Metropolitan University, Oslo, Norway; ^3^University Hospital of North Norway, Tromsø, Norway; ^4^National Center for Fetal Medicine, St. Olav's Hospital, Trondheim University Hospital, Trondheim, Norway; ^5^Department of Clinical and Molecular Medicine, Norwegian University of Science and Technology, Trondheim, Norway; ^6^Department of Obstetrics and Gynecology, Oslo University Hospital, Oslo, Norway; ^7^Faculty of Medicine, University of Oslo, Oslo, Norway; ^8^ UNDP/UNFPA/UNICEF/WHO/World Bank Special Programme of Research, Development and Research Training in Human Reproduction (HRP), Department of Reproductive Health and Research, World Health Organization, Geneva, Switzerland

**Introduction/Purpose:** The WHO Labour Care Guide, LCG, introduced as the “next‐generation” partograph in 2020, is the recommended tool for monitoring labor and assessing labor progression and replaces the former WHO partograph. The LCG offers timely reminders on appropriate clinical, women‐centered, individual and supportive care. Potential advantages of LCG in Norway are yet unknown, and this trial is crucial to gain knowledge before implementation can be considered in Norway.

**Methods:** A large nationwide, stepped wedge multicenter cluster randomized trial. Ten included units in the NORBIRTH network have been randomly allocated to timing for crossing over from control to intervention. The LCG will be used for monitoring labor and assessing labor progression in the intervention period/steps. The LCG defines start of active labor at 5 cm of cervical dilation and further progression stepwise with intervals of 1 cm. The LCG includes recording of supportive care and recording of shared decision making at any intervention. The LCG will be integrated in the already existing electronic patient journal system. In both control and intervention, women will be treated according to individual needs and local medical procedures as standard of care.

**Results:** The results may affect nationwide labor monitoring practices and provide knowledge needed to determine the benefits of implementing the LCG in hospitals in Norway and other countries with similar healthcare services, and enable national adaptations of the LCG, prior to enrollment.

**Conclusions:** No conclusions yet.

## The risk of preeclampsia according to gestational age

### **Camilla Haavaldsen** ^1^, Anne Eskild^1,2^

#### ^1^Department of Obstetrics and Gynecology, Akershus University Hospital, Lørenskog, Norway; ^2^Institute of Clinical Medicine, University of Oslo, Oslo, Norway

**Introduction/Purpose:** Knowledge about gestational age‐specific risk of preeclampsia is lacking. Previous studies have presented proportions and numbers of deliveries with preeclampsia, but not the risks of preeclampsia in ongoing pregnancies. When including deliveries only, the risk of preeclampsia will be overestimated in preterm pregnancies and underestimated in post‐term pregnancies. We therefore aimed to study the risk of preeclampsia according to gestational age in ongoing pregnancies.

**Methods:** We used data from the Medical Birth Registry of Norway, and included all singleton births at gestational weeks 21–43 in Norway, during 1970–2019 (*n* = 2 680 375). We calculated the risk of preeclampsia according to gestational age. The denominator was all women with an ongoing pregnancy at the gestational week being studied, and the numerator was women with preeclampsia at that same week of gestation.

**Results:** In preterm pregnancies, the overall risk of preeclampsia was low: 0.02% at gestational week 28, 0.05% at gestational week 32, and 0.18% at gestational week 36. In term and post‐term pregnancies, the risk of preeclampsia displayed a steeper increase according to gestational age, and the risk was 0.3% at gestational week 38, 0.97% at gestational week 40 and 1.31% at gestational week 42.

**Conclusions:** The risk of preeclampsia increased with increasing gestational age, and the absolute increase was highest beyond gestational week 37. In recent years, there has been a decline in ongoing post‐term pregnancies due to induction of labor. This decline could in part explain the decreased prevalence of preeclampsia in Norway.

## Unplanned pregnancy and neonatal outcome: a retrospective cohort study

### **Isa Reuterwall** ^1^, Jenny Niemeyer Hultstrand^2^, Alisa Carlander^1^, Maria Jonsson^2^, Tanja Tydén^2^, Merit Kullinger^1^

#### ^1^Department of Obstetrics and Gynecology, Region Västmanland, Västerås, Sweden; ^2^Department of Women's and Children's Health, Uppsala University, Uppsala, Sweden

**Introduction/Purpose:** Unplanned pregnancy is common, and although some research indicates adverse outcomes for the neonate, such as death, low birthweight and preterm birth, results are diverse. The purpose of the present study was to investigate associated neonatal outcomes of an unplanned pregnancy in a Swedish setting.

**Methods:** We conducted a retrospective cohort study in which data from 2953 women were retrieved from the Swedish Pregnancy Planning Study, covering nine Swedish counties from September 2012 through to July 2013. Pregnancy intention was measured using the London Measurement of Unplanned Pregnancy. Women with unplanned pregnancies and pregnancies of ambivalent intention were combined and referred to as unplanned. Data on neonatal outcomes (small for gestational age, low birthweight, preterm birth, Apgar score <7 at 5 minutes, and severe adverse neonatal outcome defined as death or need for resuscitation at birth) were retrieved from the Swedish Medical Birth Register.

**Results:** The prevalence of unplanned pregnancies was 31%. Women with unplanned pregnancies were younger and had a lower degree of education. Compared with women who had planned pregnancies, those with unplanned pregnancies were more likely to give birth to neonates that were small for gestational age: 3.7% vs 1.7% (adjusted OR 2.0, 95% CI 1.2–3.5). There were no significant differences in preterm birth, Apgar score <7 at 5 minutes or severe adverse neonatal outcome.

**Conclusions:** In a Swedish setting, an unplanned pregnancy may be a risk factor for a small‐for‐gestational‐age neonate. We found no other adverse effects on the neonate of unplanned pregnancies.

## Unplanned pregnancy is associated with a low risk for women during pregnancy and labor: a Swedish cohort study

### **Alisa Carlander** ^1^, Jenny Niemeyer Hultstrand^2^, Isa Reuterwall^1^, Maria Jonsson^2^, Tanja Tydén^2^, Merit Kullinger^1^

#### ^1^Department of Obstetrics and Gynecology, Region Västmanland, Västerås, Sweden; ^2^Department of Women's and Children's Health, Uppsala University, Uppsala, Sweden

**Introduction/Purpose:** Unplanned pregnancies are common and are associated with late initiation and inadequate use of antenatal care. How pregnancy planning relates to maternal health and delivery in Sweden, a country with free antenatal care and free abortion, has not been studied previously. Our aims were to study whether pregnancy planning was associated with antenatal care utilization and maternal health outcomes in Sweden.

**Methods:** Data for 2953 women from the Swedish Pregnancy Planning cohort study were linked to the Swedish Medical Birth Register. The degree of pregnancy planning was estimated using the London Measure of Unplanned Pregnancy. Women with an unplanned and ambivalent intention to become pregnant were merged and termed unplanned. Data were analyzed using Fisher's exact test and regression analyses.

**Results:** Most (69%) women reported a planned pregnancy, and 31% an unplanned pregnancy. Women with an unplanned pregnancy enrolled later in the antenatal care unit. These women were less likely to consume folic acid and had higher rates of smoking. Women with an unplanned pregnancy were more likely to have induced labor and a longer hospital stay. No associations were found between pregnancy planning and pregnancy‐induced hypertension, gestational diabetes mellitus, preeclampsia, vacuum extraction delivery, cesarean section, epidural analgesia or sphincter rupture.

**Conclusions:** Unplanned pregnancy was associated with delayed initiation of antenatal care. Pregnancy planning was not related to maternal outcomes in a Swedish setting. These findings suggest that behavior changes when the pregnancy is recognized and that women with an unplanned pregnancy can cope well in a setting with available support.

## What do we register? The validity of prenatal audit screening for alcohol disorders – a Swedish register study

### Susanne Hesselman^1,2^, **Joline Asp** ^1,3^, Ulrika Pellas^2^, Susanne Lager^1^, Anna Wikman^1^

#### ^1^Department of Women's and Children's Health, Uppsala University, Uppsala, Sweden; ^2^Center for Clinical Research Dalarna, Falun, Sweden; ^3^Center for Clinical Research Sörmland, Uppsala University, Eskilstuna, Sweden

**Introduction/Purpose:** Nordic countries have a long tradition of using population‐based data for monitoring healthcare and research. The objective of this study was to assess the external validity of the Alcohol Use Disorders Identification Test (AUDIT) in Swedish prenatal care as an indicator of alcohol‐addiction disorders.

**Methods:** Prenatal AUDIT screening points in the Swedish Pregnancy Register of 739 735 pregnancies were linked to national health databases. The AUDIT score was dichotomized into <6 points (low‐risk use) and ≥6 points (hazardous use). Alcohol disorders were defined by a diagnostic code or drugs dispensed for alcohol dependence in national health registers. The diagnostic accuracy of an AUDIT score of ≥6 points for alcohol‐addiction disorders was calculated. Women with mismatched information in the register were characterized, and compared by multinominal logistic regression with odds ratio (OR) with 95% confidence interval (CI) to women with matched information.

**Results:** Alcohol disorder was recorded in 3.1%; 3.5% reported an AUDIT ≥6 points. The diagnostic accuracy of the AUDIT ≥6 points for detection of an alcohol disorder the year prior to pregnancy was 95.7%, with a positive likelihood ratio of 8.03 (95% CI 7.5–8.6). The sensitivity was 33.0%. Younger, nulliparous, low education and of Swedish origin increased the likelihood of being misclassified with the AUDIT. Neuropsychiatric disorders were associated with being classified false‐negative (OR 10.39, 95% CI 9.89–10.90).

**Conclusions:** The accuracy of AUDIT in screening for alcohol disorders in a low‐risk use population was high but only identified one‐third of women with alcohol‐related disorders when using a cut‐off of six points.

## When to induce for overweight (the WINDOW study): an ongoing randomized controlled trial

### **Lise Qvirin Krogh** ^1,2^, Sidsel Boie^1,3^, Tine Brink Henriksen^2,4^, Jim Thornton^5^, Jens Fuglsang^1,2,6^, Julie Glavind

#### ^1^Department of Obstetrics and Gynecology, Aarhus University Hospital, Aarhus, Denmark; ^2^Department of Clinical Medicine, Aarhus University, Aarhus, Denmark; ^3^Department of Obstetrics and Gynecology, Aalborg University Hospital, Aalborg, Denmark; ^4^Department of Pediatrics, Aarhus University Hospital, Aarhus, Denmark; ^5^Department of Obstetrics and Gynecology, Nottingham University, Nottingham, UK; ^6^Steno Diabetes Center, Aarhus University Hospital, Aarhus, Denmark

**Introduction/Purpose:** In fertile women, the prevalence of obesity is 33% in the US, 20% in the UK, and 13% in Denmark. Obesity is associated with both pregnancy and labor complications. It has been suggested that induction of labor may reduce the risk of adverse events in women with obesity, but there have been no clinical trials and the impact on the rate of cesarean delivery remains unknown. The objective of this study is to assess birth outcomes in women with obesity when labor is induced at 39 weeks of gestation compared with expectant management.

**Methods:** The WINDOW study is an open label, randomized, controlled, multicenter trial conducted at delivery departments with an in‐house neonatal intensive care unit. Recruitment started October 2020. A total of 1900 women with a pre‐pregnancy BMI ≥30 kg/m^2^ are randomized in a 1:1 ratio to either labor induction at 39 +0–3 weeks of gestation, or to expectant management; ie waiting for spontaneous labor onset or induction if medically indicated. The primary outcome is cesarean delivery. Secondary outcomes consist of maternal adverse outcomes, a composite of adverse neonatal outcomes, maternal birth experience and postnatal depression. Data will be analyzed according to intention‐to‐treat.

**Results:** The study is ongoing. A total of 666 women have been enrolled from 11 recruiting sites. Preliminary results are not available.

**Conclusions:** Results of this trial will provide important knowledge about timing of delivery in women with obesity and will add information to an ongoing discussion of the overall effects of induction before term.

## Women born in Sub‐Saharan countries have an increasing incidence of stillbirth – a cohort study in Stockholm 2001–2020

### **Minna Lundén** ^1^, Ingela Hulthén Varli^2^, Helena Kopp Kallner^1^, Hanna Åmark^3^

#### ^1^Department of Clinical Sciences, Danderyd Hospital, Karolinska Institute, Stockholm, Sweden; ^2^Department of Women's and Children's Health, Karolinska Institute, Stockholm, Sweden; ^3^Department of Clinical Science and Education, Unit of Obstetrics and Gynecology, Södersjukhuset, Karolinska Institute, Stockholm, Sweden

**Introduction/Purpose:** The incidence of stillbirth in Sweden has started to decline; however, some comparable high‐income countries in Europe have an even lower incidence, indicating a potential for further reduction. The aim of our study was to investigate how the incidence of stillbirth for singleton pregnancies in different groups of women has changed over the past two decades in Stockholm County in order to detect the groups at highest risk.

**Methods:** This was a cohort study with data from the Stockholm Stillbirth Database including all cases of stillbirth in singleton pregnancies between 2001 and 2020, in total 1821 stillbirths. The time period was divided in four equal groups and the incidence of stillbirth was compared between the groups.

**Results:** The overall incidence of stillbirth in the Stockholm County has decreased from 3.8/1000 births in 2001–2005 to 2.9/1000 births in 2016–2020 (*P*‐value < 0.001). The highest incidence of stillbirth regardless of time period was among women originating from Sub‐Saharan Africa. Their incidence of stillbirth significantly rose from 7.9/1000 births in 2001–2005 to 10.1/1000 births in 2016–2020 (*P*‐value 0.025). Compared with women from other regions of the world, stillbirth in this group occurred at earlier gestational weeks and the proportion of small‐for‐gestational‐age infants was higher.

**Conclusions:** The incidence of stillbirth in Stockholm County has declined. However, among women originating from Sub‐Saharan Africa the incidence was significantly higher and still rising. More research is needed to understand why this group is at higher risk and how to monitor their pregnancies to decrease the risk.

## Women's perceptions of biological causes and potentiality of genomic risk markers in postpartum depression

### **Stinne Høgh** ^1,2,3^, Hanne Kristine Hegaard^2,3^, Kristina Martha Renault^3^, Mette Nordahl Svendsen^4^, Laura Emdal Navne^5^, Vibe Gedsø Frøkjær^1,2,6,7^

#### ^1^Neurobiology Research Unit, Copenhagen University Hospital ‐ Rigshospitalet, Copenhagen, Denmark; ^2^Department of Clinical Medicine, Faculty of Health and Medical Sciences, University of Copenhagen, Copenhagen, Denmark; ^3^Department of Obstetrics, Copenhagen University Hospital ‐ Rigshospitalet, Copenhagen, Denmark; ^4^Department of Public Health, Section for Health Services Research, University of Copenhagen, Copenhagen, Denmark; ^5^Danish Center for Social Science Research, VIVE, Copenhagen, Denmark; ^6^Department of Psychiatry, Psychiatric Center Copenhagen, Copenhagen, Denmark; ^7^Department of Psychology, University of Copenhagen, Copenhagen, Denmark

**Introduction/Purpose:** Novel evidence suggests that genomic markers for enhanced hormone sensitivity, in terms of estradiol signaling, may help identify women at high risk of postpartum depression. We explored how women with a history of postpartum depression perceived hypothetical screening for enhanced hormone sensitivity.

**Methods:** We conducted semi‐structured interviews with 13 Danish women who had a history of postpartum depression using a phenomenological approach. A transdisciplinary group of researchers analyzed the interviews thematically. Through the concept of *potentiality*, we unfolded the women's perceptions of screening for hormonal sensitivity to profile their risk of postpartum depression.

**Results:** We identified three key themes. (1) *Biology as a contributing factor to postpartum depression*. A few women believed postpartum depression could be related to sensitivity to hormonal changes. (2) *The role of external events in making sense of postpartum depression*. Most women perceived their postpartum depression as primarily triggered by external factors rather than biological factors. (3) *The ambiguous potentiality of genetic testing for risk of postpartum depression*. Hormone sensitivity testing was envisioned by some women as having the potential to prevent postpartum depression or reduce stigma but also the potential to induce depressive symptoms.

**Conclusions:** Women with a history of postpartum depression perceived the potentiality of screening for hormonal sensitivity with ambiguity. Knowledge about genomic risk markers introduced hope regarding opportunities for prevention and, at the same time, concerns about potentially inducing depressive symptoms. We suggest considering such perceptions if implementing new genetic technologies in risk profiling to direct preventive strategies for postpartum depression.

## Causes of death in stillbirth and quality of care – an example of a local audit

### **Frida Rensfeldt** ^1^, Malin Huber^2^, Katarina Tunón^2^

#### ^1^Department of Obstetrics and Gynecology, Östersund Hospital, Östersund, Sweden; ^2^Department of Clinical Science, Obstetrics and Gynecology, Umeå University, Umeå, Sweden

**Introduction/Purpose:** Neonatal deaths have decreased steadily in Sweden since the 1960s, but the stillbirth rate has been stable at around 4 per 1000 births for the last 40 years. Risk factors of stillbirth are well studied; however, the cause of death is unclear in many cases. The purpose of this study was to examine causes of death in stillbirth using a classification system. We also aimed to assess substandard care factors.

**Methods:** A retrospective clinical audit of all stillbirths delivered at Östersund County Hospital between 2006 and 2019. We performed a detailed investigation of medical records and classified the causes of death according to The Stockholm classification of stillbirth. Substandard care factors were identified and classified as probable, possible or unlikely, with respect to the cause of death.

**Results:** There were 64 cases of stillbirth. It was possible to determine a cause of death in 91% of the cases. The leading cause of death was intrauterine growth restriction (IUGR)/placental insufficiency (45%), followed by infection (13%) and placental abruption (9%). Factors of substandard care were identified in 49% of the cases but in only 10% did the substandard care factor have a probable association with the cause of death.

**Conclusions:** Introducing a local audit group and using a validated classification system made it possible to find a cause of death in about 90% of the cases of stillbirth. Since IUGR/placental insufficiency is the leading cause of death, it is important to improve current strategies to identify pregnancies at risk for IUGR, antenatally.

## Risk factors for and outcomes after SARS‐CoV‐2 in pregnancy according to disease severity: cohort study with validation of the SARS‐CoV‐2 diagnosis

### **Anna J. M. Aabakke** ^1,2^, Tanja G. Petersen^3^, Lone Krebs^2,4^, Mette Bliddal^3,5^

#### ^1^Department of Obstetrics and Gynecology, Copenhagen University Hospital ‐ Holbæk, Denmark; ^2^Department of Clinical Medicine, University of Copenhagen, Copenhagen, Denmark; ^3^ OPEN, Odense University Hospital, Odense, Denmark; ^4^Department of Obstetrics and Gynecology, Copenhagen University Hospital – Amager and Hvidovre, Hvidovre, Denmark; ^5^Research Unit OPEN, Department of Clinical Research, University of Southern Denmark, Odense, Denmark

**Introduction/Purpose:** To identify risk factors and outcomes associated with SARS‐CoV‐2 infection in pregnancy and validate information on SARS‐CoV‐2 during pregnancy in national health registers in Denmark.

**Methods:** Cohort study using data from national registers and medical records including pregnancies between March 2020 and February 2021. We compared women with a positive SARS‐CoV‐2 test during pregnancy with non‐infected pregnant women. Risk factors and pregnancy outcomes were assessed by Poisson and Cox regression models and stratified according to disease severity.

Using medical record data on actual period of pregnancy, we calculated predictive values of the SARS‐CoV‐2 diagnosis in pregnancy in the registers.

**Results:** SARS‐CoV‐2 infection was detected in 1819 (1.6%) of 111 185 pregnancies. Asthma was associated with infection (relative risk [RR] 1.63, 95% confidence interval [CI] 1.28–2.07]). Risk factors for COVID‐19 requiring hospital admission were high BMI (MR 1.06 [1.04–1.09]), asthma (RR 7.47, 95% CI 3.51–15.90) and gestational age [GA] at infection (GA 28–36 vs <22: RR 3.53, 95% CI 1.75–7.10).

SARS‐CoV‐2‐infected women more frequently had hypertensive disorders in pregnancy (adjusted hazard ratio [aHR] 1.31 [1.04–1.64]), early pregnancy loss (aHR 1.37 [1.00–1.88]), preterm delivery before GA 28 (aHR 2.31 [1.01–5.26]), iatrogenically preterm delivery before GA 37 (aHR 1.49 [1.01–2.19]) and small‐for‐gestational‐age (SGA) children (aHR 1.28 [1.05–1.54]).

The validity of the SARS‐CoV‐2 diagnosis in relation to pregnancy in the registers showed a negative predictive value of 99.9 and a positive predictive value of 82.1.

**Conclusions:** Women with SARS‐CoV‐2 during pregnancy were at increased risk of hypertensive disorders in pregnancy, early pregnancy loss, preterm delivery and having SGA children. The validity of Danish registers was acceptable for identification of SARS‐CoV‐2 infection during pregnancy.

## The association between SARS‐CoV‐2 and de novo hypertension during pregnancy – a population‐based cohort study in Sweden and Norway

### **Anne K. Örtqvist** ^1,2^, Maria C. Magnus^3^, Elisabeth Dahlqvist^1^, Jonas Söderling^1^, Kari Johansson^1,4^, Anna Sandström^1,4^, Siri E. Håberg^3^, Olof Stephansson^1,4^

#### ^1^Clinical Epidemiology Division, Department of Medicine, Solna, Karolinska Institutet, Stockholm, Sweden; ^2^Department of Obstetrics and Gynecology, Visby County Hospital, Visby, Sweden; ^3^Center for Fertility and Health, Norwegian Institute of Public Health, Oslo, Norway; ^4^Department of Women's Health, Division of Obstetrics, Karolinska University Hospital, Stockholm, Sweden

**Introduction/Purpose:** The suggested association between SARS‐CoV‐2 and de novo hypertension during pregnancy (HDP) may be due to bias. We aimed to investigate this association further, taking temporality and confounding into account.

**Methods:** The study included all pregnant women with a singleton birth after 22 gestational weeks in the Swedish Pregnancy Register and the Medical Birth Register in Norway between March 2020 and May 2022 (*n* = 312 456). De novo HDP was defined as a composite outcome of a diagnosis of gestational hypertension, preeclampsia, hemolysis, elevated liver enzymes, low platelets (HELLP) syndrome and/or eclampsia from gestational week 20 up until 1 week after delivery. Dates of all PCR‐verified SARS‐CoV‐2 tests and dates of diagnoses of HDP were collected from national health and surveillance registers. The association between SARS‐CoV‐2 and HDP was investigated using a stratified Cox proportional hazard model, with SARS‐CoV‐2 as a time‐varying variable, adjusting for background characteristics.

**Results:** SARS‐CoV‐2 infection during pregnancy was not associated with an increased risk of HDP (adjusted hazard ratio 0.99, 95% confidence interval 0.93–1.04) or with preeclampsia specifically. Results were similar for exposure to SARS‐CoV‐2 in all gestational trimesters and in different time periods corresponding to dominating virus variants.**Conclusions:** We did not find any evidence of an association between SARS‐CoV‐2 infection during pregnancy and subsequent development of HDP and preeclampsia. In this study, timing of infection and onset of HDP were accounted for, which has been lacking in previous studies. As SARS‐CoV‐2 in pregnancy is related to other adverse outcomes in pregnancy, vaccination of pregnant women in still highly recommended.

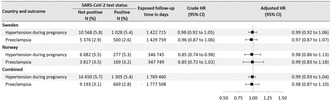

## Metformin has immunomodulatory effects in pregnant women with PCOS

### **Mariell Ryssdal** ^1,2^, Guro F. Giskeødegård^3^, Live Marie T. Stokkeland^1,2^, Anders Hagen Jarmund^1,2^, Bjørg Steinkjer^1,2^, Tone Shetelig Løvvik^1,4^, Torfinn Støve Madssen^5^, Ann‐Charlotte Iversen^1,2,4^, Eszter Vanky^1,4^

#### ^1^Department of Clinical and Molecular Medicine, Norwegian University of Science and Technology (NTNU), Trondheim, Norway; ^2^Center of Molecular Inflammation Research (CEMIR), NTNU, Trondheim, Norway; ^3^K.G. Jebsen Center for Genetic Epidemiology, Department of Public Health and Nursing, NTNU, Trondheim, Norway; ^4^Department of Obstetrics and Gynecology, St. Olav's Hospital, Trondheim University Hospital, Trondheim, Norway; ^5^Department of Circulation and Medical Imaging, NTNU, Trondheim, Norway

**Introduction/Purpose:** Polycystic ovary syndrome (PCOS) is an endocrine disorder associated with low‐grade systemic inflammation and increased risk of pregnancy complications. Metformin reduces the risk of late miscarriage and preterm birth in pregnant women with PCOS, though it is uncertain whether immunological changes play a role in this protective effect. This study aimed to investigate the effect of metformin on the maternal immunological development in women with PCOS.

**Methods:** A post‐hoc analysis was performed of two randomized controlled trials, PregMet and PregMet2, including longitudinal maternal serum samples from 615 women with PCOS. Women were randomized to metformin or placebo from first trimester to delivery. Levels of 22 cytokines and C‐reactive protein (CRP) were measured in serum sampled at gestational weeks 5–12, 19, 32 and 36.

**Results:** Metformin treatment was associated with higher serum levels of several multifunctional cytokines throughout pregnancy, with the strongest effect on eotaxin (*P* < 0.001), interleukin (IL)‐17 (*P* = 0.03) and basic fibroblast growth factor (FGF‐b) (*P* = 0.04). Assessment of the combined cytokine development confirmed the impact of metformin on half of the 22 cytokines. The immunomodulating effect of metformin was stronger in normal weight and overweight women than in obese women. Moreover, the effect of metformin was most pronounced in early pregnancy in normoandrogenic women, whereas it increased throughout pregnancy in hyperandrogenic women.

**Conclusions:** Metformin has immunomodulating properties in pregnancy. Its effect on the serum levels of many multifunctional cytokines demonstrates robust, persisting and BMI‐dependent immune mobilization in pregnant women with PCOS.

## Prenatal testosterone exposure and associations with offspring body composition at 7 years of age: Odense child cohort

### **Camilla Viola Buskbjerg Palm** ^1,2^, Dorte Glintborg^1,2^, Anders Grøntved^3^, Dorte Møller Jensen^2,4^, Jan Stener Jørgensen^2,5^, Frederik Damsgaard Højsager^6,7^, Tina Kold Jensen^6,7,8^, Marianne Skovsager Andersen^1,2^

#### ^1^Department of Endocrinology and Metabolism, Odense University Hospital, Odense, Denmark; ^2^Department of Clinical Research, Faculty of Health Sciences, University of Southern Denmark, Odense, Denmark; ^3^Department of Sports Science and Clinical Biomechanics, Research Unit for Exercise Epidemiology, University of Southern Denmark, Odense, Denmark; ^4^Steno Diabetes Center Odense (SDCO), Odense University Hospital, Odense, Denmark; ^5^Department of Gynecology and Obstetrics, Odense University Hospital, Odense, Denmark; ^6^Odense Child Cohort, Hans Christian Andersen Hospital for Children and Adolescents, Odense University Hospital, Odense, Denmark; ^7^Department of Clinical Pharmacology, Pharmacy and Environmental Medicine, University of Southern Denmark, Odense, Denmark; ^8^ OPEN Patient data Explorative Network (OPEN), University of Southern Denmark, Odense, Denmark

**Introduction/Purpose:** Prenatal testosterone exposure is associated with low birthweight and postnatal weight gain in mammals. No data on maternal testosterone levels and offspring body composition exist in human studies. Women with polycystic ovary syndrome (PCOS) have higher third‐trimester testosterone levels compared with controls; higher testosterone levels are linked to reduced birthweight, length and abdominal circumference in boys.

The aim of the study was to determine associations between prenatal exposure to testosterone, maternal diagnosis of PCOS and offspring body composition at 7 years.

**Methods:** The study is part of the prospective Odense Child Cohort study including 1486 mother–child dyads. Maternal free testosterone (FT) was calculated from total testosterone (TT) at gestational week 28. In clinical examinations at 7 years, weight, height, abdominal and head circumferences were measured. Dual X‐ray absorptiometry (DXA) estimated total body, gynoid and android fat percentages and lean body percentage. Multiple linear regression analyses were adjusted for maternal age, parity and birthweight.

**Results:** Higher maternal FT and TT were positively associated with weight (adjusted *P* = 0.014 and *P* = 0.045, respectively) and FT was positively associated with body mass index (BMI) in boys (adjusted *P* = 0.016). Maternal PCOS diagnosis was associated with lower gynoid fat percentage in girls (crude *P* = 0.05, adjusted *P* = 0.06).

**Conclusions:** Higher maternal free testosterone exposure was linked to higher weight and BMI in boys. Gynoid fat percentage was lower in girls or women with PCOS compared with controls.

## Patient‐reported satisfaction after spontaneous second‐degree tear compared with episiotomy – a Swedish register‐based cohort study

### **Mette L Josefsson** ^1^, Eva Uustal^2^, Cecilia Ekéus^1^, Sara Sohlberg^1^, Maria Jonsson^1^

#### ^1^Uppsala University, Uppsala, Sweden; ^2^Linköping University, Linköping, Sweden

**Introduction/Purpose:** Approximately 60% of primiparous women sustain a second‐degree perineal tear. There is limited knowledge about the consequences in the medium term. This study aimed to study satisfaction among first‐time mothers 1 year after a spontaneous tear compared with an episiotomy, and explore any differences between primary suture by a physician compared with a midwife.

**Methods:** This study included 5328 women with a second‐degree tear. Data were collected from the Swedish Medical Birth Register, the National Patient Register and the National Quality Register of Gynecological Surgery 2014–2019. Differences in self‐reported satisfaction between spontaneous tear and episiotomy were assessed using logistic regression and presented by odds ratios (OR) with 95% confidence intervals (CI).

**Results:** Among the 5328 women included, 81.1% sustained a spontaneous tear and 18.9% had an episiotomy. Overall, 75.2% were satisfied at 1 year. Women with an episiotomy were more dissatisfied (6.3% vs 3.6%, OR 1.7, 95% CI 1.2–2.6) or very dissatisfied (2.2% vs 0.8%, OR 2.7, 95% CI 1.4–5.2), they were also more likely to experience severe or unbearable dyspareunia (3.8 vs 2.5%, OR 1.6, 95% CI 1.0–2.5). Women sutured by a midwife were more likely to be satisfied with the outcome (OR 0.6, 95% CI 0.5–0.8).

**Conclusions:** The majority of women with a second‐degree tear reported high level of satisfaction. Women with an episiotomy were more dissatisfied compared with women with a spontaneous tear. Primary suture by a midwife compared with a physician impacted the reported satisfaction, which may reflect a variation in complexity of the second‐degree tear.

## Validation of the Finnish hospital discharge register diagnoses for preeclampsia, gestational diabetes and preterm delivery

### **Elli Toivonen** ^1^, Kirsi Taurio^1^, Eija Kortelainen^2^, Aki Havulinna^3,4^, FINNPEC, Tiina Jääskeläinen^2,5^, Hannele Laivuori^1,2,3^

#### ^1^Department of Obstetrics and Gynecology, Tampere University Hospital and Tampere University, Faculty of Medicine and Health Technology, Center for Child, Adolescent, and Maternal Health Research, Tampere, Finland; ^2^Medical and Clinical Genetics, University of Helsinki and Helsinki University Hospital, Helsinki, Finland; ^3^Institute for Molecular Medicine Finland, Helsinki Institute of Life Science, University of Helsinki, Helsinki, Finland; ^4^Finnish Institute for Health and Welfare, Helsinki, Finland; ^5^Department of Food and Nutrition, University of Helsinki, Helsinki, Finland

**Introduction/Purpose:** Centrally collected national health register data on adverse pregnancy outcomes are available for research, but the validity of the data is largely unknown. Our aim was to compare the diagnoses of preeclampsia (PE), gestational diabetes (GDM) and preterm delivery from hospital records with the registry‐based diagnoses from the Finnish Hospital Discharge Register (FHDR). Data on gestational age at delivery from the Medical Birth Registry (MBR) was also studied.

**Methods:** The Finnish Genetics of Preeclampsia Consortium (FINNPEC) Study cohort was used as a data source. Each diagnosis was ascertained from hospital records. The validity of diagnoses obtained by record linkage of FHDR and MBR was assessed against classification previously confirmed independently by a research nurse and a study physician.

**Results:** Sensitivity of PE diagnoses in the FHDR was 80.3% (95% confidence interval [CI] 78.3–82.2) and specificity 95.3% (95% CI 93.9–96.4). Sensitivity for GDM was 64.1% (95% CI 58.7–69.3) and specificity 98.5% (95% CI 97.9–98.9), whereas sensitivity and specificity for preterm delivery were 32.4% (95% CI 29.0–36.0) and 99.7% (95% CI 99.3–99.9), respectively. Sensitivity of preterm delivery in the MBR was 99.1% and specificity 99.9%.

**Conclusions:** FDHR registry diagnoses for PE have satisfactory sensitivity and high specificity. Diagnoses for GDM and preterm delivery have lower sensitivity, limiting their use in studies, and data from the MBR should be preferred when studying premature deliveries.

## Winter, spring, summer or fall: temporal patterns in placenta‐mediated pregnancy complications – an exploratory analysis

### **Maria Jeppegaard** ^1,2^, Steen C. Rasmussen^1,2,3^, Jacob Anhøj^3^, Lone Krebs^2,4^

#### ^1^Department of Gynecology and Obstetrics, University of Copenhagen, Holbæk Hospital, Holbæk, Denmark; ^2^Department of Gynecology and Obstetrics, University of Copenhagen, Amager Hvidovre Hospital, Hvidovre, Denmark; ^3^Center of Diagnostic Investigation, Rigshospitalet, University of Copenhagen, Copenhagen, Denmark; ^4^Department of Clinical Medicine, University of Copenhagen, Copenhagen, Denmark

**Introduction/Purpose:** Placenta‐mediated pregnancy complications such as growth restriction and hypertensive disorders are leading causes of maternal, fetal and neonatal morbidity as well as mortality in high‐income countries. A healthy pregnancy depends on the function and development of placenta.

The aim with this study is to investigate whether there is a seasonal variation in placenta‐mediated pregnancy complications (small for gestational age, intrauterine growth restriction, preeclampsia, preterm birth and intrauterine fetal death).

**Methods:** This is a Danish cohort study including all singleton deliveries at gestational week 22 up to and including week 41 conceived from December 2006 to November 2016 (*n* = 555 459). We used statistical process control charts to visualize data and test for patterns of non‐random variation in data over time for pregnancies with risk factors (BMI, diabetes, in vitro fertilization, maternal age >40 years, primipara, previous cesarean and smoking) and each outcome: fetal growth restriction, hypertensive disorders, preterm birth and intrauterine fetal death.

**Results:** We found a seasonal pattern in hypertensive disorders during pregnancy, with dips in pregnancies conceived in the fall season and highest risk by conception in the spring and summer season. We found no apparent seasonality in cases of preterm delivery, small for gestational age or intrauterine mortality.

**Conclusions:** In conclusion, we found a significant seasonal variation in the risk of hypertensive disorders of pregnancy, including preeclampsia, with highest risk by conception in the spring and summer season. The risk was lowest by conception in the fall. This study found no seasonal variation in other placenta‐mediated complications.

## Intimate partner violence during pregnancy and its association with adverse pregnancy outcomes in Denmark: a register‐based prospective cohort study

### **Lea Bo Sønderlund Ankerstjerne** ^1,2^, Berit Schei^3^, Karen Andreasen^1,2^, Ditte Linde^1,2^, Hanne Kristine Hegaard^4^, Vibeke Rasch^1,2^, Kristina Renault^4^

#### ^1^Department of Gynecology and Obstetrics, Odense University Hospital, Odense, Denmark; ^2^Department of Clinical Research, University of Southern Denmark, Odense, Denmark; ^3^Department of Public Health and Nursing, Norwegian University of Science and Technology, Trondheim, St. Olav's Hospital, Trondheim, Norway; ^4^Department of Obstetrics, Copenhagen University Hospital ‐ Rigshospitalet, Copenhagen, Denmark

**Introduction/Purpose:** Intimate partner violence (IPV) is one of the most common forms of violence against women and a global health problem. IPV can have serious consequences for both the woman and her offspring. The study aims to assess the association between IPV exposure and adverse pregnancy outcomes in a Danish cohort.

**Methods:** A total of 28 747 pregnant women who attended antenatal care at Rigshospitalet, Hvidovre Hospital or the hospitals in the Region of Southern Denmark from November 2019 to September 2022 were enrolled. Information about the outcome of the index pregnancy will be retrieved from The Danish Nation Patient register (in Danish Landspatientregistre [LPR2 and LPR3]) after the women have delivered. The register data will be linked to the women's questionnaire responses from their first trimester (PRO‐data). A relative risk regression will be performed and reported as crude relative risks (RR) and adjusted RR with 95% confidence intervals. The primary outcome variables will be preterm birth and low birthweight and the exposure variables will be emotional IPV, physical IPV, sexual IPV and any IPV. Relevant confounders will be included in the adjusted model based on a priori assumptions and availability in the Danish National Register and The Danish Health Data Authority (in Danish “Sundhedsdatastyrelsen”).

The study is approved by the Danish data protection agency and permission to use the data has been granted by the Region of Southern Denmark.

**Results:** The final results will be included in the presentation/poster.

**Conclusions:** The study is ongoing.

## 3. FETAL MEDICINE

## Differences in fetal growth trajectories after fresh and frozen embryo transfer, and natural conception: a population‐based register study in Sweden

### **Mårten Ageheim** ^1^, Alkistis Skalkidou^1^, Stavros Iliadis^1^, Eva Bergman^1^, Erik Lampa^2^, Linda Lindström^1^, Anna Sara Öberg^3^

#### ^1^Department of Women's and Children's Health, Uppsala University, Uppsala, Sweden; ^2^Department of Medical Sciences, Uppsala University, Uppsala, Sweden; ^3^Department of Medical Epidemiology and Biostatistics, Karolinska Institutet, Stockholm, Sweden

**Introduction/Purpose:** Fresh as well as frozen embryo transfer (ET) is associated with increased risks for abnormal fetal growth and birthweight. However, intrauterine growth has rarely been studied.

The study aim was to investigate fetal growth trajectories and proportions of small and large for gestational age (SGA, LGA) and macrosomia, in pregnancies after fresh and frozen ET, and natural conception (NC).

**Methods:** We considered all singleton pregnancies resulting in live births from 2009 to 2017 in the Swedish Pregnancy Register (*n* = 503 431). Data were available on 10 996 fresh ET, 6530 frozen ET and 175 290 NC, including linked data from several other national registers. A general least squares model was used to examine the effect of fresh or frozen ET on fetal growth between 17 and 40 gestational weeks (GW) while adjusting for confounders.

**Results:** At 17 GW, fresh and frozen ET had higher fetal weights compared with NC. Frozen ET maintained a growth rate comparable to NC throughout second trimester, thereafter declining. Fetal weight for frozen ET was higher than NC until 40 GW, when no difference was seen. Proportions of LGA and macrosomia were higher compared with NC. Fresh ET showed a continuously decreasing growth rate, with lower fetal weight compared with frozen ET from 25 GW, and to NC from 35 GW, and a higher proportion of SGA compared with NC.

**Conclusions:** This study gives new insights into the differences in fetal growth dynamics between fresh and frozen ET and NC. Clinically relevant differences in proportions of SGA, LGA and macrosomia were observed.

## Proposed cutoff for fetal scalp blood lactate in intrapartum fetal surveillance based on neonatal outcomes: a large prospective observational study

### **Linda Iorizzo** ^1,2^, Ylva Carlsson^3,4^, Christel Johansson^5^, Rim Berggren^3^, Andreas Herbst^2,6^, Mandy Wang^7,8^, Madeleine Leiding^6^, Per‐Erik Isberg^9^, Karl Kristensen^6^, Eva Wiberg‐Itzel^10^ ^,11^, Terry McGee^7^ ^,8^, Nana Wiberg^5,12^

#### ^1^Department of Obstetrics and Gynecology, Helsingborg Hospital, Helsingborg, Sweden; ^2^Department of Clinical Sciences Lund, Lund University, Lund, Sweden; ^3^Department of Obstetrics and Gynecology, Region Västra Götaland, Sahlgrenska University Hospital, Gothenburg, Sweden; ^4^Center of Perinatal Medicine and Health, Institute of Clinical Sciences, Sahlgrenska Academy, Gothenburg, Sweden; ^5^Department of Obstetrics and Gynecology, Ystad lasarett, Ystad, Sweden; ^6^Department of Obstetrics and Gynecology, Skåne University Hospital, Malmø, Sweden; ^7^Department of Obstetrics and Gynecology, Westmead Hospital, Sydney, NSW, Australia; ^8^The University of Sydney, Sydney, NSW, Australia; ^9^Department of Statistics, Lund University, Lund, Sweden; ^10^Department of Obstetrics and Gynecology, Söder Hospital, Stockholm, Sweden; ^11^Karolinska Institute, Stockholm, Sweden; ^12^Department of Clinical Sciences Malmö, Lund University, Lund, Sweden

**Introduction/Purpose:** Determination of lactate in fetal scalp blood (FBS) during labor has been recognized since the 1970s. The internationally accepted cutoff of >4.8 mmol/L to indicate fetal acidosis is exclusive for the point‐of‐care device (POC) LactatePro™, which is no longer in production. The aim of this study was to establish a new cutoff for scalp lactate based on neonatal outcomes with the use of the StatstripLactate®/StatstripXpress® Lactate system, the only POC designed for hospital use.

**Methods:** Observational study. Inclusion criteria: singleton pregnancy, vertex presentation, ≥35+0 weeks of gestation. Based on the optimal correlation between FBS lactate and cord pH/lactate, only cases with ≤25 minutes from FBS to delivery were included in the final calculations. Metabolic acidosis in cord blood defined as pH <7.05 plus BDecf >10 mmol/L and/or lactate >10 mmol/L.

**Results:** From January 2016 to March 2020, laboring women with indication for FBS were prospectively included from seven Swedish and one Australian delivery unit. A total of 3334 women were enrolled, 799 of whom were delivered within 25 minutes. The areas under the receiver operating characteristics curves (AUC) and corresponding optimal cutoff values were as follows; metabolic acidosis AUC 0.87 (95% confidence interval [CI] 0.77–0.97), cutoff 5.7 mmol/L; pH <7.0 AUC 0.83 (95% CI 0.68–0.97), cutoff 4.6 mmol/L; pH <7.05 plus BDecf ≥12 mmol/L AUC 0.97 (95% CI 0.92–1), cutoff 5.8 mmol/L; Apgar score <7 at 5 minutes AUC 0.74 (95% CI 0.63–0.86), cutoff 5.2 mmol/L; and pH <7.10 plus composite neonatal outcome AUC 0.76 (95% CI 0.67–0.85), cutoff 4.8 mmol/L.**Conclusions:** A scalp lactate level <5.2 mmol/L using the StatstripLactate®/StatstripXpress® system will safely rule out fetal metabolic acidosis.

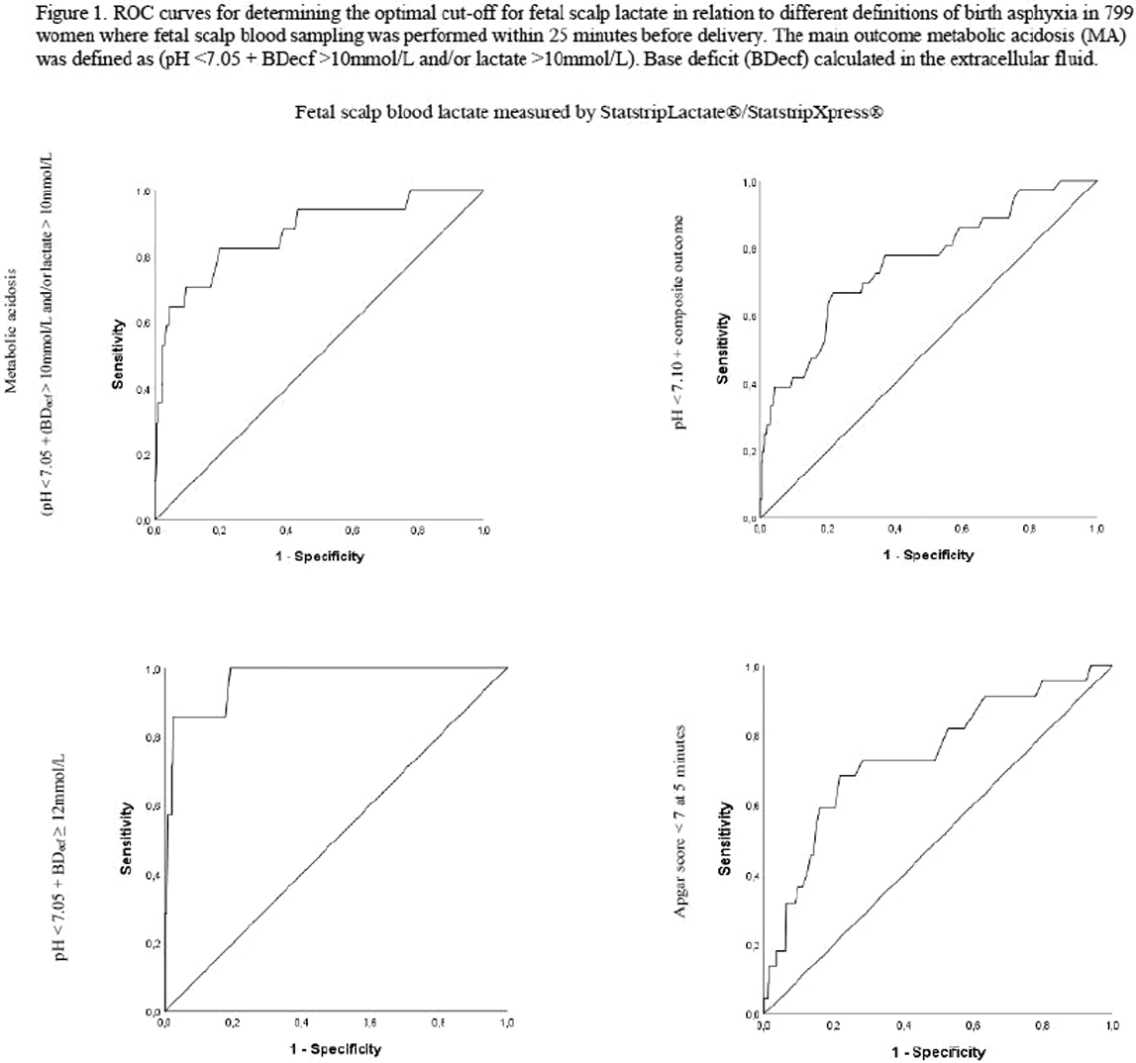

## Longitudinal referance range for the pulsatility index of fetal inferior adrenal arteries

### **Øystein Bergøy** ^1,2^, Ragnar Kvie Sande^1,2^, Torvid Kiserud^2,3^, Jörg Kessler^2,3^, Ingvild Dalen^1,4^

#### ^1^Stavanger University Hospital, Stavanger, Norway; ^2^University of Bergen, Bergen, Norway; ^3^Haukeland University Hospital, Haukeland, Norway; ^4^University of Stavanger, Stavanger, Norway

**Introduction/Purpose:** Animal models have demonstrated increased blood flow to the fetal adrenal glands when placental function is compromised. We found the inferior adrenal artery to be the main arterial blood supply to the human fetal adrenal glands. A normal reference range will enable assessment of the use of this redistribution in the surveillance of growth‐restricted fetuses.

**Methods:** We included women with low‐risk singleton pregnancies at Stavanger University Hospital, Norway, between June 2020 and March 2023. They were examined with Doppler ultrasound approximately once per month after gestational week 20. Pulsatility indices (PI) were determined by Doppler for the inferior adrenal artery. Quantile regression was used to estimate the 5th, 50th (median) and 95th percentile for PI according to gestational age, with non‐linearity handled by applying restricted cubic splines and non‐independence between repeated measurements handled by applying robust estimation of standard errors.

**Results:** We included 149 pregnant women and performed 560 Doppler assessments. In 506 examinations we were able to measure the PI in at least one inferior adrenal artery. Based on our findings, we were able to construct a longitudinal normal reference range for the PI of the fetal inferior adrenal arteries for gestational age 133–287 days.**Conclusions:** Median PI in our material was around 1.0–1.1, indicating low overall impedance. We did not see the marked late‐pregnancy drop in PI previously reported; the median has a bell shape reminiscent of the corresponding curve for the middle cerebral artery.

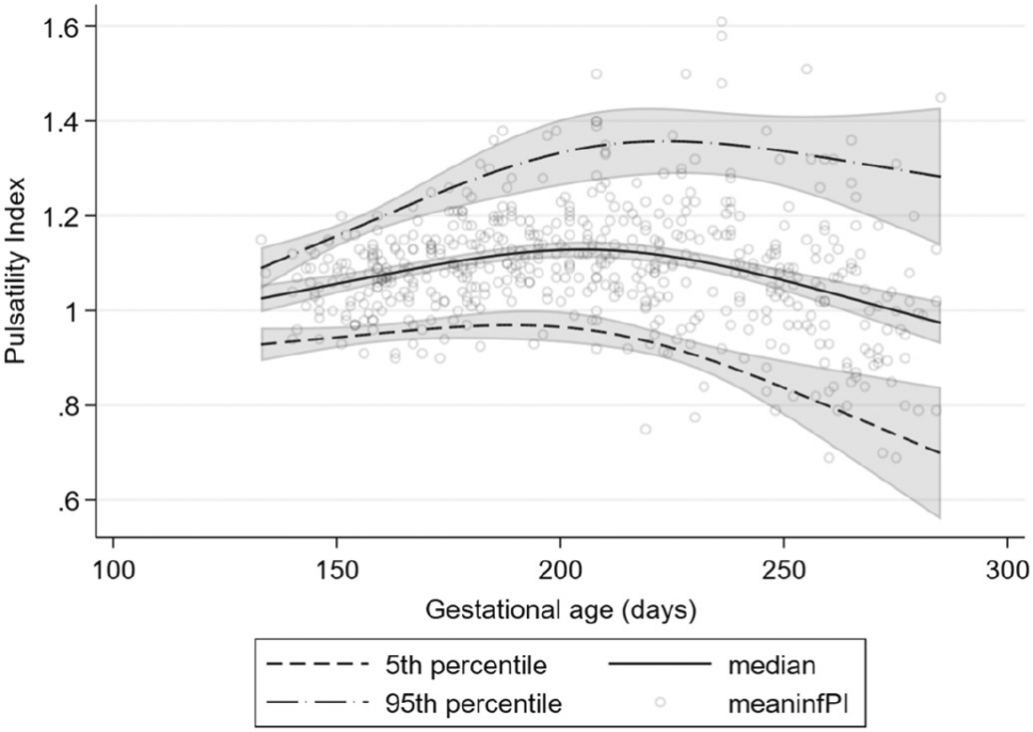

## Pregnant women's informational needs prior to decisions about prenatal screening and diagnosis in Sweden

### **Ellen Ternby** ^1^, Ove Axelsson^1,2^, Susanne Georgsson^3,4^, Charlotta Ingvoldstad Malmgren^5,6,7^

#### ^1^Department of Women's and Children's Health, Uppsala University, Uppsala, Sweden; ^2^Center for Clinical Research Sörmland, Uppsala University, Eskilstuna, Sweden; ^3^The Swedish Red Cross University, Stockholm, Sweden; ^4^Department of Clinical Science, Intervention and Technology, Karolinska Institutet, Stockholm, Sweden; ^5^Department of Clinical Genetics, Karolinska University Hospital, Stockholm, Sweden; ^6^Center for Fetal Medicine, Karolinska University Hospital, Stockholm, Sweden; ^7^Center for Research and Bioethics, CRB, Uppsala University, Uppsala, Sweden

**Introduction/Purpose:** To explore what is important to pregnant women when receiving information about prenatal screening and diagnosis prior to decision‐making regarding prenatal diagnosis.

**Methods:** Q methodology was used for exploring subjective viewpoints. In 2020–2021, 45 pregnant women ranked 50 statements regarding what is important when receiving information about prenatal testing in a fixed quasi‐normal distribution, from “most important” to “least important”. Q factor analysis was performed using Centroid factor extraction and Varimax factor rotation.

**Results:** Three factors were found representing different viewpoints on the subject:

Factor 1: *Stepwise information and decision‐making* – viewing information and decision‐making as a step‐by‐step process, focusing on screening and information about available methods in step 1. Information about conditions screened for, is desired only if screening indicates an anomaly.

Factor 2: *Decision‐making as a continuous process based on couple autonomy* – striving for an informed decision as a couple regarding the complete concept of accepting or declining screening, how to handle the test result and possible effects of conditions screened for.

Factor 3: *As much reliable fact‐based information as early as possible and the importance of individual autonomy in decision‐making* – getting as much non‐directive, good quality information as early as possible from competent healthcare professionals. The self‐autonomous decision is prioritized over involving partners.

**Conclusions:** The different viewpoints highlight the complexities when providing information. Some pregnant women want extensive information from the start, whereas others prefer a step‐by‐step process. As the informational needs differ, women can benefit from individualized rather than standardized information in individual rather than group consultations with healthcare professionals.

## Association between levothyroxine treatment for maternal subclinical hypothyroidism with negative TPOAb and infants’ neurodevelopment

### **Qiongjie Zhou**, Zhekun Zhao, Xiaotian Li

#### Obstetrics and Gynecology Hospital of Fudan University, Fudan, China

**Introduction/Purpose:** Levothyroxine treatment is recommended more for pregnant women with subclinical hypothyroidism and positive thyroid peroxidase antibodies (SCH‐TPOAb^+^) than those with negative SCH‐TPOAb. This study was aimed to evaluate the association between levothyroxine treatment for SCH with different TPOAb status and infant neurodevelopment.

**Methods:** In this secondary data analysis of a Chinese prospective cohort, pregnant women diagnosed with negative or positive SCH‐TPOAb at first antenatal visit were enrolled. Hypothyroid participants diagnosed before conception were excluded. Primary outcome was offspring's neurodevelopment at 1, 3, 6 and 12 months of age, assessed by the Gesell Development Diagnosis Scale (GDDS). Subgroup analyses stratified by maternal thyroid stimulating hormone (TSH) levels, maternal free levothyroxine (T_4_) levels, and anti‐thyroglobulin antibody (TgAb) status at enrollment were also conducted. Sensitivity analyses where participants receiving no treatment at first antenatal visit were excluded.

**Results:** From January 2012 to December 2013, a total of 171 participants were enrolled, including 116 SCH‐TPOAb^–^ and 55 SCH‐TPOAb^+^. Compared with those women with positive SCH‐TPOAb, women with negative SCH‐TPOAb demonstrated lower TSH levels at first antenatal visit and at 12–16+6 gestational weeks, and unexpectedly higher TSH levels at 30 to 34+6 gestational weeks and delivery (*P* < 0.05). Infants of the SCH‐TPOAb− group displayed a lower neurodevelopmental score at 1 year old than those of the SCH‐TPOAb^+^ group (adjusted *P* < 0.05). These results were confirmed in subgroup analyses and sensitivity analyses (*P* < 0.05).

**Conclusions:** Infants born of SCH‐TPOAb− mothers demonstrated a slightly lower neurodevelopmental score at 1 year old than those of SCH‐TPOAb^+^ mothers, which might be associated with the less satisfactory thyroid function control of pregnant women with negative SCH‐TPOAb.

## Can high maternal vitamin D levels positively affect prenatal neurodevelopment in obese pregnancies?

### **Caroline Sylvest Worm** ^1,2^, Anna Louise Vestergaard^1,2,3^, Matilde Kanstrup Andersen^1,2^, Krista Bossow^2^, Agnete Larsen^2,4^, Pinar Bor^1,3,4^

#### ^1^Department of Obstetrics & Gynecology, Randers Regional Hospital, Randers, Denmark; ^2^Department of Biomedicine, Aarhus University, Aarhus, Denmark; ^3^Department of Clinical Medicine, Aarhus University, Aarhus, Denmark; ^4^Shared senior authorship

**Introduction/Purpose:** Overweight and obesity among pregnant women is a global health problem associated with neurodevelopmental, metabolic and cardiovascular health issues in their children. Maternal overweight/obesity increases macrophage presence in the placenta, indicating increased placental inflammation; animal studies show that placental inflammation may cause adverse effects in the offspring. During fetal development, the placenta supports brain development by supplying neurotrophic signals including brain‐derived neurotrophic factor (BDNF) and inflammatory cytokines.

Vitamin D plays an important role in the function of the immune system and in brain development. Notably, vitamin D deficiency is common among pregnant women worldwide.

This study aims to investigate whether obesity and high vitamin D serum levels during pregnancy affect prenatal neurodevelopment as determined by the inflammatory status and the BDNF levels of the placenta and umbilical cord blood.

**Methods:** Women are recruited at Randers Regional Hospital as part of a large randomized clinical trial investigating the effect of increased vitamin D supplement during pregnancy (10 μg vs 90 μg vitamin D supplement). Umbilical cord blood samples and placental samples are collected within 5 hours after birth. Gene and protein expression of pro‐inflammatory cytokines and BDNF will be analyzed with qPCR and ELISA.

**Results:** The study is ongoing.

Primary outcome: Neurotrophic levels in umbilical cord blood.

Secondary outcome: Inflammatory status in placental tissue.

**Conclusions:** We expect that this study will provide a new insight into how maternal obesity affects the placenta and whether increased vitamin D supplementation during pregnancy can ameliorate such adverse effects.

## Placental volume measurements using tracked 2D ultrasound and artificial intelligence

### **Karianne Sagberg** ^1,2^, Torgrim Lie^3^, Helene Fjeldvik Peterson^1,2^, Vigdis Hillestad^1,4^, Anne Eskild^1,2^, Lars Eirik Bø^3^

#### ^1^Department of Obstetrics and Gynecology, Akershus University Hospital, Lørenskog, Norway; ^2^Institute of Clinical Medicine, University of Oslo, Oslo, Norway; ^3^Department of Health Research, SINTEF Digital, Trondheim, Norway; ^4^Department of Diagnostic Imaging, Akershus University Hospital, Lørenskog, Norway

**Introduction/Purpose:** Placental volume measurements can potentially help identify high‐risk pregnancies. However, it is difficult to capture the entire placenta in a single 3D ultrasound image. We aimed to develop a new automated ultrasound method for placental volume measurement that allows the placenta to be imaged from multiple angels, using 2D ultrasound with position tracking and artificial intelligence in the form of a convolutional neural network (CNN) for automatic image segmentation.

**Methods:** Tracked 2D ultrasound images of the placenta were acquired from 43 pregnant women at gestational week 27. We manually segmented the placenta in 2583 images and used the images from 38 placentas to train a CNN for automatic segmentation. The CNN was then applied to the images from the remaining 15 placentas, and the results were combined with tracking data to generate a 3D model and calculate the placental volume. The volumes of these 15 placentas were also estimated from manually segmented 2D and 3D ultrasound images. The methods were compared with MRI.

**Results:** The CNN achieved an F1‐score of 0.84. The correlation with MRI‐based placental volume measurements was similar for automatically segmented (intra‐class correlation [ICC] 0.64, 95% confidence interval [CI] 0.21–0.86), manually segmented (ICC 0.66, 95% CI 0.24–0.87) and 3D (ICC 0.63, 95% CI 0.20–0.86) ultrasound. Ultrasound measured smaller volumes than MRI but this systematic error was less pronounced for the methods based on 2D ultrasound, probably because more placental tissue was captured in the images.

**Conclusions:** Placental volume can be automatically measured using tracked 2D ultrasound and a trained CNN. The method may be further improved.

## Protocol for 20 years’ follow‐up after a randomized controlled trial on Mediterranean diet in pregnancy

### **Hannibal Troensegaard** ^1^, Per Ole Iversen^1,2^, Kirsten B. Holven^1,3^, Kjetil Retterstøl^1,4^

#### ^1^Department of Nutrition, Institute of Basic Medical Sciences, University of Oslo, Oslo, Norway; ^2^Department of Hematology, Oslo University Hospital, Oslo, Norway; ^3^Norwegian National Advisory Unit on Familial Hypercholesterolemia, Oslo University Hospital, Oslo, Norway; ^4^The Lipid Clinic, Department of Medicine, Oslo University Hospital, Oslo, Norway

**Introduction/Purpose:** Inadequate maternal diet during pregnancy can impair offspring health and may increase the risk of cardiovascular disease later in life. The purpose of the proposed study is to assess long‐term risk factors for cardiovascular disease among mothers and their offspring 20 years after they participated in a Mediterranean diet intervention trial during pregnancy.

**Methods:** The “Cardiovascular Risk Reduction Diet In Pregnancy” (CARRDIP) study was a randomized controlled trial performed in 1999–2001. Participants were randomized to adhere to either a Mediterranean diet or a control diet during pregnancy.

The present study will focus on three main categories in mother and offspring of the original CARRDIP study: (1) metabolic and inflammatory status, (2) clinical status and medical history and (3) body composition and cardiovascular risk status.

**Results:** The mother‐offspring pairs from the original CARRDIP study will be invited to a clinical examination and blood sample collection. Mother–offspring pairs (*n* = 269) from the original CARRDIP study had complete datasets according to the study‐protocol.

An extensive amount of data from these mothers during pregnancy and their offspring in the neonatal period have been collected and will be used in comparative analyses with data to be collected in the CARRDIP follow‐up study.

**Conclusions:** Previous studies investigating the effects of nutrition during pregnancy on offspring health have been observational, animal studies or short‐term randomized controlled trials.

This project aims to study the long‐term effects of dietary intervention during pregnancy on offspring cardiovascular risk markers.

## The importance of antenatal identification of small‐for‐gestational‐age fetuses

### **Emma Hertting** ^1,2^, Pelle G. Lindqvist^1,2^, Lotta Herling^3,4^, Eva Wiberg‐Itzel^1^ ^,2^

#### ^1^Department of Clinical Science and Education, Karolinska Institutet, Södersjukhuset, Stockholm, Sweden; ^2^Department of Obstetrics and Gynecology, Södersjukhuset, Stockholm, Sweden; ^3^Center for Fetal Medicine, Karolinska University Hospital, Stockholm, Sweden; ^4^Department of Clinical Science, Intervention and Technology, Karolinska Institutet, Stockholm, Sweden

**Introduction/Purpose:** To investigate the association between antenatal identification of small‐for‐gestational‐age fetuses and adverse perinatal and childhood outcome, in a setting using risk screening for identification.

**Methods:** A register‐based study of all newborns in the Stockholm region in 2014 and 2017. Adverse outcomes for newborns non‐identified and identified as small for gestational age in fetal life were compared using logistic regression models.

Main outcome measures: Severe *adverse outcome*, a composite outcome defined as at least one of the following – *Stillbirth, Severe newborn distress* (Apgar score <4 at 5 minutes or pH <7 or resuscitation activities <10 minutes) *Severe neonatal outcome* (hypoxic ischemic encephalopathy grade 2–3 or necrotizing enterocolitis or neonatal seizures or intraventricular hemorrhage grade 3–4 or bronchopulmonary disease or death at <1 year), *Severe childhood outcome* (cognitive impairment or motor impairment or cerebral palsy or hearing impairment or visual impairment or death at 1–3 years). Each subgroup of outcomes was also analyzed separately.

**Results:** There was a moderately increased risk for adverse outcome in non‐identified compared with identified small‐for‐gestational‐age fetuses, using the complete composite outcome (adjusted odds ratio [aOR] 1.36, 95% confidence interval [CI] 1.03–1.78). Studying subgroups of outcomes, risk of stillbirth and risk of severe newborn distress were significantly increased for non‐identified small‐for‐gestational‐age fetuses (aOR 7.72, 95% CI 3.95–15.10 and aOR1.65, 95% CI 1.18–2.29, respectively) but not risk for severe neonatal and childhood outcome.

**Conclusions:** Risk screening to identify small‐for‐gestational‐age fetuses at risk is insufficient to prevent stillbirth.

## Accuracy and precision of sonographic fetal weight estimation in Sweden

### **Linda Lindström** ^1,2^, Sven Cnattingius^1^, Ove Axelsson^2,3^, Michaela Granfors^1,4^

#### ^1^Division of Clinical Epidemiology, Department of Medicine (Solna), Karolinska Institutet, Stockholm, Sweden; ^2^Department of Women's and Children's Health, Uppsala University, Uppsala, Sweden; ^3^Center for Clinical Research Sörmland, Uppsala University, Eskilstuna, Sweden; ^4^Department of Women's Health, Division of Obstetrics, Karolinska University Hospital, Stockholm, Sweden

**Introduction/Purpose:** Fetal growth assessment is an essential part of modern obstetric care. The Persson and Weldner formula for estimated fetal weight (EFW) is univerally used in Sweden. The objective was to evaluate accuracy and precision of this formula and to compare it with Hadlock's second formula and Shepard's formula.

**Methods:** All 31 521 singleton pregnancies derived from the Pregnancy Register between 2014 and 2021 were delivered at 22+0 weeks or later, with an ultrasound EFW performed within 2 days before delivery.

Bland–Altman analysis, mean percentage error (MPE), proportion of estimates within ±10% of birthweight, and proportion with under‐ and overestimated weight were calculated according to the formulae by Persson and Weldner, Hadlock 2 and Shepard. Last, calculations were made after stratification into small, appropriate and large for gestational age (SGA, AGA and LGA), and gestational age.

**Results:** Using the Persson and Weldner formula, MPE was −2.7 (standard deviation [SD] 8.9) and the proportion of EFW within ±10% from actual birthweight was 76.0%. MPE was largest for severe SGA (<3rd percentile, −5.4) and for the most preterm fetuses (<24 weeks, −5.4). For Hadlock 2 and Shepard's formulae, MPE were 3.9 (SD 8.9) and 3.4 (SD 9.7), and the proportions of EFW within ±10% were 69.4% and 67.1%. MPE was largest for severe LGA (>97th percentile), 7.6 and 9.4.

**Conclusions:** The Persson and Weldner formula is generally accurate for fetal weight estimation, and is as good or better than Hadlock 2 and Shepard's formulae. The precision is lowest in extreme preterm and SGA fetuses, which is of importance in clinical decision making.

## 4. REPRODUCTION

## Fertility counseling and fertility preservation among early‐onset female cancer patients – a Finnish registry‐based study

### **Johanna Melin** ^1,2^, Elina Holopainen^1^, Hanna Savolainen‐Peltonen^1^

#### ^1^Department of Obstetrics and Gynecology, University of Helsinki, Helsinki University Hospital, Helsinki, Finland; ^2^Monash Centre for Health Research and Implementation, School of Public Health and Preventive Medicine, Monash University, Melbourne, Australia

**Introduction/Purpose:** According to recent recommendations, clinicians should inform cancer patients as early as possible about the potential impact of cancer treatment on their fertility. There are limited published data on fertility counseling (FC), fertility preservation (FP) and fertility preservation cycle outcomes in female cancer patients but studies suggest that cancer patients have a lower or similar number of oocytes retrieved compared with age‐matched comparisons.

**Methods:** In this register‐based cohort study we identified 192 female cancer patients who received FC at the age of 16–42 years, between 2011 and 2019. For each patient, we sampled three female comparisons, which were matched by age at IVF treatment cycle, calendar time, number of previous pregnancies and smoking. The primary outcome analyzed was the number of oocytes retrieved and secondary outcomes included number of stimulation days, total gonadotropin dose and number of frozen embryos.

**Results:** In FP treatment cycles the total gonadotropin dose used was higher among cancer patients than female comparisons (2243 ± 963 IU vs 1679 ± 765 IU, *P* < 0.001). The number of oocytes retrieved (13.9 ± 7.7 vs 12.0 ± 6.5, *P* = 0.04) and frozen embryos (4.3 ± 3.2 vs 2.8 ± 2.8, *P* = 0.01) was also significantly higher among cancer patients. The number of stimulation days was similar in both groups (10.0 ± 2.7 vs 9.9 ± 1.65, *P* = 0.41).

**Conclusions:** A good ovarian response can be achieved in female cancer patients but this might require increased doses of gonadotropins. Compared with previous results, our study mirrors a more active approach among clinicians towards offering FP for cancer patients during the more recent years.

## Miscarriage treatment‐related adverse events in Finland: results of a nationwide registry study 1998–2016

### **Nea Helle** ^1^, Maarit Niinimäki^2,3,4^, Tomi Seppälä^5^, Anna But^6^, Mika Gissler^7,8,9^, Oskari Heikinheimo^1^, Maarit Mentula^1^

#### ^1^Department of Obstetrics and Gynecology, University of Helsinki, and Helsinki University Hospital, Helsinki, Finland; ^2^Department of Obstetrics and Gynecology, Oulu University Hospital, Oulu, Finland; ^3^ PEDEGO Research Unit, University of Oulu, Oulu, Finland; ^4^Medical Research Center Oulu (MRC Oulu), University of Oulu, Oulu, Finland; ^5^Department of Information and Service Management, Aalto University, Espoo, Finland, and Business School, University of Eastern Finland; ^6^Faculty of Medicine, Department of Public Health, Clinicum, University of Helsinki, Helsinki, Finland; ^7^Finnish Institute for Health and Welfare (THL), Helsinki, Finland; ^8^Departments of Molecular Medicine and Surgery, and Neurobiology, Karolinska Institute, Stockholm, Sweden; ^9^Academic Primary Health Care Center, Region Stockholm, Stockholm, Sweden

**Introduction/Purpose:** Treatment practices of early miscarriage have changed from mainly surgical to medical management during recent decades. We evaluated the rate of miscarriage treatment‐related adverse events according to different treatment options (surgical vs non‐surgical) for missed abortion and blighted ovum in a large national study.

**Methods:** All women with missed abortion (*n* = 45 758) or blighted ovum (*n* = 23 835) during 1998–2016 were identified from Finnish registers. Retained products of conception and/or vaginal bleeding requiring surgical treatment, infection, secondary surgery for any reason, procedure‐related adverse events, and severe adverse events (eg thromboembolic disorders) within 42 days after the initial admission were considered adverse events.

**Results:** Mean age was 30.9 years (SD 6.4) and 53.9% of women were primigravid. In missed abortion, at least one adverse event was detected in 6.9% after surgical and 22.3% after non‐surgical treatment (*P* < 0.001). For blighted ovum, the corresponding figures were 5.5% and 24.0% (*P* < 0.001). In missed abortion, surgical treatment was followed by infection in 2.9% compared with 3.6% (*P* < 0.001) in non‐surgical treatment; there were retained products of conception and/or vaginal bleeding in 1.9% and 4.6% (*P* < 0.001), respectively. For blighted ovum, the corresponding figures were 2.2% and 2.4% (*P* = 0.46); 1.3% and 3.2% (*P* < 0.001). Severe adverse events were rare after surgical and non‐surgical treatment (missed abortion 0.2% vs 0.3%, *P* = 0.19; blighted ovum 0.3% vs 0.4%, *P* = 0.63).**Conclusions:** Severe adverse events are rare in miscarriages in both treatment options. However, after non‐surgical treatment, retained products of conception were more common in both types of miscarriages and infections in missed abortion.

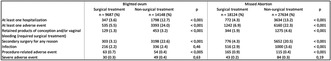

## Born into Life – a longitudinal study before conception, during fetal life and in the first years of life

### **Ellika Andolf** ^1^, Catarina Almqvist^2,3^, Göran Pershagen^4^, Anna Hedman^3^

#### ^1^Department of Clinical Sciences, Karolinska Institutet, Danderyd University Hospital, Stockholm, Sweden; ^2^Karolinska University Hospital, Stockholm, Sweden; ^3^Department of Medical Epidemiology and Biostatistics, Karolinska Institutet, Stockholm, Sweden; ^4^Institute of Environmental Medicine, Karolinska Institutet, Stockholm, Sweden

**Introduction/Purpose:** We know that conditions before conception, during fetal life and in the first years of life affect growth as well as diseases later in life.

**Methods:** LifeGene is a project designed to build a resource for research in all medical disciplines. Participants and family members between 18 and 45 years of age were recruited from the Stockholm area. Physical measurements, hearing, lung function and questionnaire data are available from 52 107 participants. Blood, serum and urine from about 30 000 are stored in the Karolinska Institutet Biobank. The study participants in the Born into Life study were recruited from participants in the LifeGene study who became pregnant. The women were examined on two occasions during pregnancy (questionnaires, saliva, blood and stool samples) and in connection with childbirth (maternal blood at admission and 3 days postpartum, cord blood, four samples from the maternal part of placenta and four from the fetal frozen directly, another sample prepared with formaldehyde). Some study participants had partners who participated in the LifeGene study. Their children were examined (questionnaires, height and weight, blood sampling, stool and saliva) at 6, 12 and 24 months of age and at 5–6 years of follow‐up when lung function testing was also performed.

**Results:** DNA, epigenetics, proteomics, metabolomics and salivary cortisol have been analyzed. Several articles have been published from this cohort: LifeGene.se, Born into Life | Karolinska Institutet (ki.se).

**Conclusions:** We welcome researchers to new innovative collaborations on research issuses using data from the Born into Life cohort. Since we also have data from before pregnancy this biobank is unique.

## Fertility subsequent to methotrexate and salpingectomy treatment of first ectopic pregnancy

### **Mathias Fagervik** ^1^, Cecilie Therese Hagemann^1,2^, Finn Egil Skjeldestad^3^

#### ^1^Department of Clinical and Molecular Medicine Faculty of Medicine and Health Sciences, NTNU, Trondheim, Norway; ^2^Department of Obstetrics and Gynecology, St. Olav's Hospital, Trondheim, Norway; ^3^Department of Community Medicine, Faculty of Health Sciences, UiT The Artic University of Norway, Tromsø, Norway

**Introduction/Purpose:** Little is known about the need for infertility examinations and outcome of infertility treatment among women with a diagnosis of ectopic pregnancy (EP). In this study we assess fertility after methotrexate (MTX) and salpingectomy (SE) treatment after first spontaneously conceived EP.

**Methods:** The study population comprised women admitted to the Department of Gynecology and Obstetrics, St. Olav's Hospital, Trondheim, Norway, from January 2005 through December 2014, with follow‐up through December 2021. Among 263 women eligible for analysis, 25 women refused study participation and 27 women had no pregnancy plans. After exclusion of 11 women who had expectant management, and four women who had tubotomy, and two women with ovarian and one woman with cornual pregnancy, 37 women with MTX treatment and 186 women with partial or total SE remained for analysis.

**Results:** Baseline characteristics (age and parity) were alike between the MTX and SE groups. First reproductive event after index pregnancy revealed 70% with an intrauterine pregnancy (IUP), 5% with repeat EP, 25% with no pregnancy, with no difference across treatment groups. Overall, 16% (95% confidence interval [CI] 14.1–18.0) of the women sought medical attention for infertility. We observed high birthrates among both fertile (83%–84%) and infertile women (74%–75%) (after IVF/ICSI treatment) in both treatment groups.

**Conclusions:** We found high birth rates among infertile and fertile women after first spontaneously conceived EP in both treatment groups. We encourage researchers to report separate fertility outcomes for fertile and infertile women after first spontaneously conceived EP, as infertile patients give an important contribution to assessment of births among EP patients.

## Hyperandrogenic symptoms are a persistent suffering in midlife women with PCOS: a prospective cohort study in Sweden

### **Sofia Persson** ^1^, Kumari A. Ubhayasekera^2^, Jonas Bergquist^2^, Sahruh Turkmen^3^, Inger Sundström Poromaa^1^, Evangelia Elenis^1^

#### ^1^Department of Women's and Children's Health, Uppsala University Hospital, Uppsala, Sweden; ^2^Department of Chemistry ‐ BMC, Analytical Chemistry and Neurochemistry, Uppsala University, Uppsala, Sweden; ^3^Department of Clinical Sciences, Obstetrics and Gynecology, Sundsvalls Research Unit, Umeå University, Umeå, Sweden

**Introduction/Purpose:** Polycystic ovary syndrome (PCOS) is a common endocrine disorder among women, and the majority suffer from hyperandrogenism. Hyperandrogenism causes psychological morbidity and impaired quality of life in women with PCOS during the reproductive years, but data on prevalence and impact during midlife are lacking. Thus, this study aimed to address whether hyperandrogenism persists into midlife and, if so, what impact it has on quality of life.

**Methods:** To answer this question, we performed a multicenter prospective cohort study where we included women already diagnosed with PCOS who had reached the age of 45 years or more as well as age‐matched controls. All participants underwent a physical exam, structured medical interview, biochemical testing and filled out self‐assessment questionnaires.

**Results:** More than 40% of the women with PCOS and 82% of those who presented with the hyperandrogenic phenotype at the diagnostic work‐up still suffered from hirsutism. Circulating testosterone levels were similar between women with PCOS and controls, but free androgen index was higher in women with PCOS, independent of weight. Women with hyperandrogenic PCOS expressed persisting concerns regarding hirsutism at the follow‐up assessment.

**Conclusions:** Women with PCOS who present with hyperandrogenic symptoms at the time they are diagnosed with PCOS have a higher risk of persistent androgenic symptoms and impaired quality of life in midlife.

## Male hypogonadism and the risk of pre‐diabetes and type 2 diabetes from the age of 31 to 46 years: a population‐based follow‐up study

### **Amanda Tuomisto** ^1,2^, Pekka Pinola^1,2^, Hannu Martikainen^1,2^, Juha Auvinen^2,3^, Juha S. Tapanainen^4^, Maarit Niinimäki^1,2^, Laure C. Morin‐Papunen^1,2^

#### ^1^Department of Obstetrics and Gynecology, Oulu University Hospital, Wellbeing Services County of North Ostrobothnia, Oulu, Finland; ^2^Medical Research Center, Research Unit of Clinical Medicine, University of Oulu, Oulu, Finland; ^3^Center for Life Course Health Research, University of Oulu, Oulu, Finland; ^4^Department of Obstetrics and Gynecology, University of Helsinki and Helsinki University Hospital, Helsinki, Finland

**Introduction/Purpose:** In men, cross‐sectional studies have shown associations between hypoandrogenemia (HA) and abnormal glucose metabolism (AGM; pre‐diabetes and type 2 diabetes, T2D). Conversely, one‐third of men with T2D have HA. However, longitudinal studies are scarce, with short follow‐ups.

The purpose of this study was to investigate the association of HA with the risks of AGM in men in a 15‐year follow‐up.

**Methods:** In a population birth cohort, HA (*T* < 12.1 nmol/L, *n* = 132) and normoandrogenic (NA) men (*T* ≥ 12.1 nmol/L, *n* = 2561) at age 31 were followed until age 46. Blood samples were drawn at ages 31 and 46, and 75‐g oral glucose tolerance test (OGTT, *n* = 1409) was performed at age 46.

**Results:** Men with HA at age 31 had a greater BMI at age 46 (mean ± SD: 29.7 ± 5.45 vs 27.06 ± 3.9 kg/m^2^) and a larger waist circumference (103.0 ± 13.7 vs 97.1 ± 11.2 cm; *P* < 0.001 for both) and displayed significantly impaired insulin sensitivity (HOMA‐IR:1.7 ± 0.95 vs 1.4 ± 0.9, *P* = 0.001) and compensatory increased insulin secretion (fasting insulin: 11.8 ± 6.6 vs 10.5 ± 58.6 mU/L, *P* = 0.003; HOMA‐B:100.2 ± 35.2 vs 88.0 ± 33.4, *P* = 0.001). HA men had increased risk of developing AGM by age 46 (odds ratio [OR] 1.78, 95% confidence interval [CI] 1.06–2.99); however, the risk became non‐significant after adjustments for BMI at age 31 or 46. Men in the lowest testosterone quartile at age 31 had a greater risk for AGM compared with men within the highest quartile (*P* < 0.001).

**Conclusions:** Men with HA in early adulthood had an increased risk to develop AGM by age 46, but this risk seemed to be driven by BMI. A longer follow‐up is needed to clarify the long‐lasting metabolic risks linked to HA.

## Pre‐abortion contraceptive planning predicts the rate and type of post‐abortion hormonal contraception use

### **Camilla Jäntti** ^1,2^, Elena Toffol^1^, Jari Haukka^1^, Oskari Heikinheimo^1,3^

#### ^1^University of Helsinki, Helsinki, Finland; ^2^Department of Obstetrics and Gynecology, Hyvinkää Hospital, Hyvinkää, Finland; ^3^Department of Obstetrics and Gynecology, Helsinki University Hospital, Helsinki, Finland

**Introduction/Purpose:** To examine the types and rate of hormonal contraceptive (HC) methods chosen at the time of the abortion, and how they correspond to actual post‐abortion HC use.

**Methods:** We identified 8428 women undergoing induced abortion between July 2017 and December 2018, and their planned post‐abortion contraception using the Finnish national Register of Induced Abortions. The redeemed prescriptions of HC were identified from the Prescription Center. We examined the types and rate of HC methods chosen at the time of the abortion and how they corresponded to actual HC use during a 1‐year follow‐up. Additionally, we assessed factors affecting post‐abortion contraceptive choices using Poisson regression models.

**Results:** At the time of the abortion, 83% (*n* = 7023) of the women were planning to start using HC. Planning any HC pre‐abortion was associated with higher probability to purchase HC after the abortion (incidence rate ratio [IRR] 2.30, 95% confidence interval [CI] 2.07–2.55), especially in the case of vaginal ring (IRR 42.66, 95% CI 33.89–53.71) and contraceptive patch (IRR 156.33, 95% CI 111.31–219.55). The following variables were associated with lower IRR for starting HC after the abortion: highest educational level of bachelor's degree or missing information on education, civil status as married or divorced and history of delivery or induced abortion.

**Conclusions:** Majority of women undergoing abortion plan to use HC for post‐abortion contraception. Planning any HC method at the time of an induced abortion is the most important predictor of starting the method within the year after the abortion.

## Pre‐pregnancy complications – associated factors and wellbeing in early pregnancy. A Swedish cohort study

### **Unnur Gudnadottir** ^1^, Juan Du^1^, Luisa W. Hugerth^1,2^, Lars Engstrand^1^, Ina Schuppe‐Koistinen^1^ ^,3^, Eva Wiberg Itzel^4^, Emma Fransson^1,5^, Nele Brusselaers^1,6,7^

#### ^1^Center for Translational Microbiome Research, Department of Microbiology, Tumor and Cell Biology (MTC), Karolinska Institutet, Solna, Sweden; ^2^Department of Medical Biochemistry and Microbiology, Uppsala University, Uppsala, Sweden; ^3^Science for Life Laboratory, Solna, Sweden; ^4^Department of Clinical Science and Education, Södersjukhuset, Stockholm, Sweden; ^5^Department of Women's and Children's Health, Uppsala University, Uppsala, Sweden; ^6^Global Health Institute, University of Antwerp, Antwerp, Belgium; ^7^Department of Head and Skin, Ghent University, Ghent, Belgium

**Introduction/Purpose:** Many couples experience difficulties becoming pregnant or carrying a pregnancy to term due to unknown causes. We define pre‐pregnancy complications as: prior recurrent pregnancy loss, prior late miscarriages, time to pregnancy over 1 year or use of artificial reproductive technologies. We aim to identify factors associated with pre‐pregnancy complications and poor well‐being in early pregnancy.

**Methods:** Online questionnaire data from 5330 unique pregnancies in Sweden were collected from November 2017 to February 2021. Multivariable logistic regression modeling was used to investigate potential risk factors for pre‐pregnancy complications and differences in early pregnancy symptoms.

**Results:** Pre‐pregnancy complications were identified in 1142 participants (21%).

In additional to previously known risk factors such as high BMI, age and endometriosis, we identified thyroid medication (odds ratio [OR] 2.20, 95% confidence interval [CI] 1.76–2.74), opioids and other strong pain medication (OR 1.97, 95% CI 1.19–3.19), asthma and allergy medication (OR 1.23, 95% CI 1.03–1.46) as risk factors. Different subgroups of pre‐pregnancy complications had unique risk factors.

The groups also experienced different symptoms in early pregnancy. Women that had experienced recurrent pregnancy loss were at higher risk of depression during their current pregnancy (OR 1.71, 95% CI 1.21–2.38). All groups were at higher risk of any complications in their current pregnancy, such as vaginal bleeding (OR 1.72, 95% CI 1.15–2.55).

**Conclusions:** We report one of the largest pregnancy cohorts with a high frequency of pre‐pregnancy complications. Prescribed drug use and bodyweight were the top potentially modifiable risk factors in all groups. Participants that experienced pre‐pregnancy complications also had higher risk of depression and pregnancy problems in early pregnancy.

## Single motherhood by choice in Sweden: unanticipated support needs and financial problems

### **Ylva Af Sandeberg** ^1^, Gunilla Sydsjö^2,3^, Agneta Skoog Svanberg^1^, Claudia Lampic^4,5^, Camilla Steinfelt^6^, Anna‐Karin Lind^7^, Evangelia Elenis^1,8^

#### ^1^Department of Women's and Children's Health, Uppsala University, Uppsala, Sweden; ^2^Department of Biomedical and Clinical Sciences, Division of Children's and Women's Health, Linköping University, Linköping, Sweden; ^3^Department of Obstetrics and Gynecology, Linköping University Hospital, Linköping, Sweden; ^4^Department of Women's and Children's Health, Karolinska Institutet, Stockholm, Sweden; ^5^Department of Psychology, Umeå University, Umeå, Sweden; ^6^Livio Fertilitetscentrum Gärdet, Stockholm, Sweden; ^7^Livio Fertilitetscentrum Göteborg, Göteborg, Sweden; ^8^Reproduction Center, Uppsala University Hospital, Uppsala, Sweden

**Introduction/Purpose:** The aim was to describe single mothers by choice with regard to motives for treatment, support needs and financial situation after the child's birth.

**Methods:** Web‐based survey performed during fall 2022. Study participants were recruited through fertility clinics and social media. Eligible women had been approved for gamete donation treatment as single women in Sweden and had a child aged 0.5–5 years.

**Results:** This survey comprises 260 women (28–48 years old), 61% of whom had had public financed treatment. Most participants held a university/master/PhD degree (87%), their median monthly salary was 3600€ and most owned their home (70%). The dominant motive for seeking treatment as a single woman was absence of a suitable partner to build a family with (82%). Half of women reported pregnancy/delivery complications, with 36% needing more practical support than anticipated from their network, especially during the first 3 months after delivery. Following child birth, half of women (52%) had decreased their employment grade and one in four women reported their current financial status to be worse than anticipated, being unable to manage unexpected big expenses and living on the edge financially. However, the large majority did not regret their decision to become single mothers (93%) and 40% planned to have additional children.

**Conclusions:** Single motherhood is a well‐thought unregrettable action, despite unanticipated financial difficulties and need of practical support from the social network.

## Uterine immune cell profiling of patients with early pregnancy bleeding

### **Ylva Guterstam** ^1,2^, Katharina Schott^3^, Martin Ivarsson^3^, Jonna Bister^3^, Niklas Björkström^3^, Ganesh Acharya^1^, Sebastian Brusell Gidlöf^1,2^

#### ^1^Karolinska Institutet, Department of Clinical Science, Intervention and Technology, Division of Obstetrics and Gynecology, Stockholm, Sweden; ^2^Karolinska University Hospital, Patient Area Gynecology and Reproduction, Stockholm, Sweden; ^3^ Karolinska Institutet, Department of Medicine, Center for Infectious Medicine, Stockholm, Sweden

**Introduction/Purpose:** Immune cell function is important for successful placentation and regulation of the trophoblast invasion. However, the immune cell function in early pregnancy loss is poorly understood. Here, we aimed to characterize the composition, frequency and phenotype of immune cells in vaginal blood in women presenting with first‐trimester bleeding. Furthermore, we correlated serum and saliva proteome to vaginal immune cell phenotype and outcome of pregnancy.

**Methods:** 28 healthy donors were enrolled in the study and donated vaginal blood, matched peripheral blood and saliva at t*w*o timepoints. Flow cytometry was used to determine the phenotype and frequencies of immune cells in vaginal and peripheral blood. Proteome in serum and saliva was analyzed with a proximity extension assay (Olink® Immun‐Oncology Target 96).

**Results:** Vaginal blood immune cells contained all main immune cell lineages including B cells, NK cells, T cells and monocytes. Notably, vaginal blood contained immune cells with tissue residency markers including CD49a. Women who miscarried had a higher frequency of CD49a^+^ vaginal blood‐derived NK cells compared with those who did not miscarry, and this correlated with serum levels of granzyme A and H, CSF1, CAIX and TWEAK. Women in the miscarriage group also had a higher frequency of peripheral blood T cells expressing CD49a.

**Conclusions:** Our study provides novel insight to human reproductive immunology in relation to uneventful pregnancy and miscarriage. Tissue resident NK cells in vaginal blood alone or in combination with serological markers may be a potential prognostic factor in the prediction of pregnancy outcome in women with early pregnancy bleeding.

## Effect of phthalate mixtures on endometrial cell gene expression and function in vitro

### Nadja Visser^1^, Anastasios Damdimopoulos^2^, Eva Davey^1^, Theodora Kunovac Kallak^1^, Susanne Lager^1^, Agne Velthut‐Meikas^3^ , Kristine Roos^3^, Richelle D. Björvang^1,4^, Darja Lavogina^5,6^, Mary Laws^7^, Azazul Chowdhury^8^, Peter Bergsten^8^, Andres Salumets^4,6,9^, Jodi Flaws^7^, Pauliina Damdimopoulou^4^, Matts Olovsson^1^, Ilari Tarvainen^4^

#### ^1^Department of Women's and Children's Health, Uppsala University, Uppsala, Sweden; ^2^Bioinformatics and Expression Analysis Core Facility, Department of Biosciences and Nutrition, Karolinska Institutet, Stockholm, Sweden; ^3^Department of Chemistry and Biotechnology, Tallinn University of Technology, Tallinn, Estonia; ^4^Department of Clinical Science, Intervention and Technology, Karolinska Institute & Karolinska University Hospital, Stockholm, Sweden; ^5^Institute of Chemistry, University of Tartu, Tartu, Estonia; ^6^Competence Center on Health Technologies, Tartu, Estonia; ^7^Department of Comparative Biosciences, University of Illinois, Urbana‐Champaign, IL, USA; ^8^Department of Medical Cell Biology, Uppsala University, Uppsala, Sweden; ^9^Department of Obstetrics and Gynecology, Institute of Clinical Medicine, University of Tartu, Tartu, Estonia

**Introduction/Purpose:** Endocrine‐disrupting chemicals are compounds interfering with hormone balances. Phthalates are a group of EDCs often used as plasticizers found in PVC floors, plastic toys for children and self‐care products such as shampoo and make‐up. Several studies found negative associations with phthalate exposure and fertility outcomes. However, most of these studies have focused on male fertility and fewer on female fertility. In particular, the endometrium is not well studied. Therefore, this project investigates the effects of epidemiologically relevant phthalate mixtures on endometrial cell gene expression and function in vitro.

**Methods:** Stromal and epithelial cells were isolated from hysterectomy biopsies and cultured for phthalate exposure studies. The THESC (immortalized stromal cells) and Ishikawa (epithelial carcinoma) cell lines were also included. Three epidemiologically relevant phthalate mixtures were based on phthalate concentrations measured in urine samples from the Midlife Women's Health Study cohort. We determined effects of phthalate mixtures on endometrial genes and processes using RNA sequencing and PCR. Endometrial cell functioning will be investigated by assays measuring ATP metabolism (Seahorse), protein expression (JESS) and cell adhesion (cell migration).

**Results:** Phthalate mixtures did not negatively affect cell viability and altered processes and genes related to mitochondrial and cytoskeleton function, including *NDUF*, *ATP5/6* and *ACTB*. The results of the on‐going PCR validations and functional assays will be shown in this presentation.

**Conclusions:** Altogether, this project indicates that different phthalate mixtures can regulate gene expression in human endometrial cells in vitro at epidemiologically relevant concentrations. Further studies need to address the possible impact on endometrial biology and fertility in women.

## Factors affecting female sexual well‐being: a 5‐year follow‐up of a randomized clinical trial on post‐abortion contraception

### **Janina Bosas** ^1^, Elena Toffol^1^, Elina Pohjoranta^1,2^, Maarit Mentula^1,2^, Ritva Hurskainen^1^, Satu Suhonen^1^, Oskari Heikinheimo^1,2^

#### ^1^University of Helsinki, Helsinki, Finland; ^2^Department of Obstetrics and Gynecology, Helsinki University Hospital, Helsinki, Finland

**Introduction/Purpose:** Sexual well‐being is associated with general well‐being. In addition, several factors such as overweight, infertility, anxiety and sex hormones play a role. The effects of hormonal contraception remain debated. We characterized the factors associated with sexual well‐being in fertile‐aged women following induced abortion.

**Methods:** A spin‐off study of a randomized clinical trial with a 5‐year follow‐up examining the effects of routine provision of intrauterine contraception as part of abortion care. Sexual well‐being, anxiety and quality of life were assessed annually using validated questionnaires (McCoy Female Sexuality Questionnaire, State–Trait Anxiety Inventory and EuroQoL), along with data on general and reproductive health, and relationship status. Of the 742 women participating in the trial, altogether 290 (39%) provided sufficient follow‐up data and were included in this study.

**Results:** Based on trajectories of McCoy scores across the 5‐year follow‐up, two groups were identified: those with stable and higher (*n* = 223, 76.9%) and those with declining sexual well‐being (*n* = 67, 23.1%). Women in the group of declining sexual well‐being had significantly higher levels of anxiety and lower quality of life at all time points. They also had chronic diseases more often and were less happy in their relationships. No differences were found in the method of contraception when classified as either hormonal vs non‐hormonal, or long‐acting vs short‐acting reversible contraception.

**Conclusions:** Lower anxiety and higher quality of life are associated with stable and higher sexual well‐being. Method of contraception or relationship status is not associated with sexual well‐being during long‐term follow‐up in fertile aged women.

## Higher risk of type 2 diabetes in women with hyperandrogenic polycystic ovary syndrome

### **Sofia Persson** ^1,2^, Evangelia Elenis^1^, Sahruh Turkmen^2,3^, Michael S. Kramer^4,5^, Eu‐Leong Yong^5^, Inger Sundström Poromaa^1^

#### ^1^Department of Women's and Children's Health, Uppsala University Hospital, Uppsala, Sweden; ^2^Department of Obstetrics and Gynecology, Sundsvall County Hospital, Sundsvall, Sweden; ^3^Department of Clinical Sciences, Obstetrics and Gynecology, Sundsvall Research Unit, Umeå University, Umeå, Sweden; ^4^Departments of Epidemiology, Biostatistics and Occupational Health and of Pediatrics, McGill University Faculty of Medicine, Montreal, Quebec, Canada; ^5^Department of Obstetrics and Gynecology, National University Hospital, National University of Singapore, Singapore

**Introduction/Purpose:** The purpose was to assess the risk of type 2 diabetes (T2D) in women with polycystic ovary syndrome (PCOS) in relation to body mass index (BMI) and the hyperandrogenic (HA) PCOS phenotype.

**Methods:** A population‐based cohort study with data from six Swedish national registers. Participants were followed for up to 19 years. All women with an International Statistical Classification of Diseases and Related Health Problems, version 10 (ICD‐10), diagnosis of PCOS, androgen excess or anovulatory infertility born between 1950 and 1999 (*n* = 52 535) were identified in the Patient Register. The HA PCOS phenotype was defined by two filled prescriptions for anti‐androgenic drugs. For each woman with PCOS, five control women (*n* = 254 624) were randomly chosen from the Total Population Register, matched for age and geographic area. The main outcomes were ICD‐10 diagnosis of T2D or prescription of antidiabetic treatment other than metformin.

**Results:** The cumulative incidence rates of T2D were 1.3%, 4.4%, and 14.2% in controls (non‐PCOS women) and women with normoandrogenic (NA) and HA PCOS, respectively. After adjustment for BMI, women with PCOS had a twofold higher rate of T2D than non‐PCOS women (adjusted hazard ratio 2.52, 95% confidence interval [CI] 2.15–2.96). Women with HA PCOS had a higher rate of T2D than those with NA PCOS (adjusted hazard ratio 3.86, 95% CI 3.16–4.72).

**Conclusions:** Polycystic ovary syndrome is an independent risk factor for T2D, even after adjustment for BMI. Women with the HA PCOS phenotype face an even higher risk of T2D than those with the NA PCOS phenotype.

## Minor changes in incidence of primary and secondary infertility across birth cohorts in 1916–1975, but major differences in treatment success

### Finn Egil Skjeldestad

#### Institute of Community Medicine, UiT The Arctic University of Norway, Tromsø, Norway

**Introduction/Purpose:** There have been tremendous achievements in assisted reproductive technologies (ARTs) over the past 50 years. The present study assessed infertility outcomes among women of reproductive age during the rise of ART.

**Methods:** The seventh survey of the Tromsø Study (Tromsø7), 2015–2016 collected among a lot information about health and infertility. Infertility experience was defined as reporting one of more of the following: the clinical definition of infertility (ie infertility period of >1 year), infertility examination, use of ART and the birth of a child conceived during ART. Women with secondary infertility were those who reported infertility experience and had at least one naturally conceived child. Parous women without infertility experience were classified as fertile. The main exposure was birth cohort (10‐year birth cohorts 1916–35, aged 80–98 years, to 1966–75, aged 40–49 years).

**Results:** The prevalence of primary infertility was significantly higher in the 1956–75 cohort (6.0%; 95% confidence interval [CI] 5.4–6.6) than the 1916–55 cohort (3.7%; 95% CI 3.2–4.3). The prevalence of secondary infertility was higher than that of primary infertility across all birth cohorts and was highest for the 1966–75 cohort (10%), with no differences between the other birth cohorts (6%–7%). ART success increased substantially with time, reaching 58% for primary and 46% for secondary infertility in the 1966–75 cohort.

**Conclusions:** There were minor differences in the prevalence of primary and secondary infertility across the 1916–75 cohorts. Advances in ART over the past 50 years comprised 2.0% and 3.3% of population growth in the 1956–65 and 1966–75 cohorts, respectively: a remarkable achievement.

## 5. GENERAL GYNECOLOGY

## Declining effectiveness of treatment for cervical precancer in Denmark

### Tina Randrup^1^, Kathrine Dyhr Lycke^1,2,3^, Maja Lundegaard Iversen^1^, Ane‐Kersti Skaarup Knudsen^1,2,3^, **Anne Hammer** ^1,2,3^

#### ^1^Department of Obstetrics and Gynecology, Gødstrup Hospital, Herning, Denmark; ^2^ NIDO ‐ Center for Research and Education, Gødstrup Hospital, Herning, Denmark; ^3^Department of Clinical Medicine, Aarhus University, Aarhus, Denmark

**Introduction/Purpose:** An effective cervical cancer screening program relies on a high‐quality screening test and a high participation rate. However, it is of utmost importance that women with cervical precancer receive treatment to avoid progression to cancer. Here, we aimed to describe the temporal trends in the proportion of women receiving adequate treatment for cervical precancer in Denmark, overall and stratified by histology, age and residing region.

**Methods:** We conducted a population‐based cross‐sectional study using data from Danish national registers collected for the purpose of another study. We included women aged 18–40 years diagnosed with cervical intraepithelial neoplasia diagnosed during 1995–2021 with a subsequent record of a cone biopsy. We collected information on margin status, age, histology, year of diagnosis and residing region. Results were mainly presented descriptively.

**Results:** A total of 63 720 women had a record of a cone biopsy. Most women had CIN3 (67%) detected in the cone specimen, followed by CIN2 (17%). The proportion of women with negative margins decreased over time, from 66% in 1995–2012 to 54% in 2017–2021, and decreased with increasing severity, from 71% in CIN1 to 50% in CIN3. Women aged 23–30 were more likely to have negative margins as compared with those aged 41–50 (57% vs 46%). The proportion of women with negative margins varied from 42% in the Capital Region to 67% in Central Denmark Region.

**Conclusions:** An increasing number of women receive inadequate treatment for cervical precancer in Denmark; more studies are needed to determine the underlying cause of these findings.

## Risk of progression of cervical intraepithelial neoplasia grade 2 by HPV vaccination status

### Louise Krog^1,2,3^, Kathrine Dyhr Lycke^1,2,3^, Johnny Kahlert^4^, Tina Randrup^1^, Pernille Tine Jensen^3,5^, Anne Rositch^4^, **Anne Hammer** ^1,2,3^

#### ^1^Department of Obstetrics and Gynecology, Gødstrup Hospital, Herning, Denmark; ^2^ NIDO ‐ Center for Research and Education, Gødstrup Hospital, Herning, Denmark; ^3^Department of Clinical Medicine, Aarhus University, Aarhus, Denmark; ^4^Department of Epidemiology, Johns Hopkins Bloomberg School of Public Health, Baltimore, MD, USA; ^5^Department of Obstetrics and Gynecology, Aarhus University Hospital, Aarhus, Denmark

**Introduction/Purpose:** Historically, cervical intraepithelial neoplasia grade 2 (CIN2) has been removed surgically to avoid progression to cancer. However, as surgical treatment is associated with increased risk of preterm birth and regression rates of CIN2 are high, many countries have adopted active surveillance for CIN2. Little is known about which variables may be associated with increased risk of progression. Here, we aimed to investigate the impact of HPV vaccination on risk of progression in women undergoing active surveillance for CIN2.

**Methods:** We conducted a population‐based cohort study using data from Danish national registers. Women were included if they were aged 18–40 and had undergone active surveillance for CIN2 during 2007–2020. Women were defined as vaccinated if they had received ≥1 HPV vaccine dose ≥1 year prior to diagnosis. Women were followed until date of cervical intraepithelial neoplasia grade 3 or worse, cone biopsy, hysterectomy, death, emigration or end of follow‐up. We calculated crude and adjusted risk ratios (RR) using a modified Poisson regression.

**Results:** We included 9203 women with CIN2, 3876 (42.1%) of whom had received ≥1 HPV vaccine dose. Overall, HPV vaccination was not associated with a lower risk of progression (RR 0.98, 95% confidence interval [CI] 0.93–1.04). However, women vaccinated before age 15 (RR 0.67, 95% CI 0.58–0.77) or between age 15 and 17 (RR 0.83, 95% CI 0.72–0.95) had a significantly lower risk of progression compared with unvaccinated women. Adjusted estimates will be presented.

**Conclusions:** HPV vaccination before age 18 is associated with lower risk of progression in women undergoing active surveillance for CIN2.

## Salpingectomy for sterilization (SALSTER), a Swedish register‐based randomized trial: first results on perioperative outcomes

### **Leonidas Magarakis** ^1^, Annika Idahl^2^, Karin Sundfeldt^1,3^, Per Liv^4^, Mathias Pålsson^1^, Annika Strandell^1,3^

#### ^1^Department of Obstetrics and Gynecology, Institute of Clinical Sciences, Sahlgrenska Academy, University of Gothenburg, Gothenburg, Sweden; ^2^Department of Clinical Sciences, Obstetrics and Gynecology, Umeå University, Umeå, Sweden; ^3^Region Västra Götaland, Sahlgrenska University Hospital, Department of Gynecology, Gothenburg, Sweden; ^4^Department of Public Health and Clinical Medicine, Umeå University, Umeå, Sweden

**Introduction/Purpose:** Βased on the hypothesis that οpportunistic salpingectomy reduces the risk of ovarian carcinoma incidence, salpingectomy at sterilization is increasingly being offered. Safety aspects of performing salpingectomy instead of tubal ligation are insufficiently studied.

This trial evaluates the safety, regarding complications and ovarian function, of salpingectomy for sterilization vs tubal ligation at laparoscopy.

**Methods:** In a national, multicenter, register‐based randomized non‐inferiority trial, women <50 years wishing laparoscopic sterilization were included. The Swedish research network SNAKS reaching across all gynecological departments supported SALSTER. The Swedish National Quality Register of Gynecological Surgery (GynOp) was used for inclusion, randomization and follow‐up. GynOp randomly allocated women 1:1 to either salpingectomy or tubal ligation, stratified by age and center. Primary outcomes are complications up to 8 weeks postoperatively, and age at menopause. The non‐inferiority margins were set at 10% for complication rate and 2 years for age at menopause. A statistical analysis plan will be available at ClinicalTrials.gov, where the trial is registered (NCT03860805).

**Results:** Recruitment ended February 2023, when 1030 women had been included by 41 centers over 4 years. According to preliminary data, 519 women were allocated to salpingectomy and 511 to tubal ligation, among which 482 and 496, respectively, will be available for analysis. Baseline characteristics and secondary perioperative outcomes (operative time, bleeding and hospital stay) will be presented at the NFOG Congress.

**Conclusions:** SNAKS and GynOp have facilitated the conduct of the trial. SALSTER will provide high‐quality evidence to aid women in shared decision‐making regarding removal of healthy tubes at sterilization.

## Psychiatric disorders following diagnosis of endometriosis at a young age – a longitudinal cohort study in Finland

### **Elina Rasp** ^1^, Liisu Saavalainen^1^, Mika Gissler^2^, Päivi Härkki^1^, Oskari Heikinheimo^1^, Kristiina Rönö^1^

#### ^1^Department of Obstetrics and Gynecology, University of Helsinki and Helsinki University Hospital, Helsinki, Finland; ^2^Finnish Institute for Health and Welfare (THL), Helsinki, Finland, Region Stockholm, Academic Primary Health Care Center, Stockholm, Sweden; Karolinska Institute, Department of Molecular Medicine and Surgery, Stockholm, Sweden

**Introduction/Purpose:** Endometriosis is linked with higher rates of depression, anxiety and a large spectrum of psychiatric disorders among adults. In this longitudinal cohort study, we investigated the psychiatric disorders following a diagnosis of endometriosis at a young age.

**Methods:** 4532 women receiving a surgically confirmed diagnosis of endometriosis before the age of 25 years in 1987–2012, were retrieved from the Finnish Hospital Discharge Register. The reference cohort (*n* = 9014) was matched by age and municipality on the index date. Follow‐up ended in 2019. The outcomes were psychiatric disorders classified into nine groups requiring psychiatric in‐patient overnight care in 1987–2019, and both in‐ and outpatient care in 1998–2019. Cox regression analysis was performed to investigate hazard ratios (HR) and 95% confidence intervals (95% CI).

**Results:** The mean age of the cohort was 22.5 years (standard deviation (SD) 1.9) at the beginning and 42.3 years (SD 7.2) at the end of the follow‐up. Women receiving the diagnosis of endometriosis at a young age had a higher risk of subsequent diagnosis of depressive, anxiety and stress‐related, bipolar, and other affective, personality, and nonorganic sleeping disorders. The HRs ranged for psychiatric in‐patient overnight care from 1.70 (95% confidence interval [CI] 1.29–2.24) for bipolar to 2.43 (95% CI 2.00–2.96) for depressive disorders. For both in‐ and outpatient diagnoses, the HRs ranged from 1.62 (95% CI 1.24–2.09) for bipolar to 2.09 (95% CI 1.34–3.26) for nonorganic sleeping disorders.

**Conclusions:** Young women with a diagnosis of endometriosis are at higher risk for subsequent psychiatric disorders than their peers.

## Sex hormone‐binding globulin predicts hypercoagulability during hormonal contraceptive use

### **Annina Haverinen** ^1^, Kaisu Luiro^1^, Timea Szanto^2,3^, Marika Kangasniemi^4^, Terhi Piltonen^4^, Riitta Lassila^2,3^, Juha Tapanainen^1^, Oskari Heikinheimo^1^

#### ^1^Department of Obstetrics and Gynecology, University of Helsinki and Helsinki University Hospital, Helsinki, Finland; ^2^Department of Hematology and Comprehensive Cancer Center, Unit of Coagulation Disorders, Helsinki University Hospital, Helsinki, Finland; ^3^Research Program in Systems Oncology, Faculty of Medicine, University of Helsinki, Helsinki, Finland; ^4^Department of Obstetrics and Gynecology, University of Oulu, Oulu University Hospital and Medical Research Center PEDEGO Research Unit, Oulu, Finland

**Introduction/Purpose:** The hypercoagulability state induced by combined oral contraceptive (COC) use is reflected in enhanced in vitro thrombin generation (TG). Increased TG potential during COC use could explain the associated increased risk of venous thromboembolism (VTE). Increases in sex hormone‐binding globulin (SHBG) levels, in turn, reflect the hepatic impact of COCs and have been suggested to predict VTE risk during COC use. COCs containing natural estradiol valerate (EV) impact SHBG and TG less than synthetic ethinylestradiol (EE) does. We investigated the association between changes in SHBG and TG during hormonal contraceptive use.

**Methods:** 56 healthy, normal‐weight (BMI 19–24.9), 18‐ to 35‐year‐old women were randomized to use EV and dienogest (DNG) (EV + DNG), *n* = 20, EE + DNG, *n* = 19, or an active control DNG‐alone (*n* = 17) for 9 weeks. Plasma SHBG levels and in vitro TG were measured before and after the intervention. The association between the change in TG and SHBG levels was analyzed using simple linear regression.

**Results:** The change in SHBG levels correlated with the change in peak thrombin concentrations (Pearson's *r* = 0.76, *P* < 0.001). The overall regres‐sion was statistically significant (*R* ^2^ = 0.57, *F* [df regression, df residual] = 66.3 (1,50), *P* < 0.001), and the change in plasma SHBG levels predicted the change in peak thrombin concentrations (*β* = 0.76, *P* < 0.001).

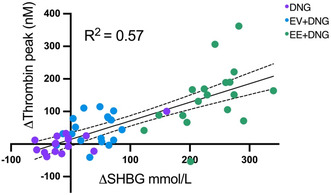

**Conclusions:** During hormonal contraceptive use, increases in plasma SHBG levels predicted in vitro peak thrombin concentrations. This finding suggests that SHBG could be used as a surrogate marker to estimate the net effects of hormonal contraceptives on the coagulation system.

## Data resource profile: women's health study (WENDY) – a protocol of a population‐based study assessing gynecological and metabolic health in women

### **Terhi Piltonen** ^1^, Maria Ohtamaa^1^, Lotta Vuokila^1^, Riikka Arffman^1^, Elisa Korhonen^1^, Minna Männikkö^2^, Laura Huilaja^3^, Suvi‐Päivikki Sinikumpu^3^, Tero Rautio^4^, Katariina Kilpivaara^4^, Jari Jokelainen^2^, Eetu Kiviniemi^2^, Pekka Pinola^1^, Minna Törnävä^5^, Elina Komsi^1^, Marika Kangasniemi^1^, Maria Rajecki^6^, Kaisu Luiro^6^, Jenni Kinnunen^1^, Susanna Savukoski^1^

#### ^1^Department of Obstetrics and Gynecology, Oulu University Hospital, Research Unit of Clinical Medicine, Medical Research Center, University of Oulu, Oulu, Finland; ^2^Northern Finland Birth Cohorts, Arctic Biobank, Infrastructure for Population Studies, Faculty of Medicine, University of Oulu, Oulu, Finland; ^3^Department of Dermatology, Oulu University Hospital, Research Unit of Clinical Medicine, Medical Research Center, University of Oulu, Oulu, Finland; ^4^Department of Surgery, Oulu University Hospital, Research Unit of Translational Medicine, Medical Research Center, University of Oulu, Oulu, Finland; ^5^Health and Social Care, School of Applied Sciences, Tampere, Finland; ^6^Department of Obstetrics and Gynecology, Helsinki University Hospital, University of Helsinki, Helsinki, Finland

**Introduction/Purpose:** Several common gynecological conditions are poorly recognized in healthcare due to lack of knowledge and resources. Moreover, some women are reluctant to report their symptoms due to social or cultural aspects. The Women's health study (WENDY) includes a population sample of 1918 women and explores gynecological and overall health in women in their mid‐thirties.

**Methods:** A majority of the study participants were female members of the Northern Finland Birth Cohort (NFBC) 1986, a population‐based birth cohort originally including 9432 live newborns (4567 girls) in the Northern Finland area between July 1985 and June 1986. Invitations were sent to 4292 women, of whom 1544 participated (36%). The WENDY population was also expanded with a random selection of 1112 women born in the same area between August 1986 and December 1987; of these women, 374 participated (34%).

**Results:** The results will be generated from the collected data with a possibility of data linkage to the Finnish national registers and to the earlier NFBC1986 data collections. The data include a broad clinical examination, including several different measurements by study nurses and gynecologists, biological samples, ultrasound examinations as well as vast range of questions and validated questionnaires on gynecological and reproductive health/history, physical and mental health, quality of life, lifestyle, life situation, health awareness and opinions.

**Conclusions:** WENDY provides a unique and comprehensive data source that can be broadly utilized to gain deeper insight into women's health at reproductive age, gynecological diseases and symptoms and to improve collaborations and women's health worldwide. Collaborators can contact PI at Terhi.Piltonen(at)oulu.fi.

## Trends in the incidence and prevalence of menopausal hormone therapy in Sweden from 2000 to 2021

### **Micaela Sundell** ^1,2^, Jan Brynhildsen^2,3^, Anna‐Clara Spetz Holm^2,4^, Mats Fredrikson^2^, Mikael Hoffmann^5^

#### ^1^Department of Biomedical and Clinical Sciences, Linköping University, Linköping, Sweden; ^2^Department of Obstetrics and Gynecology, Kalmar County Hospital, Kalmar, Sweden; ^3^Department of Obstetrics and Gynecology, School of Medical Sciences, Faculty of Medicine and Health, Örebro University, Örebro, Sweden; ^4^Department of Obstetrics and Gynecology, Linköping University Hospital, Linköping, Sweden; ^5^Department of Health, Medicine and Caring Sciences, Linköping University, Linköping, Sweden

**Introduction/Purpose:** To describe the dynamics of incidence, prevalence and defined daily doses (DDD) of menopausal hormone therapy (MHT) in Sweden during 2000–2021 and to analyze the impact of different run‐in periods on the incidence.

**Methods:** Swedish dispensation data on >4 million women ≥30 years in 2006–2021 was exported from the Swedish eHealth Agency as aggregated patient‐level data; DDD, 1‐year prevalence and incidence of MHT use were studied. The predictive values for incidence representing first‐ever use of MHT were calculated for different run‐in periods, with 120 months as a reference.

**Results:** From 2000 to 2010, the DDD and the prevalence of MHT decreased by over 80% in women aged 50–54 years. From 2018, the use increased at all ages except for the oldest, 65–69 years.

The predictive value for incident users to be true first‐ever users with a run‐in of 18 months was 88% in women aged 50–54 years in 2021. The incidence was stable between 2007 and 2016. From 2017 the incidence increased, being most pronounced for the ages close to menopause.

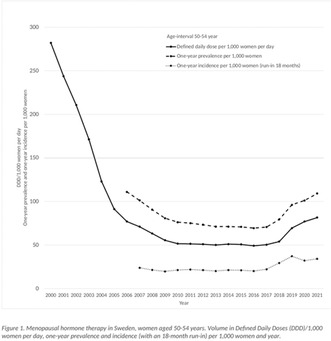

**Conclusions:** The use of MHT decreased significantly after the turn of the century. It has increased since 2017, mainly in the age groups close to menopause. The increase in incidence preceded the prevalence and seems to be a more sensitive measure than prevalence or DDD for detecting early changes in prescription trends of MHT. A run‐in period of 18 months seems to be reliable to identify incident users in the ages close to menopause.

## Altered GABAA receptor function in women with endometriosis: a possible pain‐related mechanism

### **Anton Sandström** ^1,2^, Marie Bixo^1^, Torbjörn Bäckström^1^, Anna Möller^3,4^, Sahruh Turkmen^1,2^

#### ^1^Department of Clinical Sciences, Obstetrics and Gynecology, Umea University, Umea, Sweden; ^2^Department of Obstetrics and Gynecology, Sundsvall County Hospital, Sundsvall, Sweden; ^3^Department of Obstetrics and Gynecology, Stockholm South General Hospital, Stockholm, Sweden; ^4^Department of Clinical Science and Education, Karolinska Institutet, Stockholm, Sweden

**Introduction/Purpose:** The mechanism underlying endometriosis‐related pain remains poorly understood. Previous studies have indicated that γ‐aminobutyric acid (GABA) type A (GABA_A_) receptors and GABAergic substances (eg endogenous neurosteroids) play important mechanistic roles in various pain conditions. Our primary objective was to compare GABA_A_ receptor function between women with endometriosis and healthy controls by performing a challenge test with diazepam, a GABA_A_ receptor agonist, using the change in saccadic eye velocity (ΔSEV) as the main outcome. The secondary objective was to investigate the relationship between GABA_A_ receptor function and serum levels of allopregnanolone, an endogenous positive modulator of the GABA_A_ receptor, in the participating women.

**Methods:** 15 women with pelvic pain and laparoscopically confirmed endometriosis, and 10 healthy, symptom‐free, control women, aged 18–40 years, underwent the diazepam challenge test during the follicular phase of the menstrual cycle. Basal serum allopregnanolone levels were measured prior to diazepam injection.

**Results:** Compared with healthy controls, women with pelvic pain and confirmed endometriosis had a significantly smaller change in SEV after GABA_A_ receptor stimulation with diazepam, indicating lower sensitivity to diazepam. The SEV response was not correlated with the serum allopregnanolone levels.

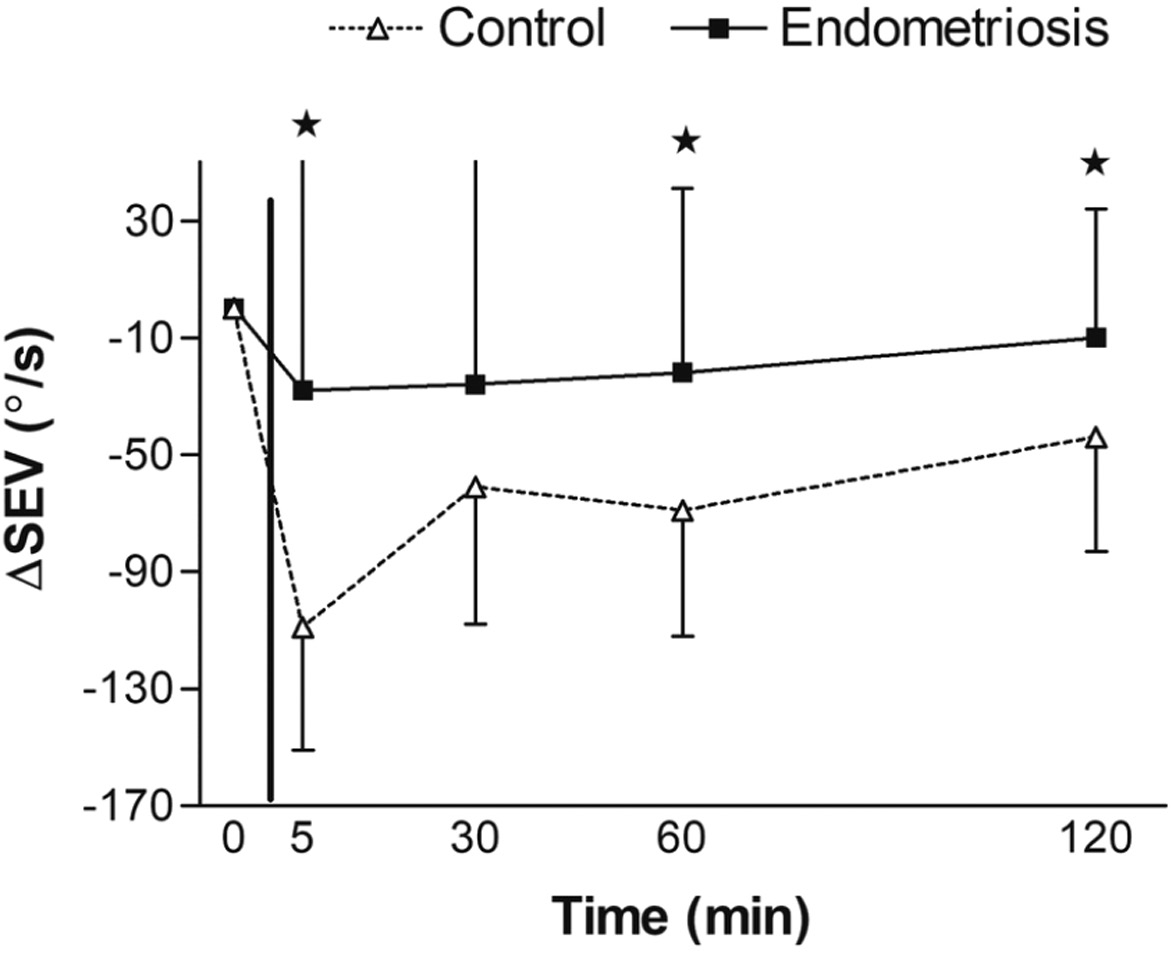

**Conclusions:** Women with painful endometriosis show altered GABA_A_ receptor function, depicted as a muted response to an exogenous GABA_A_ receptor agonist.

## Amsterdam hyperactive pelvic floor scale. Translation and psychometric testing of a Norwegian version among women with pelvic floor dysfunctions

### **Vilde Dons** ^1^, Ingvild Marie Haug Bratlie^1^, Cecilie Therese Hagemann^2,3^, Signe Nilssen Stafne^4^, Siri Spetalen^1^, Susan Saga^5^

#### ^1^Faculty of Medicine and Health Sciences (MH faculty), Norwegian University of Science and Technology (NTNU), Trondheim, Norway; ^2^Department of Clinical and Molecular Medicine (IKOM), Faculty of Medicine and Health Sciences (MH faculty), Norwegian University of Science and Technology (NTNU), Trondheim, Norway; ^3^Department of Obstetrics and Gynecology, St. Olav's Hospital, Trondheim, Norway; ^4^Department of Clinical Services, St. Olav's Hospital, Trondheim, Norway; ^5^Department of Public Health and Nursing (ISM), Faculty of Medicine and Health Sciences (MH faculty), Norwegian University of Science and Technology (NTNU), Trondheim, Norway

**Introduction/Purpose:** A Dutch research group has developed the Amsterdam Hyperactive Pelvic Floor Scale (AHPFS), a 30‐item self‐report questionnaire which intended to cover symptoms related to pelvic floor dysfunctions. The overall aim of this study was to translate AHPFS to Norwegian and assess the validity and reliability in a Norwegian setting of women with hyperactive pelvic floor symptoms.

**Methods:** A group of health care personnel fluent in Dutch and Norwegian translated the original AHPFS into Norwegian. Modifications were made after comments from experts. We performed cognitive interviews with patients. Women were invited to fill out a web‐based version of the translated AHPFS together with some background questions and additional validated questionnaires targeting pelvic floor dysfunction. Content/face validity, construct validity, test–retest reliability/stability and correlation with the already validated questionnaires were performed.

**Results:** Content/face validity demonstrated the questionnaire was perceived as relevant and understandable; some adjustments were made in introduction section and to response categories.

Of 397 women filling in the questionnaire at baseline, 232 scored ≥11 on the AHPFS and were included. Healthy volunteers constituted 28%, patients from St. Olav's Hospital 46%, and from the Vulvodynia/Endometriosis Patient Association 26%. Mean age was 38 years.

An adapted five‐factor structure similar to the original, covering most of the original scales but with only 16 items. There was excellent test–retest stability.

**Conclusions:** Our modified Norwegian version of the AHPFS reduced the number of items from 30 to 16, showed satisfying psychometric properties and could be an adequate instrument for measuring symptoms in patients with a hypertonic pelvic floor.

## Association between preoperative spatial pain extent and postoperative pain after benign hysterectomy with emphasis on pelvic pain

### **Peter Lukas** ^1^, Lena Nilsson^2^, Ninnie Borendal Wodlin^1^, Björn Gerdle^3^, Lars Arendt‐Nielsen^4^ ^,5^, Preben Kjölhede^1^

#### ^1^Department of Obstetrics and Gynecology in Linköping, and Department of Biomedical and Clinical Sciences, Linköping University, Linköping, Sweden; ^2^Department of Anesthesiology and Intensive Care in Linköping, and Department of Biomedical and Clinical Sciences, Linköping University, Linköping, Sweden; ^3^Pain and Rehabilitation Center, and Department of Health, Medicine and Caring Sciences, Linköping University, Linköping, Sweden; ^4^Center for Neuroplasticity and Pain (CNAP), Department of Health Science and Technology, Faculty of Medicine, Aalborg University, Aalborg, Denmark; ^5^Department of Medical Gastroenterology, Mech‐Sense, Aalborg University Hospital, Aalborg, Denmark

**Introduction/Purpose:** The objective of the study was to evaluate the association between patient‐reported spatial pain spread preoperatively and postoperatively, with emphasis on pelvic pain.

**Methods:** A prospective longitudinal observational multicenter study was conducted in five hospitals in the southeast Sweden during 2011 and 2017. In all, 471 women, aged 28–59 years, scheduled for benign hysterectomy, abdominal or vaginal, were enrolled. The Swedish Postoperative Symptom Questionnaire and the Margolis patient pain drawing was used to measure pain, filled in preoperatively and 1 year postoperatively. The Margolis pain drawing outlines the spatial extent of pain in 45 areas of the front and back of the body.

**Results:** Preoperatively, 17.6% reported no pain areas, 7.0% reported pelvic pain only, 31.8% reported other areas of pain besides the pelvis, and 40.3% reported other areas of pain without the pelvis. After 1 year, de novo pelvic pain occurred in 9.1%. None of these occurred in women without pain areas preoperatively. Of the women with only pelvic pain preoperatively, 90.3% were cured of the pain but 16.1% developed pain in other areas outside the pelvis. Of the women with pain in other areas beside the pelvis preoperatively, 82.2% were cured of the pelvic pain and 17.8% had persistent pelvic pain.

**Conclusions:** The preoperative spatial extent of pain seemed to be important for both the outcome of pelvic pain and the development of de novo pelvic pain. This information may be important in the preoperative counseling to achieve a realistic expectation of the hysterectomy.

## Association of miscarriage and mental health – a Finnish nationwide register‐based study

### **Reetta Linnakaari** ^1^, Maarit Niinimäki^2,3^, Maarit Mentula^1^, Oskari Heikinheimo^1^, Tomi Seppälä^4^, Mika Gissler^5,6,7^

#### ^1^Department of Obstetrics and Gynecology, University of Helsinki, and Helsinki University Hospital, Helsinki, Finland; ^2^Department of Obstetrics and Gynecology, Oulu University Hospital, Wellbeing Services County of North Ostrobothnia, Oulu, Finland; ^3^Medical Research Center, Research Unit of Clinical Medicine, University of Oulu, Finland; ^4^Department of Information and Service Management, Aalto University, Espoo, Finland, and Business School, University of Eastern Finland; ^5^Finnish Institute for Health and Welfare (THL), Helsinki, Finland; ^6^Departments of Molecular Medicine and Surgery, and Neurobiology, Karolinska Institute, Stockholm, Sweden; ^7^Academic Primary Health Care Center, Region Stockholm, Stockholm, Sweden

**Introduction/Purpose:** A loss of pregnancy is a potentially traumatic or distressing experience to a woman. Our aim was to study whether the incidence of psychiatric disorders increases during the first year after miscarriage.

**Methods:** We identified 115 554 women, aged 15–49, who had their first pregnancy resulting in miscarriage and received treatment in a public hospital between 2000 and 2015 in Finland from national registers. We investigated the psychiatric diagnoses of these women 2 years before and 1 year after the miscarriage. Division of the diagnoses rested on ICD‐10: psychoactive substance‐use disorders (group F1), schizophrenia, schizotypal and delusional disorders (F2), mood disorders (F3), neurotic, stress‐related and somatoform disorders (F4).

**Results:** Of 96 857 psychiatric diagnostic codes, 59 825 occurred during the 2 years before the miscarriage (29 913 per year) and 36 899 on the day of miscarriage or during the first year after that. The most common types of disorders were mood disorders (43,2% of all diagnoses) and neurotic, stress‐related and somatoform disorders (26.3%). There was a 50.5% annual increase (from 2117 to 3187) of group F1 disorders, a 20.7% increase (2614 to 3155) of group F2 disorders, a 23.0% increase (15 075 to 18 537) of group F3 disorders, and a 34.9% increase (8840 to 11 927) of group F4 disorders after the miscarriage.

**Conclusions:** We found a 23.4% increase in the rate of psychiatric diagnosis during the first year after miscarriage. This indicates that miscarriage is associated with impaired mental health. Further studies are needed to better understand this impairment and how to identify the women in need of support.

## Associations between arterial health and sexual function in late middle‐aged women

### Viivi Virkkunen^1^, Katja Kero^1^, Mari Koivisto^2^, Teemu Niiranen^3,4^, Olli Heinonen^5^, Sari Stenholm^6,7^, **Päivi** **Polo‐Kantola** ^1^

#### ^1^Department of Obstetrics and Gynecology, Turku University Hospital and University of Turku, Turku, Finland; ^2^Department of Biostatistics, University of Turku, Turku, Finland; ^3^Department of Internal Medicine, Turku University Hospital and University of Turku, Turku, Finland; ^4^Department of Public Health Solution, Finnish Institute for Health and Welfare, Helsinki, Finland; ^5^Paavo Nurmi Center & Unit for Health and Physical Activity, University of Turku, Turku, Finland; ^6^Department of Public Health, University of Turku and Turku University Hospital, Turku, Finland; ^7^Center for Population Health Research, University of Turku and Turku University Hospital, Turku, Finland

**Introduction/Purpose:** Female sexual dysfunction is highly common but its etiology and contributive factors are poorly understood. The purpose of this study was to assess the relationship between arterial health and female sexual function in late middle‐aged women.

**Methods:** The cross‐sectional study consisting 117 women (age range 60–64 years) from the Finnish Retirement and Aging study. Arterial health was assessed by measuring pulse wave velocity (PWV), ankle‐brachial index (ABI), blood pressure and pulse pressure. Sexual function was measured with the Female Sexual Function Index (FSFI) questionnaire, which consisted of a total score and five sub‐scores. Associations were examined in multivariable regression analyses, which were adjusted with relationship happiness, menopausal hormone therapy, smoking, alcohol use, body mass index and depressive symptoms.

**Results:** Higher ABI was associated with higher sexual satisfaction (*β* = 2.135, *P* = 0.012). Furthermore, higher diastolic blood pressure was associated with higher total FSFI score (*β* = 0.213, *P* = 0.013) and with higher desire (*β* = 0.020, *P* = 0.008), arousal (*β* = 0.037, *P* = 0.034), satisfaction (*β* = 0.025, *P* = 0.026) and pain (*β* = 0.050, *P* = 0.018) sub‐scores. Additionally, higher pulse pressure was associated with lower orgasm score (*β* = −0.031, *P* = 0.037). No correlations between PWV or systolic blood pressure and sexual function were observed.

**Conclusions:** Better arterial health, measured by ABI, diastolic blood pressure and pulse pressure, were found to be associated with better female sexual function. Our findings suggest the importance of peripheral arterial health for female sexual function.

## Combined oral contraceptive pill compared with no medical treatment in the management of polycystic ovary syndrome: a systematic review

### **Maria Forslund** ^1,2^, Johanna Melin^2,3^, Simon Alesi^2^, Terhi Piltonen^4^, Daniela Romualdi^5^, Chau Thien Tay^2^, Selma Witchel^6^, Alexia Pena^7^, Aya Mousa^2^, Helena Teede^2,8^

#### ^1^Department of Obstetrics and Gynecology, Institute of Clinical Sciences, Sahlgrenska Academy, University of Gothenburg, Gothenburg, Sweden; ^2^Monash Centre for Health Research & Implementation, School of Public Health and Preventive Medicine, Monash University, Melbourne, Australia; ^3^Department of Obstetrics and Gynecology, University of Helsinki, Helsinki University Hospital, Helsinki, Finland; ^4^Department of Obstetrics and Gynecology, Research Unit of Clinical Medicine and Medical Research Center, Oulu University Hospital, University of Oulu, Oulu, Finland; ^5^Department of Obstetrics and Gynecology, Fondazione Policlinico Universitario Agostino Gemelli IRCCS, Italy; ^6^Division of Pediatric Endocrinology, Department of Pediatrics, UPMC Children's Hospital of Pittsburgh, Pittsburgh, PA, USA; ^7^Discipline of Paedriatics, The University of Adelaide and Robinson Research Institute, Australia; ^8^Endocrine and Diabetes Units, Monash Health, Melbourne, Australia

**Introduction/Purpose:** Polycystic ovary syndrome (PCOS) is the most common endocrinopathy in women of reproductive age, and is associated with adverse reproductive, metabolic and psychological outcomes. One common treatment is the combined oral contraceptive pill (COCP). As part of the update of the International Evidence‐Based Guidelines for the Assessment and Management of PCOS, a systematic review was performed to inform evidence‐based recommendations. The objective was to provide guideline developers with the best available evidence on the use of COCP compared with no medical treatment in the management of PCOS.

**Methods:** Studies published before 2017 were captured in a previous systematic review, part of the 2018 guidelines, and were updated here using searches from 2017 to July 2022. Eligibility criteria and outcomes were designed in collaboration with clinical experts, researchers and consumers. The systematic review was performed according to PRISMA guidelines.

**Results:** Four studies were included, three of which had a high risk of bias. No studies could be combined for meta‐analysis regarding any comparison or outcome. COCP treatment improved cycle regularity compared with no medical treatment, with low certainty of evidence. Additional evidence regarding COCP treatment vs no medical treatment (controls/placebo/lifestyle) is currently only available at a very low certainty of evidence.

**Conclusions:** COCP improved cycle regulation with a low certainty of evidence. No other benefits or potential adverse effects were identified with very low certainty of evidence. The COCP is frontline medical treatment based on established efficacy in the broader population on cycle regulation, hirsutism and contraception. However, research in PCOS is seriously lacking.

## Early intervention after rape to prevent post‐traumatic stress disorder and pelvic floor dysfunction – a multicenter randomized control trial

### **Cecilie Therese Hagemann** ^1,2^, Tina Haugen^1,3^, Joar Øveraas Halvorsen^3,4^, Berit Schei^2,5^, Paul Jarle Mork^5^, Oddgeir Friborg^6^

#### ^1^Department of Clinical and Molecular Medicine (IKOM), Faculty of Medicine and Health Sciences (MH faculty), Norwegian University of Science and Technology (NTNU), Trondheim, Norway; ^2^Department of Obstetrics and Gynecology, St. Olav's Hospital, Trondheim, Norway; ^3^Department of Psychiatry, St. Olav's Hospital, Trondheim, Norway; ^4^Department of Psychology, NTNU, Trondheim, Norway; ^5^Department of Public Health and Nursing (ISM), Faculty of Medicine and Health Sciences (MH faculty), NTNU, Trondheim, Norway; ^6^Department of Psychology, Arctic University of Norway, Tromsø, Norway

**Introduction/Purpose:** Rape is a common cause of post‐traumatic stress disorder (PTSD) among women. Several adverse reproductive health consequences, such as sexual dysfunction and chronic pelvic pain are described after sexual assault. PTSD has been found to play a role in the association between experiencing sexual assault and dyspareunia and sexual dysfunction.

Prolonged exposure therapy (PE) is an evidence‐based treatment for PTSD. We have developed a modified PE protocol (mPE), consisting of 4‐weekly 60‐minute sessions, and studies indicate that if implemented early after rape, mPE may prevent the development of PTSD.

The primary objective is to test whether mPE is superior to treatment as usual (TAU) in preventing the development of PTSD. Secondary objectives, and the purpose of this sub‐study, is to assess whether mPE may prevent sexual dysfunction and chronic pelvic pain.

**Methods:** We are conducting a multi‐site randomized control trial in which patients are recruited from three Sexual Assault Centers (SACs) early after rape and randomized to intervention (mPE) or TAU. For the secondary outcomes this study has a prospective cohort design.

Outcomes are assessed through self‐reported questionnaires measuring PTSD, quality of life, chronic pelvic pain, and sexual dysfunction assessed at baseline, at 6 weeks, and 3, 6, and 12 month post‐assault.

**Results:** We will not present results yet.

**Conclusions:** The present trial aims to investigate whether a relatively simple intervention, with a low number of sessions, possible to be performed by those who already perform the SAC follow‐up consultations, is more effective in preventing mental and psychosexual distress after rape than TAU.

## Emotional brain activation in women with premenstrual dysphoric disorder and association to progesterone‐derived neurosteroids

### **Louise Stiernman** ^1^, Manon Dubol^2^, Erika Comasco^2^, Inger Sundström‐Poromaa^2^ , Carl‐Johan Boraxbekk^3,4^, Marie Bixo^1^

#### ^1^Department of Clinical Sciences, Umeå University, Umeå, Sweden; ^2^Department of Womens’ and Children's Health, Uppsala University, Uppsala, Sweden; ^3^Department of Radiation Sciences, Umeå University, Umeå, Sweden; ^4^Umeå Center for Functional Brain Imaging (UFBI), Umeå University, Umeå, Sweden

**Introduction/Purpose:** Premenstrual dysphoric disorder (PMDD) is characterized by severe mood symptoms in the luteal phase of the menstrual cycle. PMDD symptoms are hypothesized to be linked to an altered sensitivity to normal luteal phase levels of allopregnanolone (ALLO), a GABA_A_‐modulating progesterone metabolite. Moreover, the endogenous epimer of ALLO, isoallopregnanolone (ISO), has been shown to alleviate PMDD symptoms through its selective and dose‐dependent antagonism of the ALLO effect. There is preliminary evidence showing altered recruitment of brain regions during emotional processing in PMDD, but whether this is associated to levels of ALLO, ISO or to their relative concentration is unknown.

**Methods:** In the present study, female subjects with PMDD and controls underwent functional magnetic resonance imaging in the mid‐follicular and the late‐luteal phase of the menstrual cycle. Brain responses to emotional stimuli were investigated and related to serum levels of ovarian steroids, the neurosteroids ALLO and ISO, and their ratio ISO/ALLO.

**Results:** Subjects with PMDD exhibited greater activity in brain regions which are part of emotion‐processing networks during the late‐luteal phase of the menstrual cycle. Furthermore, activity in key regions of emotional networks – the parahippocampal gyrus and amygdala – was differentially associated to the ratio of ISO/ALLO levels in PMDD subjects and controls. Specifically, positive relationships between ISO/ALLO levels and brain activity were found in PMDD subjects, whereas the opposite was observed in controls.

**Conclusions:** Female subjects with PMDD show altered emotional brain responses in the late‐luteal phase of the menstrual cycle which may be related to an abnormal response to physiological levels of GABA_A_‐active neurosteroids.

## Hyperactive pelvic floor in women. a survey among patients and healthy volunteers

### **Siri Spetalen** ^1^, Cecilie Therese Hagemann^2,3^, Signe Nilssen Stafne^4^, Ingvild Marie Haug Bratlie^1^, Vilde Dons^1^, Susan Saga^5^

#### ^1^Faculty of Medicine and Health Sciences (MH faculty), Norwegian University of Science and Technology (NTNU), Trondheim, Norway; ^2^Department of Clinical and Molecular Medicine (IKOM), Faculty of Medicine and Health Sciences (MH faculty), Norwegian University of Science and Technology (NTNU), Trondheim, Norway; ^3^Department of Obstetrics and Gynecology, St. Olav's Hospital, Trondheim, Norway; ^4^Department of Clinical Services, St. Olav's Hospital, Trondheim, Norway; ^5^Department of Public Health and Nursing (ISM), Faculty of Medicine and Health Sciences (MH faculty), Norwegian University of Science and Technology (NTNU), Trondheim, Norway

**Introduction/Purpose:** Hyperactive pelvic floor has a complex etiology and symptom presentation. The Dutch self‐report questionnaire Amsterdam Hyperactive Pelvic Floor Scale (AHPFS) for women has not been tested in Norway. We wanted to use this to explore the symptom severity of hyperactive pelvic floor among different samples of women.

**Methods:** This is a descriptive cross‐sectional study with questionnaire data from patients referred to the gynecological outpatient clinic or to a pelvic floor physiotherapist at St. Olav's Hospital, members of Vulvodynia/Endometriosis Patient Associations and healthy volunteers. The participants were invited to fill out web‐based questionnaires addressing symptoms related to hyperactive pelvic floor, including the AHPFS, and sociodemographic data.

**Results:** A total of 397 women participated, reporting a mean score on the AHPFS of 12.4 (SD 3.9, range 6–30). Members of the patient associations scored highest (mean 15.5, SD 3.8), followed by patients from St. Olav's Hospital (mean 12.7, SD 4.0) and then healthy volunteers (mean 10.7, SD 2.8). We found greater symptom burden in patients referred to St. Olav's Hospital and members of the patient associations, and better mental health and sexual functioning in the healthy volunteers. The AHPFS scores were higher among unemployed women, women with chronic pelvic pain and vulvodynia, and women who had experienced sexual assault.

**Conclusions:** We found that hyperactive pelvic floor is prevalent in gynecological patients and in women with vulvodynia/endometrioses, but also among healthy controls. Knowledge about symptom severity and associated risk factors of hyperactive pelvic floor in women can be useful to better identify affected women.

## Impact of symptoms of anxiety and depression on the use of unplanned telephone contacts and visits with the healthcare after benign hysterectomy

### **Gulnara Kassymova** ^1^, Gunilla Sydsjö^1^, Lena Nilsson^2^, Ninnie Borendal Wodlin^1^, Preben Kjölhede^1^

#### ^1^Department of Obstetrics and Gynecology in Linköping, and Department of Biomedical and Clinical Sciences, Linköping University, Linköping, Sweden; ^2^Department of Anesthesiology and Intensive Care in Linköping, and Department of Biomedical and Clinical Sciences, Linköping University, Linköping, Sweden

**Introduction/Purpose:** The aim of the study was to evaluate the association between symptoms of anxiety and depression, and the occurrence of unplanned telephone contacts (uTCs) and unplanned visits (uVs) within 6 weeks after discharge from benign hysterectomy.

**Methods:** A randomized single‐blinded controlled multicenter intervention study comparing four telephone follow‐up strategies was conducted in five hospitals in southeast Sweden between 2011 and 2017. In all, 487 women, aged 28–59 years, scheduled for abdominal or vaginal hysterectomy for benign conditions were enrolled. Unplanned telephone contacts (uTCs) and unplanned visits (uVs) within 6 weeks after discharge with a healthcare provider were prospectively registered. Symptoms of anxiety and depression were assessed preoperatively by means of the Hospital Anxiety and Depression Scale (HADS‐A for anxiety; HADS‐D for depression).

**Results:** In summary, 224 (46.0%) women had uTCs and 203 (41.7%) had uVs. The multivariate analyses revealed that HADS‐A and HADS‐D, respectively, were significant independent risk factors for uTCs, but not for uVs. A higher score of HADS‐A and HADS‐D was associated with a higher occurrence of uTCs. Neither intervention nor age, BMI, mode of hysterectomy or duration of hospital stay influenced the occurrence of uTCs or uVs, whereas the occurrence of postoperative complications was a strong independent risk factor for uTCs and uVs.

**Conclusions:** Symptoms of anxiety and depression preoperatively and postoperative complications increase the burden on the patient and healthcare after benign hysterectomy. Efforts should be made to alleviate anxiety and depression in the perioperative course and reduce the emergence of surgical complications.

## Incomplete transverse vaginal septum presenting as recurrent vulvovaginitis – a rare presentation

### **Susan Diane Akinyi Adongo** ^1^, Abdul Ghani Nur Azurah^2^, Noorkardiffa Syawallina Omar^3^, Jerilee Mariam Khong Azhary^4^, Sweet Yi Esther Loh^2^, Ani Amelia Zainuddin^2^

#### ^1^Department of Obstetrics and Gynecology, Kenyatta National Hospital, Kenyatta, Kenya; ^2^Faculty of Medicine, Universiti Kebangsaan, Bangi, Malaysia; ^3^Department of Obstetrics and Gynecology, Universiti Teknologi MARA, Shah Alam, Malaysia; ^4^Department of Obstetrics and Gynecology, Universiti Malaya, Kuala Lumpur, Malaysia

**Introduction/Purpose:** Transverse vaginal septum is a rare abnormality of the female genital tract caused by a defect in the fusion of the urogenital sinus and the mullerian structures. It can either be complete or incomplete. We present a case to showcase its relevance as a differential diagnosis of recurrent vulvovaginitis.

**Methods:** A 29‐year‐old female presented with persistent, yellowish, foul‐smelling vaginal discharge and vulval itching for 5 years worsening after her menses or sexual intercourse. There was associated superficial dyspareunia, difficulty in full vaginal penetration but no abdominal pain. Abdominal exam was normal. Vaginal exam revealed a short vaginal length of 4 cm. The cervix could not be felt or seen on speculum exam. Pelvic ultrasound revealed minimal hematocolpos. Surgical excision of the septum was done. Copious amounts of pus mixed with old blood was drained and the wound edges sutured circumferentially. The patient is doing well 3 months after surgery.

**Results:** Complete transverse vaginal septum is commonly diagnosed in early adolescence with symptoms of primary amenorrhea, low abdominal pain, hematocolpos and dyspareunia. For incomplete septa, the presentation is more variable and may include secondary amenorrhea and recurrent vaginal infections. Vaginal septum in a patient with recurrent vaginitis is unlikely, especially in the context of relatively normal menses. Accumulation of menstrual debris above the septum and consequent infection may explain this. The diagnostic criteria for vaginal septa were utilized.

**Conclusions:** A diagnosis of incomplete transverse vaginal septum should be considered as a differential diagnosis in the management of patients with long‐standing recurrent vulvovaginitis unresponsive to treatment.

## The attitudes and practice patterns of obstetrician‐gynecologists towards sexual problems

### **Anna Aromaa** ^1,2,3^, Päivi Polo‐Kantola^2^ ^,3^, Sanna‐Mari Manninen^2,4^, Jarna Grönlund^5^, Markus Riskumäki^6^, Tero Vahlberg^6^, Katja Kero^2,3^

#### ^1^Department of Obstetrics and Gynecology, Satasairaala Central Hospital, Satakunta Hospital District, Pori, Finland; ^2^Department of Obstetrics and Gynecology, University of Turku, Turku, Finland; ^3^Department of Obstetrics and Gynecology, Turku University Hospital, Turku, Finland; ^4^Department of Health Promotion, Metropolia University of Applied Sciences, Helsinki, Finland; ^5^Occupational Healthcare Center, Mehiläinen, Raisio, Finland; ^6^Department of Biostatistics, University of Turku, Turku, Finland

**Introduction/Purpose:** Although female sexual problems are common, their assessment during obstetrician‐gynecologist (OB/GYN) appointments is not routine. We evaluated attitudes and practice patterns regarding sexual problems in the clinical work of Finnish OB/GYNs.

**Methods:** A web‐based questionnaire was sent through the register of The Finnish Society of Obstetrics and Gynecology. Background information included gender, age, education, occupational status, number of patients treated/day and number of patients dealt with sexual health issues/day.

The three fields included: (1) attitudes regarding sexual problems, (2) practice patterns regarding sexual history taking and (3) practice patterns regarding sexual problems.

**Results:** The survey was completed by 328 respondents and 299 questionnaires were eligible for the analysis (specialists 83%, *n* = 249, residents 17%, *n* = 50).

Most of the OB/GYNs (95%, *n* = 285/299) considered treating sexual problems as an important part of the healthcare but half (52%, *n* = 155/299) defined diagnosing female sexual problems to be difficult. Compared with the male OB/GYNs (29%, *n* = 6/21), the female OB/GYNs (54%, *n* = 149/278) were more likely to report the difficulty.

Less than half (45%, *n* = 136) reported asking often about sexual problems when taking general anamnesis. As a method to conduct sexual history, the majority (86%, *n* = 258/299) used open conversation. One‐third (34%, *n* = 102/299) reported distinct instructions in their organization relating to referring patients to continued care.

**Conclusions:** Even though almost all OB/GYNs reported treatment of sexual problems as an important healthcare issue, less than half of them asked about sexual problems. Diagnosing female sexual problems was considered difficult, and practice patterns regarding sexual problems seemed to be still unorganized.

## The risk of cervical stenosis after conization in postmenopausal women

### **Eva Hauge**, Pinar Bor

#### Department of Gynecology and Obstetrics, Randers Regional Hospital, Randers, Denmark

**Introduction/Purpose:** Since the HPV test was introduced in the Danish screening program in 2012, significantly more postmenopausal women are referred for colposcopy, and thus more diagnostic cone biopsies are performed due to inadequate colposcopy. Conization can cause cervical stenosis, which can lead to suboptimal follow‐up of cervical dysplasia and delayed diagnosis of endometrial pathology. Previous studies have found that the risk of stenosis is low in premenopausal women. In contrast, there is scant knowledge about the risk of stenosis after conization in postmenopausal women. Thus, the aim of this study was to investigate the risk of developing stenosis after conization in postmenopausal women.

**Methods:** Retrospective study. Women ≥45 years who underwent conization at the Randers Regional Hospital in 2012–2019 were included. Exclusion criteria; women in premenopause, who had previously conization, missed follow‐up or had cervical cancer. The frequency of cervical stenosis was assessed based on medical records (stenosis/agglutination) and treatment (dilation/re‐conization).

**Results:** In all, 567 conizations in women ≥45 years were performed during the study period; 300 women met the inclusion criteria and 79 women (26.3%) developed stenosis after conization. Age >60 years at the time of conization increased the risk of stenosis (relative risk [RR] 1.29, 95% confidence interval [CI] 1.04–1.57). In addition, persistent HPV was a risk factor for the development of stenosis (RR 1.51, 95% CI 1.02–2.22).

**Conclusions:** Postmenopausal women are at high risk of developing cervical stenosis after conization. The risk of stenosis should be included in the guidance of women in relation to diagnostic conization or continued conservative follow‐up, in cases where adequate colposcopy with biopsy is not possible.

## Vaginal CO_2_ laser therapy for genitourinary syndrome in breast cancer survivors – a randomized blinded controlled trial

### **Sine Jacobsen** ^1^, Marianne Glavind‐Kristensen^2^ , Anders Bonde Jensen^3,4^, Axel Forman^2^, Pinar Bor^1,4^

#### ^1^Department of Obstetrics and Gynecology, Randers Regional Hospital, Randers, Denmark; ^2^Department of Obstetrics and Gynecology, Aarhus University Hospital, Aarhus, Denmark; ^3^Department of Oncology, Aarhus University Hospital, Aarhus, Denmark; ^4^Department of Clinical Medicine, Aarhus University, Aarhus, Denmark

**Introduction/Purpose:** Between 50% and 75% of breast cancer survivors (BCS) experience one or more symptoms as part of the genitourinary syndrome of menopause (GSM), which results in impairment of their quality of life. Vaginal CO_2_ laser induces a wound‐healing cascade with formation of elastin‐collagen fibers resulting in tissue remodeling in the vaginal wall. Furthermore, laser therapy may improve proliferation of vaginal epithelial cells and glycogen production, which is essential to the growth of vaginal lactobacilli.

**Methods:** The main study is a single‐center, participant‐blinded, randomized study with 12 months’ follow‐up to compare the efficacy of vaginal laser therapy vs placebo laser in BSC on endocrine therapy. This study will be performed at the Department of Obstetrics and Gynecology, Randers Regional Hospital from January 2023 to June 2025.

1. Study one is a dose–response study with 30 participants exploring the ideal number of laser treatments needed to achieve an effect on GSM symptoms in BCS.
2. Study two is a randomized, blinded placebo‐controlled study with 60 participants comparing vaginal laser therapy with placebo laser therapy.
3. Study three is a 1‐year follow‐up of second study.

**Results:** Primary outcome was the 10‐cm visual analog scale (VAS) for vaginal dryness.

Secondary outcomes were VAS for vaginal pain, itching, soreness and the following validated questionnaires: FSFI, UDI‐6, ICIQ‐FLUTSsex, SCS‐W and VHI. Changes in vaginal histology and changes in vaginal and urine microbiome were other outcomes.

Patient inclusion started January 2023.

**Conclusions:** The short‐ and long‐term effect of vaginal laser therapy in breast cancer survivors with GSM symptoms will be investigated for the first time in Denmark.

## Women's health physiotherapists’ clinical practice and endometriosis patients’ views of general exercise – a mixed‐methods study

### **Merete Kolberg Tennfjord** ^1,2^, Kari Bø^1,3^, Marie Ellström Engh^1,4^, Rakel Gabrielsen^1^

#### ^1^Akershus University Hospital, Department of Obstetrics and Gynecology, Lørenskog, Norway; ^2^Kristiania University College, Department of Health and Training, Oslo, Norway; ^3^Norwegian School of Sport Sciences, Department of Sports Medicine, Oslo, Norway; ^4^University of Oslo, Faculty division Akershus University Hospital, Lørenskog, Norway

**Introduction/Purpose:** General exercise could be beneficial for women with endometriosis‐associated symptoms. However, there is limited knowledge of patients’ acceptance and tolerance of such treatment and how physiotherapists implement exercises for this patient group in clinical practice. The purpose of this mixed‐method study was to investigate endometriosis patients’ views of exercise as treatment and whether women's health physiotherapists are implementing general exercise in treating the condition.

**Methods:** A qualitative study among endometriosis patients participating in a randomized controlled trial (RCT) and an questionnaire‐based survey of women's health physiotherapists working in the Nordic countries. The RCT randomizes to a 1‐day course with education of exercise as pain management or the course plus weekly supervised moderate to high intensity general exercise over 4 months. A convenience sample of 19/82 study participants (mean age 31.4 years) was asked if participation in the study changed their view of exercise as part of the treatment for endometriosis. Thematic analysis was used. The questionnaire‐based survey covered questions of physiotherapy practice of general exercise for this patient group.

**Results:** A majority of participants were motivated to use exercise as part of their treatment in the future. The participants performing the exercises found them acceptable and tolerable. In all, 108 physiotherapists responded to the survey; 68% recommend individual general exercise as part of the treatment but only 15.7% offer supervised exercises.

**Conclusions:** After study participation, endometriosis patients were positive towards exercise as treatment, and supervised moderate to high‐intensity general exercise was accepted and tolerable. However, supervised exercise is not frequently offered by women's health physiotherapists.

## The influence of an extended postnatal program on contraceptive use during the first year postpartum – a randomized controlled trial

### **Kajsa Sandberg Kedfors** ^1^, Helena Hognert^1^, Ian Milsom^1^, Mattias Molin^2^, Ingela Lindh^1^

#### ^1^Department of Obstetrics and Gynecology, Sahlgrenska Academy at Gothenburg University, Gothenburg, Sweden; ^2^Statistiska Konsultgruppen Sweden AB, Gothenburg, Sweden

**Introduction/Purpose:** Low use of contraception postpartum is associated with an increased risk for an unplanned pregnancy. The objectives of this randomized controlled trial (RCT) were to investigate whether earlier and repeated postpartum care visits (PPCVs) would increase early initiation of contraception and contraceptive use at 1‐year postpartum.

**Methods:** Women in the 37th week of pregnancy were randomized to two PPCVs (the intervention group, 3 and 7 weeks) or to standard care (1 visit at 7 weeks). The participants completed questionnaires about contraception at each visit and at the 1‐year follow‐up.

**Results:** 958 women completed the study (intervention group, *n* = 447; standard group, *n* = 511). Contraceptive use at 7 weeks postpartum was higher in the intervention group (55.5%) compared with the standard group (48.5%), difference 6.9 (95% confidence interval [CI] 0.4–13.5) (*P =* 0.037). The use of long‐acting reversible contraceptives was more common in the intervention group (56.9%) compared with the standard group (48.0%), difference 8.9 (95% CI −0.3 to 18.0) (*P =* 0.059) and condom use was higher in the standard group (19.0%) than in the intervention group (12.1%), difference 6.9 (95% CI 0.1–13.6) (*P =* 0.047). Contraceptive use 1 year postpartum was higher in the intervention group (55.7%) than in the standard group (47.2%), difference 8.6 (95% CI 1.2–15.9) (*P =* 0.023).

**Conclusions:** These results indicate the importance of earlier and additional PPCVs to increase early start‐up of contraception and to obtain a higher frequency of contraceptive use at 1‐year postpartum to reduce unplanned pregnancies close to delivery.

## Use of different kinds of combined oral contraceptive pills in polycystic ovary syndrome: a systematic review and meta‐analysis

### **Maria Forslund** ^1,2^, Johanna Melin^2,3^, Simon Alesi^2^, Terhi Piltonen^4^, Daniela Romualdi^5^, Chau Thien Tay^2^, Selma Witchel^6^, Alexia Pena^7^, Aya Mousa^2^, Helena Teede^2,8^

#### ^1^Department of Obstetrics and Gynecology, Institute of Clinical Sciences, Sahlgrenska Academy, University of Gothenburg, Gothenburg, Sweden; ^2^Monash Centre for Health Research & Implementation, School of Public Health and Preventive Medicine, Monash University, Melbourne, Australia; ^3^Department of Obstetrics and Gynecology, University of Helsinki, Helsinki University Hospital, Helsinki, Finland; ^4^Department of Obstetrics and Gynecology, Clinical Research Unit, Medical Research Center, Oulu University Hospital, University of Oulu, Oulu, Finland; ^5^Department of Obstetrics and Gynecology, Fondazione Policlinico Universitario Agostino Gemelli IRCCS, Rome, Italy; ^6^Division of Pediatric Endocrinology, Department of UPMC Children's Hospital of Pittsburgh, University of Pittsburgh, Pittsburgh, PA, USA; ^7^Discipline of Pediatrics, The University of Adelaide and Robinson Research Institute, Adelaide, Australia; ^8^Endocrine and Diabetes Units, Monash Health, Melbourne, Australia

**Introduction/Purpose:** Combined oral contraceptive pills (COCPs) are one of the most commonly used medical treatments in women with polycystic ovary syndrome (PCOS) but it is unclear what the best formulation is. The objective was to compare effectiveness between different COCP preparations as part of the update of the International Evidence‐Based Guidelines on Assessment and Management of PCOS.

**Methods:** A systematic review and meta‐analysis was performed, aligning the PRISMA guidelines. Studies including women with PCOS, comparing two different COCP treatments in a randomized controlled trial were included. The random‐effects model was used in the meta‐analyses.

**Results:** Overall, 19 RCTs were identified. Fourth generation COCP may be more beneficial compared with third generation agents on biochemical hyperandrogenism, but evidence was largely of very low certainty. Ethinyl estradiol (EE)/cyproterone acetate (CPA) may be better than conventional COCPs in reducing hirsutism as well as biochemical hyperandrogenism (free androgen index, total testosterone), with low certainty of evidence. However, this may be associated with an increased metabolic risk (higher cholesterol and LDL) with very low certainty of evidence. No conclusion could be made regarding second vs later generations of COCP, or regarding EE doses, due to very low certainty of evidence. No evidence regarding natural estrogens in COCP was identified.

**Conclusions:** On current evidence, EE/CPA may be better than conventional COCPs in reducing clinical as well as biochemical hyperandrogenism but it is not recommended at present as a first line treatment in PCOS due to a higher VTE risk in the general population.

## Effect of metformin in the management of polycystic ovary syndrome: a systematic review and meta‐analysis of randomized controlled trials

### **Johanna Melin** ^1,2^, Maria Forslund^1,3^, Simon Alesi^1^, Terhi Piltonen^4^, Daniela Romualdi^5^, Poli Mara Spritzer^6^, Chau Thien Tay^1^, Alexia Pena^7^, Selma Feldman Witchel^8^, Aya Mousa^1^, Helena Teede^1,9^

#### ^1^Monash Centre for Health Research and Implementation, School of Public Health and Preventive Medicine, Monash University, Melbourne, Australia; ^2^Department of Obstetrics and Gynecology, University of Helsinki, Helsinki University Hospital, Helsinki, Finland; ^3^Department of Obstetrics and Gynecology, Institute of Clinical Sciences, Sahlgrenska Academy, University of Gothenburg, Sweden; ^4^Department of Obstetrics and Gynecology, Research Unit of Clinical Medicine and Medical Research Center, Oulu University Hospital, University of Oulu, Oulu, Finland; ^5^Department of Woman and Child Health and Public Health, Fondazione Policlinico Universitario “Agostino Gemelli” IRCCS, Rome, Italy; ^6^Gynecological Endocrinology Unit, Division of Endocrinology, Hospital de Clínicas de Porto Alegre, Universidade Federal do Rio Grande do Sul, Porto Alegre, Brazil; ^7^ Discipline of Paediatrics, The University of Adelaide and Robinson Research Institute, Adelaide, Australia; ^8^Division of Pediatric Endocrinology, Department of Pediatrics, UPMC Children's Hospital of Pittsburgh, Pittsburgh, PA, USA; ^9^Endocrine and Diabetes Units, Monash Health, Melbourne, Australia

**Introduction/Purpose:** PCOS is characterized by insulin resistance and hyperandrogenism, exacerbated by excess bodyweight, and carries an increased risk for cardiometabolic disorders. Metformin is known to improve insulin sensitivity and have a positive effect on weight in women with PCOS. However, key knowledge gaps remain, regarding efficacy of metformin for specific health outcomes metformin in PCOS. The aim of this study was to evaluate the effectiveness of metformin, alone or in combination with lifestyle interventions, in the management of PCOS.

**Methods:** This systematic review with meta‐analyses was conducted according to PRISMA guidelines and included randomized controlled trials (RCTs) conducted on humans and published in English through July 2022. Screening, risk of bias assessments and quality appraisal were performed in duplicate.

**Results:** We included 32 RCTs, comprising 864 women with PCOS, using a metformin dose of 850–2000 mg. There was moderate certainty of evidence that metformin was superior to placebo in lowering body mass index (BMI) (MD −0.53, 95% confidence interval [CI] −0.95 to −0.12 kg/m^2^), homeostatic model assessment for insulin resistance (HOMA‐IR) (MD −0.50, 95% CI −0.91 to −0.09) and fasting glucose (MD −0.13, 95% CI −0.19 to −0.07 mmol/L). For BMI, fasting glucose and lipids, the effect of metformin was more pronounced in the subgroup with BMI >25 kg/m^2^.

**Conclusions:** These findings inform the forthcoming 2023 International Evidence‐based PCOS guidelines recommending that metformin should be considered as an adjunct to active lifestyle intervention in all adult women with PCOS, especially for those who have a BMI >25 kg/m^2^, for improving weight, insulin resistance and lipid profile.

## Climacteric status at the age of 46: association with perceived work ability and a 2‐year follow‐up on disability days and unemployment days

### **Tiia Saarinen** ^1,2,3^, Susanna Savukoski^1,2,3^, Paula Pesonen^4^, Eeva Vaaramo^4^, Leena Ala‐Mursula^5^ , Maarit Niinimäki^1,2,3^

#### ^1^Department of Obstetrics and Gynecology, Oulu University Hospital, Wellbeing Services County of North Ostrobothnia Oulu; ^2^Research Unit of Clinical Medicine, University of Oulu, Oulu, Finland; ^3^Medical Research Center, University of Oulu, Oulu University Hospital, Wellbeing Services County of North Ostrobothnia, Oulu, Finland; ^4^Infrastructure for Population Studies, Faculty of Medicine, University of Oulu, Oulu, Finland; ^5^Center for Life Course Health Research, Faculty of Medicine, University of Oulu, Oulu, Finland

**Introduction/Purpose:** Menopausal transition can have negative impact on women's work ability as a result of bothersome symptoms and increased morbidity. Mean age at menopause is 51 years. We investigated how preceding early‐onset climacteric transition affects women's perceived work ability at the age of 46 and consequent registered disability and unemployment.

**Methods:** The study population consisted of 2661 women from the prospective Northern Finland Birth Cohort 1966 who participated a follow‐up study at the age of 46 years. These women were divided into two groups by their climacteric status, based on their menstrual history and follicle‐stimulating hormone value: climacteric and preclimacteric women. The participants’ work ability was investigated using the Work Ability Score (0–7 = poor vs 8–10 = good). Their consequent disability and unemployment days during the following 2 years after the study visit were collected from national registers. The association between climacteric status and poor work ability was assessed with binary logistic regression. Disability and unemployment days were analyzed with Poisson regression.

**Results:** Climacteric women were more often smokers and had a lower level of education. More advanced climacteric status at the age of 46 was associated with poor work ability (odds ratio [OR] 1.43, 95% confidence interval [CI] 1.07–1.89) and higher risk for disability and unemployment days in the 2‐year follow‐up incidence rate ratio (IRR): respectively 1.11 (95% CI 1.09–1.13) and 1.13 (95% CI 1.11–1.14) when adjusted with smoking and education.

**Conclusions:** Climacteric status at the age of 46 is associated with 1.4‐fold risk of poor perceived work ability and 11%–13% higher risk for disability and unemployment, corresponding to over 1 month of lost workdays in 2 years.

## Risk of preterm birth after active surveillance for cervical intraepithelial neoplasia grade 2

### **Kathrine Dyhr Lycke** ^1,2,3^, Johnny Kahlert^4^, Dina Overgaard Eriksen^1,2,3^, Camilla Omann^1,3^, Lars Henning Pedersen^5,6^, Iben Sundtoft^1^, Lone Kjeld Petersen^7,8^, Anne Hammer^1,2,3^

#### ^1^Department of Obstestrics and Gynecology, Gødstrup Hospital, Herning, Norway; ^2^ NIDO | Center for Research and Education, Gødstrup Hospital, Lørenskog, Denmark; ^3^Department of Clinical Medicine, Aarhus University, Aarhus, Denmark; ^4^Department of Clinical Epidemiology, Aarhus University Hospital, Aarhus, Denmark; ^5^Department of Obstetrics and Gynecology, Aarhus University Hospital, Aarhus, Denmark; ^6^Department of Biomedicine, Aarhus University, Aarhus, Denmark; ^7^Department of Obstetrics and Gynecology, Odense University Hospital, Odense, Denmark; ^8^Department of Clinical Research, University of Southern Denmark, Odense, Denmark

**Introduction/Purpose:** To reduce the risk of preterm birth associated with loop electrosurgical excision procedure (LEEP), many countries have introduced active surveillance of cervical intraepithelial neoplasia grade 2 (CIN2). However, cervical dysplasia may also be associated with preterm birth. Here, we aimed to investigate the risk of preterm birth in women undergoing active surveillance for CIN2.

**Methods:** We conducted a Danish register‐based cohort study on women with CIN2 and a subsequent singleton delivery from 1998 through 2018. Women were categorized into active surveillance (cervical biopsy and/or cytology) or LEEP based on their first cervical sample after CIN2. We assessed the risk of preterm (<37+0 weeks) and moderate preterm (<34+0 weeks) birth and calculated relative risks (RR) using modified Poission regression.

**Results:** We included 10 924 women with CIN2 and subsequent delivery. In total, 910 births (8.3%) were preterm (<37+0 weeks) and 234 (2.1%) were moderate preterm (<34+0 weeks). The risk of preterm (<37+0 weeks) and moderate preterm (<34+0 weeks) birth was comparable between active surveillance and immediate LEEP (relative risk [RR] 0.97, 95% confidence interval (CI) 0.86–1.10, and RR 0.89, 95% CI 0.71–1.12, respectively). However, for women undergoing LEEP during or after active surveillance, the risk of preterm birth (<37+0 weeks) was higher than for women treated with immediate LEEP (RR 1.29, 95% CI 1.10–1.52). Similar results were observed when stratifying by parity, age and cytology at CIN2 diagnosis. Adjusted estimates will be presented at the conference.

**Conclusions:** The risk of preterm birth was comparable between women undergoing active surveillance or immediate LEEP of CIN2, but the risk was higher for women treated with LEEP after initial active surveillance.

## Regression and progression rates of untreated cervical intraepithelial neoplasia grade 2

### **Kathrine Dyhr Lycke** ^1,2,3^, Johnny Kahlert^4^, Rikke Kamp Damgaard^1,2,3^, Dina Overgaard Eriksen^1,2,3^, Mary Holten Bennetsen^5^, Lone Kjeld Petersen^6,7^, Patti Gravitt^8^, Anne Hammer^1,2,3^

#### ^1^Department of Obstetrics and Gynecology, Gødstrup Hospital, Herning, Denmark; ^2^ NIDO | Center for Research and Education, Gødstrup Hospital, Herning, Denmark; ^3^Department of Clinical Medicine, Aarhus University, Aarhus, Denmark; ^4^Department of Clinical Epidemiology, Aarhus University Hospital, Aarhus, Denmark; ^5^Department of Pathology, Randers Regional Hospital, Randers, Denmark; ^6^Department of Obstetrics and Gynecology, Odense University Hospital, Odense, Denmark; ^7^Department of Clinical Research, University of Southern Denmark, Odense, Denmark; ^8^Center for Global Health, National Cancer Institute, Rockville, MD, USA

**Introduction/Purpose:** Studies have demonstrated spontaneous regression rates of 50%–60% for cervical intraepithelial neoplasia grade 2 (CIN2). The high regression rates have resulted in implementation of active surveillance in many countries; however, most studies included less than 100 women. Here, we aimed to estimate regression and progression of untreated CIN2 in a larger setting using the Danish healthcare registers.

**Methods:** We conducted a nationwide register‐based cohort study on women with incident CIN2 from 1998 to 2020 who underwent active surveillance. We excluded women aged 40+ years and women with prior CIN2+ or LEEP. Using the cumulative incidence function, we estimated regression (histologic ≤CIN1) and progression (histologic CIN3+) rates at 6, 12, 18 and 24 months after CIN2 diagnosis. We used modified Poisson regression to estimate the relative risk (RR) of progression within 24 months stratified by index cytology and age.

**Results:** We included 11 056 women with CIN2. Most were 23–29 years (61%) and nearly half had high‐grade index cytology (46%). The regression rate after 24 months was 62.9% (95% confidence interval [CI] 61.9–63.8) and the progression rate was 33.3% (95% CI 32.4–34.2). Women with high‐grade index cytology had higher risk of CIN3+ compared with women with normal index cytology (adjusted relative risk [RR] 1.58, 95% CI 1.43–1.76). Of note, we found no difference in risk of progression between women aged 30–40 and 23–29 (adjusted RR 0.98, 95% CI 0.88–1.10).

**Conclusions:** Our findings support active surveillance for CIN2, particularly in women with ≤ low‐grade index cytology. However, studies are needed to explore the potential long‐term consequences of active surveillance for CIN2.

## The educational game SonoQz improves diagnostic accuracy in ultrasound assessment of ovarian tumors

### **Erica Smedberg** ^1,2^, Måns Åkerlund^3^, Mikael Andersson Franko^1^, Elisabeth Epstein^1,2^

#### ^1^Department of Clinical Science and Education, Södersjukhuset, Karolinska Institutet, Stockholm, Sweden; ^2^Department of Gynecology and Obstetrics, Södersjukhuset, Stockholm, Sweden; ^3^Harvard University, Cambridge, MA, USA

**Introduction/Purpose:** SonoQz was developed as an educational game for medical doctors to practice assessment of ovarian tumors. The aim of the study was to determine whether SonoQz improves diagnostic accuracy in ultrasound assessment of ovarian tumors.

**Methods:** The game comprises ultrasound images of 324 ovarian tumors. Training in the SonoQz app, assessing at least 200 cases, was preceded by a pre‐training test, and followed by a post‐training test. In the training phase, each case was followed by correct answer and a comment about the specific case. Two equal tests (A and B), each consisting of 20 cases, were used as pre‐ and post‐training tests. Users were asked to classify each tumor (1) according to IOTA Simple Rules and (2) as benign or malignant, and (3) to suggest a specific histological diagnosis. Logistic mixed models were used to determine any improvement in test scores, sensitivity, and specificity.

**Results:** Fifty‐eight doctors from 19 medical centers participated. Comparing the pre‐ and post‐training test, median scores in Simple Rules assessment increased from 73% to 83%, differentiation between benign and malignant tumors from 86% to 95%, and making a specific diagnosis from 43% to 63% (*P* < 0.001). When classifying tumors as benign or malignant, specificity increased from 70% to 89% (*P* < 0.001); sensitivity was not changed (98% vs 97%, *P* = 0.157).

**Conclusions:** SonoQz is an effective educational game that significantly improves accuracy in assessing ovarian tumors. Additionally, it increases the chance of correctly detecting ovarian cancer by reducing the number of false positives while maintaining high sensitivity.

## Intraoperative cyst rupture in the surgical treatment of ovarian cysts – a national prospective cohort study

### **Maliha Chaudhry** ^1^, Pernilla Dahm Kähler^1^, Maria Forslund^1^, Karin Sundfeldt^1^, Benjamin Ulfenborg^2^

#### ^1^Department of Obstetrics and Gynecology, Institute of Clinical Sciences, Sahlgrenska Academy, University of Gothenburg, Gothenburg, Sweden; ^2^School of Bioscience, Department of Biology and Bioinformatics, University of Skövde, Skövde, Sweden

**Introduction/Purpose:** The gold standard treatment for assumed benign ovarian cysts is laparoscopy. Although this method has many benefits, it has been proposed that laparoscopy increases the risk of intraoperative cyst rupture. This study examined the impact of laparoscopy as a treatment for ovarian cysts, as well as the impact of cystic enucleation and baseline characteristics, on the risk of intraoperative cyst rupture.

**Methods:** A total of 46 952 patients underwent surgery for ovarian cysts during the study period from 2008 to 2020. Of these, data on cyst rupture were available in 34 481 patients: namely, preoperative cyst rupture (*n* = 3385), intraoperative cyst rupture (*n* = 10 028) or no rupture (*n* = 21 068). The cohort consisted of 31 096 patients including the groups with intraoperative cyst rupture and no rupture. Data were extracted from The Swedish National Quality Register for Gynecological Surgery.

**Results:** Of the 31 096 patients, a greater proportion of women with intraoperative cyst rupture had the cyst removed by laparoscopy (69.38% vs 48.46%) and a greater proportion of women with intraoperative cyst rupture had the cyst removed by cystic enucleation (5.03% vs 1.44%). The removal of ovarian cysts by laparoscopy was associated with an increased adjusted odd ratio [aOR] for cyst rupture (aOR 2.6). Cysts removed through cystic enucleation also had increased odds of cyst rupture (aOR 3.1). Other factors associated with increased odds of intraoperative cyst rupture were intraabdominal adhesions (aOR 2.1) and endometriosis (aOR 3.2).

**Conclusions:** Laparoscopy increases the risk of intraoperative cyst rupture, as does cystic enucleation, intraabdominal adhesions and endometriosis. It is recommended that these risk factors are considered at the preoperative assessment.

## 6. UROGYNECOLOGY

## Subjective and objective cure rates up to 20 years after tension‐free vaginal tape (TVT) surgery

### **Berit Rein Solhaug** ^1^, Ingrid Volloyhaug^1^, Rune Svenningsen^2^, Maria Oyasaether Nyhus^1^

#### NTNU/St. Olav's Hospital, Trondheim, Norway; ^2^Oslo Universitetssykehus/Ullevål, Oslo, Norway

**Introduction/Purpose:** Retropubic tension‐free vaginal tape (TVT) has been the preferred surgical treatment for stress urinary incontinence the last 20 years. The aim of this study was to evaluate subjective and objective cure rates up to 20 years after TVT surgery.

**Methods:** We merged results from two sub‐cohorts having undergone TVT surgery in 2001–2004 and 2011–2012 at three Norwegian Hospitals. They attended a physical follow‐up as part of a cross‐sectional study in 2022. Subjective cure was defined as stress index <3 using a validated questionnaire, and objective cure as <3 g on a cough–jump pad‐weighing test. Voiding difficulties were assessed by flowrate and residual volume. Mann–Whitney *U*‐test was used for comparative analyses.

**Results:** In all, 268 women were examined. Mean age was 62 (range 37–91) years, BMI 26.5 (SD 11.9) kg/m^2^ and time since surgery 16.5 years (SD 4.5). Sixty‐one percent (146/240) were subjectively cured compared with 95% (210/221) objectively cured on testing. No difference between subjectively cured and non‐cured was found for mean flow (8.7 vs 8.1 mL/s, *P* = 0.33), max. flow (18.6 vs 16.8 mL/s, *P* = 0.12) or residual volume (83.3 vs 87.8 mL, *P* = 0.12). However, subjectively cured had less leakage on objective testing than subjectively non‐cured (0.04 vs 4.6 g, *P* < 0.001).

**Conclusions:** Sixty‐one percent were subjectively cured and 95% objectively cured on testing up to 20 years after TVT. Subjectively cured had significantly less leakage on objective testing compared with subjectively non‐cured women. Flow and residual volume did not differ between subjectively cured and non‐cured.

## Laparoscopic sacral colpopexy vs vaginal sacrospinous colpopexy for treatment of post‐hysterectomy vaginal vault prolapse. A randomized trial

### **Møyfrid Kjøllesdal** ^1^, Marie Ellström Engh^2,3^, Mats Brännström^1^, Jan‐Henrik Stjerndahl^1^, Milada Cvancacova Småstuen^4^, Janusz Marcickiewicz^5^

#### ^1^Department of Obstetrics and Gynecology, Institute of Clinical Science, Sahlgrenska Academy, University of Gothenburg, Gothenburg, Sweden; ^2^Department of Obstetrics and Gynecology, Akershus University Hospital, Lørenskog, Norway; ^3^University of Oslo, Faculty Division, Akershus University Hospital, Lørenskog, Norway; ^4^University of Oslo, Oslo, Norway; ^5^Division of Obstetrics and Gynecology, Hallands County Hospital, Varberg, Sweden

**Introduction/Purpose:** There are no randomized controlled trials listed in the last Cochrane report comparing laparoscopic sacral colpopexy (LSC) with vaginal sacrospinous colpopexy (VSC) for the treatment of post‐hysterectomy vaginal vault prolapse. The purpose of this study was to compare laparoscopic sacral colpopexy to vaginal sacrospinous colpopexy in terms of objective and subjective outcomes with evaluations at 3, 12 and 24 months after surgery.

**Methods:** This randomized controlled trial was performed at Sahlgrenska University Hospital, Gothenburg, Sweden, during 2007–2013. Women with a previous hysterectomy were included when they had a symptomatic vaginal descent of >grade 2. Randomization was done using a 1:1 allocation ratio.

The primary outcome was the presence of anatomical failure defined clinically as having the vaginal vault, the most descending point of the anterior or posterior vaginal wall at the hymen or below, at 1 year postoperatively. The subjective outcome was assessed with the Pelvic Floor Distress Inventory symptom questionnaire.

**Results:** In all, 62 women in the VSC group and 57 women in the LSC group received allocated intervention. For vaginal vault there was a more pronounced descent in the VSC group at baseline. At 1 year there was no statistically significant difference in failure rate as defined above, between the laparoscopic (46.4%; 95% confidence interval [CI] 33.0–60.2) and vaginal (39.7%, 95% CI 27.6–to 52.8) groups. There was no significant difference between groups at any time point in the Pelvic Floor Distress Inventory symptom questionnaire.

**Conclusions:** Both laparoscopic and vaginal colpopexy seem to be effective and safe surgical procedures for the treatment of post‐hysterectomy vaginal vault prolapse.

## Does perineorrhaphy improve sexual health?

### **Malou Barbosa**, Lise Brogaard, Elin Sellén, Mette Meinert

#### Aarhus University Hospital, Aarhus, Denmark

**Introduction/Purpose:** Following vaginal delivery, an insufficiently repaired perineal tear may lead to an enlarged genital hiatus, a short perineum and consequently impaired sexual health. Perineorrhaphy is a surgical procedure where the perineal muscles are reconstructed to strengthen the perineal body, lengthen the perineum and augment the support of the pelvic organs. Although being commonly performed, little evidence exists regarding functional outcomes. It has been suggested that a perineorrhaphy can improve sexual function. The aim of this study was to prospectively assess female satisfaction and sexual distress following a perineorrhaphy.

**Methods:** The study included women undergoing a stand‐alone perineorrhaphy at Aarhus University Hospital during 2012–2019. All women answered a questionnaire, the Female Sexual Function Index (FSFI) or the Female Sexual Distress Scale (FSDS), pre‐operatively and 6 months post‐operatively.

**Results:** Forty‐eight women completed the follow‐up. The female sexual distress score was significantly reduced 6 months after the surgery. There was a tendency towards a higher sexual function; however, this was not statistically significant. Nonetheless, there was a reduction in women with sexual dysfunction.

**Conclusions:** A perineorrhaphy may have a positive impact on sexual health. It significantly decreases sexual distress and there seems to be a trend towards a higher sexual satisfaction following the procedure.

## Sexual function and pain 10–14 years after insertion of a mid‐urethral sling among women with stress urinary incontinence in Sweden

### **Anna Lundmark Drca** ^1^, Vasilis Alexandridis^2^, Marie Westergren Söderberg^1^, Maria Andrada^2^, Pia Teleman^2^, Marion Ek^1^

#### ^1^Department of Clinical Sciences and Education, Karolinska Institutet, Stockholm, Sweden; ^2^Department of Clinical Sciences, Lund University, Lund, Sweden

**Introduction/Purpose:** For more than 30 years the mid‐urethral sling (MUS) has been used to cure stress urinary incontinence. The objective of this study was to assess whether surgical technique affects the outcome after more than 10 years, regarding dyspareunia and pelvic pain.

**Methods:** In this longitudinal cohort study we used the Swedish National Quality Register of Gynecological Surgery to identify women who underwent MUS surgery in 2006–2010. Of 4348 eligible women, 2555 (59%) responded to the questionnaire sent out in 2020–2021. The two main surgical techniques, the retropubic and the obturatoric approach, were represented by 1562 and 859 women, respectively. The Urogenital Distress Inventory‐6 (UDI‐6) and the Pelvic Organ Prolapse/ Urinary Incontinence Sexual Questionnaire (PISQ‐12) as well as general questions concerning the MUS surgery were sent out to the study population. Dyspareunia and pelvic pain were defined as primary outcomes. Secondary outcomes included PISQ‐12, general satisfaction and self‐reported problems due to insertion of the sling.

**Results:** A total of 2421 women were included in the analysis; 71% reported on suffering from dyspareunia or not and 77% reported on whether suffering from pelvic pain or not. In a multivariate logistic regression analysis of the primary outcomes we found no difference between the retropubic technique and the obturatoric technique (15% vs 17%, odds ratio [OR] 1.1, 95% confidence interval [CI] 0.8–1.5). Equally important, the same applies for pelvic pain (17% vs 18%, OR 1.0, 95% CI 0.8–1.3).

**Conclusions:** Dyspareunia and pelvic pain 10–14 years after insertion of a MUS do not differ with respect to surgical technique.

## 7. GYNECOLOGICAL ONCOLOGY

## Treatment CIN: May women with a negative co‐test at first follow‐up visit return to 3‐year screening?

### **Finn Egil Skjeldestad** ^1^, Sveinung Wergeland Sørbye^2^

#### ^1^Institute of Community Medicine, UiT, The Arctic University of Norway, Tromsø, Norway; ^2^Department of Clinical Pathology, The University Hospital of North Norway, Tromsø, Norway

**Introduction/Purpose:** Some screening programs recommend that women treated for CIN only be returned to 3‐year screening after receiving two consecutive negative co‐tests. Here we evaluate adherence to these guidelines and assessed risk of residual CIN3+ disease.

**Methods:** This cross‐sectional study comprised 1397 women treated for CIN between 2014 and 2017, who had their cytology, HPV and histology samples analyzed at a single university department of pathology. Women who had the first and second follow‐up at 4–8 and 9–18 months, respectively, after treatment were considered adherent to guidelines. Follow‐up ended December 31, 2021. We used survival analysis to assess residual CIN3+ disease among women with one and two negative co‐tests, respectively.

**Results:** 71.8% (1003/1397) of women attended the first follow‐up 4–8 months after treatment, and 38.3% were considered adherent at second follow‐up. Nearly 30% of women had incomplete follow‐up at the study end. None of the 808 women who returned to 3‐year screening after two negative co‐tests were diagnosed with CIN3+, whereas two such cases were diagnosed among 887 women who had normal cytology/ASCUS/LSIL and a negative HPV test at first follow‐up (5‐year risk of CIN3+: 0.24, 95% confidence interval 0.00–0.57 per 100 woman‐years).

**Conclusions:** The adherence to follow‐up guidelines was low. The high proportion of women with incomplete follow‐up at study end requires action. The risk of CIN3+ among women with normal cytology/ASCUS/LSIL and a negative HPV test at first follow‐up supports a return to 3‐year screening.

## The risk of cervical cancer following active surveillance for cervical intraepithelial neoplasia grade 2

### Kathrine Dyhr Lycke^1,2,3^, Johnny Kahlert^4^, Lone Kjeld Petersen^5,6^, Rikke Kamp Damgaard^1,2,3^, Li C. Cheung^7^, Patti Gravitt^8^, **Anne Hammer** ^1,2,3^

#### ^1^Department of Obstetrics and Gynecology, Gødstrup Hospital, Herning, Denmark; ^2^ NIDO | Center for Research and Education, Gødstrup Hospital, Herning, Denmark; ^3^Department of Clinical Medicine, Aarhus University, Aarhus, Denmark; ^4^Department of Clinical Epidemiology, Aarhus University Hospital, Aarhus, Denmark; ^5^Department of Obstetrics and Gynecology, Odense University Hospital, Odense, Denmark; ^6^Department of Clinical Research, University of Southern Denmark, Odense, Denmark; ^7^Division of Cancer Epidemiology and Genetics, National Cancer Institute, Bethesda, MD, USA; ^8^Center for Global Health, National Cancer Institute, Rockville, MD, USA

**Introduction/Purpose:** Many countries are implementing active surveillance for cervical intraepithelial neoplasia grade 2 (CIN2) instead of immediate loop electrosurgical excision procedure (LEEP). However, the long‐term consequences of active surveillance have note been investigated. We aimed to investigate the risk of cervical cancer in women undergoing active surveillance for CIN2.

**Methods:** As active surveillance began in the 90's in Denmark, we used the Danish registries to conduct a nationwide cohort study on all women diagnosed with CIN2 from 1998 to 2020. Women were categorized into active surveillance (ie record of subsequent punch biopsy/cytology) or immediate LEEP. We used a Weibull survival model for interval‐censored time‐to‐event data to estimate the cumulative risk of cervical cancer in women undergoing active surveillance or immediate LEEP for CIN2. Estimates were adjusted for age, index cytology, calendar time and residing area.

**Results:** We included 27 524 women with CIN2; 12 483 (45%) underwent active surveillance and 15 041 (55%) were treated with immediate LEEP. During follow‐up, we identified 104 cases of cervical cancer: 56 (54%) in the active surveillance group and 48 (46%) in the LEEP group. The cumulative risk of cervical cancer was comparable across the two groups after the first 2 years of follow‐up. Hereafter, the risk increased in the active surveillance group, reaching 2.65% (95% confidence interval [CI] 2.07–3.23) after 20 years, while it remained stable in the LLETZ group (0.76%, 95% CI 0.58–0.95)).

**Conclusions:** Active surveillance for CIN2 is associated with increased risk of cervical cancer compared with immediate LEEP. These findings demonstrate the importance of increased surveillance of these women.

## Can pretreatment with vaginal estrogen improve the colposcopy performance in postmenopausal women? A randomized controlled trial

### **Vibe Munk Bertelsen** ^1^, Mette Tranberg^2^, Lone Kjeld Petersen^3^, Berit Bargum Booth^4^, Pinar Bor^5^

#### ^1^Department of Gynecology and Obstetrics, Randers Regional Hospital and Department of Clinical Medicine, Aarhus University, Aarhus, Denmark; ^2^University Research Clinic for Cancer Screening, Randers Regional Hospital, Randers, Denmark; ^3^Department of Gynecology and Obstetrics, Odense University Hospital and Department of Clinical Medicine, University of Southern Denmark, Odense, Denmark; ^4^Department of Gynecology and Obstetrics, Odense University Hospital and Randers Regional Hospital, Odense and Randers, Denmark; ^5^Department of Gynecology and Obstetrics, Randers Regional Hospital and Department of Clinical Medicine, Randers and Aarhus University, Aarhus, Denmark

**Introduction/Purpose:** Colposcopy is the most important diagnostic tool to detect cervical precancerous lesions and thereby prevention of cervical cancer. However, colposcopy is challenging in postmenopausal women, as the majority will have a nonvisible transformation zone, due to age‐dependent changes of the cervix. The risk is lengthy follow‐up with repeated colposcopies and increased risk of missing disease. This study will be among the first to investigate whether treatment with vaginal estrogen prior to colposcopy will improve the colposcopy performance in postmenopausal women.

**Methods:** A randomized controlled multicenter trial. Enrollment will be performed at gynecology departments in the Region of Southern Denmark and Central Denmark Region. A total of 150 postmenopausal women aged ≥50 years referred for colposcopy due to abnormal cervical screening results will be included. Eligible women will be randomized 1:1 to pretreatment with either vaginal application of Vagifem 30 μg or placebo once a day for 14 days prior to colposcopy.

**Results:** The trial has just started enrollment. The primary outcome will be to compare the two groups regarding the percentage of women with a visible transformation zone at colposcopy, and biopsies representative of the transformation zone.

**Conclusions:** The results are clinically relevant and may contribute with new evidence on how to improve diagnostic work‐up of postmenopausal women. This study has the potential to ensure accurate and timely diagnosis of precancerous cervical lesions among postmenopausal women, which is necessary to further reduce the number of women with late‐stage cervical cancers and poor prognosis.

## Human papilloma virus genotypes for risk assessment of cervical intraepithelial neoplasia grade 2 (CIN2)

### **Rikke Kamp Damgaard** ^1,2,3^, David Jenkins^4^, Mark H. Stoler^5^, Kathrine Dyhr Lycke^1,2,3^, Maurits de Koning^4^, Wim Quint^4,6^, Patti Gravitt^7^, Torben Steiniche^8^, Lone Kjeld Petersen^9,10^, Anne Hammer^1,2,3^

#### ^1^Department Gynecology & Obstetrics, Gødstrup Hospital, Herning, Denmark; ^2^Department Clinical Medicine, Aarhus University, Aarhus, Denmark; ^3^ NIDO I Center for Research and Education, Gødstrup Hospital, Herning, Denmark; ^4^ Viroclinics‐DDL, DDL Diagnostic Laboratory, Rijswick, The Netherlands; ^5^Department of Pathology, University of Virginia, Charlottesville, VA, USA; ^6^Methylomics, Rijswijk, The Netherlands; ^7^Center for Global Health, National Cancer Institute, Bethesda, MD, USA; ^8^Department of Pathology, Aarhus University Hospital, Aarhus, Denmark; ^9^Department of Gynecology & Obstetrics, University Hospital of Southern Denmark, Odense, Denmark; ^10^Department Clinical Research, University of Southern Denmark, Odense, Denmark

**Introduction/Purpose:** Recently, many countries have switched to active surveillance of women diagnosed with CIN2. As recurrent examinations may cause concerns about disease progression, it is essential to identify women at lowest likelihood of regression and need of immediate excisional treatment. Here, we aimed to explore the performance of human papilloma virus (HPV) genotypes in predicting regression of CIN2.

**Methods:** In this historical cohort, we included women aged 23–40 undergoing active surveillance for CIN2 in Denmark. Women were indentified through the Danish Pathology Data Bank (DPDB) at Aarhus University Hospital. HPV genotyping was performed on biopsies using the HPV SPF_10_‐ DEIA‐LiPA_25_ system. Regression was defined as having a subsequent record of CIN1 or less during follow‐up. We used a modified Poisson model to estimate likelihood of diagnostic regression in women positive for HPV16 and/or 18 vs other high‐risk HPV genotypes (hrHPV). Results were presented overall, and stratified by age and cytology.

**Results:** We identified 3623 women with a CIN2 diagnosis in DPDB, of whom 455 were included. Median age was 27 (interquartile range 24–30). Most women were positive for hrHPV (87%) and HPV16 was the most prevalent genotype (36%). During follow‐up, 58% of all women had diagnostic regression. In our main analysis we found that women positive for HPV16 or HPV16/18 were less likely to regress (0.86, 95% confidence interval [CI] 0.81–0.91, and 0.88, 95% CI (0.83–0.94), respectively, as compared with other hrHPV genotypes.

**Conclusions:** Our findings imply, that women positive for HPV16/18 are less likely to regress and may benefit from immediate excisional treatment at CIN2 diagnosis.

## Distribution of HPV, age, cytology and expert review in women undergoing active surveillance for cervical intraepithelial neoplasia grade 2 (CIN2)

### **Rikke Kamp Damgaard** ^1,2,3^, David Jenkins^4^, Mark H. Stoler^5^, Miekel M. van de Sandt^4^, Kathrine Dyhr Lycke^1,2,3^, Maurits de Koning^4^, Wim Quint^4,6^, Torben Steiniche^7^, Lone K. Petersen^8,9^, Anne Hammer^1,2,3^

#### ^1^Department Gynecology & Obstetrics, Gødstrup Hospital, Herning, Denmark; ^2^Department Clinical Medicine, Aarhus University, Denmark; ^3^ NIDO I Center for Research and Education, Gødstrup Hospital, Herning, Denmark; ^4^ Viroclinics‐DDL, DDL Diagnostic Laboratory, Rijswijk, The Netherlands; ^5^Department of Pathology, University of Virginia, Charlottesville, VA, USA; ^6^Methylomics, Rijswijk, The Netherlands; ^7^Department of Pathology, Aarhus University Hospital, Aarhus, Denmark; ^8^Department of Obstetrics and Gynecology, University of Southern Denmark, Odense, Denmark; ^9^Department of Clinical Research, University of Southern Denmark, Odense, Denmark

**Introduction/Purpose:** Many countries have switched to active surveillance of cervical intraepithelial neoplasia grade 2 (CIN2). However, little is known about the most favorable criteria for active surveillance. Here, we aimed to describe the distribution of HPV genotypes, cytology, age and expert review in women undergoing active surveillance for CIN2 to help clarify criteria for active surveillance.

**Methods:** In this cross‐sectional study, we included women aged 23–40 undergoing active surveillance for CIN2. Women were selected from the Danish Pathology Data Bank (DPDB), Aarhus University Hospital. Archived biopsies underwent HPV SPF_10_‐ DEIA‐LiPA_25_ analysis. We estimated the prevalence of high‐risk HPV (hrHPV), overall and stratified by age and index cytology. As an ancillary, we explored the distribution of hrHPV, cytology and age in expert confirmed CIN2 and nonconfirmed (>CIN2 and <CIN2) diagnosis.

**Results:** We identified 3623 women with a CIN2 diagnosis, of whom 453 were included. Most women were aged ≤30 (74%) and 52% had a high‐grade cytology. The prevalence of hrHPV genotypes was 87% and HPV16 was the most prevalent (35%). The hrHPV genotypes in the quadrivalent and the nonavalent vaccines contributed to 44% and 73% of all cases. Differences were seen in HPV16 across age‐groups (≤30 [39%] vs >30 [25%], *P* = 0.005). At expert revision, 260 (57%) had a confirmed CIN2 diagnosis. The prevalence of hrHPV in the HPV vaccines was higher in expert diagnoses of >CIN2 vs <CIN2 (*P* = 0.001 and *P* = 0.050, respectively).

**Conclusions:** High‐grade cytology and HPV16 are common features in women undergoing active surveillance for CIN2 in Denmark. Further studies should explore combined markers in predicting regression of CIN2.

## Preoperative identification of borderline ovarian tumors using the international ovarian tumor analysis group (IOTA) terminology and ADNEX model

### **Nikoline Schou Karlsen** ^1,2^, Eva Dreisler^1^, Mona Aarenstrup Karlsen^1^, Estrid Høgdall^2^, Claus Høgdall^1^, Abelone Elisabeth Sakse^1^

#### ^1^Gynecologic Department, Rigshospitalet, Copenhagen University Hospital, Copenhagen, Denmark; ^2^Molecular Unit, Department of Pathology, Herlev University Hospital, Herlev, Denmark

**Introduction/Purpose:** Predicting borderline ovarian tumors (BOT) is challenging but essential for planning appropriate management. In Denmark, the Risk of Malignancy Index (RMI) is the gold standard for predicting malignancy. We aimed to evaluate the International Ovarian Tumor Analysis (IOTA) standardized terminology and the predicted risk of malignancy using the The Assessment of Different NEoplasias in the adneXa (ADNEX) model in BOT.

**Methods:** From January 2020, patients ≥18 years with ovarian lesions were prospectively included at the Department of Gynecology, Rigshospitalet, Denmark. Gynecologists described lesions using the IOTA terminology and a template developed in the electronic patient file system. Clinical decisions were not based on the IOTA scores.

**Results:** A total of 47 patients with histologically verified BOT were included (89.4% stage I, 10.6% stage II–III). Median age was 54 years (range 21–82). RMI was ≥200 in 29 (61.7%). PET/CT was performed in 36 (79.6%) and concluded suspicion of malignancy in 18/36 (50%) (FDG uptake in 15 or suspicious CT in 3). Thus, malignancy was suspected in 18 (38.3%) and benign disease in 29 (61.7%) women preoperatively. A total of 10 (21.3%) women had to undergo secondary staging surgery. Calculating the risk of malignancy using the ADNEX model, a total of 41/47 (87.2%) had a malignancy risk >10%. All 6/47 (10.6%) with malignancy risk <10% had uni‐/multilocular lesions (<10 locules), two with diameter >100 mm.

**Conclusions:** Ultrasound is a valuable tool for recognizing BOT, and the ADNEX model was superior to RMI in preoperative risk assessment.

## 8. OTHER

## Resuscitative endovascular balloon occlusion of the aorta (REBOA) as adjunct treatment in life‐threatening postpartum hemorrhage

### Knut Haakon Stensaeth^1,2^, Marte Irene Skille Carlsen^3^, Ylva Haig^2^, Tone Shetelig Lovvik^1,2^, **Edmund Sovik** ^3^

#### ^1^Department of Obstetrics and Gynecology, St. Olavs Hospital, Trondheim, Norway; ^2^University of Science and Technology, Trondheim, Norway; ^3^Department of Anesthesia and Intensive Care, St. Olav’s University Hospital, Trondheim, Norway

**Introduction/Purpose:** Postpartum hemorrhage (PPH) remains a global health problem, responsible for 8% of maternal deaths. Resuscitative Endovascular Balloon Occlusion of the Aorta (REBOA) has been established as a novel treatment for hemorrhagic shock. We present our Norwegian experience of REBOA as an adjunct treatment in life‐threatening PPH.

**Methods:** In 2008, an ‘aortic occlusion kit’ was assembled in three Norwegian university hospitals, and in 2016, devices with enhanced safety profile were introduced. In cases of life‐threatening PPH, and concomitantly with other clinical interventions, REBOA has been implemented by the on‐call interventional radiologist. We evaluated the clinical effects as we have gained experience with the procedure.

**Results:** Our series include 64 patients with adjunct REBOA for life‐threatening PPH. The majority was due to uterus atony, and no procedure‐related complications have been observed in recent years (2016–2021). Compared with the first period (2008–2015), there was a significant reduction of hysterectomies performed (47% vs 24%), total bleeding (5.2 vs 3.7 L), blood transfusions (SAGMAN) used (9 vs 6 units) and uterine artery embolizations performed (17 vs 3). All hysterectomies (since 2016, *n* = 7) were performed due to known pathologic uterus conditions.**Conclusions:** Our Norwegian experience indicates that REBOA for controlling life‐threatening PPH is a safe interventional technique that can be performed with a high degree of technical success, as an adjunct treatment to rapidly achieve hemodynamic stability, and to preserve the reproductive function of the patient. In addition, a close multidisciplinary collaboration seems to be essential to achieve optimal results.

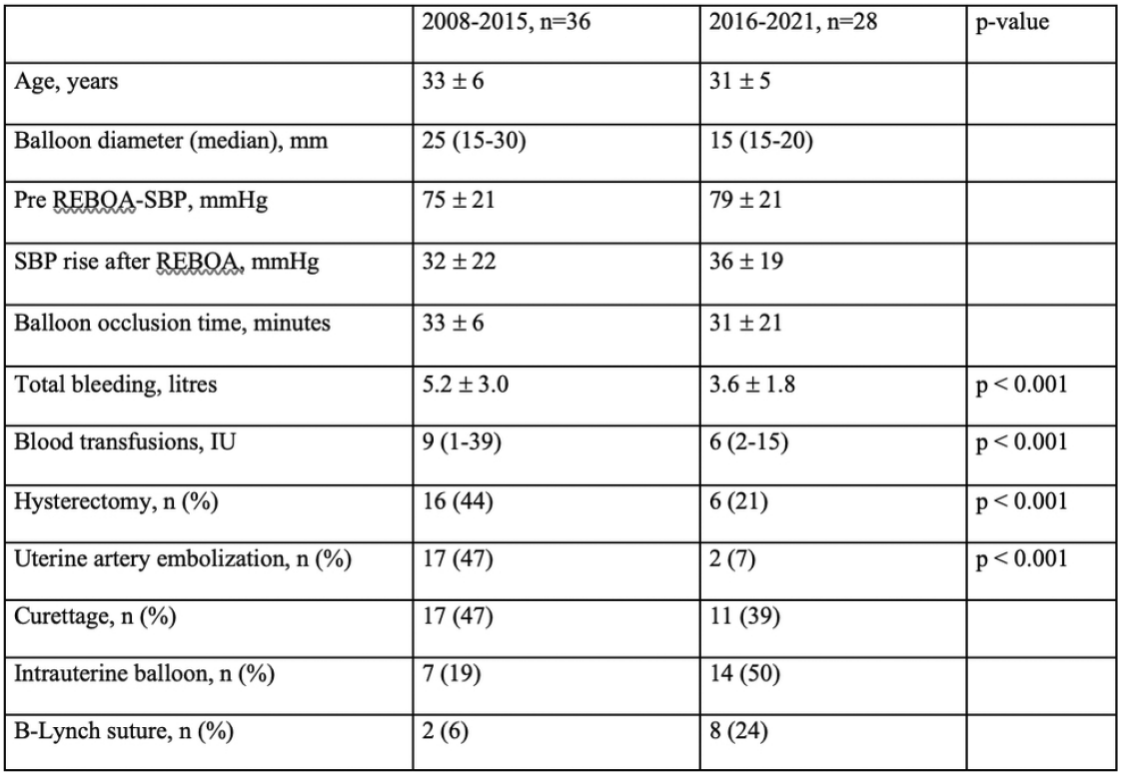

## The dose–response relationship of premenopausal alcohol consumption with age at menopause: a population study of 280 497 women in Norway

### **Julie Røgler Langås** ^1^, Anne Eskild^2,3^, Solveig Hofvind^4,5^, Elisabeth Krefting Bjelland^1,2^

#### ^1^Department of Rehabilitation Science and Health Technology, Oslo Metropolitan University, Oslo, Norway; ^2^Department of Obstetrics and Gynecology, Akershus University Hospital, Lørenskog, Norway; ^3^Institute of Clinical Medicine, Campus Ahus, University of Oslo, Lørenskog, Norway; ^4^Section of Mammographic Screening, Cancer Registry of Norway, Majorstuen, Oslo, Norway; ^5^Department of Health and Care Sciences, Faculty of Health Sciences, The Arctic University of Norway, Langnes, Tromsø, Norway

**Introduction/Purpose:** Previous research suggests that alcohol consumption is associated with high age at menopause. Yet, knowledge about the dose–response relationship is inconsistent. Therefore, we studied the pattern of the association of premenopausal alcohol consumption with age at natural menopause.

**Methods:** We performed a retrospective population‐based study using self‐reported data from 280 497 women, aged 50–69 years, attending the Norwegian breast cancer screening program (BreastScreen Norway) during 2006–2015. Associations of weekly alcohol consumption between the age of 20 and 49 years with age at menopause were estimated as hazard ratios (HR) using Cox proportional hazard models with restricted cubic splines to allow for non‐linear associations. We adjusted for year and place of birth, number of childbirths, educational level, body mass index and smoking habits.

**Results:** Mean age at natural menopause was 51.20 years (interquartile range 49–54 years). The adjusted HR of reaching menopause was highest for women consuming no alcohol (reference), and the HR decreased with alcohol consumption up to 50 g per week (adjusted HR 0.87, 95% confidence interval [CI] 0.86–0.88). Above 50 g, there was no further decrease in the HR of reaching menopause (*P* for non‐linearity <0.001).

**Conclusions:** Women who did not consume alcohol were youngest at menopause. Among alcohol consumers there was no dose–reponse association with age at menopause, indicating that alcohol consumption is not causally related to age at menopause. Our findings may suggest that characteristics of the women who did not consume alcohol, not accounted for in the data analyses, may explain their younger age at menopause.

## European population‐based surveillance of COVID‐19 in pregnancy – The INOSS network

### **Hilde Engjom** ^1,2^, Odette De Bruin^3^, Rema Ramakrishnan^2^, Serena Donati^4^, Marian Knight^2^, Catherine Deneux‐Tharaux^5^ , Outi Äyräs^6^, Anna Aabakke^7,8^, Eva Jónasdóttir^9^, Teresia Svanvik^10^, An Vercoutere^11^, Kitty Bloemenkamp^3^

#### ^1^Division of Mental and Physical Health, The Norwegian Institute of Public Health, Oslo, Norway; ^2^National Perinatal Epidemiology Unit, Oxford Department of Population Health, University of Oxford, Oxford, UK; ^3^Department of Obstetrics, Division of Women and Baby, Wilhelmina Children's Hospital, University Medical Center, Utrecht, Utrecht, The Netherlands; ^4^National Center for Disease Prevention and Health Promotion, Istituto Superiore di Sanità – Italian National Institute of Health, Rome, Italy; ^5^National Institute of Health and Medical Research (INSERM), Paris, France; ^6^Department of Obstetrics and Gynecology, Helsinki University Hospital and University of Helsinki, Helsinki, Finland; ^7^Department of Obstetrics and Gynecology, Copenhagen University Hospital‐Holbæk, Holbæk, Denmark; ^8^Department of Obstetrics and Gynecology, Copenhagen University Hospital‐Nordsjælland‐Hillerød, Hillerød, Denmark; ^9^Department of Obstetrics and Gynecology, Landspitali University Hospital, Reykjavik, Iceland; ^10^Region Västra Götaland, Sahlgrenska University Hospital, Department of Obstetrics and Gynecology, Gothenburg, Sweden; ^11^Department of Obstetrics and Gynecology, CUB Hôpital Erasme, Hôpital Universitaire de Bruxelles (H.U.B.), Université Libre de Bruxelles (ULB), Brussels, Belgium

**Introduction/Purpose:** The aim was to assess incidence of COVID‐19‐related admissions for pregnant women, the severity of COVID‐19 disease and the medical treatment provided to pregnant women with moderate/severe COVID‐19.

**Methods:** Meta‐analysis of population‐based cohort studies from 10 European countries using the INOSS case report form and aligned definitions for national or regional surveillance from March 2020 to December 2020. The wild type/Wuhan was dominant variant.

Inclusion: positive SARS‐CoV‐2 pcr test ≤7 days before hospital admission, during admission or up to 2 days after giving birth. Women were categorized by admission due to COVID‐19/symptoms, or due to labor/obstetric/asymptomatic.

Main outcomes: moderate/severe covid‐19 disease; maternal death, intensive care, or need for respiratory support. COVID‐19 specific medical treatment.

**Results:** Among 1.7 million maternities 8983 women were included; 2398 were admitted due to COVID‐19/symptomatic. The estimated incidence of admission due to COVID‐19 per 1000 maternities was 0.77 (0.49–1.19), ranging from no admissions in Iceland to 1.78 and 1.89 in France and the UK. Among women admitted due to COVID‐19 in countries with complete respiratory support information 822 women (39.5%, 34.6–44.4) had moderate/severe COVID‐19. Only one in five women with moderate/severe COVID‐19 received COVID‐19 specific medical treatment, although two of three were given thromboprophylaxis.

**Conclusions:** Population‐based surveillance in 10 European countries show striking variations in pregnant womens’ risk of COVID‐19 admission, indicating the unaddressed role played by “suppression” public health policies for protecting pregnant women. Undertreatment of pregnant women is a continuing concern requiring future priority. Investment in rapid and robust surveillance is essential.

## Challenges imposed by the COVID‐19 pandemic on the obstetrics and gynecology residency program

### Matilda Wådell^1^, Anne Örtqvist^2,3^, Karolina Linden^4^, Magnus Åkerström^5,6^, Ola Andersson^7,8^, Ylva Carlsson^9,10^, Sofie Graner^2,11^, Maria Jonsson^12,13^, Elin Naurin^14^, Verena Sengpiel^9,10^, Malin Veje^15,16^, Anna Wessberg^9^, **Mehreen Zaigham** ^17,18^

#### ^1^Department of Obstetrics and Gynecology, Hudiksvall Hospital, Hudiksvall, Sweden; ^2^Clinical Epidemiology Unit, Department of Medicine, Karolinska Institutet, Stockholm, Sweden; ^3^Department of Obstetrics and Gynecology, Visby County Hospital, Visby, Sweden; ^4^Institute of Health and Care Sciences, Sahlgrenska Academy, University of Gothenburg, Gothenburg, Sweden; ^5^Region Västra Götaland, The Institute of Stress Medicine, Gothenburg, Sweden; ^6^School of Public Health and Community Medicine, Institute of Medicine, Sahlgrenska Academy, University of Gothenburg, Gothenburg, Sweden; ^7^Pediatrics, Institution of Clinical Sciences Lund, Lund University, Lund, Sweden; ^8^Department of Neonatology, Skåne University Hospital, Malmö, Sweden; ^9^Region Västra Götaland, Department of Obstetrics and Gynecology, Sahlgrenska University Hospital, Gothenburg, Sweden; ^10^Center of Perinatal Medicine and Health, Institute of Clinical Sciences, Sahlgrenska Academy, University of Gothenburg, Gothenburg, Sweden; ^11^ BB Stockholm, Danderyd Hospital, Danderyd, Sweden; ^12^Department of Women's and Children's Health, Uppsala University, Uppsala, Sweden; ^13^Department of Obstetrics and Gynecology, Uppsala University Hospital, Uppsala, Sweden; ^14^Department of Political Science, University of Gothenburg, Gothenburg, Sweden; ^15^Institute of Biomedicine, Department of Infectious Diseases, University of Gothenburg, Gothenburg, Sweden; ^16^Region Västra Götaland, Department of Infectious Diseases, Sahlgrenska University Hospital, Gothenburg, Sweden; ^17^Obstetrics and Gynecology, Institution of Clinical Sciences Lund, Lund University, Lund, Sweden; ^18^Department of Obstetrics & Gynecology, Lund University and Skåne University Hospital, Malmö, Sweden

**Introduction/Purpose:** Our aim was to study how the training program and work situation of residents in Obstetrics and Gynecology (OB‐GYN) was affected by the COVID‐19 pandemic and illuminate how residents experienced these changes in Sweden.

**Methods:** As part of the COVID‐19 in Pregnancy and Early Childhood Staff (COPE Staff) study, between January and May 2021, all participating residents were invited to answer a 28‐question online Resident Survey focusing on their specialist education, work situation and experiences during the COVID‐19 pandemic. Free text responses were analyzed by content analysis.

**Results:** Of the 162 participating residents, 69% expressed concern that the pandemic would have a negative impact on their training. Ninety‐five reported cancellation of educational activities, 70% performed fewer surgeries and 27% had been transferred to other healthcare institutions. Working extra clinical hours was reported by 69% (7.4 ± 5.3 hours per week) and 14% had considered changing their profession due to the pandemic. Senior residents, compared with junior residents, more often experienced canceled/postponed clinical rotations (30% vs 15%, *P* = 0.02) and reported performing fewer surgeries (*P* = 0.02). The qualitative analysis highlighted the lack of surgical procedural training as a major concern for residents.**Conclusions:** The COVID‐19 pandemic has strongly impacted the training program and work situation of OB‐GYN residents in Sweden. Residents were concerned over the negative impact on their training program and senior residents reported more missed educational opportunities as compared with junior residents. Program directors and clinical supervisors can use the problem areas pinpointed by this study to support residents and compensate for missed educational opportunities.

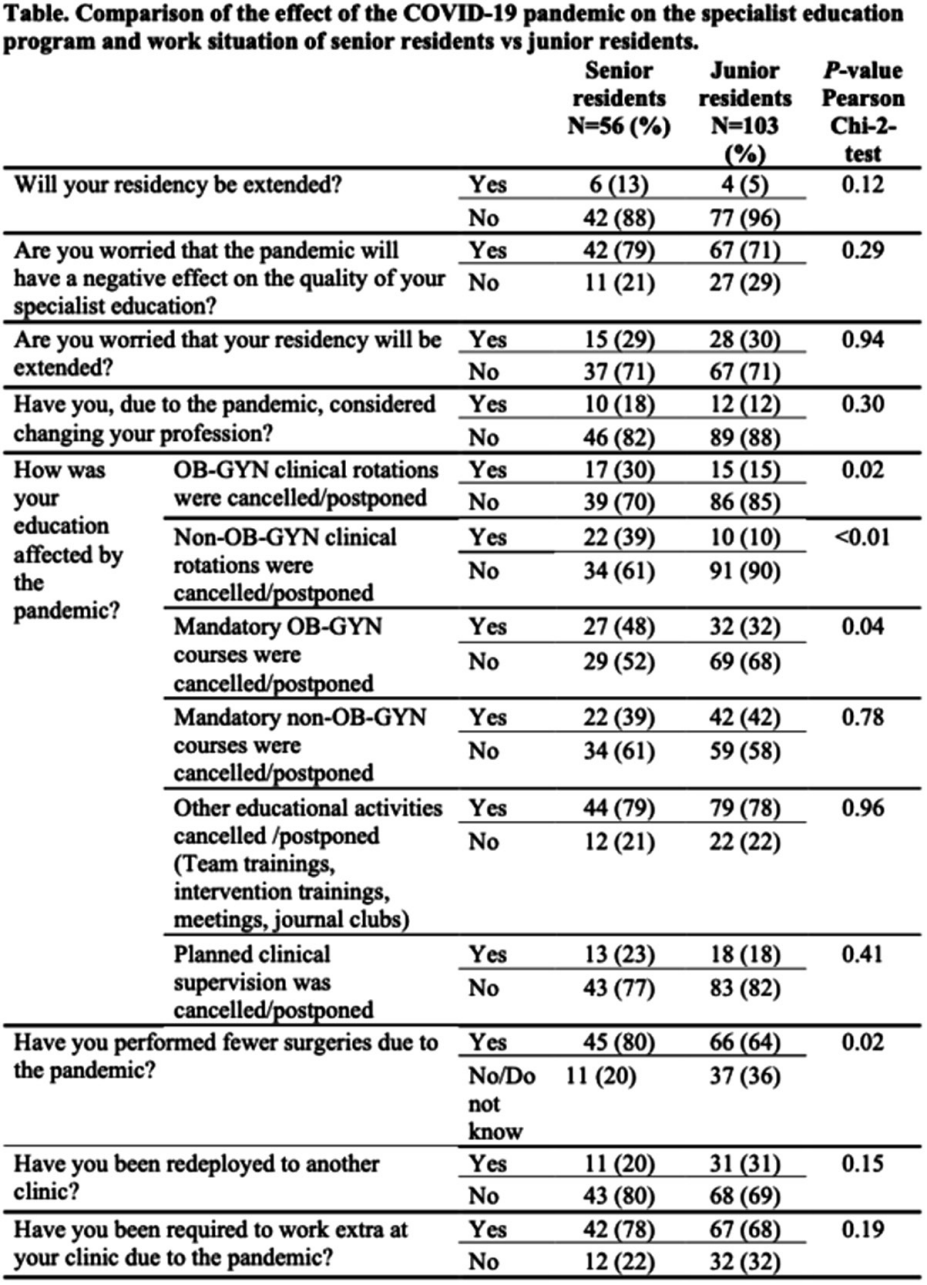

## Experience from HPV self‐sampling as part of a population based cervical cancer screening program in the region of Stockholm, Sweden

### **Kristina Elfgren** ^1,2^, Malin Vikström^1^, Daniel Öhman^1^, Katarina Lannervall^1^, Viveka Lundström^1^, Miriam Elfström^1,3^, Joakim Dillner^1,3^, Nathalie Roos^1,4^

#### ^1^Department for Screening and Prevention, Regional Cancer Centrum Stockholm Gotland, Stockholm, Sweden; ^2^Department of Gynecology and Reproductive Medicine, Clintec, Karolinska Institutet, Stockholm Sweden; ^3^Karolinska University Hospital, Department of Laboratory Medicine, Stockholm, Sweden; ^4^Clinical Epidemiology Unit, Department of Medicine, Karolinska Institutet, Stockholm, Sweden

**Introduction/Purpose:** During the COVID‐19 pandemic, the cervical cancer screening program was paused for 9 months as part of the public health restrictions. To compensate for the screening gap in the Region of Stockholm, Sweden, the Swedish Board of Health and Welfare allowed for a temporary transition from provider‐taken liquid‐based cytology (LBC) with analysis of HPV and reflex cytology, to HPV self‐sampling in 2021. There is a paucity in data globally regarding participation rates using HPV self‐sampling and an opt‐out approach as the main screening strategy among participating women in the cervical cancer screening program.

**Methods:** Descriptive statistics from the cervical cancer screening program compared participation rate by HPV self‐sampling strategy during March–December 2021 and provider‐taken LBC during 2018.

**Results:** During 2021, a total of 355 396 HPV self‐sampling kits were sent by mail to eligible women of screening age in the Region of Stockholm. By comparison, in 2018 we invited 258 816 women for provider‐taken LBC with HPV analysis and reflex cytology. The participation rate was significantly higher with HPV self‐sampling strategy (51% vs 38%; *P* < 0.0001). The largest increase in participation rate was among women 26–29 years (38% vs 54%; *P* < 0.0001) and 30–39 years (42% vs 53%; *P* < 0.0001). The participation rate among non‐responding women (defined as ≥4 screening invites), doubled with HPV self‐sampling (12% vs 23%; *P* < 0.0001). The participation in follow‐up LBC among HPV‐positive women after self‐sampling was 90%. The time span from HPV self‐sample and appointment for a provider‐taken follow‐up LBC was 3 months as per protocol.

**Conclusions:** Screening by HPV self‐sampling in the Region of Stockholm has increased the overall participation rate in the cervical cancer screening program, especially among younger women and non‐responding women. However, follow‐up testing among HPV‐positive women is crucial for early detection of pre‐cancerous lesions.

## Facilitating gynecological examination and long‐acting contraception for women with substance use disorder

### **Trine Finanger** ^1,2^, Ragnhild Skråstad^2,3^, Catherine Appleton^4,5^, Cecilie Hagemann^2,6^

#### ^1^Clinic of Substance Use and Addiction Medicine, St. Olav's University Hospital, Trondheim, Norway; ^2^Department of Clinical and Molecular Medicine, Norwegian University of Science and Technology – NTNU, Trondheim, Norway; ^3^Department of Clinical Pharmacology, St. Olav's University Hospital, Trondheim, Norway; ^4^Center for Research and Education in Security, Prisons and Forensic Psychiatry, St. Olav's University Hospital, Trondheim, Norway; ^5^Department of Mental Health, Norwegian University of Science and Technology – NTNU, Trondheim, Norway; ^6^Department of Obstetrics and Gynecology, St. Olav's University Hospital, Trondheim, Norway

**Introduction/Purpose:** Unplanned pregnancies are more frequent among women with substance use disorder (SUD), and the recommended contraceptives are intrauterine devices (IUD) or implants, such as Long Acting Contraceptives (LARCs). Women with SUD have a higher frequency of induced abortion, greater experience of sexual assault and abuse, and a higher incidence of cervical pathology. This study aimed to evaluate the patient‐reported outcomes of a facilitated gynecological service that was free of charge, addressing testing and LARC insertion for women with SUD.

**Methods:** This study recruited 48 women who were receiving treatment for their substance use in the region of Trondheim, Norway. They received a gynecological examination at the women's department's outpatient clinic by a consultant. ThinPrep Pap Test and microbiological samples were collected, and they were offered contraception, with an emphasis on LARC, free of charge.

**Results:** Half of the women consented to having an LARC, and one‐third had a pathological cervical cytology result requiring treatment or follow‐up. The patients’ self‐reported satisfaction with the service was more than 95%.

**Conclusions:** For women with SUD, a facilitated gynecological service has the potential to reduce the incidence of unplanned pregnancies by increasing the use of LARC, and also increasing the adherence to an important cervical cytology screening program that may be out of reach for women with SUD. The findings show health benefits for women with SUD as well as usefulness from a broader socioeconomic perspective. In addition, the women in the study expressed great satisfaction with the service.

## Improving patient recruitment into clinical trials

### **Silke Rossau** ^1^, Anna Louise Christ Vestergaard^1,2^, Agnete Larsen^2,3^, Pia Kirkegaard^4^, Mette Tranberg^4^, Pinar Bor^1,2^

#### ^1^Department of Gynecology and Obstetrics, Randers Regional Hospital, Randers, Denmark; ^2^Department of Clinical Medicine, Aarhus University, Aarhus, Denmark; ^3^Department of Biomedicine, Aarhus University, Aarhus, Denmark; ^4^University Research Clinic for Cancer Screening, Randers Regional Hospital, Randers, Denmark

**Introduction/Purpose:** Women have historically been underrepresented in clinical trials, with biological implications and inferior applicability of the results as a consequence. Underrepresentation contributes to the inequality in health which has come into focus in recent years. To ensure proper evidence‐based screening, diagnostics and treatment it is essential to conduct research with a high and balanced representation of the at‐risk population within the given disease. In this study we aim to investigate how recruitment of women into clinical trials can be improved, related to obstetrics and gynecology.

**Methods:** Prospective questionnaire survey and focus‐group interviews are conducted. All women who are eligible to participate in a clinical trial at Department of Obstetrics and Gynecology, Randers Regional Hospital, are invited to participate. The questionnaire investigates motives for non‐participation and what might encourage future participation. Nine focus‐group interviews, with four to six women in each focus‐group, are conducted and contribute with a thorough exploration of the mechanisms behind non‐participation, general knowledge on and attitude towards participation in clinical trials.

**Results:** Ongoing study. The primary outcome is the reasons for participation and non‐participation. The secondary outcome is characterization of non‐participants.

**Conclusions:** Our study will provide data on how to improve patient recruitment into clinical trials. It will contribute to an improved recruiting process with higher inclusion rates and shorter inclusion time, help reduce underrepresentation and improve patient involvement in research in general. An improved participation in clinical trials will also result in improved screening, diagnostics and treatment for patients in daily clinical practice.

## Lessons from national surveillance of COVID‐19 in pregnancy in Norway

### **Hilde Engjom** ^1,2^, Lill Trine Nyfløt^3,4^, Pétur Júlíusson^1^, Siri Vangen^3^, Kari Klungsøyr^1^

#### ^1^Department for Health Registry Research and Development, Norwegian Institute of Public Health, Bergen, Norway; ^2^Department of Obstetrics and Gynecology, Haukeland University Hospital, Bergen, Norway; ^3^Norwegian Center for Research on Womens’ Health, Oslo University Hospital, Oslo, Norway; ^4^Department of Obstetrics and Gynecology, Vestre Viken Hospital Drammen, Drammen, Norway

**Introduction/Purpose:** Based on increased maternal and perinatal risk in the previous H1N1 pandemic, the Norwegian Medical Birth Registry established targeted surveillance of pregnant women with coronavirus infection admitted to hospital from March 1, 2020. Suppression was implemented as a national public health strategy from March 12, 2020 until September 2021.

**Methods:** National surveillance from March 12, 2020 to December 31, 2021 at the Medical Births Registry of Norway. The source population was 103 323 women giving birth in this period. Cases were pregnant women with SARS‐CoV‐2 infection admitted to hospital, and infection was defined as a positive SARS‐CoV‐2 PCR test ≤14 days before admission, during admission or up to 2 days after birth. Cause of admission was categorized as due to COVID‐19 or other causes.

**Results:** In total, 372 pregnant women with coronavirus infection were admitted; 76 due to COVID‐19, 296 for other care (mostly labor/obstetric care). The incidence of admission due to COVID‐19 per 1000 maternities was 0.2, 0.8 and 1.5 in the wild type, alpha and delta dominant periods, respectively. No women needed intensive care in the wild type period but 40 women needed respiratory support during the alpha and delta periods; all were unvaccinated. Severe fetal or neonatal outcomes due to COVID were rare, but occurred during the alpha and delta periods to unvaccinated women.

**Conclusions:** The incidence of admission due to COVID‐19 was low in Norway but increased after national public health policies were eased in September 2021. Standardized collection of clinical information enables monitoring of treatment and avoiding misclassification errors.

## Restoration of microbiota in newborns (ROMANS) – a randomized placebo‐controlled multicenter allergy prevention study

### **Emma Fransson** ^1,2^, Marica Hamsten^1^, Maria Jonsson^2^, Christina West^3^, Maria Jenmalm^4^, Lars Engstrand^1^

#### ^1^Center for Translational Microbiome Research, Department of Microbiology, Tumor and Cell Biology (MTC), Karolinska Institutet, Solna, Sweden; ^2^Department of Women's and Children's Health, Uppsala University, Uppsala, Sweden; ^3^Department of Clinical Sciences, Pediatrics, Umeå University, Umeå, Sweden; ^4^Division of Inflammation and Infection, Department of Biomedical and Clinical Sciences, Linköping University, Linköping, Sweden

**Introduction/Purpose:** Every third child in Sweden is afflicted with allergic disease. Infants delivered by cesarean section (CS) are at increased allergy risk, possibly due to delayed and aberrant gut colonization influencing immune system development. This multicenter randomized placebo‐controlled trial aims to study whether restoration of the microbiota via smearing the neonate with maternal vaginal and fecal bacteria, could attenuate the allergy risk in infants delivered by CS. The primary outcome is immunoglobulin E (IgE)‐associated allergic disease at 24 months of age.

**Methods:** In January 2023, 117 mother–infant pairs with elective CS (of a planned 220) have been included, screened for potential pathogens, and randomized to active treatment or placebo. For a vaginal delivery control group, 129 mother–infant pairs are included. Multiple samples, to be analyzed for microbial genes and immune markers, have been collected from mother and infant from pregnancy to a child age of 24 months. The study has been approved by the Swedish Ethical Review Authority.**esults:** During the spring of 2023 we are conducted an initial investigation, comparing the proportion of maternal microbial genes in the child's gut and oral microbiome at ages 1 month, 6 and 24 months, across the three groups.

**Conclusions:** This is the first study to investigate smearing CS‐born infants with both vaginal and fecal bacteria from the mother. The safety and clinical effects of the practice still need to be evaluated; vaginal seeding is already increasingly practiced globally. The results are important to inform healthcare on how to potentially decrease allergy risk in CS‐born infants.

## Team‐based learning (TBL) in clinical disciplines for undergraduate medical students – a scoping review

### Irene Sterpu^1^, Lotta Herling^1,2^, Jonas Nordqvist^3^, Jerome Rotgens^3^, **Ganesh Acharya** ^1^

#### ^1^Division of Obstetrics and Gynecology, Department of Clinical Sciences, Intervention and Technology (CLINTEC), Karolinska Institutet, Stockholm, Sweden; ^2^Center for Fetal Medicine, Karolinska Hospital Huddinge, Huddinge, Sweden; ^3^Department of Medicine (Huddinge) Karolinska Institutet, Stockholm, Sweden

**Introduction/Purpose:** There are a growing number of medical schools that are adopting TBL. However, so far it has been mainly implemented in preclinical disciplines. TBL has been rarely implemented throughout an entire medical university within all disciplines. The aim of this study was identify in which clinical disciplines TBL was implemented and how it was implemented.

**Methods:** The scoping review was conducted according to guidance document from the Joanna Briggs Institute and will be reported according to PRISMA extensions for scoping review. Electronic databases MEDLINE, PubMed, Web of Science and Eric were searched.

**Results:** A total of 1452 articles on TBL was identified, of which 241 studies were from undergraduate preclinical disciplines and 39 studies from clinical disciplines. In all, 51% of all studies on TBL in clinical disciplines were published in USA, 36% in Asia and only 12% in Europe. Among the disciplines where TBL was implemented there were neurology (eight studies), gynecology (three studies), ophthalmology (three studies), psychiatry (three studies). A modified TBL was used in 94% of all studies, where one or more parts of the TBL process were missing.

**Conclusions:** There is a knowledge gap in the literature concerning the benefit of TBL in clinical disciplines. A modified TBL was mostly applied and with different or no comparators. The most often reported outcomes were student satisfaction, student engagement and knowledge assessment. Most of the studies reported a higher student engagement and satisfaction with the TBL. These findings were not significantly correlated with improved academic performance. The majority of the studies are descriptive and more rigorous studies are needed.

## Team‐based learning in obstetrics and gynecology

### **Irene Sterpu** ^1^, Lotta Herling^1,2^, Jonas Nordqvist^3^, Jerome Rotgens^3^, Ganesh Acharya^1^

#### ^1^Division of Obstetrics and Gynecology, Department of Clinical Sciences, Intervention and Technology (CLINTEC), Karolinska Institutet, Stockholm, Sweden; ^2^Center for Fetal Medicine, Karolinska Hospital Huddinge, Huddinge, Sweden; ^3^Department of Medicine (Huddinge) Karolinska Institutet, Stockholm, Sweden

**Introduction/Purpose:** With more and more focus on active learning, methods such as team‐based learning (TBL) are rapidly gaining popularity in medical education. Although there are many studies on the benefits of TBL in preclinical disciplines, the results are not as convincing in the clinical disciplines. Our aim was to compare the effect of TBL with tradionally small group seminars in the obstetrics and gynecology clerkship for undergraduate medical students.

**Methods:** Fifth year medical students (*n* = 132) were distributed into two groups. The study was designed as a prospective, crossover study with randomly distribution of the seminars.

Knowledge was assessed by iRAT, tRAT and final exam scores. Student satisfaction and engagement were assessed by a 14‐item questionnaire and one summary question.

**Results:** The initial groups of 10 students in each seminar were increased to 20 students in the TBL sessions. No difference in student active learning and student satisfaction was seen between the groups. The analysis of the data on knowledge acquisition is ongoing and will be ready shortly. Both seminars were equally highly rated by the students.

**Conclusions:** There was no difference in student satisfaction and student engagement between the groups, even though the student:teacher ratio was 10:1 in the traditionally seminars and 20:1 in the TBL sessions. This suggests that TBL can be particularly advantageous, with fewer faculty members needed for the seminars.

## The criminal procedure in rape cases regarding clients of the Helsinki Seri Support Center, Finland

### **Riina Korjamo** ^1,2^, Jenni Krogell^3,4^, Saara Asmundela^5^, Yrjö Reenilä^6^

#### ^1^Helsinki University Hospital, Department of Obstetrics and Gynecology, Seri Support Center, Helsinki, Finland; ^2^University of Helsinki, Helsinki, Finland; ^3^University of Eastern Finland, Kuopio, Finland; ^4^Finnish National Institute for Health and Welfare, Helsinki, Finland; ^5^Central Finland Police Department; ^6^National Prosecution Authority, Prosecution District of Southern Finland, Helsinki, Finland

**Introduction/Purpose:** We examined how the criminal procedures regarding clients of the Helsinki Seri Support Center (sexual assault center), Finland, progress.

**Methods:** This retrospective register‐based study examined the clients of Helsinki Seri Support Center. Their first visit following the rape was between June 1, 2017 and May 31, 2019; the assaults had been reported to the police before May 31, 2020. Eligible clients were ≥16 years old and had experienced sexual assault within 1 month before the visit. Demographics, information on the assaults and medical statements were collected from the medical records. Progression information in the criminal procedure were collected from the registers of the police, the national prosecution authority, the district courts and the court of appeal.

**Results:** During the study period, 688 visits were recorded, and 74% of the cases were reported to the police. Median time from the report to the end of pre‐trial investigation was 5 months, to the decision to bring the charges 13 months, and to the conviction in the district court 1 year 9 months. Forensic medical samples were used as evidence in the court in 44%, pictures of injuries in 47%, and forensic medical statement in 92% of cases.

**Conclusions:** Criminal procedures in rape cases are long‐lasting, which challenges the support services helping the rape victims. Forensic medical examination, including collected evidence, and medical statements composed by doctors working in the sexual assault center, play an important role in the criminal process. Knowledge of the criminal procedure will help providers to correctly support rape victims.

## The EIR‐study pilot: can we prevent posttraumatic stress after rape?

### **Tina Haugen** ^1,2,3^, Joar Halvorsen^2,3^, Berit Schei^4,5^, Paul Mork^5^, Oddgeir Friborg^6^, Cecilie Hagemann^1,4^

#### ^1^Department of Clinical and Molecular Medicine (IKOM), Faculty of Medicine and Health Sciences (MH faculty), Norwegian University of Science and Technology (NTNU), Trondheim, Norway; ^2^Nidaros District Psychiatric Hospital, St. Olav's Hospital, Trondheim, Norway; ^3^Department of Psychology, NTNU, Trondheim, Norway; ^4^Department of Obstetrics and Gynecology, St. Olav's Hospital, Trondheim, Norway; ^5^Department of Public Health and Nursing (ISM), Faculty of Medicine and Health Sciences (MH faculty), NTNU, Trondheim, Norway; ^6^Department of Psychology, Arctic University of Norway, Tromsø, Norway

**Introduction/Purpose:** Early Intervention after Rape (The EIR‐study) is a randomized multicenter trial comparing modified Prolonged Exposure Therapy (mPE) with treatment as usual (TAU). Previous trials have indicated that mPE can be an effective intervention to prevent the development of posttraumatic stress symptoms (PTSS) especially for those who have experienced rape.

**Methods:** Participants seeking assistance at three SACs within 72 houra after rape, were screened for eligibility and randomized to either a maximum of five sessions with mPE or TAU. The objectives are to test the feasibility of the protocol by investigating predefined criteria.

**Results:** Three SACs were recruited. Of the 235 patients attending the SACs, 41 were eligible, but only 22 (9%) consented to participate. Eight were allocated to mPE and 14 to TAU.

The mPE group had a mean of 2.8 consultations and the TAU group a mean of 4.5 consultations.

No adverse events were reported related to the intervention, leading to the preliminary conclusion that the intervention is safe.

Completion of questionnaires: 15 of the 22 participants completed the 6‐week post‐test measurements. There was no missing data on primary outcomes.

Feasibility of biological measurements: At baseline, all participants consented to give hair sample. Four participants collected all nine saliva cortisol samples correctly.

Physical activity and sleep were measured using accelerometers attached to the thigh and lower back. Except for two participants, all consented to wear the accelerometers for 7 days at baseline.

**Conclusions:** Progression criteria was met. Results from the pilot study support the feasibility of a full‐scale RCT.

## The implementation of a national recommendation concerning obstetric and neonatal emergency training in Finnish maternity hospitals

### Marja Kaijomaa^1^, Irmeli Nupponen^1^, Ulla Sankilampi^2^, **Terhi Saisto** ^1^, Hilkka Nikkinen^3^, Ilkka Ketola^1^

#### ^1^Helsinki University Hospital, Helsinki, Finland; ^2^Kuopio University Hospital, Kuopio, Finland; ^3^Oulu University Hospital, Oulu, Finland

**Introduction/Purpose:** Patient safety is the priority of maternity care, but the preparedness to face emergencies may be weak if emergency care routine and regular training are missing. In 2020, most Finnish hospitals (16/23) had less than 2000 deliveries, but the number varied from 243 to 8555.

To ensure patient safety in all hospitals, a quality project conducted by the Finnish Perinatal Society was launched.

**Methods:** National obstetric and neonatal working groups were set to harmonize the status of maternity hospital emergency training. The work was done between September 2020 and April 2021. After a survey concerning the current training status, patient safety‐centered goals were set for personnel training. The ultimate goal was the preparation and implementation of a national recommendation concerning obstetric and neonatal emergency training.

**Results:** Significant differences in the emergency training were identified between hospitals. A national recommendation concerning trainer education, target groups, quality, quantity and content of training was given in 2021. The importance of regular simulation trainings and skills stations together with the training of non‐technical skills and teamwork was emphasized. Comments from the patient associations and the Ministry of Social Affairs and Health were considered in the final summary. The importance of regional training collaboration with university hospitals was highlighted. Based on an inquiry made in 2023, the recommended simulation and skills station trainings were utilized in 19/23 hospitals.

**Conclusions:** As a result of national collaboration, a freely available and patient safety‐centered national recommendation concerning emergency training in maternity hospitals was made and successfully implemented.

## Women's negative childbirth experiences and socioeconomic factors: results from The Babies Born Better survey

### Carina Vedeler^1^, Tine Schauer Eri^1^, Roy Miodini Nilsen^2^, Ellen Blix^1^, Soo Downe^3^, Kjetil A. van der Wel^1^, **Anne Britt Vika Nilsen** ^2^

#### ^1^Oslo Metropolitan University, Oslo, Norway; ^2^Western Norway University of Applied Sciences, Bergen, Norway; ^3^University of Central Lancashire, Preston, UK

**Introduction/Purpose:** To investigate the association between women's socioeconomic status and overall childbirth experience and to explore how women reporting an overall negative birth experience describe their experiences of intrapartum care.

**Methods:** We used both quantitative and qualitative data from the Babies Born Better (B3) survey version 2, including a total of 8317 women. First, we performed regression analyses to explore the association between women's socioeconomic status and labor and birth experience, and then a thematic analysis of three open‐ended questions from women reporting a negative childbirth experience (*n* = 917).

**Results:** In total 11.7% reported an overall negative labor and birth experience. The adjusted odds ratio (OR) of a negative childbirth experience was elevated for women with non‐tertiary education, for unemployed, students and those not married or cohabiting. Women with lower subjective living standard had an adjusted OR of 1.70 (95% confidence interval [CI] 1.44–2.00) for a negative birth experience, compared with those with average subjective living standard. The qualitative analysis generated three themes: (1) uncompassionate care – lack of sensitivity and empathy, (2) impersonal care – feeling objectified, and (3) critical situations – feeling unsafe and loss of control.

**Conclusions:** Important socioeconomic disparities in women's childbirth experiences exist even in the Norwegian setting. Women reporting a negative childbirth experience described disrespect and mistreatment as well as experiences of insufficient attention and lack of awareness of individual and emotional needs during childbirth. The study shows that women with lower socioeconomic status are more exposed to these types of experiences during labor and birth.

## Phosphatidylethanol (PEth) as a biological marker of alcohol intake in early pregnancy: an observational study in 4067 pregnant women

### **Trine Finanger** ^1,2^, Olav Spigset^2,3^, Rolf Gråwe^4,5^, Trine Naalsund Andreassen^3^, Trine Løkken^3^, Trond Aamo^3^, Guro Bratt^6^, Kristin Tømmervik^1^, Vibeke Langås^7^, Kristin Finserås^7^, Kjell Åsmund Salvesen^2,8^, Ragnhild Skråstad^2,3^

#### ^1^St. Olav's Hospital, Clinic of Substance Use and Addiction Medicine, Trondheim, Norway; ^2^Norwegian University of Science and Technology, Department of Clinical and Molecular Medicine, Trondheim, Norway; ^3^St. Olav's Hospital, Department of Clinical Pharmacology, Trondheim, Norway; ^4^St. Olav's Hospital, Department of Research and Development, Trondheim, Norway; ^5^Norwegian University of Science and Technology, Department of Mental Health, Trondheim, Norway; ^6^St. Olav's Hospital, Department of Laboratory Medicine, Trondheim, Norway; ^7^St. Olav's Hospital, Department of Immunology and Transfusion Medicine, Trondheim, Norway; ^8^St. Olav's Hospital, Department of Obstetrics and Gynecology, Trondheim, Norway

**Introduction/Purpose:** Alcohol use disorder (AUD) is a common mental disorder whose prevalence is increasing. The teratogenic effect of alcohol is well documented, but there is lack of appropriate screening methods to detect alcohol use in pregnancy. PEth (phosphatidylethanol 16:0/18:1) is a specific and sensitive alcohol marker reflecting alcohol intake up to several weeks after consumption. The aim of the study was to explore the potential usefulness of PEth as a marker of prenatal alcohol exposure in a general pregnant population.

**Methods:** A total of 4566 blood samples from 4067 women submitted to St. Olav's Hospital on the indication Rhesus typing in pregnancy between September 2017 and October 2018 were collected. Blood tests for Rhesus typing are performed routinely around gestational week 12 and Rhesus‐negative women had an additional blood test taken in week 24. All samples were analyzed for PEth.

**Results:** A total of 58 women had a positive PEth in pregnancy. Fifty women (1.4%) had positive PEth at the end of their first trimester, three women (0.4%) had positive PEth in the second trimester and for five of the cases the time of sampling was unknown. There were no significant differences in proportion of women with positive PEth values related to age or rural vs urban residency.

**Conclusions:** PEth assessments of a normal pregnant population found that 1.4% had a positive sample at the end of the first trimester. This group of women may benefit from further AUD screening and healthcare services.

